# *Marchantia* liverworts as a *proxy* to plants’ basal microbiomes

**DOI:** 10.1038/s41598-018-31168-0

**Published:** 2018-08-23

**Authors:** Luis D. Alcaraz, Mariana Peimbert, Hugo R. Barajas, Ana E. Dorantes-Acosta, John L. Bowman, Mario A. Arteaga-Vázquez

**Affiliations:** 1 Departamento de Biología Celular, Facultad de Ciencias, Universidad Nacional Autónoma de México, Ciudad Universitaria, UNAM, 04510 Coyoacán, Mexico City Mexico; 2 Departamento de Ciencias Naturales, Universidad Autónoma Metropolitana, Unidad Cuajimalpa, Av. Vasco de Quiroga 4871, Col. Santa Fe Cuajimalpa, 05348 Mexico City, Mexico; 3 University of Veracruz, Institute for Biotechnology and Applied Ecology (INBIOTECA), Avenida de las Culturas Veracruzanas 101, Colonia Emiliano Zapata, 91090 Xalapa, Veracruz Mexico; 4 School of Biological Sciences, Monash University, Melbourne, Victoria, 3800 Australia

## Abstract

Microbiomes influence plant establishment, development, nutrient acquisition, pathogen defense, and health. Plant microbiomes are shaped by interactions between the microbes and a selection process of host plants that distinguishes between pathogens, commensals, symbionts and transient bacteria. In this work, we explore the microbiomes through massive sequencing of the 16S rRNA genes of microbiomes two *Marchantia* species of liverworts. We compared microbiomes from *M*. *polymorpha* and *M*. *paleacea* plants collected in the wild relative to their soils substrates and from plants grown *in vitro* that were established from gemmae obtained from the same populations of wild plants. Our experimental setup allowed identification of microbes found in both native and *in vitro Marchantia* species. The main OTUs (97% identity) in *Marchantia* microbiomes were assigned to the following genera: *Methylobacterium*, *Rhizobium*, *Paenibacillus*, *Lysobacter*, *Pirellula*, *Steroidobacter*, and *Bryobacter*. The assigned genera correspond to bacteria capable of plant-growth promotion, complex exudate degradation, nitrogen fixation, methylotrophs, and disease-suppressive bacteria, all hosted in the relatively simple anatomy of the plant. Based on their long evolutionary history *Marchantia* is a promising model to study not only long-term relationships between plants and their microbes but also the transgenerational contribution of microbiomes to plant development and their response to environmental changes.

## Introduction

All multicellular eukaryotes have a microbiome composed of prokaryotes, primarily bacteria, and eukaryotes, both uni- and multicellular. Species of the microbiome can be commensal, pathogenic, symbiotic, essential, or neutral. Land plants are no exception, with three distinct habitats colonized by microbial species — the rhizosphere underground and at the substrate surface, the phyllosphere on the aerial plant surfaces, and the endosphere within the plant body^1–3^. Land plants evolved from a freshwater algal ancestor and upon the transition to terrestrial habitats was exposed to novel microbial communities. It has long been recognized that early, and perhaps the first, land plants harbored mycorrhizal fungi, perhaps in a symbiotic relationship from the onset^4^. Furthermore, it has been suggested that early land plants may have inherited a microbiome from their algal ancestor, perhaps consisting of nitrogen-fixing bacterial and methanotrophs^5^.

Extant land plants consist of bryophytes (liverworts, mosses, hornworts), a monophyletic or paraphyletic group of gametophyte dominant plants and the monophyletic vascular plants. Bryophytes are largely *poikilohydric* — their water potential is equilibrated rapidly to external water availability. Their gametophyte bodies are in close contact with the substrate surface and ventral rhizoids act in water absorption and distribution. In contrast, the diploid sporophyte dominant vascular plants are homoiohydric, with a root system through which water is obtained. Early land plants established symbiotic associations with mycorrhizae, and it is thought that Palaeozoic drops in CO_2_ reduced phosphate (P) absorption in non-vascular plants which in addition to competition for light favoured the vascular plants Earth dominance^6^.

Extensive sequencing approaches have fostered recent studies on plant-microbe interactions, and plant microbiomes from model plants like *Arabidopsis thaliana* and agriculturally relevant species are publicly available^7–10^, and these have contributed to the study of plant-microbe interactions. Proposed models for the establishment of the plant root microbiome include the two-step model that considers the root microbiome as a product of plant-independent features. The two-step model considers edaphic factors, the general selection of microorganisms for general plant cell wall composition, rhizodeposits and the host genotype which actively selects their microbial inhabitants^11^. While considerable knowledge of vascular plant microbiomes is available, much less is known about the microbiomes of bryophytes, and knowledge is largely limited to mycorrhizal and other fungal interactions.

In contrast to vascular plants that possess roots that penetrate deeply into the soil, sometimes meters, bryophytes adhere to the substratum via rhizoids, unicellular (liverworts, hornworts) or multicellular (mosses) filaments that penetrate only the top few millimeters of the substratum. Despite these differences, the rhizoids and ventral tissues of liverworts and hornworts are colonized by mycorrhizal fungi in a similar manner as the roots hairs and root tissues of vascular plants^4^, reviewed in^12,13^. In addition to mycorrhizal fungi, pathogenic fungi infecting all three lineages bryophytes have been described in some detail reviewed in^2^. Less is known about the broader fungal microbiomes of bryophytes, however, a recent survey of fungal inhabitants of the liverwort *Marchantia polymorpha* in a variety of habitats revealed a diversity of fungi, some of which are pathogenic and others of which could promote growth of the liverwort host^14^.

In contrast to fungal components of bryophyte microbiomes, less is known about the bacterial communities inhabiting bryophytes. One exception is the well-characterized symbiotic colonization of slime cavities of hornwort thalli and ventral structures of the liverworts *Blasia* and *Cavicularia* by nitrogen-fixing cyanobacterial *Nostoc* colonies^15–17^. Recently, a few broad surveys of several bryophyte species has begun to provide insight into their broader bacterial microbiomes and have hinted at a diverse microbiome including some potential nitogen-fixing bacteria^5,18–21^.

Among liverworts, species within the genus *Marchantia* have become model genetic systems to investigate fundamental questions in evolutionary biology. Liverwort with a similar morphology have existed since the Permian^22^ possibly allowing ancient microbial associations to persist. Species of the liverwort genus *Marchantia* are cosmopolitan, inhabiting most of the terrestrial planet and can colonizing a large number of habitats including stream banks, rocks surfaces, logs, and also growing as epiphytes on trees^23^. *M*. *polymorpha* has attracted the attention of naturalists and scientists as a model system since the 18th century, with *Marchantia* pivotal to the study of sexual chromosomes in plants, sex determination, the cellular nature of organisms and to the study of the establishment of dorsoventral body plans and polarity^24^. The *M*. *polymorpha* subsp. *ruderalis* genome was the first liverwort genome sequenced^24–26^.

While *Marchantia polymorpha* subsp. *ruderalis* has not been observed to establish mycorrhizal fungal interactions, they have been observed in *M*. *polymorpha* subsp. *montivagans* and other *Marchantia* species, such as *M*. *paleacea* and *M*. *foliacea*^13,27–29^. Recently, fungal endophytes from wild populations of *M*. *polymorpha* were isolated and found their interactions with the host plant under *in vitro* conditions could be pathogenic, commensal or beneficial^14^. Again, less is known about the bacterial inhabitants of *Marchantia*. A handful of reports on culturable bacteria isolated from *Marchantia* thalli identified *Methylobacterium* as a likely commensal^30,31^. A study of the bacterial microbiome of *M*. *inflexa* in three different habitats (streamside, roadside, and greenhouse) found habitat-specific microbiome diversity, with the wild habitats harboring more similar microbiomes than that found under greenhouse conditions^21^. Interestingly, there are reports of *M*. *polymorpha* extracts able to inhibit bacterial growth^32^, suggesting an active microbial selection of its microbial guests.

**Figure 1:**
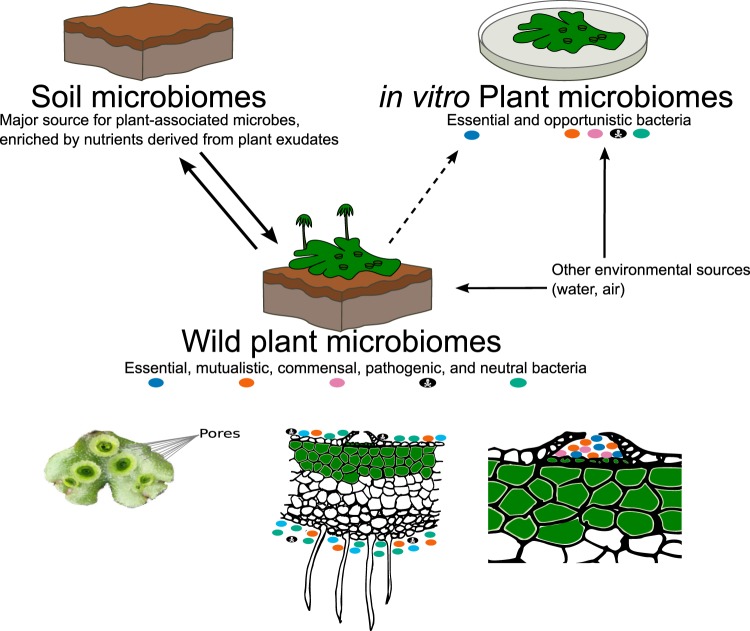
*Marchantia* microbiomes are the product of soil microbiomes and other environmental sources. Most bacteria in a plant microbiome are introduced to their soil as minor contributors from other environmental sources, such as rain and wind propagated microbes. Essential bacteria should be present in microbiomes from *Marchantia* grown in both the wild and *in vitro* conditions. By comparing *M*. *paleacea* and *M*. *polymorpha* under wild and *in vitro* conditions, we aimed to identify key plant-associated bacteria.

In this work we attempt to understand the richness and diversity of two *Marchantia* species microbial inhabitants in plants from the wild and plants established under *in vitro* conditions^33^ (Fig. 1). One species, *M*. *polymorpha* subsp. *ruderalis* is a rapid colonizer of disturbed habitats^34^ that does not form mycorrhizal associations. The second species, *M*. *paleacea*, is an ecological ‘stayer’ forming stable colonies with mycorrhizal associations. The plant’s microbiome, specifically the root-associated microbiome has been shown to have dramatic effects on plants establishment, survival, and access to nutrients^11^. Given their anatomical structure, profiling microbiomes from *Marchantia*’s thalli (also containing single cell rhizoids) would be the equivalent of microbiome profiles obtained from both root and phyllosphere in vascular plants.

## Results and Discussion

### Marchantia microbiome richness and diversity

A total of 164,385 Operational Taxonomic Units (OTUs; 97% identity 16S rRNA gene identity) were assigned to the whole dataset encompassing wild populations of *M*. *polymorpha* and *M*. *paleacea*, their source soils, and *in vitro* grown plants for both *Marchantia* species. After removal of chimeras, singletons, mitochondrial, and chloroplasts sequences, the whole dataset was reduced to 36,299 high-quality representatives OTUs that was the dataset used for this study. *Marchantia paleacea* under wild conditions recruited more OTUs (Mpala = 10,070) than wild *M*. *polymorpha* (Mpoly = 6,188) (see Table 1). Both soils of wild *M*. *paleacea* and *M*. *polymorpha* show similarly observed OTU numbers (Mpala = 8,367; Mpoly = 8,313). The *in vitro* grown *Marchantia* species showed a pattern opposite from their wild relatives, where *M*. *polymorpha* (1,162) recruited 2.89 more OTUs than *M*. *paleacea* (402). The nonparametric Chao1 index^35–37^ estimated an expected number of OTUs based on the number of low abundance OTUs. We used the Chao1 as a reference to determine the gap between our observed OTUs and a theoretical maximum. We observed fair sequencing coverage when comparing the observed OTUs against Chao1 Index with up to 89% of the wild *Marchantia* OTUs, up to 90% of soil OTUs coverage (*M*. *polymorpha*’s soil), and up to 99% of the *in vitro* grown *Marchantia* OTUs. Shannon’s index indicated that *Marchantia* wild species were diverse (Mpala = 8.29; Mpoly = 7.32) along with their source soils (~8.3), but the *in vitro* plants microbiomes had a substantial reduction in diversity (Mpoly = 3.69; Mpala = 1.35). We also observed diversity reduction by means of Simpson’s diversity index that assesses the probability that two sampled individuals will belong to different OTUs. We found the Simpson’s diversity index to be high for the wild *Marchantia* species (D > 0.99) but lower for *M*. *polymorpha* (D = 0.903) and *M*. *paleacea* (D = 0.576) under *in vitro* conditions. *Marchantia* species lack roots, but have single elongated cells called rhizoids. The complete *Marchantia* individual is only a few cell layers thick. Various models of root diversity were proposed. One such model was the two-step selection of rhizosphere microbiota (Bulgarelli *et al*.^11^) where the diversity decreased from soil to rhizosphere to endosphere. However, the model failed to account for every plant. Multiple accounts in the literature reported the rhizosphere was more diverse than its surrounding soil, such as rhizospheres of black pepper^38^ and rice^9^. Global meta-analysis also showed that plant rhizosphere diversity had a larger median than its soil or sediments^39^. One possible explanation for this diversity was that plant exudates influenced nutrient availability.

**Table 1:** *Marchantia* OTU richness and alpha diversity indices.

	Sequenced reads (after QC)	Raw OTUs (97% identity 16S rRNA gene)	OTUs (without singletons)	Chao1 (±SE)	Shannon (H)	Simpson (D)
Wild *M*. *paleacea*	175,252	17,613	10,070	11420.56 ± 87.79	8.29	0.998
Wild *M*. *polymorpha*	145,321	9,461	6,188	7048.66 ± 74.27	7.32	0.996
Soil *M*. *paleacea*	143,690	15,215	8,367	9696.03 ± 92.02	8.27	0.999
Soil *M*. *polymorpha*	144,918	15,051	8,313	9593.76 ± 90.75	8.26	0.999
*in vitro M*. *paleacea*	197,540	585	402	422.00 ± 10.56	1.35	0.576
*in vitro M*. *polymorpha*	167,224	1,882	1162	1229.50 ± 24.55	3.69	0.903

Number of assembled sequences with a read median size of 460 bp. We observed a total of 36,299 OTUs. The most diverse samples were the wild *Marchantia* and their soils. An expected, diversity dramatically decreased for the *in vitro Marchantia* species. Diversity indexes were calculated without singletons.

**Figure 2:**
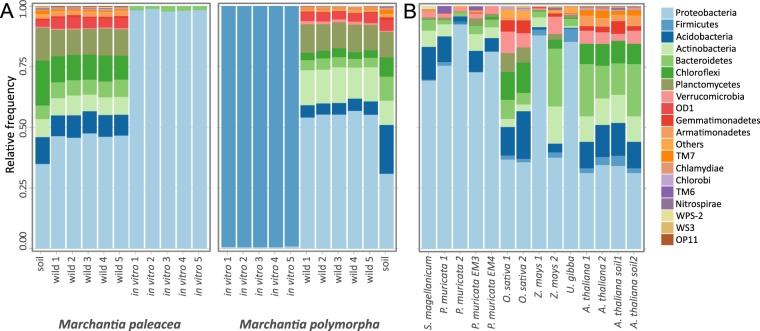
Phyla diversity in *Marchantia* microbiomes and a concise comparison of plant-related microbiomes. **(A)** *M*. *paleacea* and *M*. *polymorpha* microbiomes. *Proteobacteria* was the most abundant phylum for both wild *M*. *paleacea* and *M*. *polymorpha*, but *Firmicutes* dominated the microbiome diversity of *in vitro M*. *polymorpha*. **(B)** *Proteobacteria* was the most abundant phylum in the plant microbiomes analyzed but each microbiome had different frequencies, spanning from 30% up to 90%. Plant microbiomes were ordered according to their phylogenetic distances. *Sphagnum* moss^13^ served a representative for bryophytes (along with *Marchantia* species); *Pinus* represented gymnosperms^14^; rice^16^ and maize^15^ represented monocots; and the carnivorous plant *Utricularia gibba*^68^ and *Arabidopsis*^11^ represented dicots (see Methods).

We conducted taxonomic assignments of the OTUs, showed phyla affiliations for each sample, and transformed the data sets to a relative frequency (Fig. 2). The two wild *Marchantia* hosted larger *Proteobacteria* OTUs $(\bar{x}=50.85 \% )\,$than their source soils$\,(\bar{x}=32.81 \% )$. The second most abundant phylum hosted by *M*. *paleacea* was *Planctomycetes* $(\bar{x}=10.78 \% )\,$while *M*. *polymorpha* hosted *Actinobacteria* $(\bar{x}=13.9 \% )$. Despite having similar abundance patterns of the main phyla, the two *Marchantia* species could be distinguished based on their wild and *in vitro* microbiome profiles. The most abundant phylum hosted by the *Marchantia* species was *Proteobacteria* $(\bar{x}=50.85 \% )\,$for all samples, except for *in vitro M*. *polymorpha* plants that hosted more *Firmicutes* $(\bar{x}=99 \% )$. In both wild *Marchantia* species, the amount of *Proteobacteria* $({\bar{x}}_{march}=99 \% ;\,{\bar{x}}_{soil}=32.8 \% )$ and *Verrucomicrobia* $({\bar{x}}_{march}=7.92 \% ;\,{\bar{x}}_{soil}=5.7 \% )$ was higher in plant microbiomes than in their soils. On the other hand, *Acidobacteria*, *Chloroflexi*, *Planctomycetes*, *Bacteroidetes*, *TM7*, *Gemmatimonadetes*, *Nitrospirae*, *Verrucomicrobia*, *Chlorobi*, and *Firmicutes* phyla had lower proportions in the wild plants relative to source soils (Fig. 2A). While *Firmicutes* was detected in all samples and better represented in the *in vitro M*. *paleacea* microbiome $(\bar{x}=99 \% )$ elsewhere for wild plants $(\bar{x}=0.07 \% )$ nor soils $(\bar{x}=0.13 \% )$. As expected, the *in vitro Marchantia* species hosted lower overall diversity than their wild relatives. In the case of *M*. *paleacea*, diversity was largely dominated by *Proteobacteria* $(\bar{x}=98.25 \% )$ and small amounts of *Bacteroidetes* $(\bar{x}=1.6 \% )$. For *in vitro M*. *polymorpha*, *Firmicutes* $(\bar{x}=99.31 \% )$ was the main phyla in addition to a small fraction $(\bar{x}=0.66 \% )$ of *Proteobacteria* (Fig. 2A). A detailed breakdown of the diversity within phyla is presented in the Supplementary Figures (Supplementary Figs S1–S5).

To better understand the relevance of a *Marchantia* microbiome composition relative to microbiomes from other non-vascular and vascular plants (Fig. 2B; Table 2), we compared *Marchantia* bacterial communities with microbiomes from the bryophyte moss *Sphagnum magellanicum*, the gymnosperm *Pinus muricata* (containing two datasets, one dataset from roots containing arbuscular mycorrhiza and another dataset from mycorrhizae-free roots), rice, maize, *Arabidopsis thaliana*, and the bladders from *Utricularia gibba* (see Methods). Taking into account that the gametophytic thalli of both *Marchantia* species grew as laminar sheets attached to the soil by rhizoids, we decided to focus our comparative analysis on microbiomes from rhizospheres of representative species. By comparing the most abundant phyla across all datasets (Fig. 2B), we observed common features for all of the plant microbiomes, such as a high relative abundance of *Proteobacteria*, *Acidobacteria*, *Firmicutes*, *Actinobacteria*, and *Bacteroidetes* for most of the plants analyzed. Some samples showed a strong bias towards *Proteobacteria* (>0.50), including *M*. *polymorpha* (wild), *Pinus muricata*, *S*. *magellanicum*, *Utricularia gibba*, and a subgroup from the maize microbiome samples. Several non-cultivable bacteria phyla appeared to be widely distributed in plants microbiomes albeit in lower abundances, such as OD1, TM7, WPS-2, WS3, and OP11. Detailed OTU tables and taxonomic assignments are available as supplementary information (Supplementary Tables S1–S5).

**Table 2:** Alpha diversity indices in the comparison of microbiomes.

	Shannon	Simpson	Reference
*M*. *paleacea* wild	8.29	0.998	This work
*M*. *polymorpha* wild	7.32	0.996	This work
*M*. *paleacea* iv	1.35	0.576	This work
*M*. *polymorpha* iv	3.69	0.903	This work
*S*. *magellanicum*	8.62	0.999	(Bragina *et al*.^73^)
*Pinus muricata*	4.97	0.979	(Nguyen & Bruns^74^)
*P*. *muricata*, no mycorrhiza	5.12	0.985	(Nguyen & Bruns^74^)
*O*. *sativa*	9.68	0.999	(Edwards *et al*.^9^)
*Z*. *mays*	5.97	0.986	(Peiffer *et al*.^10^)
*A*.*thaliana*	7.19	0.998	(Lundberg *et al*.^7^)
*U*. *gibba*	8.49	0.999	(Alcaraz, *et al*.^75^)
*M*. *paleacea* soil	8.27	0.999	This work
*M*. *polymorpha* soil	8.26	0.999	This work
*A*. *thaliana* soil	7.52	0.999	(Lundberg *et al*.^7^)

*M*. *paleacea* displays more diversity than *M*. *polymorpha* in wild conditions, but the reverse is true for *in vitro* conditions. There is a slight decrease in diversity in *P*. *muricata* without endo-mycorrhizae. Diversity was estimated via individual study clustering. Sequences were publicly available, as stated in their publications.

**Figure 3:**
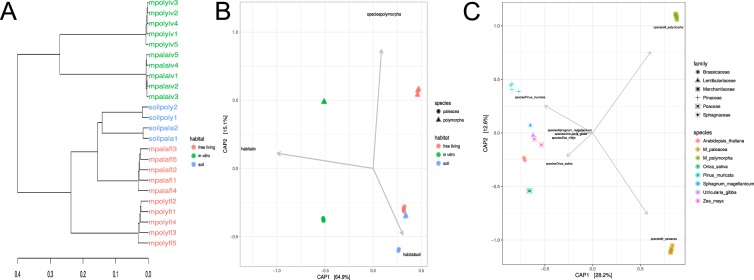
Comparison of plant microbiomes. **(A)** Weighted Unifrac distances for the *Marchantia* microbiomes were tested in this work. Diversity reduction in wild *M*. *polymorpha* (mpolyfl) showed a larger distance to its soil source when compared to *M*. *paleacea* (mpalafl). **(B)** Constrained analysis of principal coordinates (CAP) using weighted Unifrac distances with 9,999 permutational multivariate ANOVA showed significant (p > 1e-04) differences between *in vitro*, soil, and *M*. *polymorpha* microbial communities. **(C)** CAP of comparative literature plant-associated microbiomes using Bray-Curtis dissimilarities showed significant differences (p > 0.001) between microbial communities of different host plant species. Larger differences were found in *Pinus*, rice, and *Marchantia* species of this work.

Using Shannon’s index, we sorted the microbiomes under comparison based on their diversity (Table 2). Under wild conditions, *M*. *paleacea* displayed more diversity than *M*. *polymorpha*, but this diversity was reversed under *in vitro* conditions. All of the microbiomes under comparison showed large dominance values (Simpson >0.9) except for *in vitro M*. *polymorpha*. This discrepancy suggested that the microbial guests of *M*. *polymorpha* were not as structured as the other plants. In addition, random colonization by opportunistic bacteria might have occurred rather than a guest-host recognition and selection process (Table 2). The lack of microbiome diversity for *M*. *polymorpha* was supported by beta diversity analyses using both weighted and unweighted Unifrac distances and visualized with constrained analysis of principal coordinates (CAP). We found significant differences (p = 1e-04) between the *M*. *polymorpha* microbiome compositions under *in* vitro and wild conditions (Fig. 3A). Weighted Unifrac distances showed that lower diversity of *M*. *polymorpha* translated into a larger distance from its soil source than the wild plants of *M*. *paleacea*. Meanwhile, the *in vitro* microbiomes featured a distant external group compared to their wild relatives. Each species group formed a single cluster that supported a selective microbiome process for each species (Fig. 3B). The comparative analyses against other microbiomes was only possible through CAP using Bray-Curtis dissimilarities. We were unable to perform Unifrac, because the 22 samples were obtained by different methods and were not alignable (see Methods). The analyses showed significant differences (p > 0.001) between plant taxonomy (family and species) such that the microbiome composition variance split *Marchantia* from the rest of species. These analyses accounted for 28.2% of the variation (Fig. 3C). Additional beta diversity comparisons using 43 different beta diversity distances and three other types of method comparisons are available as Supplementary Fig. S8.

### Marchantia soil and *in vitro* microbiome interactions

**Figure 4:**
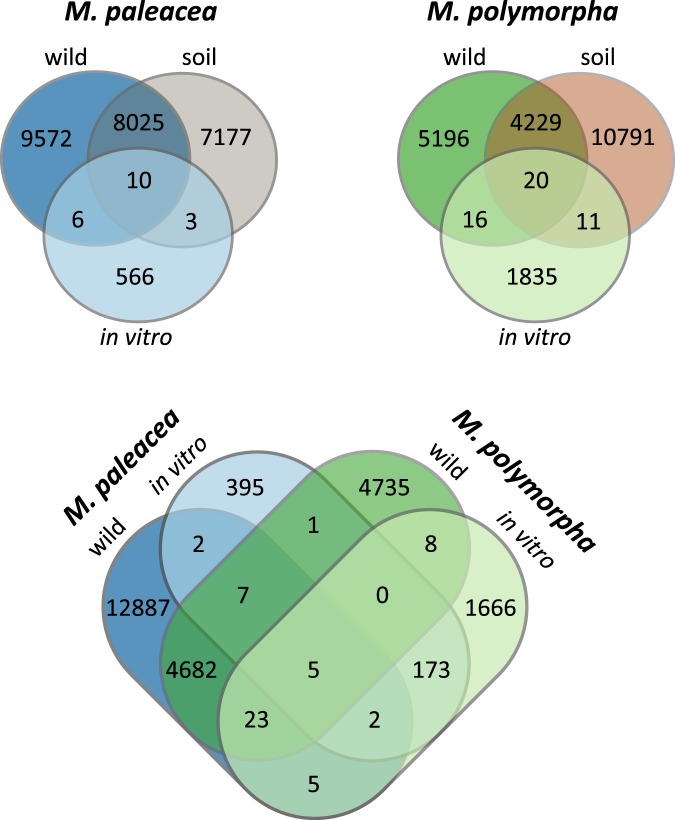
Venn diagrams of microbiomes from *M*. *paleacea*, *M*. *polymorpha* and their soils. Above, OTUs are compared from *in vitro*, wild and soil microbiomes of each *Marchantia* species. Below, wild and soil microbiomes are compared from the two *Marchantia* species.

We conducted both qualitative and quantitative analyses to determine the shared, unique, and significantly different features for *Marchantia* species and their soils. First, the qualitative approach of comparing datasets showed that 8,035 OTUs were shared between *M*. *paleacea* and its soil, 7,180 OTUs were unique to the soil, and 9,578 OTUs were unique to the wild plant (Fig. 4). Wild *M*. *polymorpha* displayed less diversity (9,461 OTUs) than wild *M*. *paleacea* (17,613 OTUs) while both plants shared 4,717 OTUs. We found 180 OTUs in both *in vitro* grown plant species. We observed 16 OTUs shared between wild and *in vitro M*. *paleacea* possibly due to having similar microbial survivors of various *in vitro* selection constraints. A group of 569 OTUs were exclusive to *in vitro* plants. In future experiments, we plan to test whether this exclusive set is the result of opportunistic or host selected bacteria. *M*. *polymorpha* showed a similar case with 4,249 OTUs shared between the wild plant and soil and 5,212 exclusive OTUs in the wild plant. Only 36 OTUs were shared between wild and *in vitro M*. *polymorpha* while 1,846 OTUs were unique to the *in vitro* plant (Fig. 4). Relative abundances of the OTUs and their identities in a Venn diagram are available as Supplementary Table S6. We were surprised by the large number of particular plant OTUs. Some of these unique bacteria could come directly from air and water. However, air and water microbiomes were found to be less diverse than soil microbial communities. Rainwater and river microbial diversity (Chao1 index) was under 1,000 OTUs, while diversity from air was below 600 OTUs^40,41^. The results were congruent with the rarefaction analysis (Supplementary Fig. S7). This diversity of microbial sources suggested that plant microbiome composition was influenced by plant-environmental interactions with some bacteria transferred from plant to plant and others dispersed by soil, air, or water.

On the other hand, the union set between the two *Marchantia* species and their soils constituted only five OTUs. The identity of these five OTUs which we called *Marchantia* microbiome members were assigned up to the genus level as *Methylobacterium* (*Alphaproteobacteria*; (relative reads abundance) ra = 0.00066), *Rhizobium* (*Alphaproteobacteria;* ra = 0.0029), *Paenibacillus chondroitinus (Firmicutes*; ra = 0.0222), *Citrobacter* (*Gammaproteobacteria*; ra = 0.00013) and *Methylobacterium organophilum* (ra = 0.00036). The relevance of the five taxa relied on a core community assumption. The core microbial community came from the soil, and the shared microbiomes was modulated by the plant hosts. This qualitative approach allowed us to determine which microbes had an intimate relation with their hosts. Next, we summarized information about the abovementioned taxa showing that their genera featured plant-associated lifestyles.

*Paenibacillus chondroitinus* (*Firmicutes*; ra = 0.0222), previously classified as *Bacillus chondroitinus*, was named for its ability to degrade complex carbohydrates, such as chondroitin and alginates (Nakamura, 1987). It was discovered to be a core member of the *Marchantia* microbiome (<0.95 confidence, as a species). Chondroitin and alginates play a role in diverse desiccation tolerance mechanisms in bacteria. For example, common soil bacteria, such as *Azotobacter vinelandii*, have the ability to produce alginates that are crucial for the development of desiccation resistant cysts. Bacteria produced alginates can also be used as virulence factors, such as *Pseudomonas aeruginosa* which happens to infect plants, insects, nematodes, and mammals^42,43^. Desiccation was one of the major challenges for plants during the transition from an aquatic to a terrestrial environment. *M*. *polymorpha* has been used as a model to study desiccation tolerance^44,45^. In the case of *Marchantia* accompanying bacteria, desiccation resistance strategies—like the one used by *Azotobacter* (alginate mediated) or the formation of spores like those in *Bacillus—*highlight the importance of molecular mechanisms for drought resistance in plants and their microbes. At the end of the dry season, drought resistance related compounds should be metabolized as is the case of chondroitin being degraded by *P*. *chondroitinus*. Additionally, alginate and depolymerized alginates have been shown to promote plant root growth by the induction of auxin biosynthesis genes in the plant^46,47^.

*Methylobacterium* species are plant-associated bacteria in both phyllosphere and roots, forming nodules in some species, helping with nitrogen fixation, and producing cytokinin *trans*-zeatin. They are known as pink-fermented facultative methylotrophic bacteria (PPFM) and are able to grow on single carbon sources (methanol and methylamine) as well as other C_2_, C_3_, and C_4_ sources^48–50^. The residence of *Methylobacterium* OTUs within *Marchantia* species was supported in other research where the same *Methylobacterium* species were present in year-long transgenerational experiments involving *Arabidopsis*^49^. *Methylobacterium* was found to occur as a core microbiome component for the dwarf shrub *Andromeda polifolia* found in alpine bog vegetation that was dominated by *Sphagnum* species^51^. Additionally, the pink pigmented bacterial colonies of *Curtobacterium* and a *Rhizobiaceae* were observed in our *in vitro* grown *M*. *polymorpha* and *M*. *paleacea*. A new *Methylobacterium* species, *M*. *marchantiae* species nov., was isolated from a thallus of *M*. *polymorpha*^31^. *Methylobacterium* is a phytosymbiont for liverworts and mosses. Some believe methanol is consumed in liverworts as the by-product of the plant’s cell wall metabolism and then emitted through *Marchantia*’s upper epidermis stomata-like pores^31,52^. *Methylobacterium* probably accumulates within the plant’s air chambers. *Methylotenera* is another member of methylotrophic bacteria (C_1_ as source) prevalent in *M*. *paleacea*. *Methylotenera mobilis* grew in the presence of methylamine as a source of carbon, nitrogen, and energy under laboratory conditions, but methylotrophy seemed to be facultative under natural conditions^53^. Our study confirmed the presence of two *Methylobacterium* OTUs (147,611 and 150,024 OTU ID) that were present in wild and *in vitro M*. *paleacea* and *M*. *polymorpha* plants as well as soil. *Marchantia* stomata-like pores (air chambers), visible to the naked eye, remained open most of the time as they were not finely regulated. Air chambers allowed gas exchange to occur while controlling the contact of internal moisture with photosynthetic cells^54^. Methanol was emitted as a volatile organic compound (VOC) from leaves and through plant stomata^55^, and the release of plant methanol was correlated with leaf growth^52^. The air chambers of *Marchantia* proved to be an ideal habitat for various bacteria, possibly *Methylobacterium*, that made use of C_1_ sources, such as methanol.

**Figure 5:**
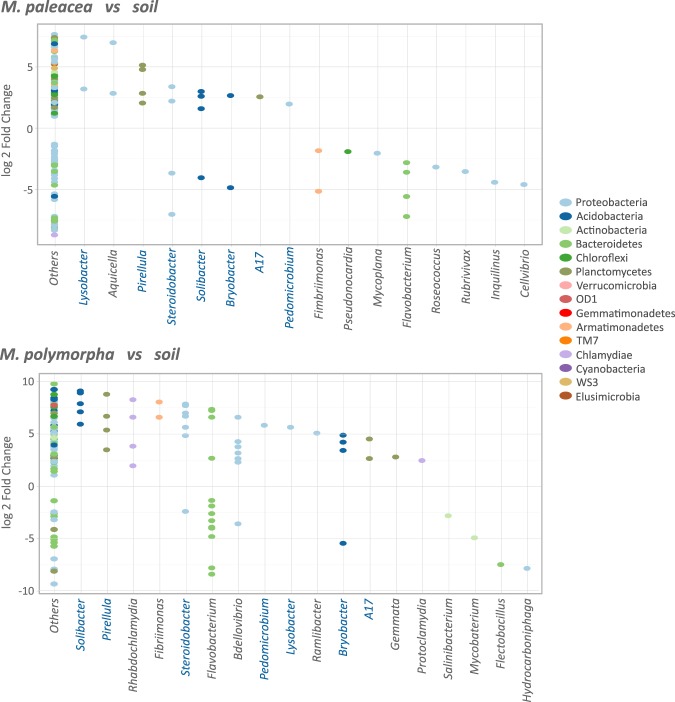
Relative abundance of the bacteria associated with *Marchantia*. The genera with significant (padj = 0.01) log2 fold changes are shown. “Other” corresponds to OTUs whose genera could not be assigned. Each dot represents an OTU. The OTUs prevalent in the *Marchantia* species are located on top of the plot while the OTUs prevalent in soil are located at the bottom. The majority of the OTUs were not assigned to a known genus, and are shown as “Others” in the plots. Shared genera between both *Marchantia* species are highlighted in blue.

We used a quantitative approach to detect interactions between significantly different OTUs among plants and soil bacteria by comparing the log2 fold change (log2fc) of the OTU abundance ratio for wild *Marchantia* species versus their soil (Fig. 5; Supplementary Table 6). We used DESeq2^56^ log2fc where fold changes less than one were negative values and fold changes greater than one were positive values, making it easy to plot the changes symmetrically in a single plot. Then we obtained an adjusted *p-*value (padj > 0.01) to correct for false positives (False Discovery Rate, FDR) using the Benjamini-Hochberg (BH) correction^57^. A total of 201 OTUs of *M*. *paleacea* and 280 OTUs of *M*. *polymorpha* displayed a significant (*padj* < = 0.01) change of abundances compared their soils (Fig. 5). The majority of OTUs were *Proteobacteria* from both *M*. *paleacea* (44%) and *M*. *polymorpha* (35%), followed by *Bacteroidetes*, *Chloroflexi*, *Acidobacteria* and *Planctomycetes* that contained around 9% (for each phylum) of the significant OTUs from both plants. The “Others” category in Fig. 5 corresponded to the vast majority of OTUs that were not phylotyped up to genus level (171/201 *M*. *paleacea*; 222/280 *M*. *polymorpha*) but to at least the phylum level and pertained to bacteria with significant log fold changes due to their source. Seven highly represented phylotyped genera along with both wild *Marchantia* species could play a major role in plant growth and development, including *Lysobacter*, *Pirellula*, *Steroidobacter*, *Solibacter*, *Bryobacter*, *A17*, and *Pedomicrobium*. Several OTUs were in surprisingly lower numbers in the plants which indicated a negative interaction. Some genera, such as *Flavobacterium* or *Steroidobacter*, showed OTU-specific enrichments and depletions that suggested species or strain specific relationships between plant and bacteria.

Plants contained derived photosynthates as carbon sources, thus their associated microbes were expected to feature metabolic machinery that were able to utilize such sources. These specialized carbon consumers, such as *Bryobacter* (*Acidobacteria*), were prevalent in wild *Marchantia*. These bacteria have been described as a typical component of *Sphagnum*-dominated bogs. They are non-motile and grow well on the residual products of *Sphagnum* decay, such as complex heteropolysaccharides, glucuronic acids, and galacturonic acids^58^. Another bacterial genus dominating *M*. *paleacea* includes *Steroidobacter* (γ*-Proteobacteria*) which was isolated from soils and plant roots^59,60^. Some strains, such as *Steroidobacter agariperforans*, degrade complex polysaccharides derived from rhizospheres and can even degrade agar. *S*. *agariperforans* was isolated as a commensal strain to a *Rhizobiales* bacterium^59^. *S*. *denitrificans* was discovered to denitrify under anoxic conditions using nitrate as an electron acceptor and to degrade steroidal hormones^60^. Denitrification could be parallel to nitrogen fixation. Denitrification in roots and nodules could reduce cytotoxic damages produced by nitrates. These nitrates could inhibit nitrogen fixation and be a final electron acceptor for rhizobia when the plant is under environmental stress, such as floods^61^. Recently, *Steroidobacter* was reported to be part of the core rhizosphere microbiome of the gymnosperm *Welwitschia mirabilis*, a 110 million-year-old living fossil^62^.

Predators, such as bacteria, have also been found to be attracted to a nutrient rich environment containing plant derived photosynthates. *Bdellovibrio bacteriovorus*, the most studied species of the *Bdellovibrio* genus, is a sophisticated Gram-negative bacterial predator^63^. *Bdellovibrio* species were isolated from a wide range of environments, including plant rhizospheres. The concentration of *B*. *bacteriovorus* in rhizospheres can reach levels higher than one order of magnitude relative to the circumventing soil. *Bdellovibrio* likely preys on bacteria attracted to the C-rich plant exudates^64^. The noticeably high concentration of *Bdellovibrio* on *Marchantia* is interesting, because it might prey on other Gram-negative inhabitants that are potential pathogens, such as *Agrobacterium*. *Agrobacterium* was reported to be prevalent in *M*. *polymorpha*.

A suggested explanation for the greater bacterial diversity observed in wild *M*. *paleacea* compared to *M*. *polymorpha* was consistent with previous work in which the mycorrhiza formation capabilities with glomeromycotan fungi (GA) were analyzed for a set of species of liverworts and were depleted in *M*. *polymorpha*^29^. Ligrone and collaborators (2007) found evidence which suggested an ancient symbiotic origin for GA and liverworts as well as several independent losses for the mycorrhiza formation across time. Molecular explanations of GA absences in *M*. *polymorpha* could be due to mutations or gene silencing in the *M*. *polymorpha* mycorrhiza plant symbiotic genes DMI1, DMI3 and IPD3^65^. We observed a larger diversity—diversity indices and OTU richness—in the *M*. *paleacea* wild microbiome than its soil substrate. This diversity had nearly twice as many observed OTUs as *M*. *polymorpha*. The lowest microbe diversity of *M*. *polymorpha* could be due to their low symbiotic capabilities for this plant under wild conditions. Another possibility for lower microbiome diversity is consequence of both *Marchantia* species ecological strategies, where *M*. *polymorpha* is a primary colonizer of disturbed habitats, whereas *M*. *paleacea* is resilient in its environment^26^.

Potential reasons for the different microbial diversity observed in the *in vitro* growing plants include: 1) Microbiological condition were cleaner for *M*. *paleacea*; 2) The microbiome of *M*. *polymorpha* was composed of opportunistic and maybe transient bacteria, while *M*. *paleacea* was actively recruiting its microbial inhabitants; 3) *M*. *paleacea* actively selected and recruited microbes which might be a trait that *M*. *polymorpha* lost; and 4) Different developmental times between *M*. *paleacea* and *M*. *polymorpha*. Indeed, *M*. *paleacea* took about 28 days longer to develop gemmae cups and gemmae than *M*. *polymorpha* under our laboratory conditions. We would like to investigate these possibilities in future research. We think that *in vitro* conditions were providing the right selective conditions for recruiting plant-associated microbiota through a frequency dependent selection mechanism driven by the plants. Isolated bacteria from *in vitro Marchantia* species were reported^30,31^, even though the plant culture media were not optimal for bacteria growth. All carbon sources for heterotrophic bacteria should be derived from plant exudates. Some of the *in vitro* bacteria were probably acquired by handling in the laboratory, aerosols, dust, and several contamination sources but some of the identified microbes are closer to plant-associated bacteria that are being transferred in growth conditions.

## Conclusions

For both *Marchantia* species, we were able to identify highly abundant OTUs from their wild sources. We observed that OTUs enriched in *Marchantia* showed evidence of plant-associated lifestyles supported by related cultured strains previously discussed. We identified genera of plant growth promoting bacteria (i.e. *Rhizobium* and *Methylobacterium)* and complex organic compound degrading bacteria (*i*.*e*. *Paenibacillus*, *Steroidobacter*, and *Lysobacter*) both reported to use plant-derived polymers and return plant hormones that provided pathogen protection to their hosts. We recorded the presence of bacterial predators, such as *Bdellovibrio*, that actively attacked and parasitized other *Proteobacteria*, suggesting that negative interactions occurred among *Marchantia* species inhabitants. The enrichment for methylotrophic bacteria was likely due to bacterial niche opportunity and specialization found in the *Marchantia* air chambers. The microbial toolkit of *M*. *paleacea* and *M*. *polymorpha* could facilitate understanding whether the microbial input affects plant development.

## Methods

### Sampling

A total of 30 *Marchantia polymorpha* and 30 *M*. *paleacea* specimens were collected in wild conditions near the locality of Oxtlapa, a municipality of Xico in the Mexican state of Veracruz (19°25′26″N, 97°3′31″W and 19°25′33″N, 97°3′31″W elev. 1,763 masl; collection date: 7/3/2013). Both plant species were growing in a rocky substrate with shallow soil (<1 cm). This location was chosen because both species were naturally occurring in sympatric conditions with less than 200 m between sampling sites for each species (see Supplementary Fig. S6). We collected specimens in the field with sterilized tweezers and carefully removed adhering soil particles. The cleaned specimens were placed into 50 ml sterile tubes that were frozen in place with liquid nitrogen. We collected approximately 50 ml of soil volume from each plant location to study its role as an inoculant for the *Marchantia* microbiomes. Landowners allowed us to sample within their terrain without the need of a special collection permit. Neither *Marchantia* species were found on the Red List of the International Union for Conservation of Nature (IUCN; http://www.iucnredlist.org/search). Gemmae from both *Marchantia* (female) species grown in the wild were disinfected and grown under *in vitro* conditions (following the detailed protocols described in Ishizaki *et al*. 2016) to generate thalli. We recovered gemmae from *in vitro* cultured thalli, and the resulting gemmae were grown and propagated *in vitro* for three subsequent cycles (gemmae-to-gemma). We processed the resulting thalli for DNA isolation.

### DNA extraction and sequencing library preparation

Five plant specimens of each *Marchantia* species were used for each treatment (wild and *in vitro*) for DNA extractions. The naturally occurring populations of *M*. *polymorpha* and *M*. *paleacea* were gently washed away from the surrounding particles with phosphate buffer. Then they were washed by vortex mixing with 1% Tween-20 and the resulting pellets were used to extract the metagenomic DNA. We used the same procedure on the *in vitro* specimens of the *Marchantia* species. These samples were removed from the agar plate with sterile tweezers and washed with 1% Tween-20 since there were no soil particles attached to them. We washed the plant pellets and soil samples to extract plant-derived metagenomic DNA following MoBio’s PowerSoil procedures (MoBio Laboratories, Solana Beach, CA). We observed the *Marchantia* microbiome as the microbes attached to plant thalli surfaces. Because of the small size of the plants (1 cm × 5 cm; and only a few cell layers thick), we recovered microbes from the rhizoids, epidermis, pores that were analogous to the rhizosphere, phylosphere, and endophytic microbiome of other plants We followed the phosphate buffer washing-vortexing protocol that was used in several plants microbiome studies^7^.

We used PCR primers from the V3-V4 (341 F, 805 R; 464 bp amplicon) region of the 16S rRNA gene (Klinsworth 2012) which was recommended by the MiSeq™ Illumina® protocol with 5′ overhangs for multiplex library preparation. We performed triplicate PCR reactions using a final volume of 30 μL using high fidelity *Pfx* platinum polymerase (Invitrogen, Carlsbad, CA) and mixed all the reactions per sample to a final volume of 90 μL. The PCR conditions were as follows: denaturation at 95 °C for 1 min; 5 cycles of denaturation at 94 °C for 30 s, annealing at 55 °C for 30 s, and extension at 68 °C for 30 s; 25 cycles of two-step cycling with denaturation at 94 °C for 5 s, and extension at 68 °C for 30 s. PCR products were column purified using High Pure PCR Product Purification Kit (Roche Diagnostics GmbH, Mannheim, Germany). The 16S rRNA amplicon libraries were constructed (barcodes and Illumina multiplex sequencing adapters added) and sequenced at the Unidad de Secuenciación Masiva at the National Autonomous University of Mexico’s Biotechnology Institute, using a Illumina® MiSeq™ run with a 2 × 300 bp configuration based on manufacturer’s directions.

### Sequence processing

We used a previously reported pipeline for 16S amplicon sequences^66^ (https://doi.org/10.6084/m9.figshare.3470555.v1). Briefly, we merged paired-end reads using PANDASEQ (Masella, Bartram, Truszkowski, Brown & Neufeld 2012) and used FASTX tools for quality control (http://hannonlab.cshl.edu/fastx_toolkit/). We trimmed all of the reads to the expected amplicon size (250 bp) and assembled them using the following parameters: a minimum probability threshold of 0.95 that accounts for the minimum probability cut-off for assembly; a minimum length of 250 bp, and a maximum length of 470 bp. Clustering and OTU picking was done using CD-HIT-EST with a 97% identity cutoff and a minimum of 97% for the read length. We tested multiple open and closed-reference OTU picking strategies and decided to use the CD-HIT program, because it fit our computer cluster configuration and the OTU range was enormous—from 6,806 to 896,439 OTUs—depending on the chosen software (QIIME options). We built the OTU table using *make_otu_table*.*py* of QIIME’s suite^67^ and picked the representative OTUs. We conducted taxonomy assignment with BLAST against the Greengenes database (13_8 release^68^). We identified chimeras and removed them with ChimeraSlayer^69^. Finally, we identified and removed mitochondrial and chloroplast sequences from the following analyses.

### Diversity metrics and statistical analysis

All of the diversity metrics were calculated with R (R Core Team 2014) and its phyloseq^70^, and vegan^71^ packages. We created plots using R′s phyloseq, ggplot2 (Wickham 2009) and RColorBrewer (www.ColorBrewer.org) libraries. With the alpha diversity metrics, we calculated the number of observed species, nonparametric Chao1 index^35–37^, Shannon’s index^72^, and Simpson’s diversity index (Simpson) for the *Marchantia* species and the comparative dataset. We generated Venn diagrams using the Draw Venn Diagram tool (http://bioinformatics.psb.ugent.be/webtools/Venn/). We calculated community distances with phyloseq. Several normalization procedures were conducted on the data, including performing relative frequency transformation to obtain bar plots that compared phyla abundances across samples/species. To search for significant differences between *Marchantia* samples, we applied a normalized logarithmic transformation (rlog) on the OTUs. We computed counts by fitting each OTU to an abundance baseline using a generalized linear model (GLM) and estimated logarithmic fold change (LFC) and dispersion for each OTU. We corrected for false positives (False Discovery Rate, FDR) using the Benjamini-Hochberg (BH) correction. We made these comparisons using the R′s DESeq2 package^56^. Detailed R procedures can be found in Supplementary Table S7.

### Compared microbiomes

For comparison purposes, we chose several available root microbiomes for plant species in order to get a general representation of land plants. From publicly available repositories of published data (see Table 2), we selected sequences from one *Sphagnum magellanicum* moss microbiome^73^ and four samples from a *Pinus muricata* rhizosphere microbiome study^74^. We selected root microbiome samples from individuals with and without arbuscular mycorrhizas, two samples from B73 maize root microbiomes^10^, two samples from rice’s roots (Edwards *et al*.^9^), one sample from bladder associated microbes of the carnivorous plant *Utricularia gibba*^75^; and two root associated microbiomes from *Arabidopsis thaliana*^7^. Sequences for comparative microbiomes were downloaded from the databases stated in their respective publications. All of the sequences groups were processed individually (see Sequence processing) for diversity estimations. We performed QIIME’s *pick_closed_otus*.*py (*Caporaso *et al*.^67^) using a similarity cutoff of 0.97 and utilized the gg_otus_13_8 database^68^. Although the comparison microbiome studies were conducted with different technologies, sequencing lengths, and 16S rRNA gene variable regions, we were able to compare the samples using a closed-reference full-length 16S rRNA sequence to cluster the sequences.

## Electronic supplementary material

Supplementary information

Suplementary dataset

## Data Availability

Data for the *Marchantia* microbiome was deposited in the GenBank/EMBL/DDBJ Bioproject database under the accession code PRJNA320287. Raw genomic sequence data were deposited in the Sequence Read Archive (SRA) GenBank/EMBL/DDBJ for the whole study under the accession code SRP078003 as well as the following individual accessions: SRR3746049, SRR3746050, SRR3746051, SRR3746052, SRR3746053 and SRR3746054. Raw OTU tables, taxonomic assignments for *Marchantia* and the comparative dataset are available as supplementary information (Supplementary Tables S1–S5).
