# Supplementary material for: Marchantia liverworts as a proxy to plants’ basal microbiomes
Source: Sci Rep. 2018 Aug 23;8:12712. doi: 10.1038/s41598-018-31168-0 (PMC6107579; doi:10.1038/s41598-018-31168-0)
Supplement: Supplementary file 1 — Supplementary information [file 41598_2018_31168_MOESM1_ESM.pdf]

Marchantia liverworts as a *proxy* to plants' basal microbiomes

Luis D. Alcaraz[1*], Mariana Peimbert[2], Hugo R. Barajas[1], Ana E. Dorantes-Acosta[3], John L. Bowman[4], and Mario A. Arteaga-Vázquez[3*]

[1]Departamento de Biología Celular. Facultad de Ciencias e Instituto de Ecología. Universidad Nacional Autónoma de México. Ciudad Universitaria, UNAM, 04510, Cd. Mx., México.

[2]Departamento de Ciencias Naturales. Universidad Autónoma Metropolitana, Unidad Cuajimalpa. Av. Vasco de Quiroga 4871, Col. Santa Fe Cuajimalpa, 05348, Cd. Mx., México.

[3]University of Veracruz, Institute for Biotechnology and Applied Ecology (INBIOTECA), Avenida de las Culturas Veracruzanas 101, Colonia Emiliano Zapata, 91090, Xalapa, Veracruz, México.

[4]School of Biological Sciences, Monash University, Melbourne, Victoria 3800, Australia.

*Correspondence authors: lalcaraz@ciencias.unam.mx, maarteaga@uv.mx

S1 Fig. *Acidobacteria* main families. Colors are repetitive, because they belong to the same family. Each horizontal bar represents an individual OTU. Bar height represents relative frequency of each OTU. OTUs are sorted from the most abundant at the bottom to the least abundant at the top of the histogram.

S2 Fig. *Actinobacteria* main families. Colors are repetitive, because they belong to the same family. Each horizontal bar represents an individual OTU. Bar height represents relative frequency of each OTU. OTUs are sorted from the most abundant at the bottom to the least abundant at the top of the histogram.

S3. Fig. *Bacteroidetes* main families. Colors are repetitive, because they belong to the same family. Each horizontal bar represents an individual OTU. Bar height represents relative frequency of each OTU. OTUs are sorted from the most abundant at the bottom to the least abundant at the top of the histogram.

S4. Fig. *Planctomycetes* main families. Colors are repetitive, because they belong to the same family. Each horizontal bar represents an individual OTU. Bar height represents relative frequency of each OTU. OTUs are sorted from the most abundant at the bottom to the least abundant at the top of the histogram.

S5. Fig. *Proteobacteria* main families. Colors are repetitive, because they belong to the same family. Each horizontal bar represents an individual OTU. Bar height represents relative frequency of each OTU. OTUs are sorted from the most abundant at the bottom to the least abundant at the top of the histogram.

S6. Fig. Field sampling images. The habitat for our wild specimens consisted of rocky surfaces with shallow soil (>1cm depth).

S7. Fig. Rarefaction curves for *Marchantia* OTUs.

S8. Fig. Beta diversity with multiple distances and comparison methods.

S1 Table. Unfiltered *Marchantia* microbiome OTU table. **Excel file

S2 Table. Unfiltered *Marchantia* OTU taxonomic assignments. **Excel file

S3 Table. *Marchantia* metadata file. **Excel file

S4 Table. Comparative microbiomes OTU table. **Excel file

S5 Table. Comparative microbiomes metadata file. **Excel file

S6 Table. Qualitative analyses of shared OTUs and relative abundances. Significant differential OTUs and relative frequencies. **Excel file

S7 Table. Venn diagram results. Shared OTUs.

S8 Table. R analysis protocols.

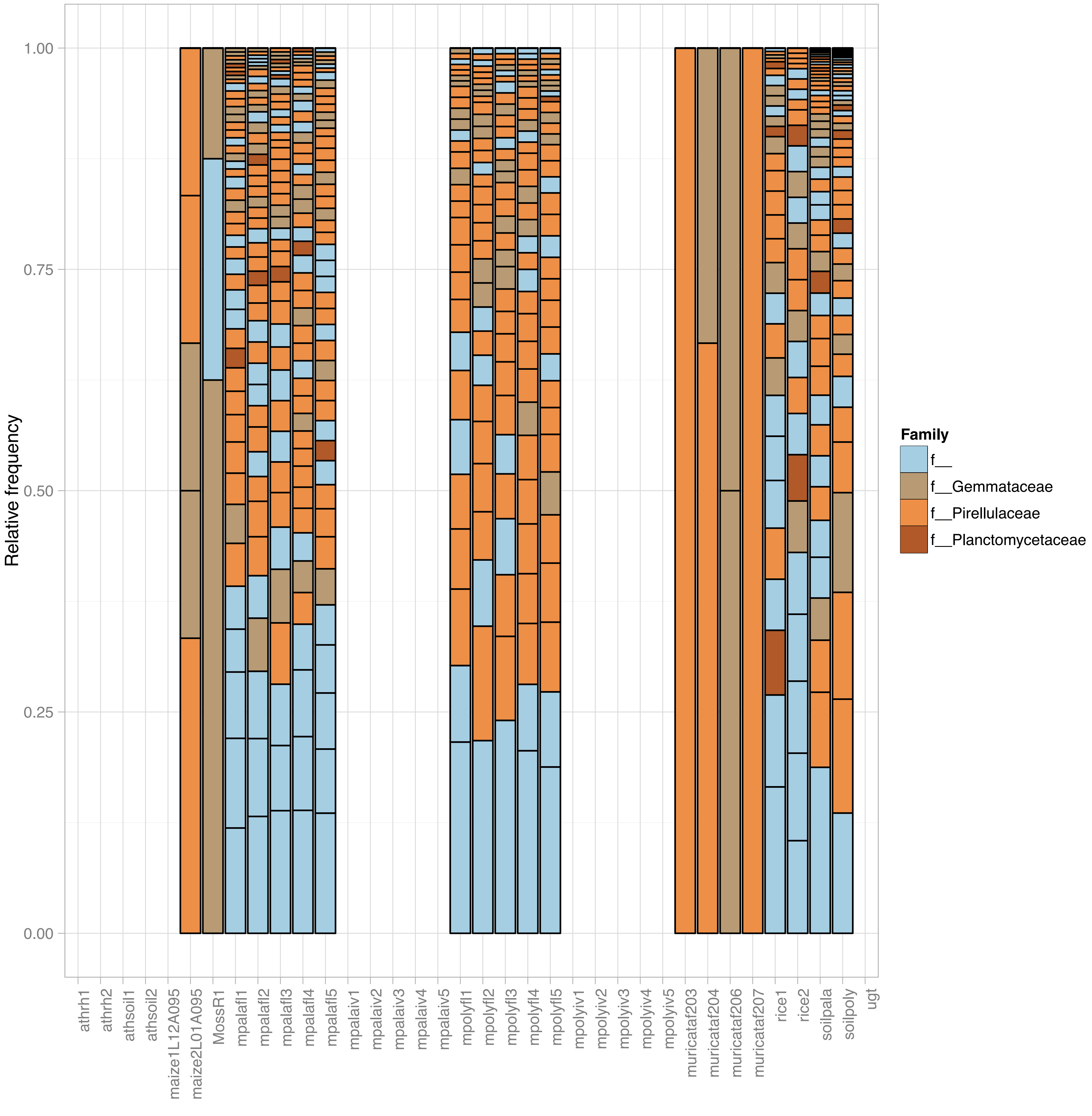

Relative frequency
1.00
0.75
0.50
0.25
0.00
athrh1
athrh2
athsoil1
athsoil2
maize1L12A095
maize2L01A095
MossR1
mpalafl1
mpalafl2
mpalafl3
mpalafl4
mpalafl5
mpalaiv1
mpalaiv2
mpalaiv3
mpalaiv4
mpalaiv5
mpolyfl1
mpolyfl2
mpolyfl3
mpolyfl4
mpolyfl5
mpolyiv1
mpolyiv2
mpolyiv3
mpolyiv4
mpolyiv5
muricataf203
muricataf204
muricataf206
muricataf207
rice1
rice2
soilpala
soilpoly
ugt
Family
f__
f__Gemmataceae
f__Pirellulaceae
f__Planctomycetaceae

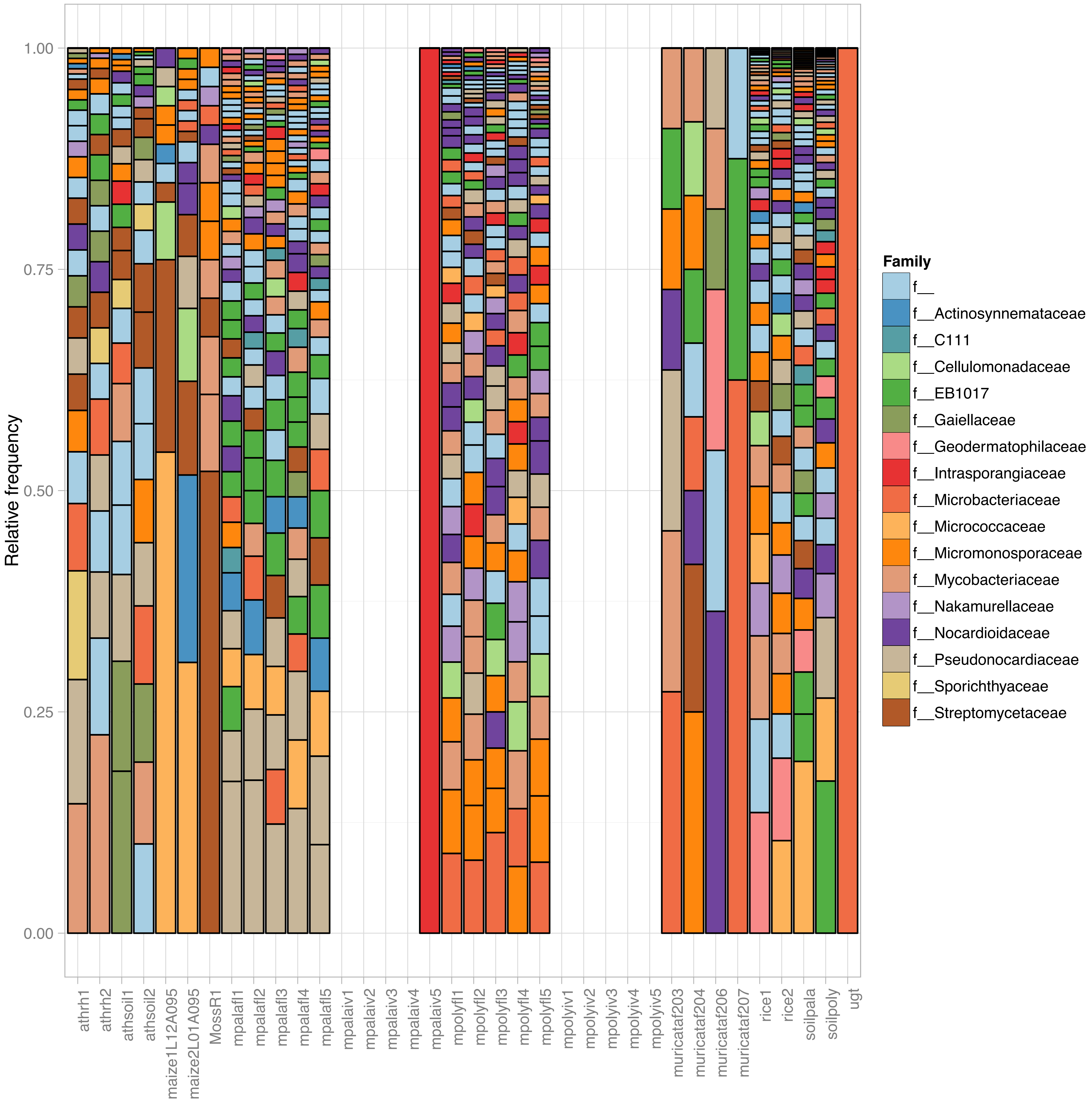

Relative frequency
1.00
0.75
0.50
0.25
0.00
athrh1
athrh2
athsoil1
athsoil2
maize1L12A095
maize2L01A095
MossR1
mpalafl1
mpalafl2
mpalafl3
mpalafl4
mpalafl5
mpalaiv1
mpalaiv2
mpalaiv3
mpalaiv4
mpalaiv5
mpolyfl1
mpolyfl2
mpolyfl3
mpolyfl4
mpolyfl5
mpolyiv1
mpolyiv2
mpolyiv3
mpolyiv4
mpolyiv5
muricataf203
muricataf204
muricataf206
muricataf207
rice1
rice2
soilpala
soilpoly
ugt
Family
f__
f__Actinosynnemataceae
f__C111
f__Cellulomonadaceae
f__EB1017
f__Gaiellaceae
f__Geodermatophilaceae
f__Intrasporangiaceae
f__Microbacteriaceae
f__Micrococcaceae
f__Micromonosporaceae
f__Mycobacteriaceae
f__Nakamurellaceae
f__Nocardioidaceae
f__Pseudonocardiaceae
f__Sporichthyaceae
f__Streptomycetaceae

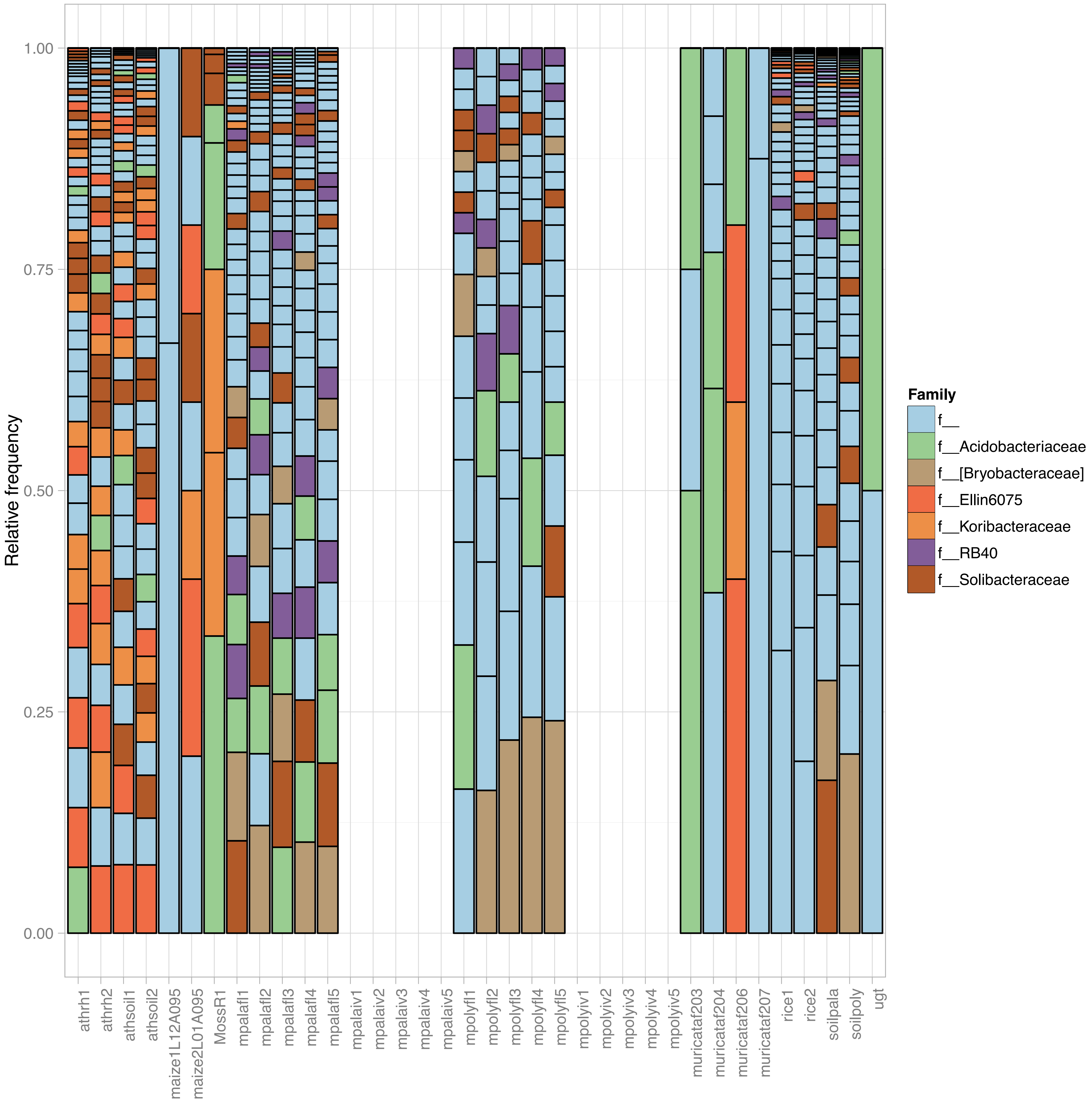

Relative frequency
1.00
0.75
0.50
0.25
0.00
athrh1
athrh2
athsoil1
athsoil2
maize1L12A095
maize2L01A095
MossR1
mpalafl1
mpalafl2
mpalafl3
mpalafl4
mpalafl5
mpalaiv1
mpalaiv2
mpalaiv3
mpalaiv4
mpalaiv5
mpolyfl1
mpolyfl2
mpolyfl3
mpolyfl4
mpolyfl5
mpolyiv1
mpolyiv2
mpolyiv3
mpolyiv4
mpolyiv5
muricataf203
muricataf204
muricataf206
muricataf207
rice1
rice2
soilpala
soilpoly
ugt
Family
f__
f__Acidobacteriaceae
f__[Bryobacteraceae]
f__Ellin6075
f__Koribacteraceae
f__RB40
f__Solibacteraceae

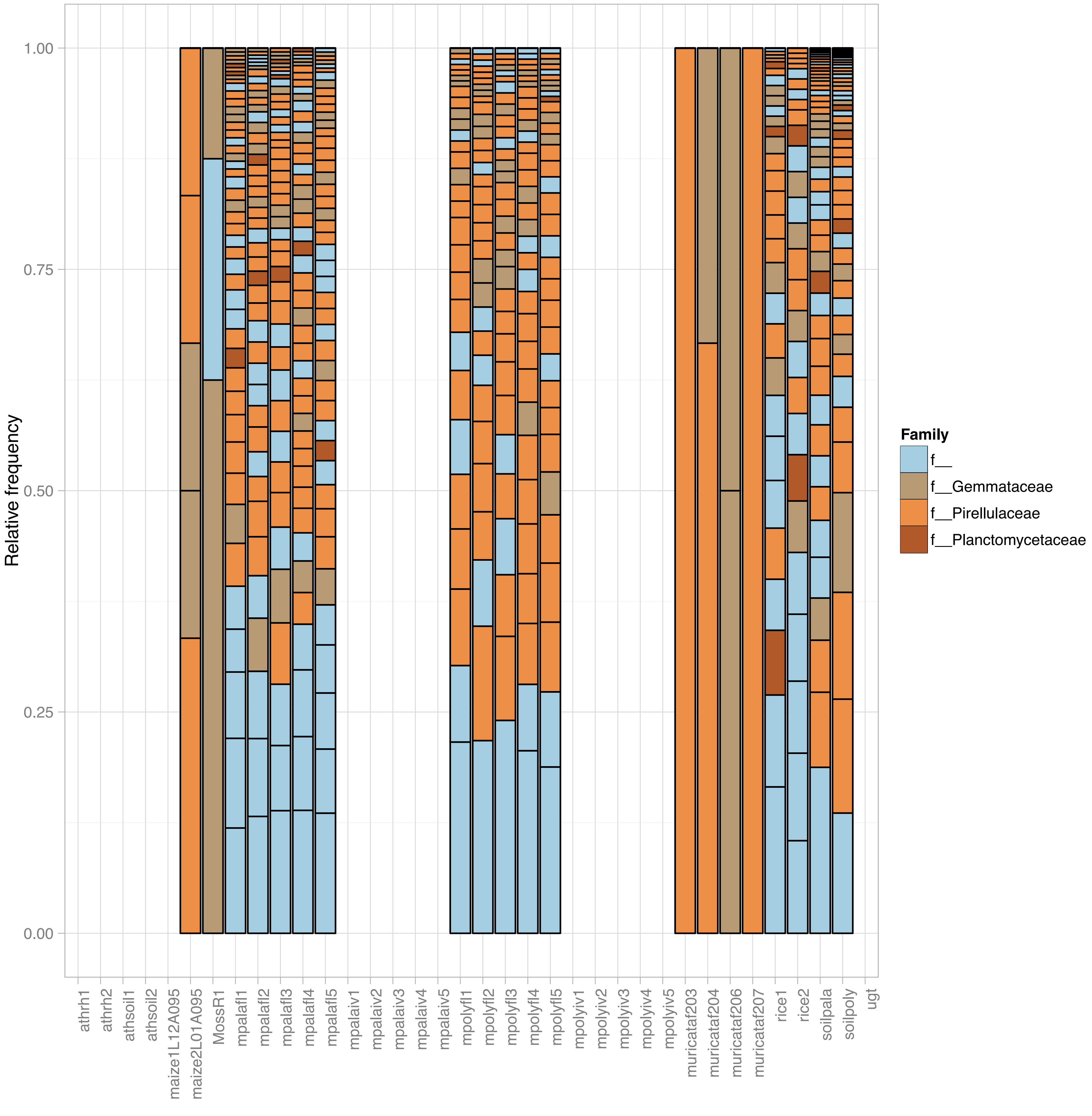

Relative frequency
1.00
0.75
0.50
0.25
0.00
athrh1
athrh2
athsoil1
athsoil2
maize1L12A095
maize2L01A095
MossR1
mpalafl1
mpalafl2
mpalafl3
mpalafl4
mpalafl5
mpalaiv1
mpalaiv2
mpalaiv3
mpalaiv4
mpalaiv5
mpolyfl1
mpolyfl2
mpolyfl3
mpolyfl4
mpolyfl5
mpolyiv1
mpolyiv2
mpolyiv3
mpolyiv4
mpolyiv5
muricataf203
muricataf204
muricataf206
muricataf207
rice1
rice2
soilpala
soilpoly
ugt
Family
f__
f__Gemmataceae
f__Pirellulaceae
f__Planctomycetaceae

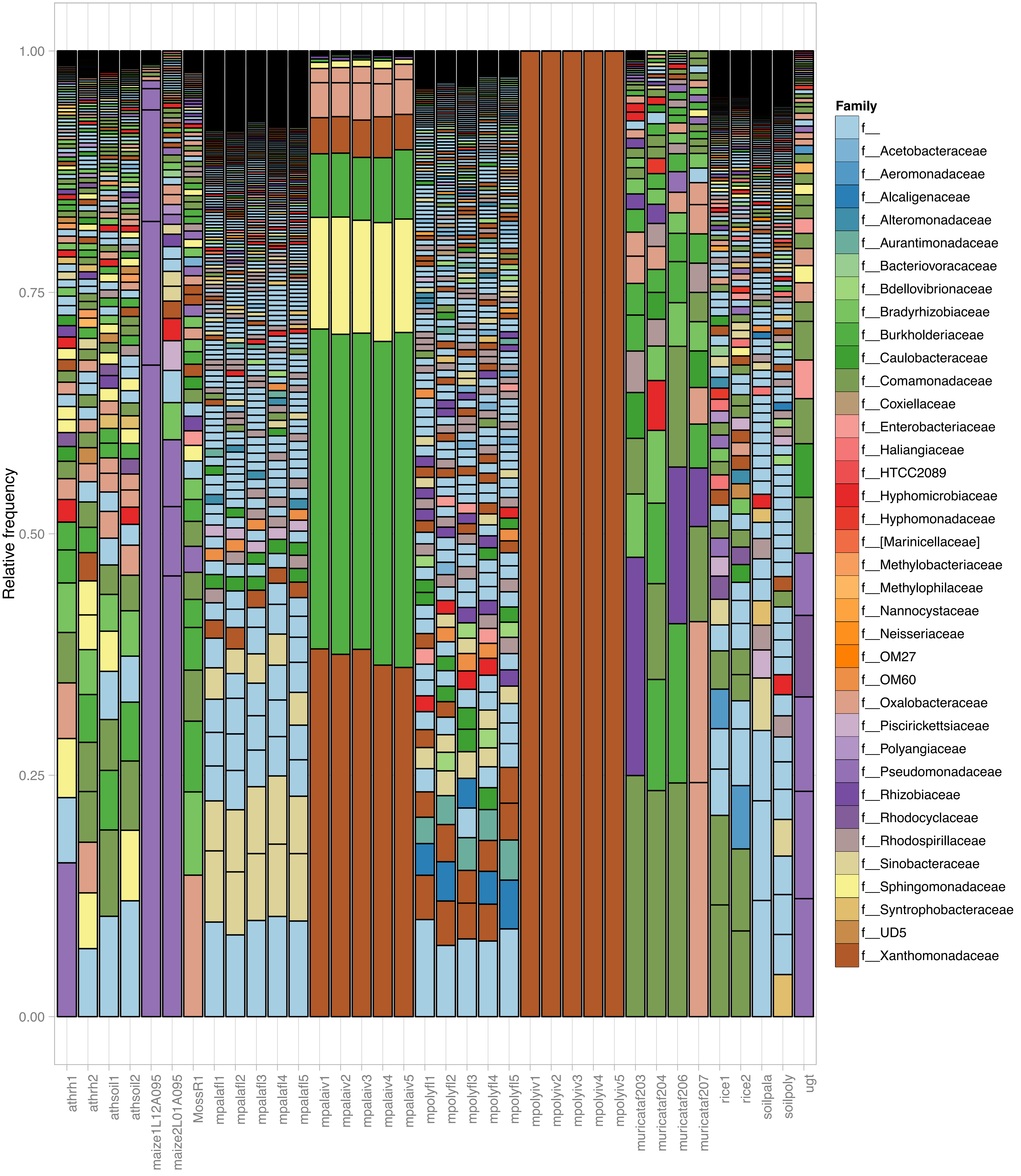

Relative frequency
1.00
0.75
0.50
0.25
0.00
athrh1
athrh2
athsoil1
athsoil2
maize1L12A095
maize2L01A095
MossR1
mpalafl1
mpalafl2
mpalafl3
mpalafl4
mpalafl5
mpalaiv1
mpalaiv2
mpalaiv3
mpalaiv4
mpalaiv5
mpolyfl1
mpolyfl2
mpolyfl3
mpolyfl4
mpolyfl5
mpolyiv1
mpolyiv2
mpolyiv3
mpolyiv4
mpolyiv5
muricataf203
muricataf204
muricataf206
muricataf207
rice1
rice2
soilpala
soilpoly
ugt
Family
f__
f__Acetobacteraceae
f__Aeromonadaceae
f__Alcaligenaceae
f__Alteromonadaceae
f__Aurantimonadaceae
f__Bacteriovoracaceae
f__Bdellovibrionaceae
f__Bradyrhizobiaceae
f__Burkholderiaceae
f__Caulobacteraceae
f__Comamonadaceae
f__Coxiellaceae
f__Enterobacteriaceae
f__Haliangiaceae
f__HTCC2089
f__Hyphomicrobiaceae
f__Hyphomonadaceae
f__[Marinicellaceae]
f__Methylobacteriaceae
f__Methylophilaceae
f__Nannocystaceae
f__Neisseriaceae
f__OM27
f__OM60
f__Oxalobacteraceae
f__Piscirickettsiaceae
f__Polyangiaceae
f__Pseudomonadaceae
f__Rhizobiaceae
f__Rhodocyclaceae
f__Rhodospirillaceae
f__Sinobacteraceae
f__Sphingomonadaceae
f__Syntrophobacteraceae
f__UD5
f__Xanthomonadaceae

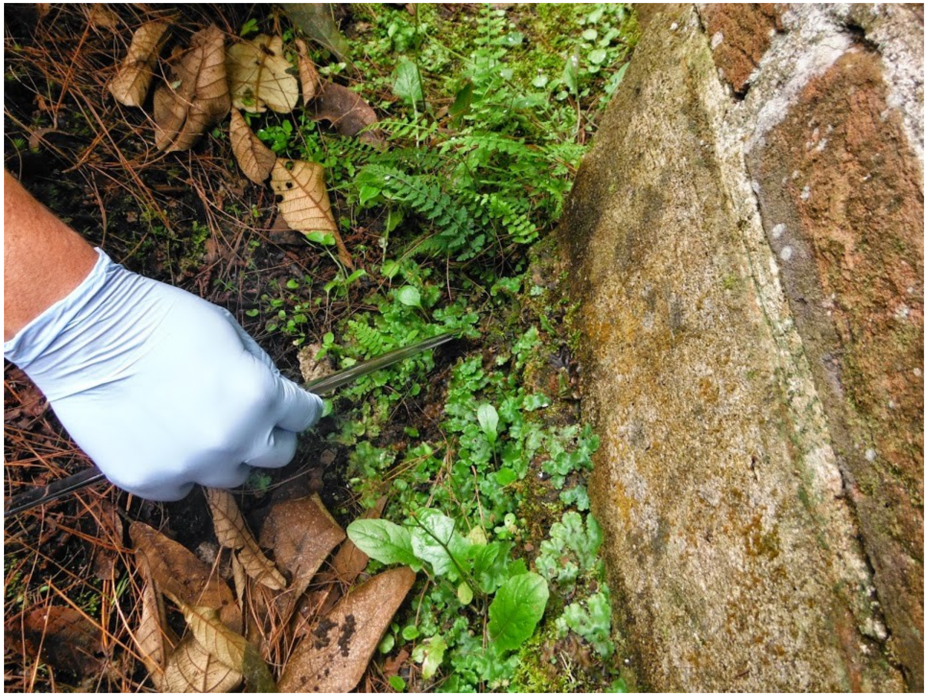
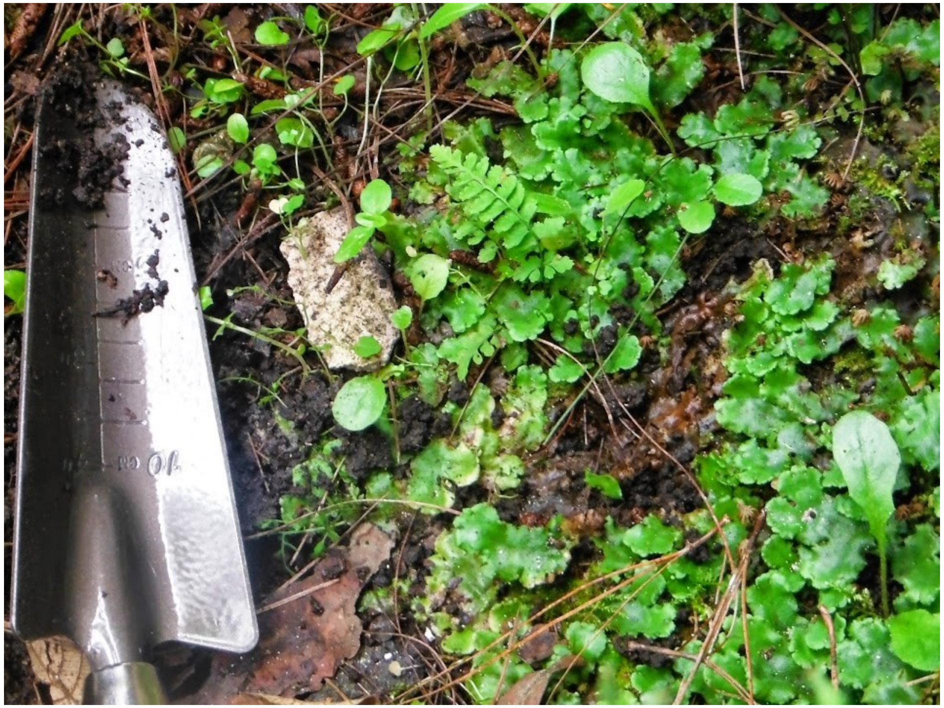

Marchantia paleacea & *Marchantia polymorpha* wild samples collect environments

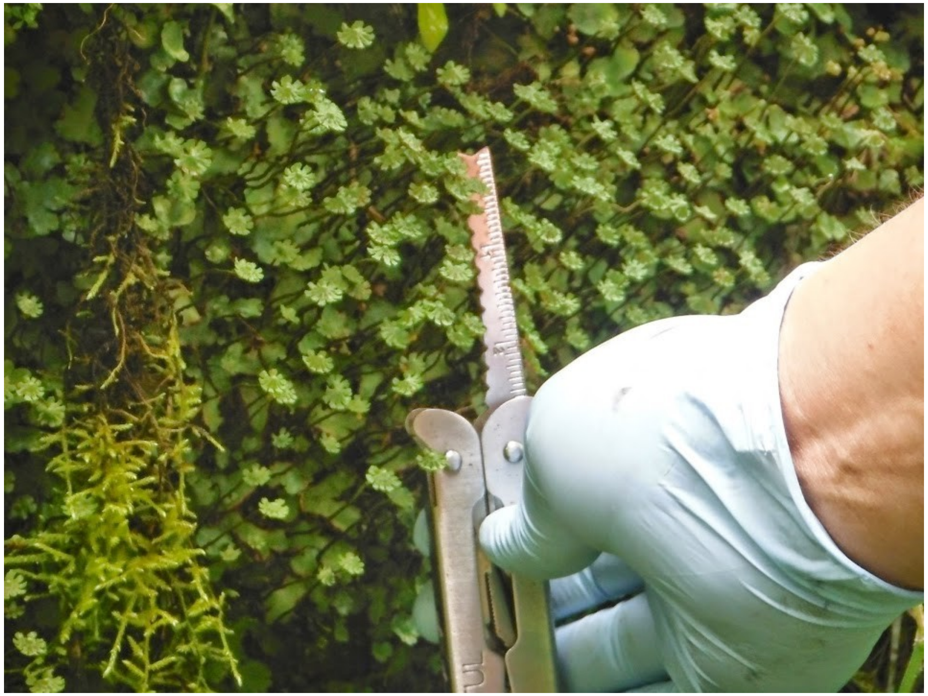
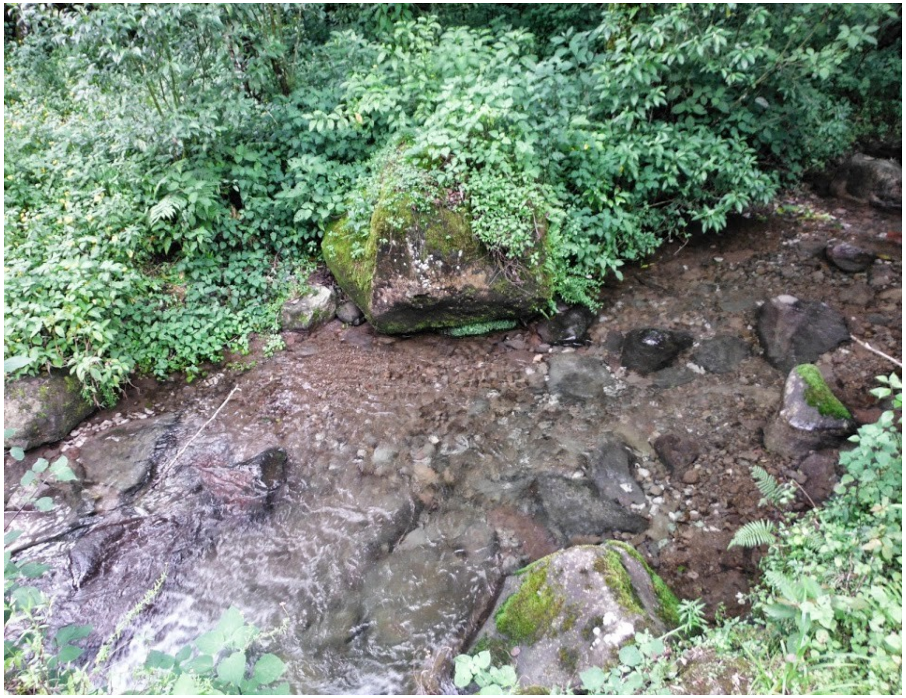

Select a Metric: observed_otus **Select a Category:** Treatment

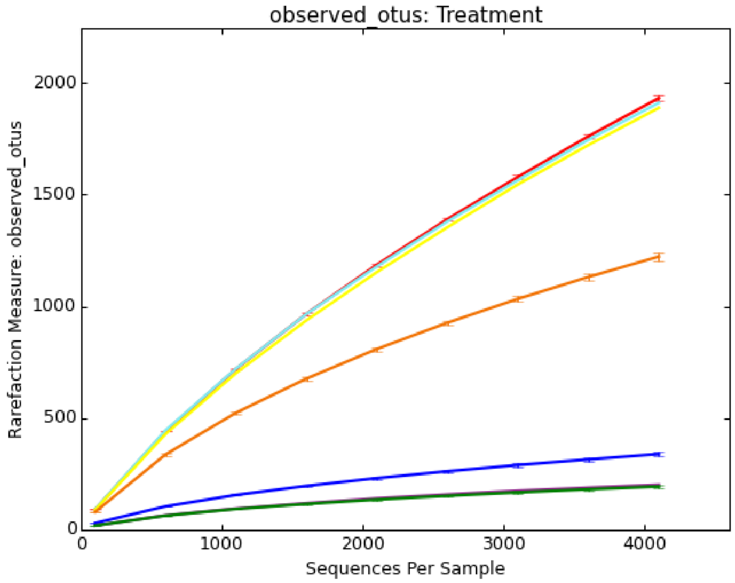

Show Categories:

Legend

mpalafl
mpalaiv
mpolyfl
mpolyiv
mpolyivpolymorpha
soilpala
soilpoly

If the lines for some categories do not extend all the way to the right end of the x-axis, that means that at least one of the samples in that category does not have that many sequences.

Treatment	Seqs/Sample	chao1 Ave.	chao1 Err.	observed_otus Ave.	observed_otus Err.
mpalafl	100.0	746.996	25.205	91.095	0.334
mpalafl	600.0	1943.451	32.305	440.555	1.077
mpalafl	1100.0	2664.429	45.277	719.710	2.587
mpalafl	1600.0	3305.821	51.395	966.033	4.448
mpalafl	2100.0	3820.115	54.242	1186.257	6.388
mpalafl	2600.0	4316.762	57.516	1389.790	7.133
mpalafl	3100.0	4793.768	43.153	1578.277	9.344
mpalafl	3600.0	5269.535	77.467	1759.953	11.321
mpalafl	4100.0	5670.192	53.585	1931.068	12.571
mpalaiv	100.0	108.634	2.090	30.138	0.640
mpalaiv	600.0	267.604	12.508	105.046	2.064
mpalaiv	1100.0	371.314	16.664	156.050	2.986
mpalaiv	1600.0	439.371	20.902	196.350	4.618
mpalaiv	2100.0	504.258	23.689	231.134	4.839
mpalaiv	2600.0	559.395	31.562	261.952	6.911
mpalaiv	3100.0	609.140	38.320	289.896	7.734
mpalaiv	3600.0	655.316	54.545	314.956	9.771
mpalaiv	4100.0	698.190	64.660	339.050	10.456
mpolyfl	100.0	391.899	18.074	79.956	0.836
mpolyfl	600.0	1046.943	30.194	337.260	3.965
mpolyfl	1100.0	1410.349	21.817	523.766	5.343
mpolyfl	1600.0	1649.697	29.206	676.522	8.372
mpolyfl	2100.0	1868.185	31.453	809.094	8.612
mpolyfl	2600.0	2063.114	37.672	926.366	11.488
mpolyfl	3100.0	2235.602	58.426	1033.044	13.636
mpolyfl	3600.0	2392.467	66.027	1130.936	14.350
mpolyfl	4100.0	2538.128	89.823	1222.258	16.626
mpolyiv	100.0	58.619	3.139	18.750	0.313
mpolyiv	600.0	159.077	6.822	63.635	0.965
mpolyiv	1100.0	203.600	9.850	93.280	2.760
mpolyiv	1600.0	238.931	10.984	115.715	3.123
mpolyiv	2100.0	263.218	13.679	135.403	4.391
mpolyiv	2600.0	292.747	14.467	152.222	4.683

mpolyiv	3100.0	315.615	17.522	167.347	5.280
mpolyiv	3600.0	337.288	23.191	181.175	6.046
mpolyiv	4100.0	349.906	23.476	193.650	6.549
mpolyivpolymorpha	100.0	53.677	nan	18.540	nan
mpolyivpolymorpha	600.0	167.410	nan	64.920	nan
mpolyivpolymorpha	1100.0	211.194	nan	95.030	nan
mpolyivpolymorpha	1600.0	253.161	nan	118.890	nan
mpolyivpolymorpha	2100.0	282.723	nan	141.080	nan
mpolyivpolymorpha	2600.0	298.806	nan	158.150	nan
mpolyivpolymorpha	3100.0	334.248	nan	174.990	nan
mpolyivpolymorpha	3600.0	336.703	nan	188.360	nan
mpolyivpolymorpha	4100.0	353.492	nan	201.130	nan
soilpala	100.0	746.492	nan	89.280	nan
soilpala	600.0	1924.456	nan	428.680	nan
soilpala	1100.0	2720.898	nan	700.970	nan
soilpala	1600.0	3315.195	nan	937.270	nan
soilpala	2100.0	3899.911	nan	1152.880	nan
soilpala	2600.0	4354.659	nan	1353.380	nan
soilpala	3100.0	4852.526	nan	1544.810	nan
soilpala	3600.0	5291.179	nan	1720.960	nan
soilpala	4100.0	5723.614	nan	1887.260	nan
soilpoly	100.0	733.994	nan	92.510	nan
soilpoly	600.0	1804.843	nan	445.040	nan
soilpoly	1100.0	2527.358	nan	721.390	nan
soilpoly	1600.0	3207.716	nan	965.630	nan
soilpoly	2100.0	3721.825	nan	1178.170	nan
soilpoly	2600.0	4272.824	nan	1379.370	nan
soilpoly	3100.0	4762.266	nan	1563.880	nan
soilpoly	3600.0	5158.644	nan	1744.720	nan
soilpoly	4100.0	5525.320	nan	1909.810	nan

In [3]:
```
library(ggplot2)
library(vegan)
library(phyloseq)

ls()


p1 <- plot_richness(marchantia)
p1$data

marchantiafilter = marchantia

marchwosingleton <-  genefilter_sample(marchantiafilter, filterfun_sample(func
tion(x) x > 2), A =
0.1 * nsamples(marchantiafilter))

marchantiaf = prune_taxa(marchwosingleton, marchantiafilter)

p <- plot_richness(marchantiaf)

    p$data

    p


#Muestra 11 Mpala VL Marchantia paleacea raíces vida libre

#Muestra 12 Mpolim VL Marchantia polymorpha raíces vida libre

#Muestra 13 Mpala Suelo Marchantia paleacea suelo

#Muestra 14 Mpolim Suelo Marchantia polymorpha suelo

#Muestra 15 Mpala in vitro Marchantia paleacea in vitro

#Muestra 16 Mpolim in vitro Marchantia polymorpha Tak1 in vitro
```

'abund_table' 'colours' 'dat' 'df' 'dfra' 'getEdgeLength' 'getLabel' 'getPalette' 'GP' 'GP1' 'gp.ord' 'GP.ord' 'GP.ord2' 'GP.ord3' 'GP.ord4' 'gpt' 'gpt2' 'i' 'ig' 'march' 'march0' 'march1f' 'march2' 'march2f' 'march2.sub' 'march3' 'marchantia' 'marchantia2' 'marchantia_data' 'marchantiaf' 'marchantiafilter' 'marchfilter' 'march_ordCAP.ORD' 'march_ordDCA.ORD' 'march_ordunifrac' 'march_ordunifrac.ORD' 'march.scale' 'marchwosingleton' 'N' 'newick' 'ninfa1F' 'otu' 'OTU' 'otu2' 'OTU2' 'p' 'p1' 'p2' 'p3' 'paleto' 'phylum' 'phylum.sum' 'read.newick' 'sampledata' 'taxi' 'taxi2' 'taximat' 'taximat2' 'termDocMatrix' 'tmp' 'top20phyla' 'tree' 'wh0' 'x'

samples	variable	value	se
S11	Observed	4.839100e+04	NA
S12	Observed	2.511300e+04	NA
S13	Observed	4.834000e+04	NA
S14	Observed	4.792200e+04	NA
S16	Observed	4.378000e+03	NA
S15	Observed	1.113000e+04	NA
S11	Chao1	1.597909e+05	1931.94581
S12	Chao1	8.985656e+04	1642.49991
S13	Chao1	1.928886e+05	2589.42503
S14	Chao1	1.861114e+05	2470.47897
S16	Chao1	2.438393e+04	1279.07428
S15	Chao1	6.141860e+04	2037.03681
S11	ACE	1.819408e+05	276.81796
S12	ACE	9.301381e+04	199.69638
S13	ACE	2.207858e+05	285.41501
S14	ACE	2.149299e+05	284.22211
S16	ACE	2.882827e+04	82.09568
S15	ACE	6.930778e+04	136.21263
S11	Shannon	9.060710e+00	NA
S12	Shannon	7.890812e+00	NA
S13	Shannon	9.321081e+00	NA
S14	Shannon	9.291682e+00	NA
S16	Shannon	2.133400e+00	NA
S15	Shannon	4.325586e+00	NA
S11	Simpson	9.990366e-01	NA
S12	Simpson	9.973484e-01	NA
S13	Simpson	9.994187e-01	NA
S14	Simpson	9.994185e-01	NA
S16	Simpson	7.602520e-01	NA
S15	Simpson	9.218630e-01	NA
S11	InvSimpson	1.037955e+03	NA
S12	InvSimpson	3.771292e+02	NA
S13	InvSimpson	1.720173e+03	NA
S14	InvSimpson	1.719551e+03	NA
S16	InvSimpson	4.171047e+00	NA
S15	InvSimpson	1.279803e+01	NA

samples	variable	value	se
S11	Observed	1.007000e+04	NA
S12	Observed	6.188000e+03	NA
S13	Observed	8.367000e+03	NA
S14	Observed	8.313000e+03	NA
S16	Observed	4.020000e+02	NA
S15	Observed	1.162000e+03	NA
S11	Chao1	1.142056e+04	87.796772
S12	Chao1	7.048666e+03	74.272460
S13	Chao1	9.696032e+03	92.027879
S14	Chao1	9.593766e+03	90.759364
S16	Chao1	4.220000e+02	10.562775
S15	Chao1	1.229500e+03	24.549985
S11	ACE	1.087779e+04	45.497629
S12	ACE	6.649053e+03	37.083800
S13	ACE	9.109782e+03	41.750728
S14	ACE	9.003207e+03	41.479007
S16	ACE	4.094762e+02	9.005443
S15	ACE	1.176787e+03	15.469548
S11	Shannon	7.832222e+00	NA
S12	Shannon	7.076396e+00	NA
S13	Shannon	7.850556e+00	NA
S14	Shannon	7.847346e+00	NA
S16	Shannon	1.848861e+00	NA
S15	Shannon	3.418550e+00	NA
S11	Simpson	9.982968e-01	NA
S12	Simpson	9.963685e-01	NA
S13	Simpson	9.987744e-01	NA
S14	Simpson	9.987979e-01	NA
S16	Simpson	7.474239e-01	NA
S15	Simpson	9.033814e-01	NA
S11	InvSimpson	5.871345e+02	NA
S12	InvSimpson	2.753661e+02	NA
S13	InvSimpson	8.159425e+02	NA
S14	InvSimpson	8.318664e+02	NA
S16	InvSimpson	3.959203e+00	NA
S15	InvSimpson	1.034997e+01	NA

```
Warning message:
“Removed 30 rows containing missing values (geom_errorbar).”
```

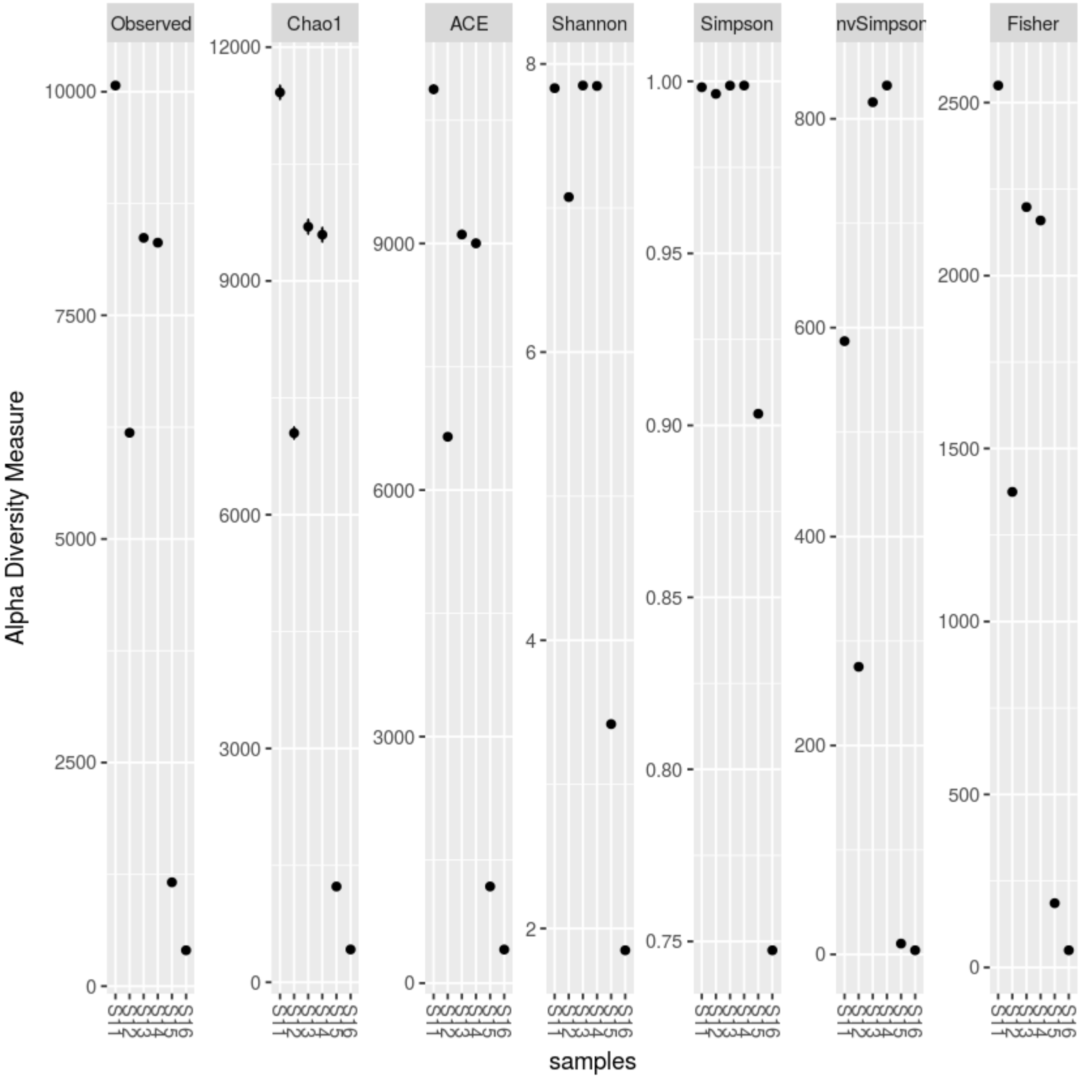

In [14]:
```
library(phyloseq)
library(vegan)
library(ggplot2)
library(vegan)
library(dplyr)
library(scales)
library(grid)
library(reshape2)
library(Rcpp)
require(phytools)
options(warn=-1)

otu <- as.matrix(read.table("marchantia_jul17.tab", header=T, row.names = 1))
OTU = otu_table(otu, taxa_are_rows=T)
march = phyloseq(OTU)
marchdata = read.table("marchantia_metadata.txt", header=T, row.names=1)
sampledata = sample_data(data.frame(id=marchdata$id,habitat=marchdata$habitat,
species=marchdata$species, row.names=sample_names(march)))
require(phytools)
source("../read.newick.R")
tree<-read.newick("marchantia_sub_17jul_align.tree")
tree <- collapse.singles(tree)
march = phyloseq (OTU, sampledata, phy_tree(tree))
march
wh0 = genefilter_sample(march, filterfun_sample(function(x) x > 2), A=0.2*nsam
ples(march))
march_subset = prune_taxa(wh0,march)
```

```
phyloseq-class experiment-level object
otu_table()   OTU Table:         [ 18295 taxa and 24 samples ]
sample_data() Sample Data:       [ 24 samples by 3 sample variables ]
phy_tree()    Phylogenetic Tree: [ 18295 tips and 18293 internal nodes ]
```

Constrained Analysis of Principal Coordinates (CAP)

In [138]:
```
march_ordCAP.ORD <- ordinate(march_subset, "CAP", "unifrac", ~ habitat + speci
es )

march_ordCAP.ORD

cap_plot <- plot_ordination(march_subset, march_ordCAP.ORD, color="habitat", a
xes =c(1,2)) + aes(shape= species) + geom_point(aes(colour = habitat), alpha=0
.4, size=4) + geom_point(colour = "grey90", size=1.5) + theme_bw()

arrowmat <- vegan::scores(march_ordCAP.ORD, display="bp")
arrowdf <- data.frame(labels=rownames(arrowmat), arrowmat)

# Define the arrow aesthetic mapping
arrow_map <- aes(xend = CAP1,
    yend = CAP2,
    x = 0,
    y = 0,
    shape = NULL,
    color = NULL,
    label = labels)

label_map <- aes(x = 1.3 * CAP1,
    y = 1.3 * CAP2,
    shape = NULL,
    color = NULL,
    label = labels)

arrowhead = arrow(length = unit(0.02, "npc"))

cap_plot  +
  geom_segment(
    mapping = arrow_map,
    size = .5,
    data = arrowdf,
    color = "gray",
    arrow = arrowhead
  ) +
  geom_text(
    mapping = label_map,
    size = 2,
    data = arrowdf,
    show.legend = FALSE
  )

an <- anova(march_ordCAP.ORD, permutations=9999)
an
```

```
Call: capscale(formula = distance ~ habitat + species, data = data)

              Inertia Proportion Eigenvals Rank
Total         6.32235    1.00000   6.33528     
Constrained   5.27315    0.83405   5.27316    3
Unconstrained 1.04920    0.16595   1.06212   15
Imaginary                         -0.01293    5
Inertia is squared Unknown distance

Eigenvalues for constrained axes:
 CAP1  CAP2  CAP3 
4.382 0.746 0.146 

Eigenvalues for unconstrained axes:
  MDS1   MDS2   MDS3   MDS4   MDS5   MDS6   MDS7   MDS8   MDS9  MDS10  MDS11 
0.8965 0.0968 0.0246 0.0111 0.0089 0.0084 0.0049 0.0037 0.0028 0.0024 0.0013 
 MDS12  MDS13  MDS14  MDS15 
0.0006 0.0001 0.0001 0.0000 
```

	Df	SumOfSqs	F	Pr(>F)
**Model**	3	5.273152	33.50594	1e-04
**Residual**	20	1.049198	NA	NA

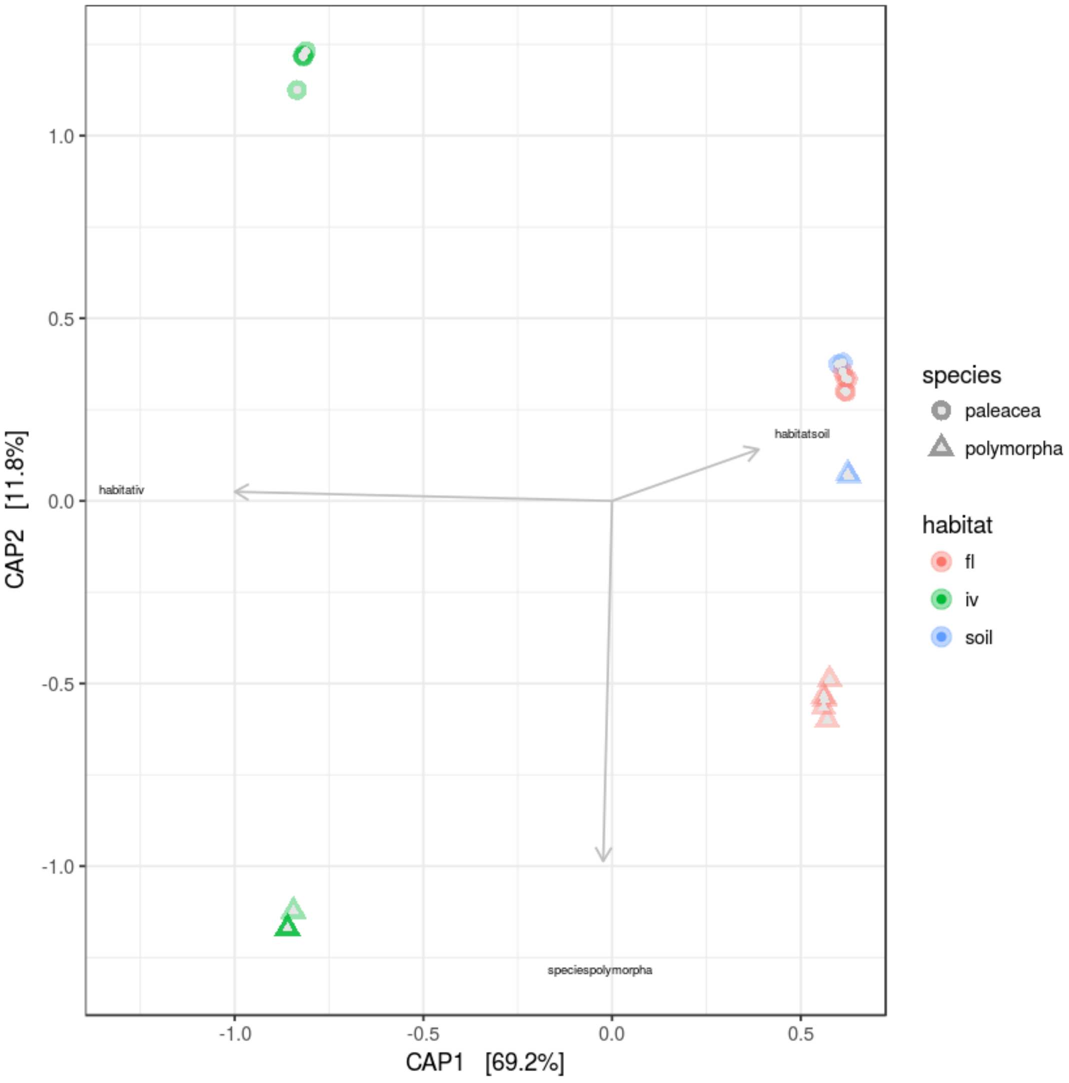

En el cluster se instaló la última varsión de R y phyloseq (devtools) y ya funciona el wunifrac. De la ANOVA sale lo siguiente:

Permutation test for capscale under reduced model Permutation: free Number of permutations: 999

Model: capscale(formula = distance ~ habitat + species, data = data) Df SumOfSqs F Pr(>F)
Model 3 1.27173 36.801 0.001 ***

Residual 20 0.23038

Signif. codes: 0 '*' **0.001** '' 0.01 '*' 0.05 '.' 0.1 ' ' 1

In [12]:
```
march_ordCAP.ORD <- ordinate(march_subset, "CAP", "wunifrac", ~ habitat + spec
ies)

cap_plot <- plot_ordination(march_subset, march_ordCAP.ORD, color="habitat", a
xes =c(1,2)) + aes(shape= species) +
geom_point(aes(colour = habitat), alpha=0.7, size=4) + theme_light()

arrowmat <- vegan::scores(march_ordCAP.ORD, display="bp")
arrowdf <- data.frame(labels=rownames(arrowmat), arrowmat)

# Define the arrow aesthetic mapping
arrow_map <- aes(xend = CAP1,
    yend = CAP2,
    x = 0,
    y = 0,
    shape = NULL,
    color = NULL,
    label = labels)

label_map <- aes(x = 1.3 * CAP1,
    y = 1.3 * CAP2,
    shape = NULL,
    color = NULL,
    label = labels)

arrowhead = arrow(length = unit(0.02, "npc"))

cap_plot  +
  geom_segment(
    mapping = arrow_map,
    size = 1,
    data = arrowdf,
    color = "gray",
    arrow = arrowhead
  ) +
  geom_text(
    mapping = label_map,
    size = 3,
    data = arrowdf,
    show.legend = FALSE
  )

anova(march_ordCAP.ORD,permutations=9999)

ggsave("CAPwunifracmarchantia.pdf", width = 20, height = 20, units = "cm")
```

	Df	SumOfSqs	F	Pr(>F)
**Model**	3	0.7666061	37.09582	1e-04
**Residual**	20	0.1377704	NA	NA

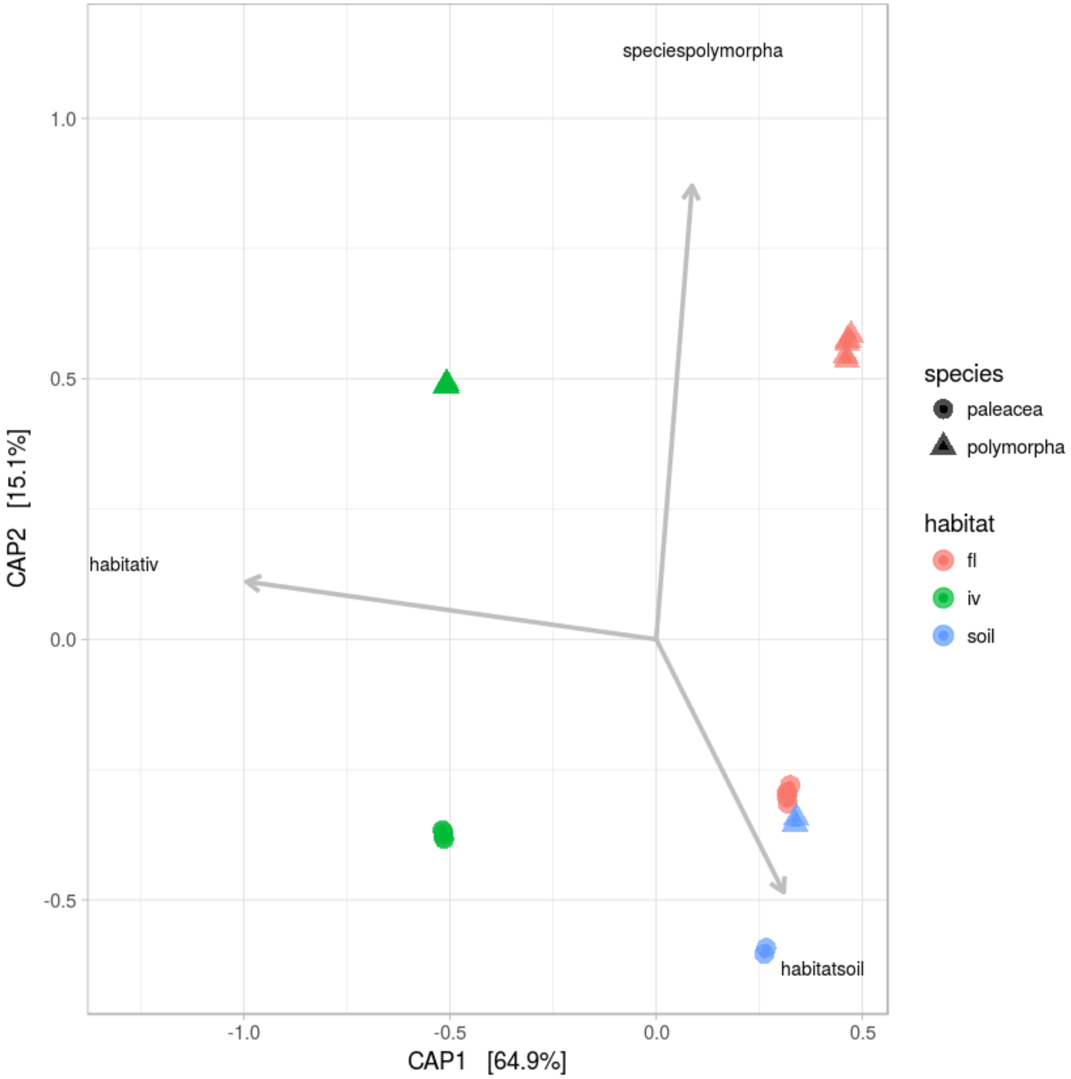

In [17]:

```
library(ape)
library(plyr)
dist_methods <- unlist(distanceMethodList)

#Remove the two distance-methods that require a tree, and the generic custom m
ethod that requires user-defined distance arguments.
# These require tree
#dist_methods[(1:3)]
dist_methods[(3)]
# Remove them from the vector
#dist_methods <- dist_methods[-(1:3)]
dist_methods <- dist_methods[-(3)]
# This is the user-defined method:
dist_methods["designdist"]
# Remove the user-defined distance
dist_methods = dist_methods[-which(dist_methods=="ANY")]

#NMDS



plist <- vector("list", length(dist_methods))
print(distanceMethodList)

for( i in dist_methods ){
 # Calculate distance matrix
    iDist <- distance(march_subset, method=i)
    # Calculate ordination
    iMDS  <- ordinate(march_subset, "NMDS", distance=iDist)
    ## Make plot
    p <- NULL
    # Don't carry over previous plot (if error, p will be blank)
    # Create plot, store as temp variable, p
    p <- plot_ordination(march_subset, iMDS, color="habitat", shape="species")
    p <- p + ggtitle(paste("NMDS using distance method ", i, sep=""))
    plist[[i]] = p
}

df = ldply(plist, function(x) x$data)
names(df)[1] <- "distance"
    #aqui es importante fijarse como se llaman los ejes de las distintas metri
cas para graficar correctamente (DCA, NMDS, Axis.1, etc) puedes verlo con head
(df) para ver los ejes

 p = ggplot(df, aes(NMDS1, NMDS2, color=habitat, shape=species))
 p = p + geom_point(size=3, alpha=0.5)
 p = p + facet_wrap(~distance, scales="free")
 p = p + ggtitle("NMDS on various distance metrics for Marchantia")
 p
```

```
--------------------------------------------------------------------------------
You have loaded plyr after dplyr - this is likely to cause problems.
If you need functions from both plyr and dplyr, please load plyr first, then dp
lyr:
library(plyr); library(dplyr)
--------------------------------------------------------------------------------

Attaching package: ‘plyr’

The following object is masked from ‘package:maps’:

    ozone

The following objects are masked from ‘package:dplyr’:

    arrange, count, desc, failwith, id, mutate, rename, summarise,
    summarize
```

DPCoA: 'dpcoa'

designdist: 'ANY'

```
$UniFrac
[1] "unifrac"  "wunifrac"

$DPCoA
[1] "dpcoa"

$JSD
[1] "jsd"

$vegdist
 [1] "manhattan"  "euclidean"  "canberra"   "bray"       "kulczynski"
 [6] "jaccard"    "gower"      "altGower"   "morisita"   "horn"
[11] "mountford"  "raup"       "binomial"   "chao"       "cao"

$betadiver
 [1] "w"   "-1"  "c"   "wb"  "r"   "I"   "e"   "t"   "me"  "j"   "sor" "m"
[13] "-2"  "co"  "cc"  "g"   "-3"  "l"   "19"  "hk"  "rlb" "sim" "gl"  "z"

$dist
[1] "maximum"   "binary"    "minkowski"

$designdist
[1] "ANY"

Run 0 stress 9.9846e-05
Run 1 stress 0.0008450643
Run 2 stress 0.001187496
Run 3 stress 0.001068756
Run 4 stress 0.0003024217
... Procrustes: rmse 0.07874547  max resid 0.1218313
Run 5 stress 0.0002557205
... Procrustes: rmse 0.08998009  max resid 0.1391126
Run 6 stress 0.0005340013
... Procrustes: rmse 0.07444577  max resid 0.1150959
Run 7 stress 9.740106e-05
... New best solution
... Procrustes: rmse 0.09282578  max resid 0.1438853
Run 8 stress 0.001611553
Run 9 stress 0.00115336
Run 10 stress 0.0001794957
... Procrustes: rmse 0.01846158  max resid 0.02859146
Run 11 stress 0.0008987171
Run 12 stress 0.001054118
Run 13 stress 0.001025019
Run 14 stress 0.001087963
Run 15 stress 0.002219088
Run 16 stress 0.0012751
Run 17 stress 0.002369311
Run 18 stress 0.0002780724
... Procrustes: rmse 0.02552544  max resid 0.03920545
Run 19 stress 0.0005793681
... Procrustes: rmse 0.01078473  max resid 0.01422053
Run 20 stress 0.001165253
*** No convergence -- monoMDS stopping criteria:
    19: no. of iterations >= maxit
     1: stress < smin
Run 0 stress 7.325019e-05
Run 1 stress 9.547246e-05
... Procrustes: rmse 0.01424873  max resid 0.02092586
Run 2 stress 9.499654e-05
... Procrustes: rmse 0.08987489  max resid 0.136251
Run 3 stress 9.482006e-05
... Procrustes: rmse 0.0686725  max resid 0.1011785
```

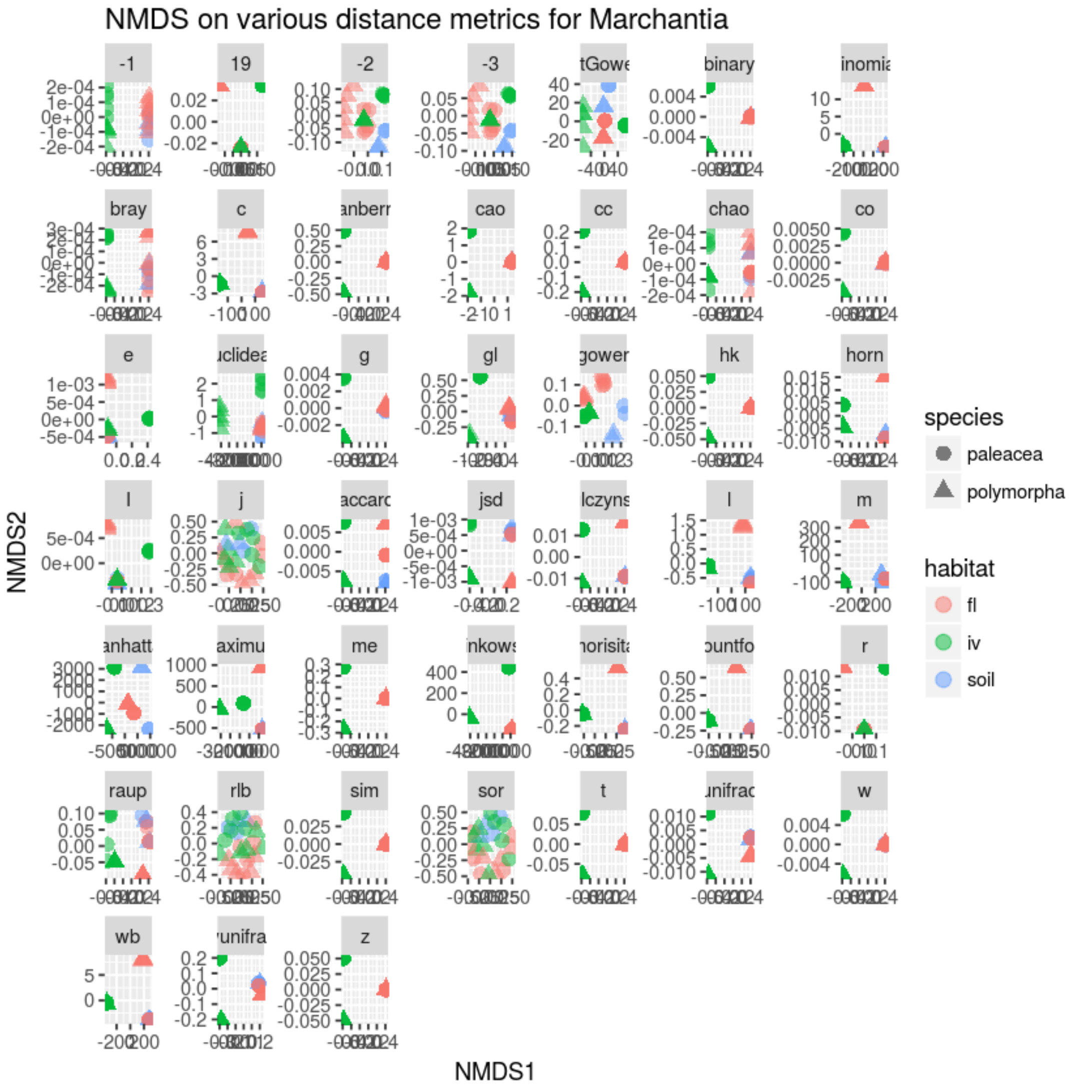

In [17]:

```
ggsave("nmds_all_distances.pdf", p, width = 40, height = 20, units = "cm")
```

In [167]:
```
IMDS <- ordinate(march_subset, "NMDS", distance="unifrac")
p2 = plot_ordination(march_subset, IMDS, color="habitat", shape="species") + g
eom_point(size=3, alpha=0.5) + ggtitle("NMDS unweighted Unifrac")
p2 + theme_light()

xMDS <- ordinate(march_subset, "NMDS", distance="wunifrac")
p3 = plot_ordination(march_subset, xMDS, color="habitat", shape="species") + g
eom_point(size=3, alpha=0.5) + ggtitle("NMDS weighted Unifrac")
p3 + theme_light()
```

```
Run 0 stress 9.476405e-05
Run 1 stress 0.0009255761
Run 2 stress 0.0008274472
Run 3 stress 0.000214317
... Procrustes: rmse 0.08821371  max resid 0.1365173
Run 4 stress 0.000196336
... Procrustes: rmse 0.07462991  max resid 0.1153821
Run 5 stress 0.0005636465
... Procrustes: rmse 0.0745715  max resid 0.1153063
Run 6 stress 0.001064922
Run 7 stress 0.000488189
... Procrustes: rmse 0.08489163  max resid 0.1311091
Run 8 stress 9.842825e-05
... Procrustes: rmse 0.02488392  max resid 0.03592335
Run 9 stress 0.0007842193
Run 10 stress 0.001297794
Run 11 stress 0.0001160674
... Procrustes: rmse 0.04658355  max resid 0.07128874
Run 12 stress 9.801771e-05
... Procrustes: rmse 0.02885821  max resid 0.04370521
Run 13 stress 9.740117e-05
... Procrustes: rmse 0.09308339  max resid 0.1442727
Run 14 stress 0.001016505
Run 15 stress 0.000200554
... Procrustes: rmse 0.06011058  max resid 0.09254245
Run 16 stress 9.676243e-05
... Procrustes: rmse 0.09045428  max resid 0.1402053
Run 17 stress 9.919729e-05
... Procrustes: rmse 0.02429737  max resid 0.03668175
Run 18 stress 0.0008268107
Run 19 stress 0.001253415
Run 20 stress 0.0002239878
... Procrustes: rmse 0.08808431  max resid 0.1363106
*** No convergence -- monoMDS stopping criteria:
    15: no. of iterations >= maxit
     5: stress < smin
```

```
Run 0 stress 9.794819e-05
Run 1 stress 9.86319e-05
... Procrustes: rmse 0.06063744  max resid 0.08795023
Run 2 stress 8.981458e-05
... New best solution
... Procrustes: rmse 0.09729401  max resid 0.1612128
Run 3 stress 9.934872e-05
... Procrustes: rmse 0.04850523  max resid 0.09046514
Run 4 stress 9.351864e-05
... Procrustes: rmse 0.0777921  max resid 0.1157023
Run 5 stress 9.658276e-05
... Procrustes: rmse 0.03076623  max resid 0.05567668
Run 6 stress 9.42247e-05
... Procrustes: rmse 0.03298301  max resid 0.05929062
Run 7 stress 0.0001593874
... Procrustes: rmse 0.04186053  max resid 0.07719109
Run 8 stress 9.389728e-05
... Procrustes: rmse 0.04156354  max resid 0.07326465
Run 9 stress 8.835452e-05
... New best solution
... Procrustes: rmse 0.02949213  max resid 0.05322198
Run 10 stress 9.201946e-05
... Procrustes: rmse 0.02087387  max resid 0.03906592
Run 11 stress 9.186964e-05
... Procrustes: rmse 0.02280506  max resid 0.04208302
Run 12 stress 9.653521e-05
... Procrustes: rmse 0.01636171  max resid 0.03026727
Run 13 stress 9.333249e-05
... Procrustes: rmse 0.04073673  max resid 0.06681446
Run 14 stress 9.471841e-05
... Procrustes: rmse 0.009498462  max resid 0.01746721
Run 15 stress 9.047169e-05
... Procrustes: rmse 0.01660133  max resid 0.0308743
Run 16 stress 6.736933e-05
... New best solution
... Procrustes: rmse 0.01707078  max resid 0.03142546
Run 17 stress 8.632247e-05
... Procrustes: rmse 0.09420935  max resid 0.1420937
Run 18 stress 9.496583e-05
... Procrustes: rmse 0.01928512  max resid 0.02195069
Run 19 stress 9.932495e-05
... Procrustes: rmse 0.07316493  max resid 0.1241022
Run 20 stress 9.990931e-05
... Procrustes: rmse 0.01181941  max resid 0.02165392
*** No convergence -- monoMDS stopping criteria:
    19: stress < smin
     1: scale factor of the gradient < sfgrmin
```

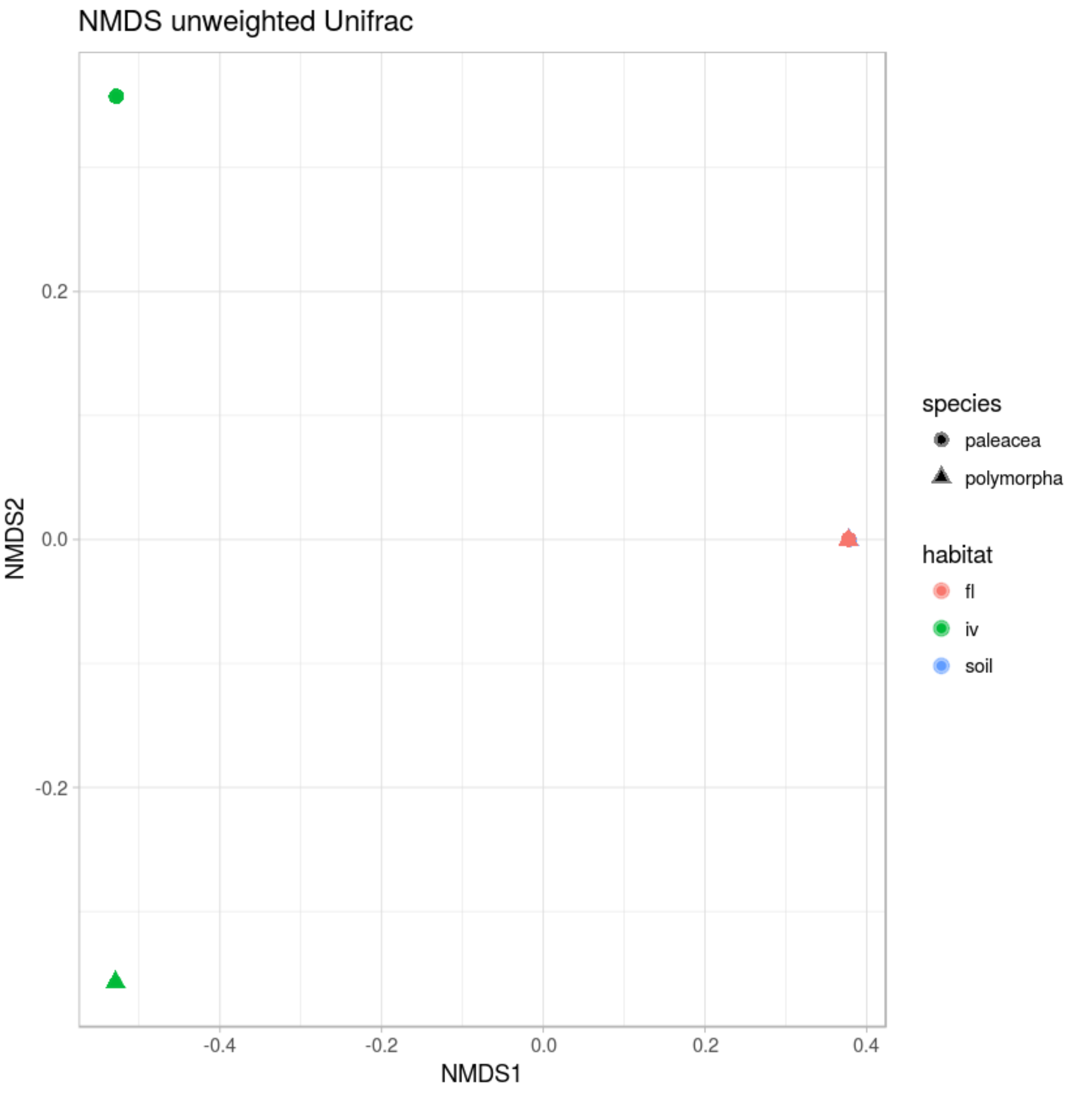
NMDS unweighted Unifrac
NMDS2
0.2
0.0
-0.2
-0.4
-0.2
0.0
0.2
0.4
NMDS1
species
paleacea
polymorpha
habitat
fl
iv
soil

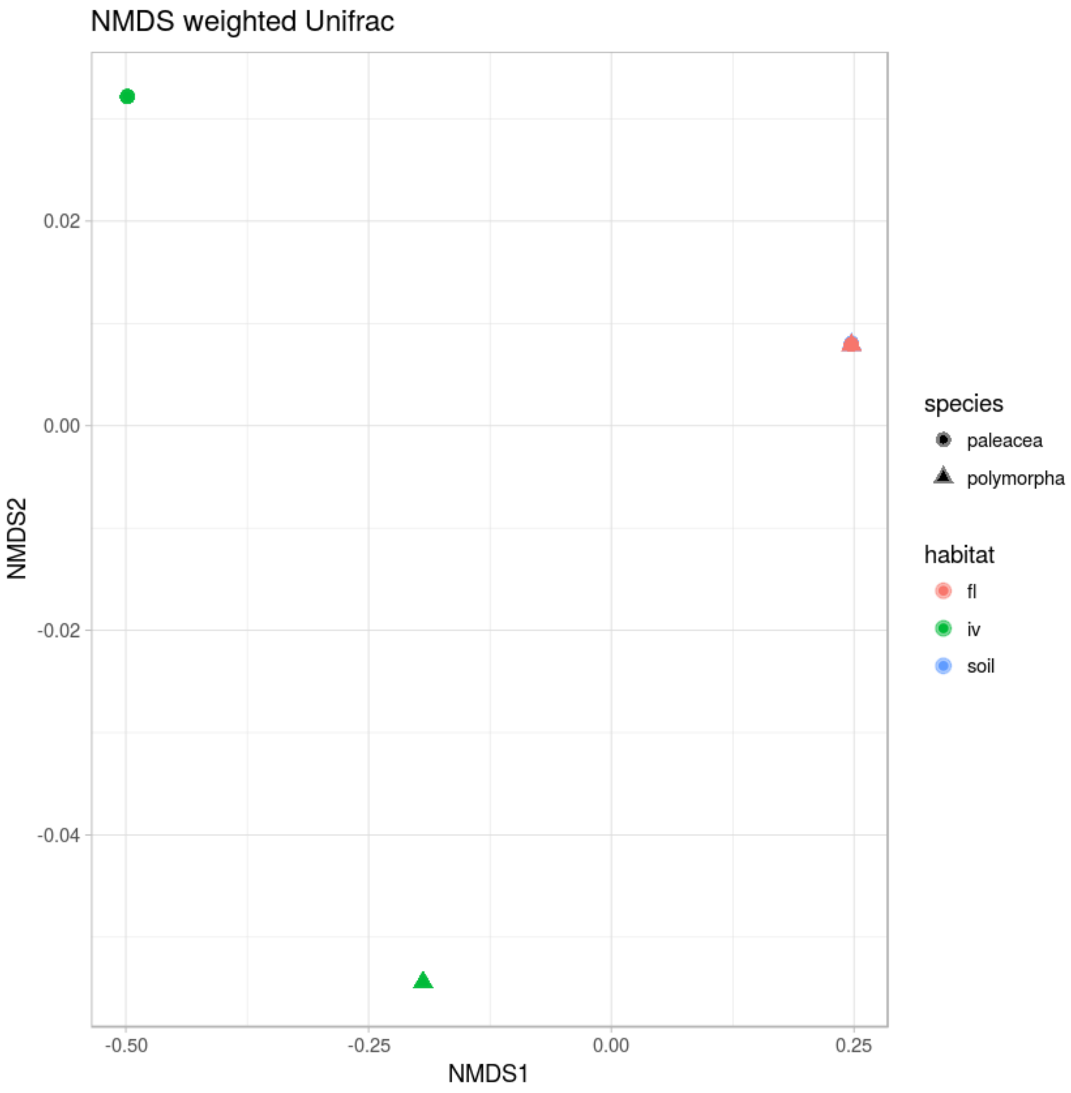
NMDS weighted Unifrac
0.02
0.00
-0.02
-0.04
NMDS2
-0.50
-0.25
0.00
0.25
NMDS1
species
paleacea
polymorpha
habitat
fl
iv
soil

In [39]:
```
sample_data(march_subset)
marchantita <- get_variable(march_subset, "habitat") %in% c("polymorpha", "pal
eacea")
sample_data(march_subset)$marchantita <- factor(marchantita)
```

	id	habitat	species	marchantita
**mpolyiv2**	mpolyiv2	iv	polymorpha	TRUE
**mpolyiv5**	mpolyiv5	iv	polymorpha	TRUE
**soilpala1**	soilpala1	soil	paleacea	TRUE
**soilpala2**	soilpala2	soil	paleacea	TRUE
**mpalafl1**	mpalafl1	fl	paleacea	TRUE
**mpalafl4**	mpalafl4	fl	paleacea	TRUE
**mpalafl5**	mpalafl5	fl	paleacea	TRUE
**soilpoly1**	soilpoly1	soil	polymorpha	TRUE
**soilpoly2**	soilpoly2	soil	polymorpha	TRUE
**mpalafl2**	mpalafl2	fl	paleacea	TRUE
**mpalafl3**	mpalafl3	fl	paleacea	TRUE
**mpolyfl1**	mpolyfl1	fl	polymorpha	TRUE
**mpolyfl3**	mpolyfl3	fl	polymorpha	TRUE
**mpolyfl4**	mpolyfl4	fl	polymorpha	TRUE
**mpolyfl5**	mpolyfl5	fl	polymorpha	TRUE
**mpalaiv2**	mpalaiv2	iv	paleacea	TRUE
**mpalaiv3**	mpalaiv3	iv	paleacea	TRUE
**mpolyfl2**	mpolyfl2	fl	polymorpha	TRUE
**mpalaiv1**	mpalaiv1	iv	paleacea	TRUE
**mpalaiv4**	mpalaiv4	iv	paleacea	TRUE
**mpalaiv5**	mpalaiv5	iv	paleacea	TRUE
**mpolyiv1**	mpolyiv1	iv	polymorpha	TRUE
**mpolyiv3**	mpolyiv3	iv	polymorpha	TRUE
**mpolyiv4**	mpolyiv4	iv	polymorpha	TRUE

In [140]:

```
marchantita <- get_variable(march_subset, "habitat")
sample_data(march_subset)$marchantita <- factor(marchantita)
colorCodes <- levels(marchantita)
library(RColorBrewer)
#sample_data(march_subset)
marchUF <- UniFrac(march_subset, weighted=FALSE)
#colorScale <- colors()[c(26,51,76)]
#colorScale    <-rainbow(length(levels(get_variable(march_subset, "marchantita
"))))
colorScale    <- brewer.pal(length(levels(get_variable(march_subset, "marchant
ita"))),"Dark2")
#colorScale
cols <- colorScale[get_variable(march_subset, "marchantita")]
#cols
#march.tip.labels <- as(get_variable(march_subset, "marchantita"), "character"
)
# This is the actual hierarchical clustering call, specifying average-link clu
stering
march.hclust     <- hclust(marchUF, method="average")
library(dendextend)
dend <- as.dendrogram(march.hclust)
labels_colors(dend) <- cols[order.dendrogram(dend)]
dend %>% set("labels_cex", .75) %>% plot(main="Unifrac unweighted distances",h
oriz=T)
#ggplot(dend, horiz=T)
#plot(march.hclust, col=cols)
```

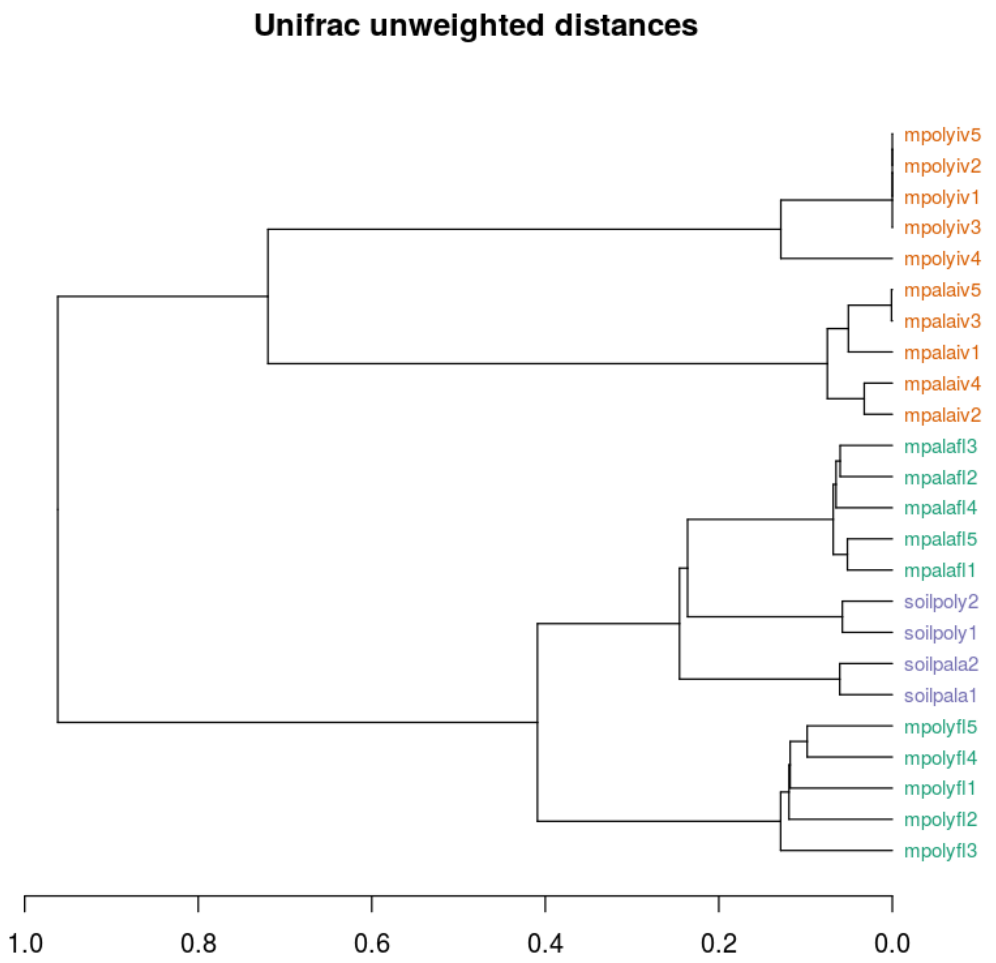
Unifrac unweighted distances
mpolyiv5
mpolyiv2
mpolyiv1
mpolyiv3
mpolyiv4
mpalaiv5
mpalaiv3
mpalaiv1
mpalaiv4
mpalaiv2
mpalafl3
mpalafl2
mpalafl4
mpalafl5
mpalafl1
soilpoly2
soilpoly1
soilpala2
soilpala1
mpolyfl5
mpolyfl4
mpolyfl1
mpolyfl2
mpolyfl3
1.0
0.8
0.6
0.4
0.2
0.0

In [16]:
```
marchantita <- get_variable(march_subset, "habitat")
sample_data(march_subset)$marchantita <- factor(marchantita)
colorCodes <- levels(marchantita)
library(RColorBrewer)
#sample_data(march_subset)
marchUF <- UniFrac(march_subset,weighted=TRUE)
#colorScale <- colors()[c(26,51,76)]
#colorScale    <-rainbow(length(levels(get_variable(march_subset, "marchantita
"))))
colorScale    <- brewer.pal(length(levels(get_variable(march_subset, "marchant
ita"))),"Dark2")
#colorScale
cols <- colorScale[get_variable(march_subset, "marchantita")]
#cols
#march.tip.labels <- as(get_variable(march_subset, "marchantita"), "character"
)
# This is the actual hierarchical clustering call, specifying average-link clu
stering
march.hclust     <- hclust(marchUF, method="average")
library(dendextend)
dend <- as.dendrogram(march.hclust)
pdf("Wunifrac_dendrogram.pdf")
labels_colors(dend) <- cols[order.dendrogram(dend)]
dend %>% set("labels_cex", .75) %>% plot(main="Unifrac weighted distances",hor
iz=T)
dev.off()
#ggplot(dend, horiz=T)
#plot(march.hclust, col=cols)
```

png: 2

In [22]:

```
plist <- vector("list", length(dist_methods))
print(distanceMethodList)
for( i in dist_methods ){
 # Calculate distance matrix
    iDist2 <- distance(march_subset, method=i)
    # Calculate ordination
    iMDS2  <- ordinate(march_subset, "DCA", distance=iDist2)
    ## Make plot
    p <- NULL
    # Don't carry over previous plot (if error, p will be blank)
    # Create plot, store as temp variable, p
    p <- plot_ordination(march_subset, iMDS2, color="habitat", shape="species"
)
    p <- p + ggtitle(paste("DCA using distance method ", i, sep=""))
    plist[[i]] = p
}

df = ldply(plist, function(x) x$data)
names(df)[1] <- "distance"
    #aqui es importante fijarse como se llaman los ejes de las distintas metri
cas para graficar correctamente (DCA, NMDS, Axis.1, etc) puedes verlo con head
(df) para ver los ejes
 p = ggplot(df, aes(DCA1, DCA2, color=habitat, shape=species))
 p = p + geom_point(size=3, alpha=0.5)
 p = p + facet_wrap(~distance, scales="free")
 p = p + ggtitle("DCA on various distance metrics for Marchantia")
 p
    ggsave("DCA_metrics.pdf", width = 40, height = 20, units = "cm")
```

```
$UniFrac
[1] "unifrac"  "wunifrac"

$DPCoA
[1] "dpcoa"

$JSD
[1] "jsd"

$vegdist
 [1] "manhattan"  "euclidean"  "canberra"   "bray"       "kulczynski"
 [6] "jaccard"    "gower"      "altGower"   "morisita"   "horn"
[11] "mountford"  "raup"       "binomial"   "chao"       "cao"

$betadiver
 [1] "w"   "-1"  "c"   "wb"  "r"   "I"   "e"   "t"   "me"  "j"   "sor" "m"
[13] "-2"  "co"  "cc"  "g"   "-3"  "l"   "19"  "hk"  "rlb" "sim" "gl"  "z"

$dist
[1] "maximum"   "binary"    "minkowski"

$designdist
[1] "ANY"
```

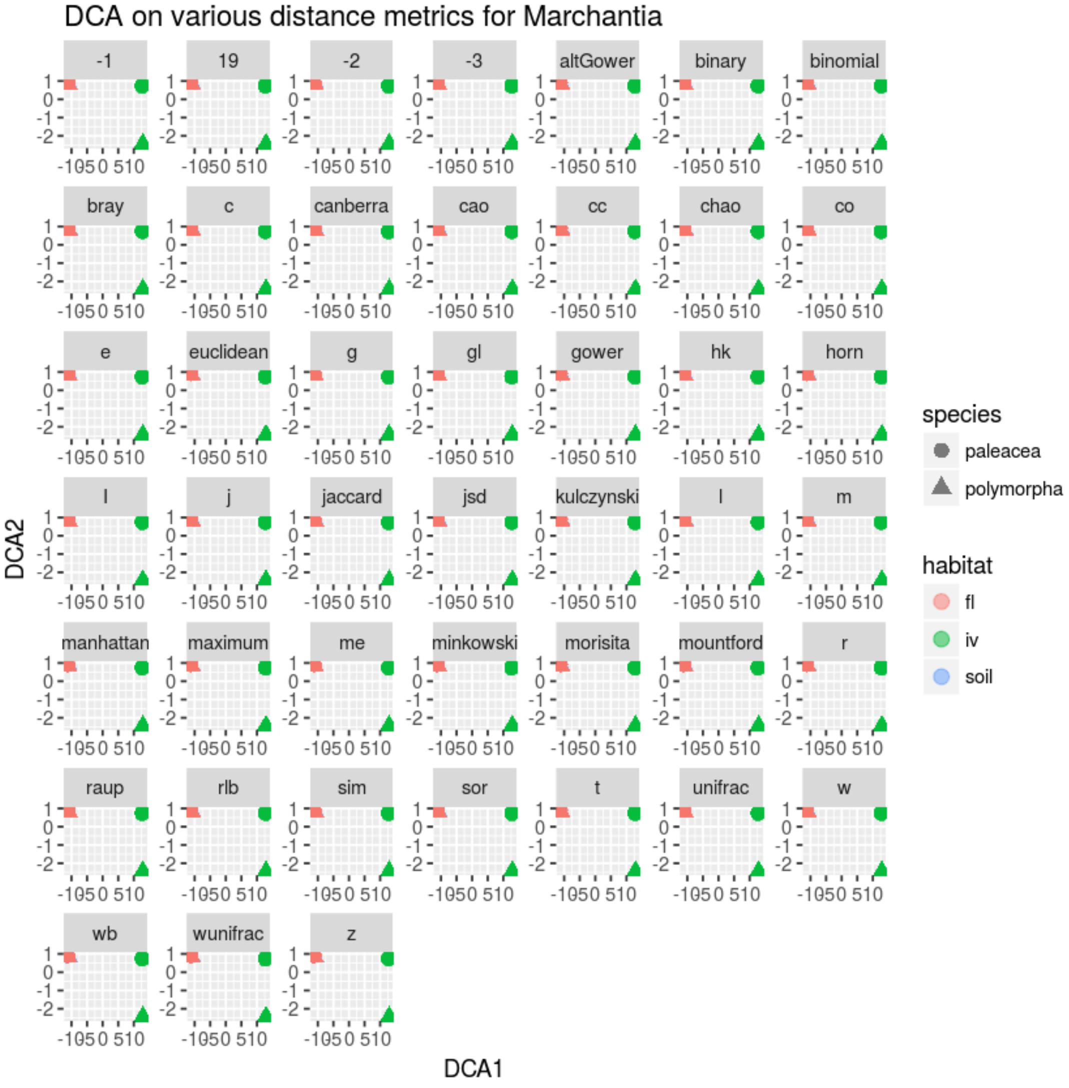
DCA on various distance metrics for Marchantia
-1
19
-2
-3
altGower
binary
binomial
bray
c
canberra
cao
cc
chao
co
e
euclidean
g
gl
gower
hk
horn
l
j
jaccard
jsd
kulczynski
l
m
manhattan
maximum
me
minkowski
morisita
mountford
r
raup
rlb
sim
sor
t
unifrac
w
wb
wunifrac
z
DCA2
DCA1
species
paleacea
polymorpha
habitat
fl
iv
soil

In [23]:
```
plist <- vector("list", length(dist_methods))
print(distanceMethodList)
for( i in dist_methods ){
 # Calculate distance matrix
    iDist2 <- distance(march_subset, method=i)
    # Calculate ordination
    iMDS2  <- ordinate(march_subset, "CCA", distance=iDist2)
    ## Make plot
    p <- NULL
    # Don't carry over previous plot (if error, p will be blank)
    # Create plot, store as temp variable, p
    p <- plot_ordination(march_subset, iMDS2, color="habitat", shape="species"
)
    p <- p + ggtitle(paste("CCA using distance method ", i, sep=""))
    plist[[i]] = p
}

df = ldply(plist, function(x) x$data)
names(df)[1] <- "distance"
    #aqui es importante fijarse como se llaman los ejes de las distintas metri
cas para graficar correctamente (DCA, NMDS, Axis.1, etc) puedes verlo con head
(df) para ver los ejes
 p = ggplot(df, aes(CA1, CA2, color=habitat, shape=species))
 p = p + geom_point(size=3, alpha=0.5)
 p = p + facet_wrap(~distance, scales="free")
 p = p + ggtitle("CCA on various distance metrics for Marchantia")
 p
    ggsave("CCA_metrics.pdf", width = 40, height = 20, units = "cm")
```

```
$UniFrac
[1] "unifrac"  "wunifrac"

$DPCoA
[1] "dpcoa"

$JSD
[1] "jsd"

$vegdist
 [1] "manhattan"  "euclidean"  "canberra"   "bray"       "kulczynski"
 [6] "jaccard"    "gower"      "altGower"   "morisita"   "horn"
[11] "mountford"  "raup"       "binomial"   "chao"       "cao"

$betadiver
 [1] "w"   "-1"  "c"   "wb"  "r"   "I"   "e"   "t"   "me"  "j"   "sor" "m"
[13] "-2"  "co"  "cc"  "g"   "-3"  "l"   "19"  "hk"  "rlb" "sim" "gl"  "z"

$dist
[1] "maximum"   "binary"    "minkowski"

$designdist
[1] "ANY"
```

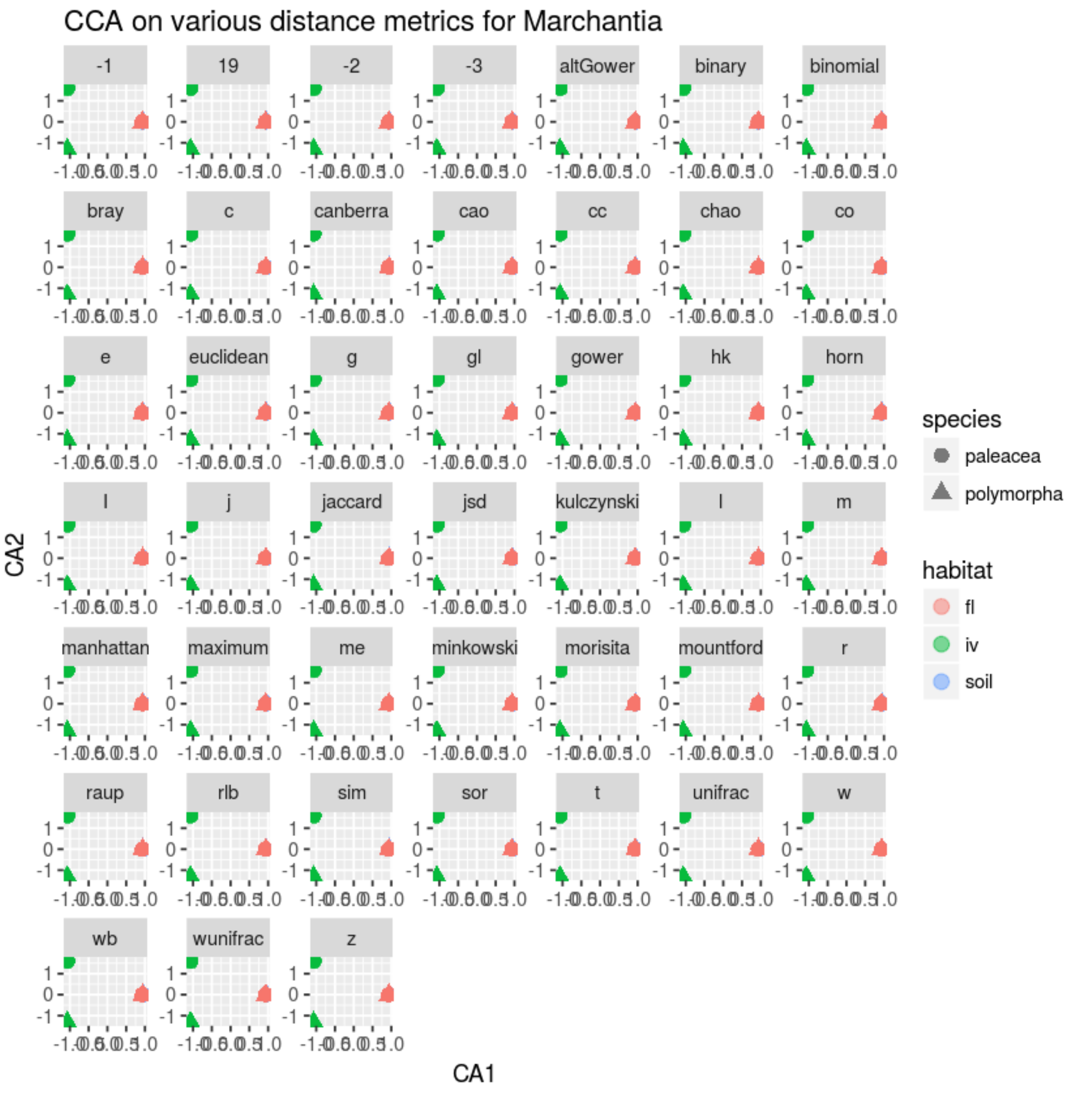
CCA on various distance metrics for Marchantia
-1
19
-2
-3
altGower
binary
binomial
bray
c
canberra
cao
cc
chao
co
e
euclidean
g
gl
gower
hk
horn
l
j
jaccard
jsd
kulczynski
l
m
manhattan
maximum
me
minkowski
morisita
mountford
r
raup
rlb
sim
sor
t
unifrac
w
wb
wunifrac
z
CA2
CA1
species
paleacea
polymorpha
habitat
fl
iv
soil

In [25]:

```
options(warn=-1)


dist = "jaccard"
ord_meths = c("DCA", "CCA", "RDA", "DPCoA", "NMDS", "MDS", "PCoA")
plist = llply(as.list(ord_meths), function(i, march_subset, dist){
        ordi = ordinate(march_subset, method=i, distance=dist)
        plot_ordination(march_subset, ordi, "samples", color="habitat", shape=
"species")
}, march_subset, dist)

names(plist) <- ord_meths
pdataframe = ldply(plist, function(x){
    df = x$data[, 1:2]
    colnames(df) = c("Axis_1", "Axis_2")
    return(cbind(df, x$data))
})
names(pdataframe)[1] = "method"

p = ggplot(pdataframe, aes(Axis_1, Axis_2, color=habitat, shape=species))
p = p + geom_point(size=4)
p = p + facet_wrap(~method, scales="free")
p = p + scale_fill_brewer(type="qual", palette="Set1")
p = p + scale_colour_brewer(type="qual", palette="Set1") + theme_bw()
p = p + ggtitle("Jaccard distance on various Ordinations for Marchantia")
p
 ggsave("Jaccard_distances.pdf", width = 40, height = 20, units = "cm")
```

```
Square root transformation
Wisconsin double standardization
Run 0 stress 9.950987e-05
Run 1 stress 0.00021297
... Procrustes: rmse 0.1087469  max resid 0.1682795
Run 2 stress 7.786858e-05
... New best solution
... Procrustes: rmse 0.004298159  max resid 0.006188049
... Similar to previous best
Run 3 stress 0.0009019083
Run 4 stress 0.0007485717
Run 5 stress 7.459967e-05
... New best solution
... Procrustes: rmse 0.1179356  max resid 0.1827478
Run 6 stress 0.0001952467
... Procrustes: rmse 0.006809374  max resid 0.008003544
Run 7 stress 9.305516e-05
... Procrustes: rmse 0.1004125  max resid 0.1474372
Run 8 stress 9.483684e-05
... Procrustes: rmse 0.02411557  max resid 0.03726638
Run 9 stress 0.001447644
Run 10 stress 0.001257815
Run 11 stress 9.991861e-05
... Procrustes: rmse 0.02498175  max resid 0.03857691
Run 12 stress 0.001218296
Run 13 stress 0.001251119
Run 14 stress 0.0001533029
... Procrustes: rmse 0.01024467  max resid 0.01563828
Run 15 stress 0.1605149
Run 16 stress 0.001074625
Run 17 stress 8.785466e-05
... Procrustes: rmse 0.007145634  max resid 0.01094475
Run 18 stress 9.347364e-05
... Procrustes: rmse 0.0310897  max resid 0.04792954
Run 19 stress 9.401055e-05
... Procrustes: rmse 0.1006968  max resid 0.1477837
Run 20 stress 0.0002110968
... Procrustes: rmse 0.005901496  max resid 0.007953676
*** No convergence -- monoMDS stopping criteria:
     9: no. of iterations >= maxit
     8: stress < smin
     3: scale factor of the gradient < sfgrmin
```

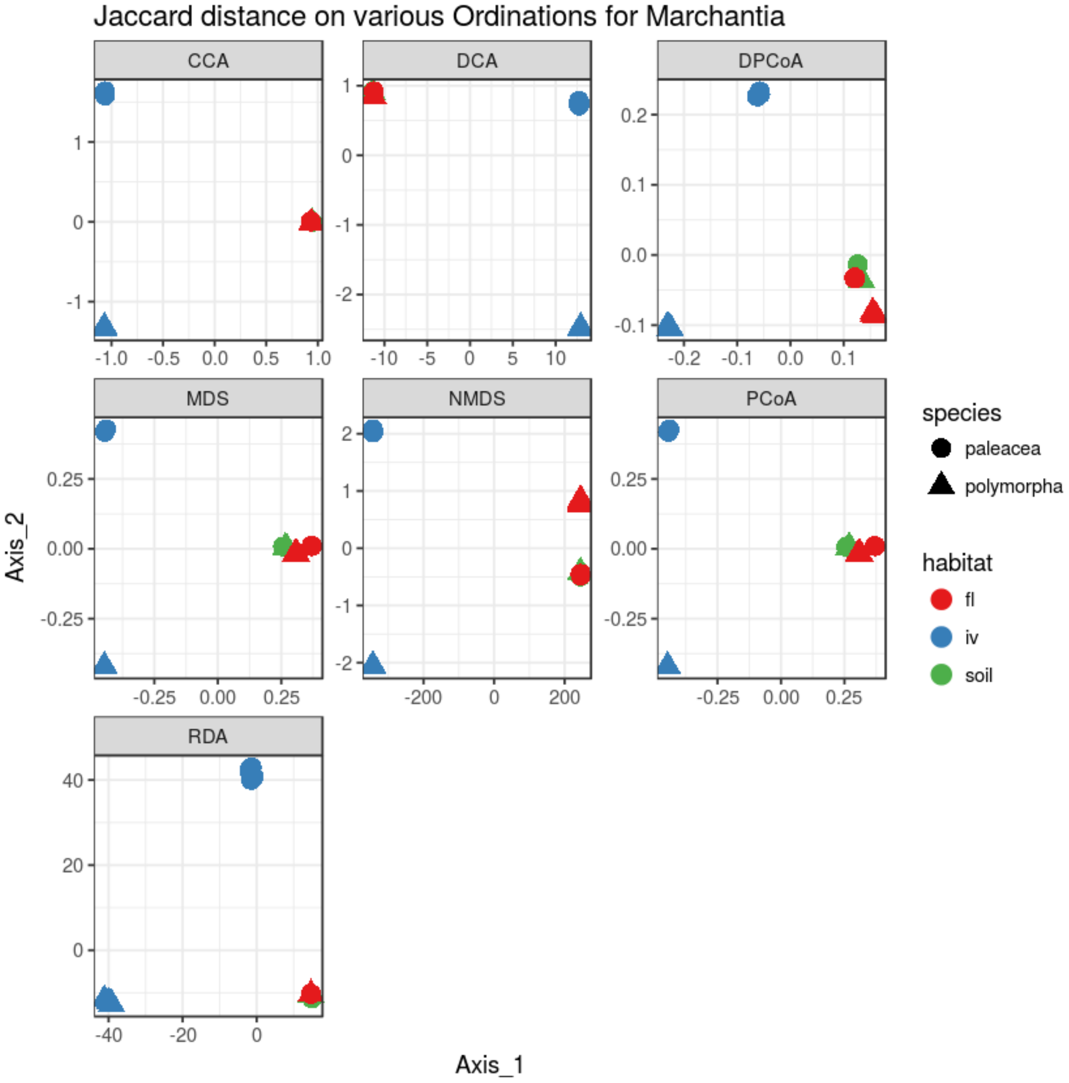
Jaccard distance on various Ordinations for Marchantia
CCA
DCA
DPCoA
MDS
NMDS
PCoA
RDA
Axis_1
Axis_2
species
paleacea
polymorpha
habitat
fl
iv
soil

In [2]:
```
library(phyloseq)
library(ggplot2)
library(RColorBrewer)
otu <- as.matrix(read.table("otu_tablefilter.tab", header=T, row.names=1))
OTU = otu_table(otu, taxa_are_rows=T)
taximat = as.matrix(read.table("otu_tablefilter.tax", header=T, row.names=1))
march = phyloseq (OTU, taxi)
taxi = tax_table(taximat)
march = phyloseq (OTU, taxi)
march_data = read.table("metadata.txt", header=T, row.names=1)
sample_data =sample_data (data.frame(id=march_data$id, species=march_data$spec
ies, family=march_data$family, kind=march_data$kind, row.names=sample_names(ma
rch)))
march = phyloseq(OTU, taxi, sample_data)
march
sample_data(march)
```

```
phyloseq-class experiment-level object
otu_table()   OTU Table:         [ 10314 taxa and 36 samples ]
sample_data() Sample Data:       [ 36 samples by 4 sample variables ]
tax_table()   Taxonomy Table:    [ 10314 taxa by 7 taxonomic ranks ]
```

	id	species	family	kind
**rice2**	rice2	Oriza_sativa	Poaceae	flowering
**rice1**	rice1	Oriza_sativa	Poaceae	flowering
**mpalafl2**	mpalafl2	M_paleacea	Marchantiaceae	liverwort
**mpalafl5**	mpalafl5	M_paleacea	Marchantiaceae	liverwort
**mpalafl4**	mpalafl4	M_paleacea	Marchantiaceae	liverwort
**mpolyfl2**	mpolyfl2	M_polymorpha	Marchantiaceae	liverwort
**mpolyfl3**	mpolyfl3	M_polymorpha	Marchantiaceae	liverwort
**soilpala**	soilpala	M_paleacea	soil	soil
**maize2L01A095**	maize2L01A095	Zea_mays	Poaceae	flowering
**mpolyfl1**	mpolyfl1	M_polymorpha	Marchantiaceae	liverwort
**mpolyfl4**	mpolyfl4	M_polymorpha	Marchantiaceae	liverwort
**mpolyfl5**	mpolyfl5	M_polymorpha	Marchantiaceae	liverwort
**ugt**	ugt	Utricularia_gibba	Lentibulariaceae	flowering
**athsoil1**	athsoil1	Arabidopsis_thaliana	soil	soil
**athrh1**	athrh1	Arabidopsis_thaliana	Brassicaceae	flowering
**athsoil2**	athsoil2	Arabidopsis_thaliana	soil	soil
**athrh2**	athrh2	Arabidopsis_thaliana	Brassicaceae	flowering
**soilpoly**	soilpoly	M_polymorpha	soil	soil
**mpalafl3**	mpalafl3	M_paleacea	Marchantiaceae	liverwort
**mpalafl1**	mpalafl1	M_paleacea	Marchantiaceae	liverwort
**muricataf206**	muricataf206	Pinus_muricata	Pinaceae	conifer
**MossR1**	MossR1	Sphagnum_magellanicum	Sphagnaceae	moss
**muricataf204**	muricataf204	Pinus_muricata	Pinaceae	conifer
**muricataf203**	muricataf203	Pinus_muricata	Pinaceae	conifer
**mpolyiv2**	mpolyiv2	M_polymorpha	Marchantiaceae	liverwort
**mpolyiv3**	mpolyiv3	M_polymorpha	Marchantiaceae	liverwort
**mpolyiv4**	mpolyiv4	M_polymorpha	Marchantiaceae	liverwort
**mpolyiv1**	mpolyiv1	M_polymorpha	Marchantiaceae	liverwort
**mpolyiv5**	mpolyiv5	M_polymorpha	Marchantiaceae	liverwort
**maize1L12A095**	maize1L12A095	Zea_mays	Poaceae	flowering
**mpalaiv2**	mpalaiv2	M_paleacea	Marchantiaceae	liverwort
**mpalaiv4**	mpalaiv4	M_paleacea	Marchantiaceae	liverwort
**mpalaiv3**	mpalaiv3	M_paleacea	Marchantiaceae	liverwort
**mpalaiv5**	mpalaiv5	M_paleacea	Marchantiaceae	liverwort
**mpalaiv1**	mpalaiv1	M_paleacea	Marchantiaceae	liverwort
**muricataf207**	muricataf207	Pinus_muricata	Pinaceae	conifer

In [3]:
```
#de esta forma se quitan las muestras que no queremos.
#samples_to_keep <- c("rice2", "rice1", "mpalafl2", "mpalafl5",
#                    "mpalafl4", "mpolyfl2", "mpolyfl3", "soilpala", "maize2L0
1A095", "mpolyfl1", "mpolyfl4", "mpolyfl5", "ugt", "athsoil1", "athrh1", "aths
oil2", "athrh2", "soilpoly", "mpalafl3", "mpalafl1", "muricataf206", "MossR1",
"muricataf204", "muricataf203", "maize1L12A095", "muricataf207")
samples_to_keep <- c("rice2", "rice1", "mpalafl2", "mpalafl5",
                    "mpalafl4", "mpolyfl2", "mpolyfl3",
                     "maize2L01A095", "mpolyfl1", "mpolyfl4", "mpolyfl5", "ugt
", "athrh1",
                     "athrh2", "mpalafl3", "mpalafl1", "muricataf206",
                     "MossR1", "muricataf204", "muricataf203", "maize1L12A095"
, "muricataf207")

march_subset <- prune_samples(samples_to_keep, march)
march_subset
```

```
phyloseq-class experiment-level object
otu_table()   OTU Table:         [ 10314 taxa and 22 samples ]
sample_data() Sample Data:       [ 22 samples by 4 sample variables ]
tax_table()   Taxonomy Table:    [ 10314 taxa by 7 taxonomic ranks ]
```

In [4]:
```
march_ordCAP.ORD <- ordinate(march_subset, "CAP", "bray", ~ species + family )

cap_plot <- plot_ordination(march_subset, march_ordCAP.ORD, color="species", a
xes =c(1,2))+
aes(shape= family) + geom_point(aes(colour = species), alpha=0.4, size=4)  + t
heme_bw()

arrowmat <- vegan::scores(march_ordCAP.ORD, display="bp")
arrowdf <- data.frame(labels=rownames(arrowmat), arrowmat)

# Define the arrow aesthetic mapping
arrow_map <- aes(xend = CAP1,
    yend = CAP2,
    x = 0,
    y = 0,
    shape = NULL,
    color = NULL,
    label = NULL)
#se puede poner labels en este último campo y se ponen las etiquetas a las fle
chas...
label_map <- aes(x = 1.3 * CAP1,
    y = 1.3 * CAP2,
    shape = NULL,
    color = NULL,
    label = labels)

arrowhead = arrow(length = unit(0.02, "npc"))

cap_plot  +
  geom_segment(
    mapping = arrow_map,
    size = .5,
    data = arrowdf,
    color = "gray",
    arrow = arrowhead
  ) +
 geom_text(
    mapping = label_map,
    size = 2,
    data = arrowdf,
    show.legend = FALSE
  )
#quitar esta última sección para quitar las etiquetas
anova(march_ordCAP.ORD)
ggsave("CAP_Bray_marchantia_comparative.pdf", width = 20, height = 20, units =
"cm")
```

```
Warning message:
“Ignoring unknown aesthetics: shape, label”Warning message:
“Ignoring unknown aesthetics: shape”
```

	Df	SumOfSqs	F	Pr(>F)
**Model**	7	6.890243	8.884718	0.001
**Residual**	14	1.551032	NA	NA

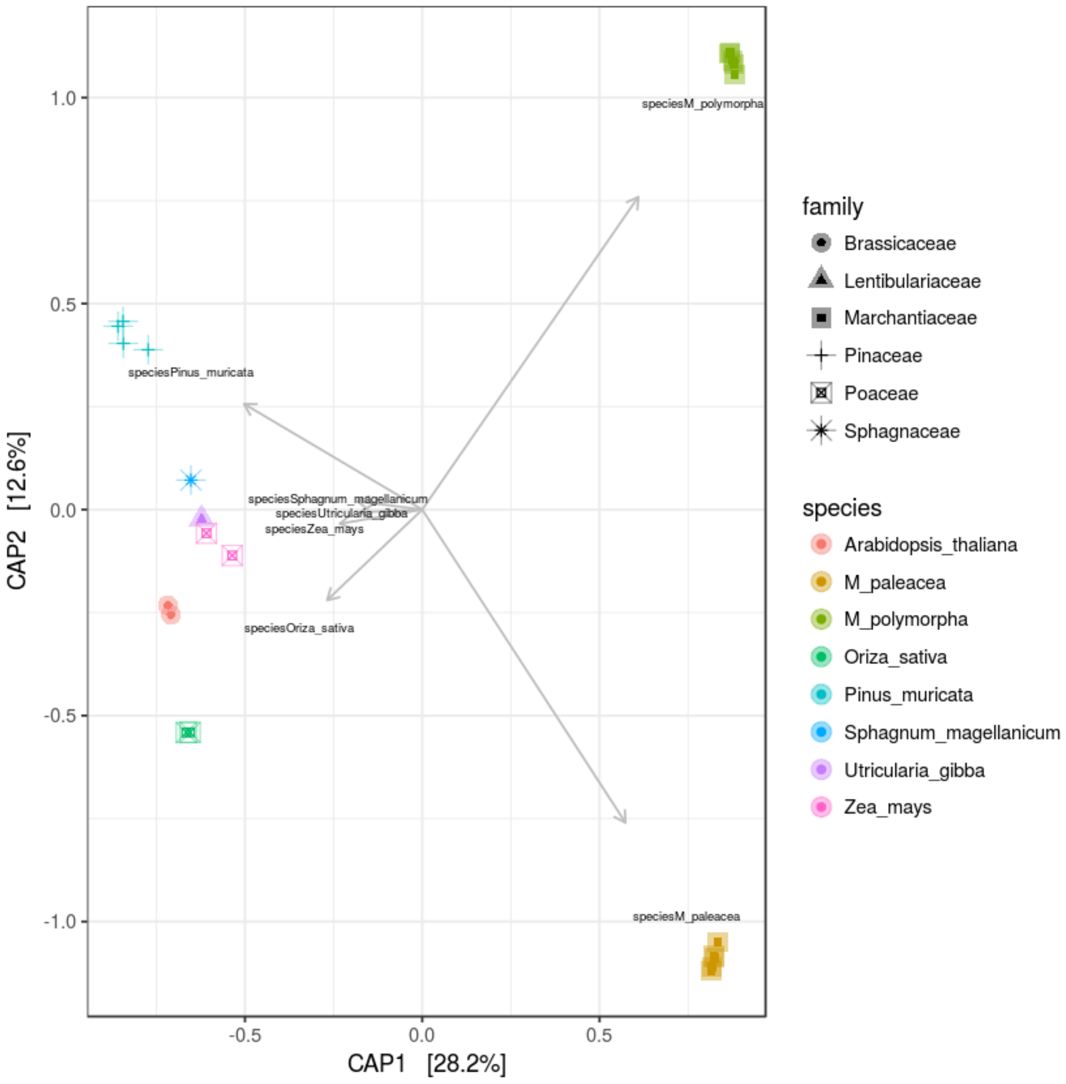

In [4]:
```
options(warn=-1)
library(ape)
library(plyr)
dist_methods <- unlist(distanceMethodList)

#Remove the two distance-methods that require a tree, and the generic custom m
ethod that requires user-defined distance arguments.
# These require tree
dist_methods[(1:3)]
# Remove them from the vector
dist_methods <- dist_methods[-(1:3)]
# This is the user-defined method:
dist_methods["designdist"]
# Remove the user-defined distance
dist_methods = dist_methods[-which(dist_methods=="ANY")]

#NMDS



plist <- vector("list", length(dist_methods))
print(distanceMethodList)

for( i in dist_methods ){
 # Calculate distance matrix
    iDist <- distance(march_subset, method=i)
    # Calculate ordination
    iMDS  <- ordinate(march_subset, "NMDS", distance=iDist)
    ## Make plot
    p <- NULL
    # Don't carry over previous plot (if error, p will be blank)
    # Create plot, store as temp variable, p
    p <- plot_ordination(march_subset, iMDS, color="species", shape="family")
    p <- p + ggtitle(paste("NMDS using distance method ", i, sep=""))
    plist[[i]] = p
}

df = ldply(plist, function(x) x$data)
names(df)[1] <- "distance"
    #aqui es importante fijarse como se llaman los ejes de las distintas metri
cas para graficar correctamente (DCA, NMDS, Axis.1, etc) puedes verlo con head
(df) para ver los ejes

 p = ggplot(df, aes(NMDS1, NMDS2, color=species, shape=family))
 p = p + geom_point(size=3, alpha=0.5)
 p = p + facet_wrap(~distance, scales="free")
 p = p + ggtitle("NMDS on various distance metrics for Marchantia")
 p
```

**UniFrac1**	'unifrac'
**UniFrac2**	'wunifrac'
**DPCoA**	'dpcoa'

designdist: 'ANY'

```
$UniFrac
[1] "unifrac"  "wunifrac"

$DPCoA
[1] "dpcoa"

$JSD
[1] "jsd"

$vegdist
 [1] "manhattan"  "euclidean"  "canberra"   "bray"       "kulczynski"
 [6] "jaccard"    "gower"      "altGower"   "morisita"   "horn"
[11] "mountford"  "raup"       "binomial"   "chao"       "cao"

$betadiver
 [1] "w"   "-1"  "c"   "wb"  "r"   "I"   "e"   "t"   "me"  "j"   "sor" "m"
[13] "-2"  "co"  "cc"  "g"   "-3"  "l"   "19"  "hk"  "rlb" "sim" "gl"  "z"

$dist
[1] "maximum"   "binary"    "minkowski"

$designdist
[1] "ANY"

Run 0 stress 0.1257919
Run 1 stress 0.1596213
Run 2 stress 0.1137733
... New best solution
... Procrustes: rmse 0.1168675  max resid 0.277071
Run 3 stress 0.1137703
... New best solution
... Procrustes: rmse 0.0007195382  max resid 0.002833795
... Similar to previous best
Run 4 stress 0.1274771
Run 5 stress 0.1137734
... Procrustes: rmse 0.0007328831  max resid 0.002887553
... Similar to previous best
Run 6 stress 0.1137767
... Procrustes: rmse 0.001259233  max resid 0.004952696
... Similar to previous best
Run 7 stress 0.1137692
... New best solution
... Procrustes: rmse 0.0002244034  max resid 0.0008425625
... Similar to previous best
Run 8 stress 0.1213348
Run 9 stress 0.131903
Run 10 stress 0.1137732
... Procrustes: rmse 0.000901024  max resid 0.003570791
... Similar to previous best
Run 11 stress 0.1137708
... Procrustes: rmse 0.0004303656  max resid 0.001705972
... Similar to previous best
Run 12 stress 0.1411755
Run 13 stress 0.1213349
Run 14 stress 0.1213347
Run 15 stress 0.141175
Run 16 stress 0.125792
Run 17 stress 0.1226701
Run 18 stress 0.1137714
... Procrustes: rmse 0.0005620939  max resid 0.002228979
... Similar to previous best
Run 19 stress 0.1348242
Run 20 stress 0.1226699
```

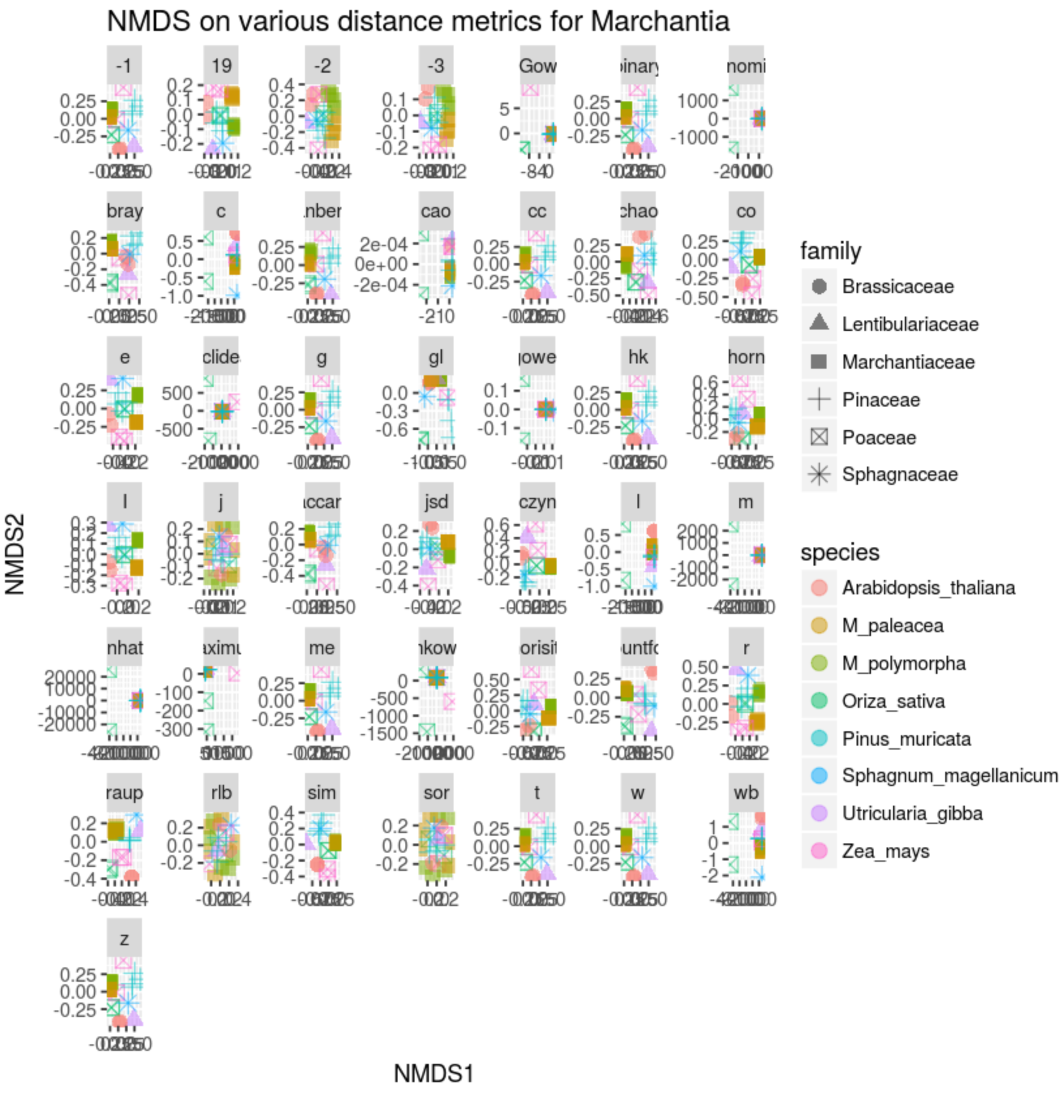

In [23]:
```
ggsave("nmds_grandote.pdf", p, width = 40, height = 20, units = "cm")
```

iMDS <- ordinate(march_subset, "NMDS", distance="jaccard")

```
## Make plot
q <- NULL
# Don't carry over previous plot (if error, p will be blank)
# Create plot, store as temp variable, p
q <- plot_ordination(march_subset, iMDS, color="species", shape="family")
```

q = q + geom_point(size=3, alpha=0.5) + theme_bw() q

In [ ]:

```
library(phyloseq)
library(ggplot2)
library(RColorBrewer)
otu <- as.matrix(read.table("otu_tablefilter.tab", header=T, row.names=1)
)
otu
OTU = otu_table(otu, taxa_are_rows=T)
taximat = as.matrix(read.table("otu_tablefilter.tax", header=T, row.names=1))
taximat = as.matrix(read.table("otu_tablefilter.tax", header=T, row.names=1))
taximat
march = phyloseq (OTU, taxi)
taxi = tax_table(taximat)
march = phyloseq (OTU, taxi)
march_data = as.matrix(read.table("metadata.txt", header=T, row.names=1==
))
march_data = as.matrix(read.table("metadata.txt", header=T, row.names=1))
march_data
march_data = read.table("metadata.txt", header=T, row.names=1))
march_data = read.table("metadata.txt", header=T, row.names=1)
sample_data (data.frame(species=march_data$species, family=march_data$family,
kind=march_data$kind, row.names=sample_names(march)))
sample_data =sample_data (data.frame(species=march_data$species, family=march_
data$family, kind=march_data$kind, row.names=sample_names(march)))
march = phyloseq(OTU, taxi, sampledata)
march = phyloseq(OTU, taxi, sample_data)
march
plot_richness(march)
p = plot_richness(march)
ggsave("plot_richnes.pdf", p)
pdf("plot_richness.pdf")
p
dev.off()
p$data
sample_data$species
pdf("plot_richness.pdf")
plot_richness(march, measures =c("Shannon", "Simpson", "Chao1", "Observed"), c
olor="species")
dev.off()
savehistory()
save()
save.image()
march1 = merge_samples(march, "species")
sample_data(march1)$species <- factor(sample_names(march1))
pdf("plot_richness.pdf")
plot_richness(march1, x="species",  measures =c("Shannon", "Simpson", "Chao1",
"Observed"), color="species")
dev.off()
q()
plot_richness(march1, x="species",  measures =c("Shannon", "Simpson", "Chao1",
"Observed"), color="species")
library(phyloseq)
library(ggplot2)
plot_richness(march1, x="species",  measures =c("Shannon", "Simpson", "Chao1",
"Observed"), color="species")
rm (march)
march_data = as.matrix(read.table("metadata.txt", header=T, row.names=1))
march = phyloseq(OTU, taxi)
sample_data =sample_data (data.frame(species=march_data$species, family=march_
da
ta$family, kind=march_data$kind, id=march_data$id, row.names=sample_names(marc
h)))
sample_data =sample_data (data.frame(species=march_data$species, family=march_
data$family, kind=march_data$kind, id=march_data$id, row.names=sample_names(ma
```

Names	total	elements
mpalafl mpalaiv mpolyfl mpolyiv soils	5	otu_147611 otu_8969 otu_150011 otu_2102 otu_150024
mpalafl mpalaiv mpolyfl soils	7	otu_33060 otu_26997 otu_2729 otu_16934 otu_3800 otu_1472 otu_1686
mpalafl mpolyfl mpolyiv soils	19	otu_8406 otu_2372 otu_147708 otu_7759 otu_25032 otu_147667 otu_139675 otu_150010<br>otu_147680 otu_147463 otu_145010 otu_147610 otu_147482 otu_147450 otu_8998<br>otu_142926 otu_145856 otu_146708 otu_22443
mpalafl mpolyfl soils	3072	otu_7825 otu_103278 otu_82423 otu_96699 otu_80867 otu_83670 otu_149670<br>otu_150857 otu_143862 otu_6470 otu_825 otu_104395 otu_145004 otu_144261 otu_307<br>otu_147022 otu_2632 otu_153328 otu_150406 otu_264 otu_112119 otu_150849<br>otu_106386 otu_27079 otu_146777 otu_83650 otu_108759 otu_23422 otu_152783<br>otu_22534 otu_26188 otu_119751 otu_42983 otu_150091 otu_27574 otu_148806<br>otu_106154 otu_2388 otu_145341 otu_138151 otu_147607 otu_151842 otu_104754<br>otu_139227 otu_5349 otu_105231 otu_105577 otu_140750 otu_38321 otu_128385<br>otu_154518 otu_4025 otu_147542 otu_143383 otu_104204 otu_28591 otu_150065<br>otu_3654 otu_114068 otu_106549 otu_27123 otu_153567 otu_150506 otu_100234<br>otu_81392 otu_104442 otu_81723 otu_14405 otu_144723 otu_137645 otu_33392<br>otu_126285 otu_1000 otu_147591 otu_105474 otu_6092 otu_23811 otu_103296<br>otu_78989 otu_105745 otu_146591 otu_106782 otu_133199 otu_2475 otu_147623<br>otu_104161 otu_141953 otu_97272 otu_105652 otu_103730 otu_150280 otu_126208<br>otu_152105 otu_35063 otu_104533 otu_106030 otu_25584 otu_103710 otu_103380<br>otu_98414 otu_103605 otu_26892 otu_104016 otu_106590 otu_58544 otu_132503<br>otu_139518 otu_144101 otu_150272 otu_28008 otu_144341 otu_64419 otu_147007<br>otu_144124 otu_139524 otu_149990 otu_142127 otu_19728 otu_18830 otu_22948<br>otu_149791 otu_25204 otu_146308 otu_106108 otu_99242 otu_7563 otu_7457 otu_7907<br>otu_103085 otu_19514 otu_138137 otu_107097 otu_105750 otu_25374 otu_4393<br>otu_110719 otu_59639 otu_22486 otu_22322 otu_81337 otu_26184 otu_106622<br>otu_140979 otu_147585 otu_19649 otu_146935 otu_103800 otu_100173 otu_926<br>otu_38578 otu_79195 otu_148212 otu_146311 otu_29613 otu_8187 otu_28633<br>otu_103691 otu_127853 otu_147844 otu_2505 otu_22818 otu_17931 otu_2899<br>otu_136526 otu_24659 otu_33212 otu_139711 otu_26825 otu_108087 otu_127400<br>otu_88420 otu_3344 otu_8457 otu_103108 otu_145 otu_3578 otu_138588 otu_104271<br>otu_126288 otu_140431 otu_151407 otu_151109 otu_99439 otu_146214 otu_127578<br>otu_127162 otu_127566 otu_36110 otu_104103 otu_139690 otu_103707 otu_147887<br>otu_136493 otu_103374 otu_134432 otu_27017 otu_103543 otu_24738 otu_147659<br>otu_1315 otu_24255 otu_16512 otu_13196 otu_6395 otu_29185 otu_23184 otu_146845<br>otu_5404 otu_932 otu_143828 otu_147646 otu_400 otu_150704 otu_7734 otu_24328<br>otu_7851 otu_79566 otu_951 otu_757 otu_103497 otu_25823 otu_8526 otu_107606<br>otu_151361 otu_2311 otu_6352 otu_79871 otu_25952 otu_24800 otu_5120 otu_102893<br>otu_79441 otu_103161 otu_104759 otu_25643 otu_79883 otu_103689 otu_98984<br>otu_5567 otu_141256 otu_944 otu_4421 otu_99299 otu_103885 otu_105066 otu_7849<br>otu_142374 otu_138492 otu_112898 otu_126704 otu_99543 otu_132630 otu_80496<br>otu_127127 otu_17929 otu_147357 otu_3757 otu_2794 otu_143785 otu_132507<br>otu_153094 otu_103585 otu_25290 otu_110104 otu_26010 otu_146574 otu_116904<br>otu_79323 otu_149309 otu_99676 otu_127070 otu_102545 otu_103967 otu_150755<br>otu_24104 otu_144623 otu_110594 otu_107065 otu_7693 otu_141080 otu_104314<br>otu_103313 otu_26618 otu_99993 otu_103935 otu_105507 otu_103877 otu_132898<br>otu_126213 otu_137809 otu_80506 otu_152253 otu_151273 otu_117646 otu_104379<br>otu_144500 otu_7702 otu_23490 otu_150084 otu_25042 otu_107205 otu_147080<br>otu_144287 otu_519 otu_105580 otu_25130 otu_7817 otu_150142 otu_24777 otu_132542<br>otu_137274 otu_103968 otu_61976 otu_107492 otu_128276 otu_28865 otu_79262<br>otu_3959 otu_110171 otu_103542 otu_17038 otu_18914 otu_120351 otu_3854 otu_7612<br>otu_153417 otu_8428 otu_102810 otu_38503 otu_106428 otu_103483 otu_24221<br>otu_36946 otu_103441 otu_146204 otu_3577 otu_141929 otu_38084 otu_154648<br>otu_6440 otu_149898 otu_29052 otu_108696 otu_106005 otu_147924 otu_117872<br>otu_112442 otu_153475 otu_147750 otu_8813 otu_588 otu_79015 otu_36114 otu_4<br>otu_19508 otu_133759 otu_20 otu_27916 otu_5339 otu_24210 otu_11252 otu_147714<br>otu_9109 otu_147453 otu_134590 otu_143898 otu_150245 otu_147176 otu_3611<br>otu_23158 otu_106542 otu_22871 otu_103117 otu_79234 otu_99472 otu_145505<br>otu_82002 otu_10691 otu_113938 otu_126305 otu_7176 otu_139207 otu_103780<br>otu_24933 otu_146751 otu_136081 otu_80358 otu_26803 otu_6134 otu_3899 otu_102992<br>otu_107588 otu_103684 otu_15181 otu_151081 otu_103850 otu_113580 otu_104123<br>otu_137950 otu_142587 otu_152312 otu_146364 otu_138489 otu_106761 otu_137178<br>otu_126254 otu_31738 otu_150295 otu_146277 otu_147200 otu_104714 otu_3185<br>otu_1169 otu_2671 otu_133988 otu_141889 otu_78987 otu_151142 otu_29849 otu_140981<br>otu_51998 otu_4621 otu_147671 otu_99322 otu_79174 otu_32282 otu_150471<br>otu_143966 otu_96857 otu_4542 otu_3094 otu_948 otu_105570 otu_109819 otu_1946<br>otu_5729 otu_4251 otu_22678 otu_98437 otu_25616 otu_105576 otu_106999 otu_4617<br>otu_146470 otu_150977 otu_152083 otu_9533 otu_81552 otu_104091 otu_105762<br>otu_146284 otu_146309 otu_99371 otu_105738 otu_29496 otu_107797 otu_24239<br>otu_107629 otu_150605 otu_22631 otu_150910 otu_150198 otu_107803 otu_24090<br>otu_104195 otu_80621 otu_99298 otu_79850 otu_8470 otu_3462 otu_16344 otu_143996<br>otu_112521 otu_141180 otu_107183 otu_105539 otu_117551 otu_110850 otu_8086<br>otu_105562 otu_743 otu_153470 otu_7799 otu_151422 otu_105803 otu_2771 otu_34511<br>otu_2846 otu_8390 otu_102862 otu_106342 otu_147723 otu_14239 otu_110483<br>otu_127568 otu_84828 otu_28660 otu_33482 otu_24477 otu_109601 otu_62559 otu_4002<br>otu_6996 otu_317 otu_106841 otu_153982 otu_82908 otu_103461 otu_103334<br>otu_105900 otu_152 otu_103014 otu_144681 otu_34003 otu_1807 otu_104463 otu_25409<br>otu_147245 otu_79360 otu_2302 otu_138831 otu_144811 otu_103297 otu_79286<br>otu_4275 otu_110026 otu_127446 otu_97296 otu_3376 otu_147565 otu_109482<br>otu_107410 otu_105930 otu_5604 otu_23925 otu_146215 otu_126169 otu_146636<br>otu_33453 otu_2436 otu_8386 otu_145378 otu_150129 otu_138562 otu_9254 otu_127763<br>otu_126585 otu_134436 otu_28076 otu_52266 otu_151329 otu_23772 otu_96779<br>otu_145036 otu_24124 otu_39592 otu_23128 otu_150105 otu_150488 otu_6629<br>otu_82110 otu_105627 otu_22535 otu_4165 otu_142173 otu_123335 otu_126299<br>otu_150309 otu_30927 otu_46926 otu_105199 otu_147832 otu_131474 otu_106738<br>otu_142043 otu_24257 otu_105649 otu_105561 otu_23453 otu_148448 otu_127429<br>otu_37147 otu_147081 otu_8311 otu_5829 otu_146283 otu_107254 otu_112658<br>otu_128021 otu_36341 otu_146815 otu_27636 otu_79265 otu_30072 otu_154668<br>otu_23271 otu_5652 otu_147631 otu_84576 otu_139353 otu_140074 otu_32346<br>otu_151216 otu_146670 otu_105387 otu_53165 otu_23143 otu_29666 otu_106432<br>otu_82916 otu_9266 otu_99605 otu_29112 otu_146498 otu_104680 otu_150823<br>otu_149719 otu_2840 otu_32909 otu_5711 otu_103286 otu_151678 otu_148122<br>otu_150307 otu_103959 otu_29148 otu_144745 otu_38377 otu_151909 otu_150775<br>otu_30209 otu_103144 otu_5756 otu_116095 otu_104021 otu_151414 otu_146378<br>otu_80393 otu_153271 otu_4693 otu_31150 otu_152800 otu_146677 otu_144675<br>otu_97744 otu_29169 otu_1009 otu_150133 otu_23573 otu_81058 otu_102950<br>otu_132625 otu_103985 otu_18020 otu_149226 otu_58781 otu_142950 otu_3105<br>otu_2281 otu_19433 otu_21095 otu_27999 otu_39164 otu_2431 otu_151357 otu_4158<br>otu_150311 otu_105139 otu_149696 otu_531 otu_39188 otu_3097 otu_146224<br>otu_102819 otu_29125 otu_37042 otu_146249 otu_105986 otu_82519 otu_132472<br>otu_150360 otu_150252 otu_150169 otu_150596 otu_23700 otu_930 otu_103834<br>otu_97020 otu_151538 otu_137057 otu_3910 otu_153196 otu_23215 otu_99224<br>[illegible]

otu_149719 otu_2840 otu_32909 otu_5711 otu_103286 otu_151678 otu_148122
otu_150307 otu_103959 otu_29148 otu_144745 otu_38377 otu_151909 otu_150775
otu_30209 otu_103144 otu_5756 otu_116095 otu_104021 otu_151414 otu_146378
otu_80393 otu_153271 otu_4693 otu_31150 otu_152800 otu_146677 otu_144675
otu_97744 otu_29169 otu_1009 otu_150133 otu_23573 otu_81058 otu_102950
otu_132625 otu_103985 otu_18020 otu_149226 otu_58781 otu_142950 otu_3105
otu_2281 otu_19433 otu_21095 otu_27999 otu_39164 otu_2431 otu_151357 otu_4158
otu_150311 otu_105139 otu_149696 otu_531 otu_39188 otu_3097 otu_146224
otu_102819 otu_29125 otu_37042 otu_146249 otu_105986 otu_82519 otu_132472
otu_150360 otu_150252 otu_150169 otu_150596 otu_23700 otu_930 otu_103834
otu_97020 otu_151538 otu_137057 otu_3910 otu_153196 otu_23215 otu_99224
otu_127254 otu_79594 otu_105497 otu_1013 otu_79514 otu_151452 otu_126619
otu_147736 otu_36515 otu_566 otu_813 otu_105534 otu_11774 otu_150329 otu_126234
otu_38922 otu_152388 otu_79283 otu_113638 otu_99830 otu_8067 otu_754 otu_79370
otu_4624 otu_103132 otu_7071 otu_3871 otu_163455 otu_2597 otu_153520 otu_22355
otu_103952 otu_53436 otu_5744 otu_144632 otu_18496 otu_150323 otu_80201
otu_25426 otu_146721 otu_16617 otu_138189 otu_28016 otu_980 otu_136230
otu_145135 otu_28289 otu_147628 otu_137139 otu_98659 otu_27430 otu_103735
otu_102875 otu_5237 otu_145072 otu_538 otu_149495 otu_97348 otu_84671 otu_145421
otu_148171 otu_3597 otu_139593 otu_147399 otu_127486 otu_100248 otu_5609
otu_147268 otu_921 otu_27931 otu_104910 otu_97120 otu_8443 otu_104638 otu_146291
otu_10976 otu_31451 otu_5623 otu_106698 otu_101 otu_147773 otu_139164 otu_99405
otu_649 otu_110809 otu_36493 otu_23013 otu_109570 otu_143386 otu_96943
otu_102969 otu_149518 otu_103590 otu_23233 otu_104756 otu_102879 otu_139793
otu_150561 otu_105116 otu_144368 otu_153859 otu_32322 otu_107494 otu_150948
otu_150773 otu_140809 otu_106685 otu_148772 otu_107213 otu_45049 otu_103142
otu_151716 otu_147531 otu_146621 otu_7491 otu_103671 otu_136262 otu_5634
otu_103612 otu_31366 otu_105677 otu_25286 otu_127615 otu_147248 otu_29675
otu_22913 otu_28755 otu_99688 otu_106833 otu_141904 otu_138504 otu_102881
otu_109609 otu_141039 otu_153185 otu_21835 otu_7513 otu_2915 otu_27195
otu_146802 otu_147702 otu_12129 otu_147086 otu_24226 otu_23424 otu_104122
otu_141063 otu_104760 otu_103685 otu_152913 otu_5553 otu_24194 otu_137100
otu_154090 otu_103776 otu_141911 otu_103652 otu_27985 otu_36078 otu_19925
otu_27886 otu_99561 otu_106407 otu_105159 otu_150238 otu_96871 otu_7545
otu_146296 otu_101831 otu_25220 otu_2683 otu_147395 otu_133589 otu_25885
otu_3825 otu_104029 otu_5952 otu_110557 otu_107070 otu_106674 otu_150699
otu_18048 otu_145343 otu_148916 otu_3280 otu_147430 otu_54741 otu_150158
otu_27186 otu_7559 otu_130293 otu_8502 otu_22690 otu_25353 otu_133327 otu_147373
otu_151502 otu_138667 otu_79856 otu_1143 otu_147169 otu_99438 otu_118052
otu_126298 otu_103077 otu_79708 otu_8524 otu_114786 otu_79756 otu_103644
otu_106448 otu_146806 otu_29336 otu_107421 otu_106887 otu_103809 otu_80007
otu_99423 otu_96837 otu_104213 otu_26234 otu_147640 otu_140955 otu_100716
otu_5974 otu_99584 otu_103832 otu_42586 otu_145605 otu_664 otu_99636 otu_153608
otu_17656 otu_5627 otu_106250 otu_152827 otu_6515 otu_151541 otu_23242
otu_144300 otu_147239 otu_33110 otu_102890 otu_103478 otu_7060 otu_150325
otu_81339 otu_108077 otu_106753 otu_6963 otu_79002 otu_29397 otu_102816
otu_79824 otu_1910 otu_39427 otu_17506 otu_25310 otu_7913 otu_26381 otu_106081
otu_146528 otu_103143 otu_99557 otu_103175 otu_163295 otu_1254 otu_22514
otu_24114 otu_140887 otu_6994 otu_25119 otu_132815 otu_5926 otu_146982 otu_104080
otu_147150 otu_22906 otu_105185 otu_42615 otu_150094 otu_23405 otu_102973
otu_151118 otu_149197 otu_127394 otu_23874 otu_142346 otu_1832 otu_6312
otu_151229 otu_153393 otu_142022 otu_103110 otu_103657 otu_149640 otu_138414
otu_24681 otu_138239 otu_16374 otu_150140 otu_45497 otu_102796 otu_146558
otu_139808 otu_3528 otu_151243 otu_142060 otu_80779 otu_105088 otu_2275
otu_138643 otu_570 otu_25185 otu_1046 otu_7539 otu_23301 otu_96917 otu_142315
otu_103736 otu_147014 otu_150881 otu_37420 otu_96840 otu_26727 otu_107759
otu_1103 otu_2710 otu_25657 otu_146835 otu_79942 otu_35490 otu_3513 otu_8027
otu_25947 otu_103285 otu_99475 otu_1256 otu_96770 otu_138135 otu_141141
otu_146610 otu_7294 otu_37613 otu_6830 otu_147304 otu_139517 otu_106377
otu_150350 otu_141396 otu_97266 otu_144767 otu_146696 otu_108218 otu_103206
otu_3442 otu_150879 otu_147569 otu_34033 otu_33379 otu_2485 otu_29523 otu_6464
otu_8868 otu_1010 otu_132529 otu_81155 otu_26993 otu_103147 otu_150450 otu_35981
otu_22701 otu_149010 otu_2881 otu_29961 otu_81771 otu_103888 otu_146571
otu_146607 otu_24064 otu_107203 otu_148102 otu_104603 otu_147195 otu_82384
otu_146780 otu_100245 otu_29818 otu_132464 otu_21225 otu_24672 otu_131235
otu_139557 otu_102857 otu_99063 otu_104428 otu_100236 otu_99549 otu_150290
otu_109218 otu_99609 otu_26829 otu_24788 otu_114052 otu_45090 otu_149461
otu_80550 otu_152749 otu_103372 otu_16450 otu_147685 otu_24010 otu_2809
otu_105587 otu_146313 otu_146445 otu_955 otu_103394 otu_23827 otu_3630 otu_2177
otu_29377 otu_151042 otu_144607 otu_103750 otu_8762 otu_103529 otu_23908
otu_146750 otu_148558 otu_9349 otu_5560 otu_138020 otu_97326 otu_4048 otu_147461
otu_81193 otu_16444 otu_147608 otu_128072 otu_104776 otu_5251 otu_5900
otu_148859 otu_2820 otu_38223 otu_17530 otu_26984 otu_128401 otu_22637 otu_22555
otu_147815 otu_32300 otu_152032 otu_102846 otu_80459 otu_7952 otu_103105
otu_6429 otu_106875 otu_105605 otu_147543 otu_23590 otu_22633 otu_22562
otu_139661 otu_80961 otu_110088 otu_146460 otu_147488 otu_138145 otu_104892
otu_103833 otu_103894 otu_100178 otu_22467 otu_149530 otu_1992 otu_102917
otu_8019 otu_3686 otu_150544 otu_150533 otu_147855 otu_105564 otu_150130
otu_28171 otu_984 otu_138421 otu_1773 otu_23691 otu_108800 otu_146486 otu_107237
otu_144616 otu_97424 otu_147588 otu_104530 otu_146850 otu_146603 otu_59364
otu_22648 otu_79344 otu_24990 otu_141019 otu_146417 otu_96870 otu_109062
otu_132531 otu_140767 otu_147202 otu_3595 otu_151498 otu_81796 otu_105125
otu_106514 otu_102944 otu_26632 otu_28735 otu_58009 otu_97848 otu_103679
otu_3476 otu_8602 otu_79099 otu_106121 otu_150569 otu_147877 otu_33503
otu_147078 otu_4958 otu_4415 otu_99645 otu_99397 otu_5688 otu_136798 otu_4157
otu_20665 otu_23724 otu_63159 otu_150376 otu_126341 otu_104519 otu_4486
otu_79329 otu_114101 otu_28988 otu_105666 otu_12686 otu_108172 otu_126853
otu_103613 otu_5841 otu_61915 otu_103636 otu_5807 otu_31927 otu_458 otu_103686
otu_141421 otu_103377 otu_137862 otu_140681 otu_139569 otu_132840 otu_28533
otu_149986 otu_150257 otu_9095 otu_103794 otu_137663 otu_105584 otu_12720
otu_435 otu_99342 otu_109731 otu_127048 otu_3587 otu_137246 otu_101979 otu_705
otu_104928 otu_22760 otu_110143 otu_4277 otu_104193 otu_26647 otu_80968 otu_4530
otu_139634 otu_105777 otu_150297 otu_105926 otu_152151 otu_2953 otu_103606
otu_105595 otu_150991 otu_150139 otu_892 otu_107315 otu_146244 otu_151124
otu_27404 otu_146274 otu_99839 otu_3777 otu_151180 otu_150540 otu_79057 otu_8373
otu_150336 otu_102916 otu_23742 otu_132698 otu_141945 otu_146645 otu_146519
otu_97316 otu_137558 otu_113940 otu_103688 otu_23652 otu_141868 otu_8032
otu_139544 otu_3484 otu_152041 otu_106076 otu_99303 otu_103299 otu_150222
otu_7682 otu_24767 otu_136197 otu_136341 otu_146240 otu_121284 otu_19423
otu_102900 otu_100015 otu_106405 otu_105423 otu_102892 otu_22315 otu_24655
otu_22910 otu_29139 otu_34877 otu_22890 otu_11359 otu_105092 otu_136118
otu_128269 otu_28424 otu_146974 otu_151383 otu_12282 otu_4612 otu_149931
otu_132459 otu_58077 otu_79313 otu_127476 otu_138782 otu_104808 otu_147050
otu_33244 otu_126610 otu_103134 otu_150568 otu_7114 otu_945 otu_147491 otu_7692
otu_147638 otu_147354 otu_103742 otu_151474 otu_104000 otu_31064 otu_103938
otu_147886 otu_147042 otu_139160 otu_108095 otu_57417 otu_99246 otu_4079
otu_80037 otu_82036 otu_105407 otu_25074 otu_147033 otu_146372 otu_8326
otu_153089 otu_150187 otu_24389 otu_106666 otu_17075 otu_13070 otu_85325
otu_99392 otu_25230 otu_150167 otu_3966 otu_96944 otu_2833 otu_145407 otu_2183

[illegible]
otu_7682 otu_24767 otu_136197 otu_136341 otu_146240 otu_121284 otu_19423
otu_102900 otu_100015 otu_106405 otu_105423 otu_102892 otu_22315 otu_24655
otu_22910 otu_29139 otu_34877 otu_22890 otu_11359 otu_105092 otu_136118
otu_128269 otu_28424 otu_146974 otu_151383 otu_12282 otu_4612 otu_149931
otu_132459 otu_58077 otu_79313 otu_127476 otu_138782 otu_104808 otu_147050
otu_33244 otu_126610 otu_103134 otu_150568 otu_7114 otu_945 otu_147491 otu_7692
otu_147638 otu_147354 otu_103742 otu_151474 otu_104000 otu_31064 otu_103938
otu_147886 otu_147042 otu_139160 otu_108095 otu_57417 otu_99246 otu_4079
otu_80037 otu_82036 otu_105407 otu_25074 otu_147033 otu_146372 otu_8326
otu_153089 otu_150187 otu_24389 otu_106666 otu_17075 otu_13070 otu_85325
otu_99392 otu_25230 otu_150167 otu_3966 otu_96944 otu_2833 otu_145407 otu_2183
otu_7733 otu_103410 otu_151796 otu_99959 otu_139713 otu_128350 otu_146654
otu_4019 otu_150633 otu_79532 otu_80558 otu_103901 otu_153003 otu_151080
otu_142436 otu_105008 otu_137264 otu_103658 otu_107815 otu_4006 otu_3567
otu_24737 otu_150431 otu_139548 otu_5128 otu_154327 otu_30855 otu_3527
otu_146441 otu_99314 otu_153481 otu_144562 otu_148544 otu_150262 otu_29129
otu_36708 otu_104989 otu_144302 otu_105768 otu_141836 otu_151533 otu_141644
otu_2842 otu_41404 otu_14 otu_103097 otu_98271 otu_150835 otu_146258 otu_79143
otu_99639 otu_42309 otu_99428 otu_107392 otu_146663 otu_99328 otu_106430
otu_36012 otu_149735 otu_23388 otu_23240 otu_119504 otu_100072 otu_83931
otu_151416 otu_26812 otu_128253 otu_47514 otu_1623 otu_134684 otu_25150 otu_6888
otu_150871 otu_126974 otu_148261 otu_139547 otu_543 otu_16415 otu_142884
otu_7656 otu_149059 otu_103876 otu_103314 otu_103683 otu_149945 otu_151628
otu_108862 otu_1458 otu_140692 otu_13046 otu_103960 otu_22609 otu_146865
otu_146740 otu_113001 otu_107333 otu_128575 otu_108240 otu_8013 otu_103840
otu_146413 otu_104913 otu_150230 otu_142025 otu_23989 otu_103941 otu_106271
otu_2687 otu_149564 otu_41829 otu_79125 otu_25887 otu_104922 otu_10117 otu_36741
otu_138418 otu_31741 otu_2377 otu_154376 otu_149691 otu_105928 otu_1358
otu_106049 otu_106524 otu_142980 otu_1168 otu_105135 otu_151507 otu_146430
otu_18809 otu_103752 otu_103597 otu_112133 otu_105737 otu_142105 otu_28733
otu_789 otu_154823 otu_97263 otu_139152 otu_79419 otu_105761 otu_99330
otu_112703 otu_146713 otu_147371 otu_146273 otu_103435 otu_107171 otu_150425
otu_152113 otu_3907 otu_36666 otu_133062 otu_56659 otu_107830 otu_32205
otu_110628 otu_26189 otu_105639 otu_147822 otu_143789 otu_140938 otu_58515
otu_132466 otu_106956 otu_147642 otu_103781 otu_79076 otu_138437 otu_109581
otu_857 otu_6485 otu_102854 otu_29006 otu_1064 otu_139684 otu_150677 otu_22834
otu_153129 otu_148220 otu_142385 otu_145504 otu_143802 otu_137297 otu_139564
otu_103795 otu_107372 otu_139293 otu_81257 otu_103124 otu_146420 otu_10840
otu_144340 otu_6368 otu_24214 otu_97297 otu_10105 otu_149231 otu_3599 otu_3992
otu_132652 otu_4526 otu_65661 otu_27299 otu_146756 otu_151776 otu_1535
otu_141999 otu_142909 otu_25445 otu_150151 otu_154031 otu_8697 otu_145065
otu_150121 otu_103944 otu_146862 otu_153388 otu_126431 otu_150876 otu_19575
otu_146404 otu_84591 otu_106563 otu_20585 otu_126253 otu_10580 otu_59338
otu_144883 otu_149012 otu_147989 otu_99087 otu_137697 otu_139217 otu_5741
otu_99346 otu_562 otu_102977 otu_146825 otu_144171 otu_2266 otu_115635 otu_19237
otu_31024 otu_22408 otu_147756 otu_22992 otu_105228 otu_25355 otu_146231
otu_23324 otu_151673 otu_30867 otu_103514 otu_149757 otu_128995 otu_103619
otu_105538 otu_28150 otu_146556 otu_7686 otu_30163 otu_140705 otu_2480 otu_3164
otu_80408 otu_126862 otu_147055 otu_33374 otu_80567 otu_152043 otu_3573
otu_111795 otu_112786 otu_20509 otu_96984 otu_6309 otu_106201 otu_2725 otu_152882
otu_96702 otu_96769 otu_147973 otu_108522 otu_107350 otu_151608 otu_143891
otu_30120 otu_8787 otu_149706 otu_109526 otu_106508 otu_5003 otu_146979
otu_146926 otu_79108 otu_98292 otu_146812 otu_32959 otu_36468 otu_146599
otu_103133 otu_25780 otu_79541 otu_19767 otu_133467 otu_44275 otu_79628
otu_103042 otu_99725 otu_151814 otu_152935 otu_3842 otu_103354 otu_6188
otu_102981 otu_105050 otu_141950 otu_24313 otu_106217 otu_25762 otu_17403
otu_27696 otu_146856 otu_27868 otu_103704 otu_40244 otu_142270 otu_104004
otu_152156 otu_23779 otu_22549 otu_799 otu_147163 otu_99458 otu_105515 otu_16388
otu_150134 otu_139656 otu_104596 otu_103556 otu_104173 otu_139636 otu_98602
otu_104065 otu_150867 otu_144582 otu_103680 otu_139768 otu_24683 otu_6159
otu_147499 otu_152580 otu_6138 otu_32751 otu_105648 otu_154114 otu_34310
otu_151912 otu_35122 otu_148748 otu_2170 otu_103892 otu_79533 otu_138790
otu_79672 otu_4931 otu_112207 otu_144001 otu_104653 otu_78975 otu_154060
otu_103120 otu_17607 otu_107226 otu_103406 otu_17640 otu_104635 otu_146338
otu_140066 otu_139286 otu_145306 otu_23135 otu_147600 otu_103997 otu_23665
otu_105696 otu_97569 otu_97234 otu_108822 otu_135151 otu_140787 otu_8496
otu_151772 otu_6141 otu_3120 otu_148333 otu_1133 otu_139223 otu_23698 otu_6214
otu_103767 otu_24745 otu_36164 otu_142255 otu_105390 otu_22645 otu_19762
otu_99369 otu_104824 otu_105599 otu_132632 otu_4076 otu_34261 otu_141840 otu_575
otu_104108 otu_27364 otu_133220 otu_106683 otu_6861 otu_23829 otu_99882
otu_147177 otu_137780 otu_104925 otu_96652 otu_140768 otu_106014 otu_96912
otu_147972 otu_150206 otu_99223 otu_137300 otu_103663 otu_103145 otu_84156
otu_3259 otu_102378 otu_106392 otu_151915 otu_105589 otu_107854 otu_25073
otu_151852 otu_23368 otu_163248 otu_97349 otu_23450 otu_153255 otu_151555
otu_153529 otu_84164 otu_107236 otu_103651 otu_107814 otu_79468 otu_146248
otu_96752 otu_139664 otu_138215 otu_33573 otu_4632 otu_151307 otu_106586
otu_103923 otu_148076 otu_104869 otu_136721 otu_104890 otu_2603 otu_28271
otu_104023 otu_105876 otu_108685 otu_152451 otu_146551 otu_6179 otu_81507
otu_5358 otu_102331 otu_112340 otu_11952 otu_5439 otu_22638 otu_147138 otu_23688
otu_32372 otu_150562 otu_147793 otu_2160 otu_103260 otu_106553 otu_147252
otu_136993 otu_24577 otu_152061 otu_5360 otu_21844 otu_32843 otu_99276
otu_146973 otu_107380 otu_147021 otu_24515 otu_64819 otu_106780 otu_143910
otu_1902 otu_151316 otu_27420 otu_147597 otu_96730 otu_29806 otu_81565
otu_150780 otu_150688 otu_147768 otu_103889 otu_145606 otu_144452 otu_152339
otu_23155 otu_97342 otu_103611 otu_81164 otu_115229 otu_141608 otu_110891
otu_154759 otu_25817 otu_103907 otu_79692 otu_1248 otu_146503 otu_103407
otu_103961 otu_104053 otu_106596 otu_144610 otu_150185 otu_81999 otu_147703
otu_146609 otu_103981 otu_1787 otu_5624 otu_34104 otu_30063 otu_146877 otu_23245
otu_143952 otu_119426 otu_61791 otu_150137 otu_149011 otu_155778 otu_102956
otu_151112 otu_3109 otu_5227 otu_8562 otu_151833 otu_144188 otu_587 otu_103817
otu_147179 otu_84684 otu_35295 otu_147088 otu_136475 otu_96664 otu_23234
otu_106856 otu_18237 otu_289 otu_147097 otu_126982 otu_106041 otu_25847 otu_3647
otu_144843 otu_79589 otu_147428 otu_142321 otu_138472 otu_105597 otu_9281
otu_1054 otu_107265 otu_23049 otu_146582 otu_22499 otu_150572 otu_105408
otu_106632 otu_104244 otu_22682 otu_106743 otu_2427 otu_106812 otu_7957
otu_108699 otu_720 otu_151417 otu_104931 otu_105644 otu_150515 otu_103226
otu_66651 otu_135332 otu_28946 otu_105837 otu_153905 otu_104774 otu_107006
otu_8157 otu_100097 otu_105829 otu_1994 otu_23933 otu_27083 otu_150416 otu_644
otu_5189 otu_109829 otu_915 otu_36205 otu_24123 otu_80452 otu_4314 otu_6232
otu_107747 otu_99287 otu_2137 otu_3025 otu_150286 otu_5068 otu_80018 otu_81818
otu_3738 otu_102578 otu_149009 otu_151481 otu_103369 otu_105937 otu_60221
otu_110937 otu_104829 otu_150123 otu_147919 otu_14846 otu_3011 otu_24032
otu_151617 otu_142247 otu_132743 otu_106077 otu_146484 otu_79472 otu_142512
otu_37804 otu_152016 otu_146686 otu_80346 otu_39349 otu_104115 otu_99624
otu_110055 otu_109688 otu_22351 otu_147783 otu_154251 otu_103771 otu_127028
otu_99753 otu_139274 otu_150392 otu_126161 otu_941 otu_146357 otu_146660
otu_126756 otu_2312 otu_144838 otu_103551 otu_83105 otu_80342 otu_104730
otu_108505 otu_138535 otu_104069 otu_103399 otu_105749 otu_34483 otu_114211

otu_8157 otu_100097 otu_105829 otu_1994 otu_23933 otu_27083 otu_150416 otu_644
otu_5189 otu_109829 otu_915 otu_36205 otu_24123 otu_80452 otu_4314 otu_6232
otu_107747 otu_99287 otu_2137 otu_3025 otu_150286 otu_5068 otu_80018 otu_81818
otu_3738 otu_102578 otu_149009 otu_151481 otu_103369 otu_105937 otu_60221
otu_110937 otu_104829 otu_150123 otu_147919 otu_14846 otu_3011 otu_24032
otu_151617 otu_142247 otu_132743 otu_106077 otu_146484 otu_79472 otu_142512
otu_37804 otu_152016 otu_146686 otu_80346 otu_39349 otu_104115 otu_99624
otu_110055 otu_109688 otu_22351 otu_147783 otu_154251 otu_103771 otu_127028
otu_99753 otu_139274 otu_150392 otu_126161 otu_941 otu_146357 otu_146660
otu_126756 otu_2312 otu_144838 otu_103551 otu_83105 otu_80342 otu_104730
otu_108505 otu_138535 otu_104069 otu_103399 otu_105749 otu_34483 otu_114211
otu_139551 otu_110222 otu_102797 otu_1023 otu_31439 otu_108850 otu_30638
otu_2247 otu_106544 otu_103056 otu_147340 otu_147290 otu_104710 otu_79647
otu_105690 otu_142219 otu_137266 otu_24516 otu_147794 otu_34851 otu_139650
otu_108112 otu_102864 otu_8992 otu_105672 otu_97356 otu_151035 otu_37848
otu_141167 otu_8156 otu_3317 otu_152266 otu_57661 otu_3670 otu_52604 otu_126291
otu_81562 otu_150457 otu_137737 otu_127072 otu_273 otu_97487 otu_99520 otu_24901
otu_147057 otu_97015 otu_103701 otu_2512 otu_103874 otu_24019 otu_3970
otu_144705 otu_107154 otu_102947 otu_126227 otu_149652 otu_20045 otu_103906
otu_427 otu_151297 otu_153053 otu_8135 otu_82819 otu_82546 otu_98666 otu_147568
otu_102884 otu_100292 otu_105763 otu_163230 otu_27652 otu_17678 otu_7680
otu_150055 otu_2984 otu_99267 otu_26471 otu_3732 otu_991 otu_150375 otu_156416
otu_109231 otu_5482 otu_142020 otu_25431 otu_290 otu_108288 otu_5130 otu_24917
otu_147744 otu_7613 otu_2797 otu_146334 otu_107359 otu_28331 otu_141183
otu_105810 otu_126874 otu_27243 otu_103969 otu_4983 otu_146347 otu_26 otu_19757
otu_3024 otu_146625 otu_103681 otu_57952 otu_147587 otu_103465 otu_16588
otu_5183 otu_3504 otu_100360 otu_5771 otu_126612 otu_151656 otu_109542 otu_13966
otu_143610 otu_738 otu_102840 otu_143797 otu_2479 otu_7785 otu_22684 otu_79255
otu_103812 otu_139157 otu_103893 otu_23670 otu_978 otu_146561 otu_19204 otu_2195
otu_153631 otu_54573 otu_146265 otu_8275 otu_3470 otu_107355 otu_22814 otu_14418
otu_137093 otu_151090 otu_151869 otu_139584 otu_149994 otu_5527 otu_28930
otu_146689 otu_127 otu_127739 otu_141198 otu_147236 otu_150944 otu_21677
otu_105086 otu_138505 otu_41665 otu_144208 otu_163381 otu_79404 otu_151031
otu_108106 otu_106645 otu_29730 otu_26967 otu_139296 otu_2398 otu_99643
otu_151195 otu_30378 otu_23728 otu_13777 otu_139530 otu_103929 otu_15 otu_106408
otu_23550 otu_79695 otu_84926 otu_3411 otu_2250 otu_111997 otu_152696 otu_38857
otu_100115 otu_134531 otu_139686 otu_113412 otu_7615 otu_150218 otu_152381
otu_139578 otu_79477 otu_2255 otu_9201 otu_107664 otu_7089 otu_1005 otu_22411
otu_145369 otu_11459 otu_132937 otu_24914 otu_104998 otu_27908 otu_103934
otu_7657 otu_99248 otu_2024 otu_111080 otu_150104 otu_134342 otu_151141
otu_146853 otu_103045 otu_146687 otu_147789 otu_147758 otu_79083 otu_32301
otu_106264 otu_9080 otu_97329 otu_144549 otu_145317 otu_102941 otu_126160
otu_105865 otu_4327 otu_138509 otu_6492 otu_153700 otu_153331 otu_97930
otu_139145 otu_99576 otu_22902 otu_151684 otu_103503 otu_23412 otu_26020
otu_5837 otu_2524 otu_79869 otu_151380 otu_23352 otu_150175 otu_146522
otu_109989 otu_126534 otu_104340 otu_80265 otu_152705 otu_4318 otu_146225
otu_106651 otu_140926 otu_4994 otu_35409 otu_2821 otu_107052 otu_110471
otu_22556 otu_25938 otu_146360 otu_569 otu_146469 otu_142991 otu_22950
otu_132524 otu_126155 otu_22629 otu_8769 otu_146287 otu_80960 otu_10102
otu_96860 otu_128159 otu_5522 otu_147489 otu_79806 otu_103421 otu_80489 otu_3267
otu_117687 otu_508 otu_5082 otu_24482 otu_26343 otu_34436 otu_99417 otu_2561
otu_28523 otu_110426 otu_31290 otu_104050 otu_133383 otu_1965 otu_160482
otu_139356 otu_139568 otu_141854 otu_19566 otu_35874 otu_110513 otu_153265
otu_23033 otu_30085 otu_132490 otu_154195 otu_108914 otu_5198 otu_146950
otu_104116 otu_28439 otu_4990 otu_148189 otu_146327 otu_79412 otu_146754
otu_7644 otu_146275 otu_8145 otu_105612 otu_150211 otu_103532 otu_10291
otu_149479 otu_104020 otu_79517 otu_151429 otu_106847 otu_2952 otu_3066
otu_132482 otu_126509 otu_80316 otu_100413 otu_37903 otu_104105 otu_22699
otu_61406 otu_79624 otu_104857 otu_150640 otu_100312 otu_104085 otu_104362
otu_151046 otu_18983 otu_139243 otu_22704 otu_23976 otu_29202 otu_139261
otu_150727 otu_146597 otu_23466 otu_42911 otu_102974 otu_17137 otu_23468
otu_150629 otu_147817 otu_30417 otu_148739 otu_25645 otu_140682 otu_108194
otu_30563 otu_137195 otu_106094 otu_100345 otu_137616 otu_150261 otu_80123
otu_612 otu_16493 otu_17419 otu_105192 otu_32718 otu_4565 otu_22996 otu_80051
otu_154663 otu_109440 otu_142888 otu_150647 otu_2326 otu_4011 otu_104377
otu_106016 otu_147897 otu_150651 otu_99391 otu_152326 otu_107166 otu_104057
otu_32583 otu_151402 otu_20637 otu_147049 otu_107487 otu_22816 otu_6417
otu_136946 otu_33746 otu_97070 otu_1804 otu_146306 otu_147124 otu_153662
otu_24780 otu_147490 otu_25315 otu_152711 otu_136077 otu_105136 otu_149888
otu_25888 otu_98102 otu_105906 otu_24922 otu_1008 otu_103860 otu_140853
otu_97330 otu_28103 otu_26066 otu_134318 otu_155178 otu_105495 otu_140820
otu_147346 otu_139159 otu_150487 otu_79696 otu_6295 otu_99499 otu_105548
otu_97341 otu_97213 otu_26163 otu_21032 otu_145287 otu_47940 otu_99979 otu_25097
otu_137819 otu_103749 otu_107816 otu_133017 otu_153903 otu_36413 otu_28409
otu_106241 otu_27913 otu_127118 otu_150397 otu_79246 otu_101640 otu_102895
otu_3542 otu_12646 otu_109616 otu_99317 otu_149440 otu_104246 otu_23757 otu_4209
otu_104666 otu_23809 otu_3939 otu_23258 otu_2543 otu_107752 otu_28490 otu_146405
otu_778 otu_146719 otu_149932 otu_136445 otu_103230 otu_7788 otu_30248 otu_28470
otu_139250 otu_163821 otu_147791 otu_140395 otu_103292 otu_152443 otu_117536
otu_5503 otu_13378 otu_2421 otu_111001 otu_105888 otu_146264 otu_7896 otu_104119
otu_146450 otu_6268 otu_32231 otu_152603 otu_27019 otu_98626 otu_132493
otu_103829 otu_151162 otu_31076 otu_141025 otu_100201 otu_150460 otu_142041
otu_141949 otu_57926 otu_151993 otu_150066 otu_140857 otu_2108 otu_102931
otu_3815 otu_53202 otu_35530 otu_154285 otu_105518 otu_143944 otu_144762
otu_143578 otu_4784 otu_1726 otu_109234 otu_151364 otu_116938 otu_139757
otu_150233 otu_147620 otu_43588 otu_104025 otu_966 otu_146709 otu_137859
otu_99313 otu_153197 otu_136294 otu_152964 otu_23530 otu_96970 otu_146886
otu_49432 otu_142237 otu_22525 otu_27244 otu_78984 otu_25959 otu_25538
otu_103904 otu_146333 otu_2672 otu_22845 otu_3681 otu_83464 otu_17377 otu_144196
otu_137790 otu_105448 otu_97405 otu_32175 otu_79352 otu_97055 otu_2203 otu_34252
otu_28268 otu_3127 otu_152448 otu_138068 otu_104435 otu_109133 otu_99874
otu_97162 otu_22914 otu_147725 otu_106318 otu_147300 otu_3008 otu_140695
otu_137066 otu_132926 otu_25078 otu_109842 otu_15661 otu_150850 otu_147799
otu_5053 otu_38835 otu_151894 otu_150613 otu_22585 otu_2625 otu_136461 otu_39428
otu_146563 otu_22894 otu_97332 otu_109903 otu_8856 otu_150226 otu_151609
otu_22928 otu_2938 otu_147214 otu_149038 otu_27269 otu_152417 otu_99711
otu_103463 otu_24475 otu_126570 otu_66432 otu_36599 otu_103166 otu_153530
otu_872 otu_150125 otu_109622 otu_147018 otu_1327 otu_82113 otu_142863
otu_105502 otu_21466 otu_104676 otu_154675 otu_4014 otu_105880 otu_105882
otu_132283 otu_146209 otu_28375 otu_17813 otu_147775 otu_58277 otu_140801
otu_138419 otu_3864 otu_139198 otu_150894 otu_22381 otu_136487 otu_146623
otu_109523 otu_23289 otu_112752 otu_123337 otu_147781 otu_6680 otu_139276
otu_26752 otu_22346 otu_102978 otu_103738 otu_20678 otu_24647 otu_924 otu_146340
otu_141966 otu_149955 otu_20991 otu_103123 otu_137752 otu_27699 otu_132514
otu_150194 otu_12472 otu_151867 otu_132555 otu_38913 otu_104117 otu_142611
otu_104960 otu_105432 otu_115781 otu_142291 otu_79389 otu_106886 otu_103415
otu_2324 otu_142848 otu_99614 otu_149295 otu_137869 otu_103905 otu_104292
otu_105678 otu_35277 otu_4071 otu_32588 otu_98275 otu_146766 otu_24733
otu_138051 otu_141810 otu_132519 otu_4581 otu_79176 otu_3933 otu_128764

		otu_872 otu_150125 otu_109622 otu_147018 otu_1327 otu_82113 otu_142863 otu_105502 otu_21466 otu_104676 otu_154675 otu_4014 otu_105880 otu_105882 otu_132283 otu_146209 otu_28375 otu_17813 otu_147775 otu_58277 otu_140801 otu_138419 otu_3864 otu_139198 otu_150894 otu_22381 otu_136487 otu_146623 otu_109523 otu_23289 otu_112752 otu_123337 otu_147781 otu_6680 otu_139276 otu_26752 otu_22346 otu_102978 otu_103738 otu_20678 otu_24647 otu_924 otu_146340 otu_141966 otu_149955 otu_20991 otu_103123 otu_137752 otu_27699 otu_132514 otu_150194 otu_12472 otu_151867 otu_132555 otu_38913 otu_104117 otu_142611 otu_104960 otu_105432 otu_115781 otu_142291 otu_79389 otu_106886 otu_103415 otu_2324 otu_142848 otu_99614 otu_149295 otu_137869 otu_103905 otu_104292 otu_105678 otu_35277 otu_4071 otu_32588 otu_98275 otu_146766 otu_24733 otu_138051 otu_141810 otu_132519 otu_4581 otu_79176 otu_3933 otu_128764 otu_17195 otu_148513 otu_103565 otu_6152 otu_150005 otu_8218 otu_142018 otu_146366 otu_150500 otu_8232 otu_141498 otu_106047 otu_126791 otu_27590 otu_9939 otu_103872 otu_146653 otu_103779 otu_108765 otu_105574 otu_24915 otu_104028 otu_103479 otu_126225 otu_146454 otu_150111 otu_25451 otu_36408 otu_103388 otu_141234 otu_104171 otu_33165 otu_137327 otu_31922 otu_27253 otu_103945 otu_3078 otu_107805 otu_150822 otu_24451 otu_22409 otu_26900 otu_467 otu_150603 otu_106086 otu_910 otu_24484 otu_32896 otu_149452 otu_103086 otu_150701 otu_7634 otu_141954 otu_28791 otu_150063 otu_2638 otu_16945 otu_106308 otu_104168 otu_139481 otu_79330 otu_25329 otu_22827 otu_7082 otu_20724 otu_144597 otu_5097 otu_3395 otu_100191 otu_147216 otu_6162 otu_30028 otu_2855 otu_99281 otu_24989 otu_4441 otu_24299 otu_151669 otu_99361 otu_3463 otu_147716 otu_105087 otu_127579 otu_126371 otu_79029 otu_139896 otu_3641 otu_103037 otu_26988 otu_463 otu_6532 otu_8247 otu_146684 otu_4152 otu_38806 otu_104060 otu_150530 otu_132836 otu_106399 otu_105107 otu_35491 otu_103517 otu_107719 otu_144346 otu_141891 otu_1867 otu_58047 otu_22765 otu_79920 otu_5160 otu_106627 otu_149413 otu_996 otu_150461 otu_103538 otu_147414 otu_31004 otu_150107 otu_54219 otu_151379 otu_103572 otu_139279 otu_147043 otu_105846 otu_139512 otu_31848 otu_132535 otu_103015 otu_2499 otu_150127 otu_7980 otu_138425 otu_107089 otu_7447 otu_150120 otu_144023 otu_26583 otu_145212 otu_99231 otu_7850 otu_153363 otu_140098 otu_150384 otu_132805 otu_143834 otu_102907 otu_103948 otu_105566 otu_3496 otu_154806 otu_100034 otu_918 otu_103436 otu_8872 otu_104658 otu_103773 otu_142764 otu_25265 otu_147812 otu_139692 otu_153236 otu_22772 otu_23858 otu_14792 otu_138436 otu_146956 otu_151647 otu_104613 otu_113118 otu_132749 otu_154404 otu_23053 otu_103838 otu_146322 otu_147602 otu_26332 otu_2935 otu_80405 otu_151812 otu_127022 otu_25277 otu_99922 otu_41496 otu_105204 otu_102906 otu_126601 otu_126332 otu_147020 otu_103083 otu_55 otu_17959 otu_126247 otu_106022 otu_82380 otu_25853 otu_104787 otu_98883 otu_150332 otu_147615 otu_146205 otu_37801 otu_155417 otu_150505 otu_3492 otu_7008 otu_2433 otu_107463 otu_139816 otu_145278 otu_23604 otu_97353 otu_99365 otu_28437 otu_150804 otu_107066 otu_97491 otu_22389 otu_36321 otu_6380 otu_105433 otu_151234 otu_144057 otu_134314 otu_146999 otu_134372 otu_152188 otu_103918 otu_102800 otu_27950 otu_137152 otu_140951 otu_151980 otu_96666 otu_146572 otu_106357 otu_105669 otu_22677 otu_150116 otu_148966 otu_104003 otu_847 otu_150313 otu_104398 otu_5156 otu_100917 otu_8614 otu_122617 otu_132448 otu_103847 otu_103579 otu_105175 otu_110312 otu_138209 otu_6058 otu_120504 otu_24951 otu_31789 otu_23185 otu_79219
mpalafl mpolyfl mpolyiv	4	otu_150842 otu_4464 otu_150026 otu_153401
mpalafl mpalaiv soils	1	otu_91341
mpalafl mpalaiv mpolyiv	2	otu_349 otu_79170
mpalafl mpolyiv soils	2	otu_718 otu_143328
mpalaiv mpolyiv soils	1	otu_324
mpalafl mpolyfl	1610	otu_27656 otu_22839 otu_103333 otu_146534 otu_105921 otu_153545 otu_152192 otu_154722 otu_110004 otu_22332 otu_22649 otu_105452 otu_108766 otu_144744 otu_79563 otu_141055 otu_103320 otu_79104 otu_29891 otu_132496 otu_127852 otu_22403 otu_106114 otu_26742 otu_22706 otu_144056 otu_29590 otu_6894 otu_2674 otu_139781 otu_81356 otu_108173 otu_147647 otu_141266 otu_23525 otu_108549 otu_99485 otu_154883 otu_109134 otu_33405 otu_24392 otu_108606 otu_6063 otu_81427 otu_2775 otu_22596 otu_99942 otu_5123 otu_105938 otu_80969 otu_24246 otu_103788 otu_153712 otu_147814 otu_22848 otu_102960 otu_23631 otu_150902 otu_133048 otu_6396 otu_2816 otu_137279 otu_151689 otu_126335 otu_154687 otu_23633 otu_147533 otu_83172 otu_151348 otu_108693 otu_30885 otu_107211 otu_153085 otu_109303 otu_144306 otu_151096 otu_106034 otu_109815 otu_32602 otu_152280 otu_106262 otu_105679 otu_25494 otu_82060 otu_24046 otu_110490 otu_110598 otu_105471 otu_4689 otu_111236 otu_79332 otu_136776 otu_80798 otu_147109 otu_3028 otu_31800 otu_127495 otu_8599 otu_154708 otu_2236 otu_102913 otu_110645 otu_26909 otu_153125 otu_107093 otu_151996 otu_126502 otu_146325 otu_39394 otu_153027 otu_29284 otu_83195 otu_126391 otu_29644 otu_36672 otu_141242 otu_27337 otu_2127 otu_107257 otu_79684 otu_105721 otu_23806 otu_150085 otu_153461 otu_33938 otu_150743 otu_103648 otu_151170 otu_43833 otu_83304 otu_33817 otu_7331 otu_109480 otu_79293 otu_40643 otu_150627 otu_142104 otu_97048 otu_78956 otu_79218 otu_7814 otu_104112 otu_36712 otu_29205 otu_33808 otu_32143 otu_127977 otu_22377 otu_36053 otu_80722 otu_7911 otu_134442 otu_139742 otu_105480 otu_5924 otu_99230 otu_154658 otu_26433 otu_82739 otu_147704 otu_25886 otu_23500 otu_27715 otu_147459 otu_109374 otu_146482 otu_151381 otu_79821 otu_142416 otu_31296 otu_83450 otu_79652 otu_82903 otu_103641 otu_108526 otu_81186 otu_42826 otu_132854 otu_8274 otu_140923 otu_147622 otu_38228 otu_2629 otu_23853 otu_80211 otu_81462 otu_139869 otu_34328 otu_36995 otu_142164 otu_36804 otu_153978 otu_35562 otu_126514 otu_152225 otu_105676 otu_142372 otu_137250 otu_139632 otu_24597 otu_80903 otu_109103 otu_152018 otu_81803 otu_107931 otu_103796 otu_26279 otu_141986 otu_24735 otu_107737 otu_109017 otu_491 otu_110448 otu_39499 otu_137296 otu_81954 otu_109353 otu_147663 otu_139654 otu_8225 otu_4404 otu_110556 otu_106964 otu_3018 otu_109676 otu_23767 otu_3422 otu_31060 otu_152575 otu_82026 otu_80664 otu_103942 otu_154888 otu_6868 otu_126295 otu_79612 otu_107703 otu_3666 otu_83559 otu_83435 otu_5899 otu_126423 otu_150553 otu_141920 otu_3313 otu_28608 otu_103821 otu_6571 otu_25599 otu_100387 otu_22941 otu_96755 otu_80639 otu_37175 otu_147307 otu_144029 otu_151249 otu_141897 otu_33233 otu_108078 otu_6725 otu_105637 otu_151453 otu_151286 otu_106316 otu_142323 otu_25756 otu_28192 otu_8999 otu_36075 otu_99898 otu_107717 otu_110296 otu_152594 otu_150896 otu_99647 otu_138442 otu_132683 otu_82101 otu_151130 otu_108981 otu_109533 otu_132950 otu_146207 otu_154746 otu_144149 otu_31473 otu_22344 otu_153734 otu_105508 otu_22358 otu_154846 otu_84699 otu_108410 otu_152605 otu_132673 otu_29378 otu_82826 otu_103656 otu_106830 otu_81160 otu_29897 otu_106949 otu_151612 otu_103998 otu_146793 otu_127691 otu_152488 otu_153354 otu_126222 otu_2944 otu_7429 otu_151543 otu_110285 otu_140917 otu_106218 otu_23045 otu_30398 otu_23522 otu_24353 otu_25241 otu_32925 otu_110018 otu_26570 otu_99693 [illegible]

otu_103821 otu_6571 otu_25599 otu_100387 otu_22941 otu_96755 otu_80639 otu_37175
otu_147307 otu_144029 otu_151249 otu_141897 otu_33233 otu_108078 otu_6725
otu_105637 otu_151453 otu_151286 otu_106316 otu_142323 otu_25756 otu_28192
otu_8999 otu_36075 otu_99898 otu_107717 otu_110296 otu_152594 otu_150896
otu_99647 otu_138442 otu_132683 otu_82101 otu_151130 otu_108981 otu_109533
otu_132950 otu_146207 otu_154746 otu_144149 otu_31473 otu_22344 otu_153734
otu_105508 otu_22358 otu_154846 otu_84699 otu_108410 otu_152605 otu_132673
otu_29378 otu_82826 otu_103656 otu_106830 otu_81160 otu_29897 otu_106949
otu_151612 otu_103998 otu_146793 otu_127691 otu_152488 otu_153354 otu_126222
otu_2944 otu_7429 otu_151543 otu_110285 otu_140917 otu_106218 otu_23045
otu_30398 otu_23522 otu_24353 otu_25241 otu_32925 otu_110018 otu_26570 otu_99693
otu_28308 otu_147826 otu_153578 otu_80029 otu_146899 otu_37111 otu_23290
otu_83418 otu_100098 otu_150273 otu_150535 otu_79787 otu_104066 otu_110743
otu_33955 otu_147264 otu_154439 otu_83813 otu_2802 otu_35532 otu_22808 otu_23249
otu_107905 otu_109140 otu_153759 otu_23621 otu_22926 otu_151365 otu_127415
otu_140777 otu_141122 otu_163896 otu_24769 otu_152365 otu_25198 otu_22626
otu_141838 otu_80625 otu_139163 otu_152891 otu_9179 otu_83320 otu_3945
otu_103454 otu_83635 otu_127351 otu_134609 otu_142373 otu_153566 otu_2330
otu_153954 otu_103337 otu_29072 otu_105598 otu_153638 otu_22664 otu_103739
otu_152468 otu_80178 otu_32059 otu_2141 otu_26589 otu_81874 otu_150600 otu_4765
otu_106485 otu_2599 otu_33606 otu_151399 otu_102996 otu_147804 otu_146985
otu_30082 otu_24965 otu_30697 otu_141875 otu_110499 otu_80593 otu_102835
otu_99321 otu_38088 otu_103864 otu_107545 otu_151547 otu_126800 otu_151415
otu_81146 otu_35970 otu_126551 otu_22357 otu_83238 otu_163353 otu_3055 otu_33941
otu_108354 otu_152633 otu_107098 otu_96844 otu_36842 otu_105413 otu_79987
otu_23741 otu_136709 otu_81523 otu_81085 otu_8906 otu_31863 otu_2834 otu_22472
otu_147483 otu_24596 otu_30646 otu_147835 otu_103178 otu_142292 otu_41402
otu_144375 otu_144315 otu_152899 otu_22869 otu_6568 otu_112839 otu_27814
otu_96869 otu_83026 otu_32597 otu_26952 otu_26429 otu_2196 otu_31389 otu_37048
otu_150501 otu_153619 otu_31745 otu_24164 otu_4538 otu_143878 otu_27648
otu_109553 otu_108982 otu_26942 otu_137347 otu_81077 otu_150934 otu_79414
otu_134575 otu_153408 otu_33887 otu_104040 otu_127232 otu_138142 otu_132759
otu_151263 otu_103914 otu_27503 otu_106766 otu_79106 otu_152968 otu_151997
otu_144388 otu_2920 otu_2856 otu_106139 otu_108498 otu_35622 otu_36370 otu_3191
otu_30455 otu_36636 otu_35510 otu_83497 otu_30606 otu_28156 otu_80704 otu_22384
otu_38131 otu_141973 otu_35344 otu_103228 otu_154446 otu_80529 otu_103982
otu_143839 otu_24892 otu_81106 otu_29731 otu_154232 otu_25044 otu_128245
otu_96746 otu_109300 otu_152002 otu_103676 otu_152692 otu_152460 otu_152943
otu_139682 otu_2732 otu_110395 otu_153034 otu_110502 otu_139976 otu_141910
otu_132804 otu_142363 otu_105935 otu_80537 otu_80015 otu_5540 otu_150299
otu_79828 otu_6729 otu_100339 otu_152918 otu_96896 otu_137254 otu_80000
otu_152367 otu_152519 otu_147191 otu_106545 otu_109719 otu_31367 otu_82983
otu_22917 otu_25490 otu_150724 otu_150619 otu_28167 otu_23448 otu_5980 otu_82728
otu_110309 otu_7805 otu_103452 otu_152886 otu_8549 otu_132848 otu_110148
otu_24854 otu_154602 otu_147389 otu_26305 otu_143857 otu_104098 otu_153326
otu_150556 otu_96938 otu_105590 otu_24978 otu_27121 otu_105528 otu_108081
otu_26879 otu_126663 otu_3676 otu_100227 otu_105775 otu_99791 otu_80850
otu_152430 otu_127965 otu_23991 otu_147525 otu_105788 otu_127093 otu_25698
otu_79617 otu_7609 otu_31892 otu_107471 otu_24557 otu_150314 otu_25858
otu_151539 otu_33735 otu_154730 otu_30544 otu_3790 otu_32416 otu_146617 otu_3457
otu_99483 otu_25911 otu_99857 otu_35478 otu_147039 otu_81377 otu_109806 otu_3467
otu_31662 otu_106720 otu_146270 otu_150345 otu_99575 otu_108045 otu_24556
otu_103993 otu_33600 otu_152138 otu_109121 otu_153607 otu_29130 otu_107822
otu_32279 otu_105642 otu_33526 otu_31069 otu_5044 otu_109872 otu_133033
otu_80059 otu_22338 otu_152570 otu_27955 otu_151288 otu_143874 otu_163273
otu_36645 otu_38296 otu_23587 otu_2434 otu_28683 otu_34767 otu_100286 otu_103751
otu_139765 otu_155040 otu_28403 otu_152505 otu_153395 otu_110591 otu_103624
otu_106974 otu_103661 otu_106012 otu_3632 otu_146820 otu_31844 otu_108168
otu_137866 otu_80635 otu_34800 otu_26686 otu_136978 otu_151444 otu_127712
otu_138176 otu_103815 otu_34088 otu_32381 otu_22313 otu_108100 otu_81820
otu_110114 otu_33323 otu_151242 otu_137 otu_31433 otu_22463 otu_136712 otu_110407
otu_3199 otu_30781 otu_81050 otu_24045 otu_106451 otu_155495 otu_105520
otu_107124 otu_107933 otu_152957 otu_147843 otu_109547 otu_146398 otu_151830
otu_99546 otu_153600 otu_900 otu_105769 otu_151093 otu_25065 otu_103357
otu_79915 otu_107486 otu_80275 otu_79625 otu_33637 otu_43660 otu_36081
otu_107600 otu_107349 otu_99430 otu_140002 otu_32204 otu_139298 otu_2649
otu_103224 otu_142197 otu_150539 otu_26690 otu_615 otu_110546 otu_103697
otu_99880 otu_2912 otu_35721 otu_2492 otu_103891 otu_110612 otu_153752 otu_81948
otu_24374 otu_27960 otu_108153 otu_134678 otu_99452 otu_99525 otu_24525
otu_152355 otu_108185 otu_103810 otu_126530 otu_80756 otu_29522 otu_99344
otu_146953 otu_150403 otu_109056 otu_33050 otu_35416 otu_150459 otu_103063
otu_27869 otu_110633 otu_79822 otu_105735 otu_29719 otu_127673 otu_105657
otu_3555 otu_31934 otu_153890 otu_106268 otu_6647 otu_105506 otu_83161 otu_26731
otu_106970 otu_35505 otu_26196 otu_29764 otu_107839 otu_29473 otu_32075
otu_104024 otu_143806 otu_138483 otu_108630 otu_37205 otu_79198 otu_147184
otu_83631 otu_103806 otu_96959 otu_28828 otu_108356 otu_141850 otu_30603
otu_108966 otu_5572 otu_33427 otu_109284 otu_107270 otu_110415 otu_132515
otu_107170 otu_79393 otu_153654 otu_139521 otu_152791 otu_150796 otu_25734
otu_29980 otu_23311 otu_151382 otu_154433 otu_147513 otu_105624 otu_103786
otu_79794 otu_27816 otu_137284 otu_31074 otu_28602 otu_127121 otu_80027
otu_151599 otu_107357 otu_23035 otu_32563 otu_32523 otu_32070 otu_139202
otu_108899 otu_150570 otu_31173 otu_107238 otu_154624 otu_151241 otu_108289
otu_29034 otu_146634 otu_151300 otu_32390 otu_103568 otu_30099 otu_126943
otu_153371 otu_153881 otu_150064 otu_153706 otu_103072 otu_153141 otu_24751
otu_25697 otu_142267 otu_150193 otu_36677 otu_79173 otu_34251 otu_83382
otu_146601 otu_139866 otu_80556 otu_150649 otu_35601 otu_109876 otu_80466
otu_150783 otu_79933 otu_146752 otu_107768 otu_141827 otu_24994 otu_81610
otu_110402 otu_28068 otu_150227 otu_107041 otu_5266 otu_26403 otu_31729
otu_138178 otu_888 otu_106682 otu_24916 otu_154891 otu_107083 otu_107979
otu_110526 otu_83902 otu_109680 otu_3419 otu_107444 otu_112101 otu_151471
otu_150759 otu_139581 otu_152694 otu_22516 otu_134316 otu_151125 otu_152008
otu_1007 otu_5853 otu_99677 otu_103873 otu_147636 otu_28511 otu_80221 otu_25658
otu_105710 otu_154951 otu_140052 otu_154024 otu_3550 otu_150612 otu_23963
otu_127026 otu_29358 otu_31351 otu_24296 otu_144250 otu_42406 otu_99868
otu_146666 otu_103716 otu_83693 otu_23377 otu_80173 otu_114212 otu_109227
otu_150296 otu_103654 otu_29807 otu_83078 otu_108042 otu_99716 otu_7109
otu_81801 otu_103462 otu_150396 otu_156447 otu_106229 otu_24787 otu_147666
otu_137247 otu_32184 otu_108353 otu_107467 otu_146280 otu_151859 otu_99568
otu_31508 otu_34565 otu_35127 otu_36520 otu_151988 otu_105489 otu_24991
otu_110087 otu_41534 otu_102959 otu_99965 otu_973 otu_105877 otu_33986
otu_150836 otu_150661 otu_106134 otu_3707 otu_106018 otu_99531 otu_107078
otu_41306 otu_36778 otu_150353 otu_106457 otu_30894 otu_27693 otu_23171
otu_79348 otu_110021 otu_108738 otu_152947 otu_103185 otu_28055 otu_5737
otu_28135 otu_139929 otu_26311 otu_126600 otu_144484 otu_108817 otu_139923
otu_139621 otu_24809 otu_103243 otu_152046 otu_153081 otu_24584 otu_9082
otu_146304 otu_126544 otu_142228 otu_150885 otu_146741 otu_79430 otu_105947
otu_32826 otu_34591 otu_30197 otu_127689 otu_82090 otu_108972 otu_108259
otu_142024 otu_154388 otu_153347 otu_106078 otu_8169 otu_3282 otu_106719
otu_150430 otu_31082 otu_5443 otu_127361 otu_1012 otu_105675 otu_99574 otu_33612

		[illegible]<br>otu_31508 otu_34565 otu_35127 otu_36520 otu_151988 otu_105489 otu_24991<br>otu_110087 otu_41534 otu_102959 otu_99965 otu_973 otu_105877 otu_33986<br>otu_150836 otu_150661 otu_106134 otu_3707 otu_106018 otu_99531 otu_107078<br>otu_41306 otu_36778 otu_150353 otu_106457 otu_30894 otu_27693 otu_23171<br>otu_79348 otu_110021 otu_108738 otu_152947 otu_103185 otu_28055 otu_5737<br>otu_28135 otu_139929 otu_26311 otu_126600 otu_144484 otu_108817 otu_139923<br>otu_139621 otu_24809 otu_103243 otu_152046 otu_153081 otu_24584 otu_9082<br>otu_146304 otu_126544 otu_142228 otu_150885 otu_146741 otu_79430 otu_105947<br>otu_32826 otu_34591 otu_30197 otu_127689 otu_82090 otu_108972 otu_108259<br>otu_142024 otu_154388 otu_153347 otu_106078 otu_8169 otu_3282 otu_106719<br>otu_150430 otu_31082 otu_5443 otu_127361 otu_1012 otu_105675 otu_99574 otu_33612<br>otu_24001 otu_30652 otu_38869 otu_5936 otu_27520 otu_23383 otu_105617 otu_109379<br>otu_107229 otu_96963 otu_25785 otu_36018 otu_81340 otu_79918 otu_34938 otu_7927<br>otu_36352 otu_107250 otu_109211 otu_110498 otu_41533 otu_126751 otu_128040<br>otu_83956 otu_142333 otu_103353 otu_23482 otu_41623 otu_151078 otu_2655<br>otu_26677 otu_126647 otu_152399 otu_144686 otu_152566 otu_136729 otu_28517<br>otu_7365 otu_102855 otu_110575 otu_83326 otu_32445 otu_81241 otu_25963<br>otu_146669 otu_151126 otu_106370 otu_32377 otu_27830 otu_22687 otu_80959<br>otu_36719 otu_82567 otu_8853 otu_83864 otu_153359 otu_147545 otu_35126<br>otu_153726 otu_109578 otu_104128 otu_36489 otu_103718 otu_150249 otu_30201<br>otu_108342 otu_109106 otu_153480 otu_81499 otu_138147 otu_109831 otu_103383<br>otu_152459 otu_22882 otu_108029 otu_151534 otu_150787 otu_143992 otu_33224<br>otu_147656 otu_30086 otu_150860 otu_103570 otu_79574 otu_142805 otu_35591<br>otu_138637 otu_153972 otu_109329 otu_133021 otu_147276 otu_142613 otu_79830<br>otu_151862 otu_151189 otu_79346 otu_80503 otu_151496 otu_108326 otu_139753<br>otu_32008 otu_154325 otu_105830 otu_147810 otu_31611 otu_126392 otu_150968<br>otu_106942 otu_150236 otu_141885 otu_29391 otu_126250 otu_23092 otu_103448<br>otu_24977 otu_36560 otu_23506 otu_151650 otu_25083 otu_5579 otu_83633 otu_84020<br>otu_107707 otu_99513 otu_36307 otu_23129 otu_140888 otu_107994 otu_109286<br>otu_35170 otu_23744 otu_106375 otu_108530 otu_137633 otu_6651 otu_163893<br>otu_105786 otu_30434 otu_29283 otu_890 otu_150799 otu_107347 otu_105496 otu_3861<br>otu_108080 otu_26359 otu_150884 otu_106333 otu_103695 otu_103160 otu_107118<br>otu_137820 otu_106480 otu_27446 otu_141102 otu_147201 otu_24156 otu_150242<br>otu_6779 otu_30558 otu_82358 otu_151020 otu_83696 otu_152376 otu_80097 otu_31388<br>otu_107505 otu_152252 otu_37214 otu_152503 otu_80398 otu_109862 otu_5547<br>otu_151994 otu_106633 otu_127281 otu_147102 otu_22374 otu_134834 otu_107694<br>otu_106699 otu_100012 otu_150928 otu_99426 otu_103659 otu_150141 otu_126205<br>otu_26655 otu_3943 otu_81578 otu_84139 otu_3337 otu_126413 otu_142367 otu_103732<br>otu_106400 otu_8304 otu_83548 otu_153209 otu_109177 otu_147738 otu_99613<br>otu_41246 otu_106220 otu_146303 otu_81453 otu_23442 otu_32678 otu_139187<br>otu_22532 otu_29826 otu_108433 otu_142097 otu_23079 otu_138173 otu_103516<br>otu_22632 otu_25979 otu_147256 otu_107617 otu_106198 otu_150248 otu_100035<br>otu_108147 otu_32081 otu_37577 otu_33174 otu_82957 otu_106326 otu_99988<br>otu_150813 otu_33376 otu_3936 otu_6088 otu_36506 otu_104019 otu_103571 otu_24564<br>otu_141257 otu_105719 otu_132585 otu_136078 otu_151761 otu_126402 otu_141220<br>otu_142799 otu_32868 otu_137870 otu_83652 otu_144298 otu_80768 otu_81151<br>otu_32957 otu_24349 otu_140696 otu_2972 otu_8089 otu_134852 otu_2772 otu_106441<br>otu_146913 otu_105686 otu_25166 otu_163226 otu_4468 otu_127859 otu_110027<br>otu_28488 otu_23887 otu_151089 otu_136986 otu_84097 otu_113385 otu_108896<br>otu_81314 otu_152663 otu_30951 otu_99778 otu_26136 otu_83679 otu_36564<br>otu_103044 otu_26057 otu_31071 otu_7517 otu_147171 otu_22991 otu_150268<br>otu_147576 otu_150308 otu_108849 otu_25586 otu_151801 otu_107701 otu_127755<br>otu_26697 otu_22425 otu_139168 otu_28019 otu_7629 otu_107269 otu_25231<br>otu_132656 otu_26463 otu_154530 otu_103946 otu_30131 otu_151428 otu_146863<br>otu_105560 otu_103714 otu_23927 otu_3187 otu_96983 otu_144434 otu_137641<br>otu_7610 otu_151562 otu_6488 otu_142148 otu_103760 otu_22621 otu_108404<br>otu_151837 otu_153630 otu_105874 otu_153189 otu_23076 otu_652 otu_147624<br>otu_126958 otu_108451 otu_164292 otu_156470 otu_151715 otu_141129 otu_22438<br>otu_38218 otu_152638 otu_25593 otu_153597 otu_104044 otu_144780 otu_153676<br>otu_79121 otu_139794 otu_126990 otu_35580 otu_81184 otu_147222 otu_32431<br>otu_154703 otu_152862 otu_34564 otu_146632 otu_151734 otu_1024 otu_147605<br>otu_126965 otu_110416 otu_150695 otu_22864 otu_26229 otu_106762 otu_107128<br>otu_138532 otu_103798 otu_152454 otu_82196 otu_99829 otu_29011 otu_25772<br>otu_127309 otu_142277 otu_103811 otu_151666 otu_152120 otu_24404 otu_151638<br>otu_103617 otu_81549 otu_126790 otu_36463 otu_25919 otu_28758 otu_100148<br>otu_151030 otu_37194 otu_108984 otu_150732 otu_99461 otu_155670 otu_8116<br>otu_154174 otu_9074 otu_31124 otu_154798 otu_81004 otu_80431 otu_2893 otu_153977<br>otu_107024 otu_106484 otu_79094 otu_106576 otu_22802 otu_163333 otu_142405<br>otu_108724 otu_100436 otu_106843 otu_151206 otu_34336 otu_37208 otu_38885<br>otu_31313 otu_146546 otu_6290 otu_147539 otu_7165 otu_7533 otu_150691 otu_32824<br>otu_26344 otu_152744 otu_150709 otu_150816 otu_23472 otu_39542 otu_32815<br>otu_107177 otu_152233 otu_24130 otu_82969 otu_152396 otu_152052 otu_3126<br>otu_80719 otu_147710 otu_29775 otu_30219 otu_137319 otu_107373 otu_23005<br>otu_150987 otu_103666 otu_144072 otu_27320 otu_26532 otu_80521 otu_126212<br>otu_154355 otu_132679 otu_151815 otu_144771 otu_97052 otu_147619 otu_25085<br>otu_7418 otu_154313 otu_25221 otu_142021 otu_22787 otu_3745 otu_31645 otu_25738<br>otu_79415 otu_107457 otu_154447 otu_6390 otu_5892 otu_24957 otu_150912 otu_29033<br>otu_30256 otu_81467 otu_104041 otu_29596 otu_107762 otu_153524 otu_6456<br>otu_96926 otu_108560 otu_103202 otu_82050 otu_128927 otu_29939 otu_153434<br>otu_8666 otu_147382 otu_802 otu_23417 otu_106845 otu_150737 otu_127284<br>otu_137914 otu_82882 otu_151554 otu_150205 otu_151492 otu_127875 otu_23445<br>otu_146568 otu_153568 otu_106204 otu_4619 otu_103508 otu_163832 otu_33333<br>otu_99856 otu_110020 otu_146611 otu_100084 otu_150955 otu_150321 otu_31023<br>otu_82682 otu_150838 otu_33771 otu_8083 otu_4722 otu_103675 otu_27018 otu_141237<br>otu_100089 otu_108215 otu_146496 otu_106635 otu_80631 otu_109520 otu_27659<br>otu_3740 otu_106146 otu_30668 otu_22342 otu_142027 otu_103544 otu_150904<br>otu_152698 otu_142398 otu_153035 otu_107679 otu_105501 otu_138511 otu_24879<br>otu_36567 otu_4426 otu_97032 otu_164248 otu_23524 otu_107604 otu_144050<br>otu_102858 otu_32617 otu_7494 otu_105723 otu_23884 otu_3953 otu_134645<br>otu_151167 otu_80034 otu_107627 otu_151128 otu_103386 otu_36357 otu_34890<br>otu_79907 otu_29198 otu_30948 otu_27122 otu_99611 otu_146813 otu_35285<br>otu_154620 otu_151525 otu_108477 otu_25618 otu_153048 otu_152645 otu_31921<br>otu_4521 otu_5779 otu_96682
mpalafl mpalaiv	1	otu_78206
mpalafl soils	7525	otu_99548 otu_83369 otu_23409 otu_23586 otu_2510 otu_79214 otu_33020 otu_79797<br>otu_36424 otu_3444 otu_25047 otu_137556 otu_145056 otu_97223 otu_22805 otu_79782<br>otu_24163 otu_79124 otu_100128 otu_153454 otu_32662 otu_102828 otu_2858<br>otu_12229 otu_27233 otu_147397 otu_79207 otu_2987 otu_144189 otu_21899 otu_4254<br>otu_36440 otu_99512 otu_17291 otu_152152 otu_22670 otu_35717 otu_126175<br>otu_151836 otu_143879 otu_102809 otu_2874 otu_96878 otu_146676 otu_2527<br>otu_109237 otu_146455 otu_152738 otu_32616 otu_3674 otu_103073 otu_4574<br>otu_80179 otu_27927 otu_79588 otu_79966 otu_97244 otu_81156 otu_134202 otu_31292<br>otu_132469 otu_106040 otu_3139 otu_3827 otu_137835 otu_22347 otu_146949<br>otu_25962 otu_129397 otu_151991 otu_104233 otu_146385 otu_1391 otu_99700<br>otu_27538 otu_5180 otu_151077 otu_147016 otu_13783 otu_142658 otu_150559<br>otu_30344 otu_105789 otu_99006 otu_99721 otu_103090 otu_80762 otu_146208<br>[illegible]

otu_36424 otu_3444 otu_25047 otu_137556 otu_145056 otu_97223 otu_22805 otu_79782
otu_24163 otu_79124 otu_100128 otu_153454 otu_32662 otu_102828 otu_2858
otu_12229 otu_27233 otu_147397 otu_79207 otu_2987 otu_144189 otu_21899 otu_4254
otu_36440 otu_99512 otu_17291 otu_152152 otu_22670 otu_35717 otu_126175
otu_151836 otu_143879 otu_102809 otu_2874 otu_96878 otu_146676 otu_2527
otu_109237 otu_146455 otu_152738 otu_32616 otu_3674 otu_103073 otu_4574
otu_80179 otu_27927 otu_79588 otu_79966 otu_97244 otu_81156 otu_134202 otu_31292
otu_132469 otu_106040 otu_3139 otu_3827 otu_137835 otu_22347 otu_146949
otu_25962 otu_129397 otu_151991 otu_104233 otu_146385 otu_1391 otu_99700
otu_27538 otu_5180 otu_151077 otu_147016 otu_13783 otu_142658 otu_150559
otu_30344 otu_105789 otu_99006 otu_99721 otu_103090 otu_80762 otu_146208
otu_1053 otu_31782 otu_126528 otu_83823 otu_7277 otu_106806 otu_81629 otu_106640
otu_147038 otu_107697 otu_31705 otu_23658 otu_51990 otu_138446 otu_146493
otu_106011 otu_29869 otu_106679 otu_97037 otu_142572 otu_34817 otu_150231
otu_139315 otu_30226 otu_152684 otu_89097 otu_146452 otu_97063 otu_27828
otu_146658 otu_148556 otu_103181 otu_31911 otu_3644 otu_34512 otu_80115
otu_139301 otu_106758 otu_132720 otu_134765 otu_82858 otu_22312 otu_137882
otu_33420 otu_795 otu_2548 otu_102868 otu_126841 otu_29268 otu_102845 otu_749
otu_12890 otu_64427 otu_99586 otu_134453 otu_146223 otu_35906 otu_79063
otu_154797 otu_151632 otu_6809 otu_2206 otu_99873 otu_12885 otu_23010 otu_35560
otu_106009 otu_101119 otu_105486 otu_151237 otu_139245 otu_133123 otu_150937
otu_151209 otu_61783 otu_99813 otu_83332 otu_126597 otu_142084 otu_1833
otu_34892 otu_97240 otu_81445 otu_136592 otu_84141 otu_35474 otu_99843
otu_151512 otu_139539 otu_3304 otu_25094 otu_147166 otu_105989 otu_81316
otu_157258 otu_2316 otu_163999 otu_3803 otu_132594 otu_28252 otu_137958 otu_5125
otu_127617 otu_26939 otu_105443 otu_134620 otu_154399 otu_2174 otu_146506
otu_99554 otu_146318 otu_80859 otu_27711 otu_3978 otu_51118 otu_134597 otu_24820
otu_136713 otu_145364 otu_100041 otu_3377 otu_83007 otu_105563 otu_80230
otu_108071 otu_36774 otu_27643 otu_151640 otu_80183 otu_7353 otu_26219
otu_150088 otu_11066 otu_147019 otu_137810 otu_1383 otu_5018 otu_3216 otu_152825
otu_163224 otu_80711 otu_33351 otu_163228 otu_7412 otu_106243 otu_6517 otu_28236
otu_23880 otu_79469 otu_109918 otu_80671 otu_145143 otu_150266 otu_91365
otu_79011 otu_79434 otu_6293 otu_30187 otu_126815 otu_151420 otu_107330
otu_152812 otu_136115 otu_35113 otu_84085 otu_83754 otu_138531 otu_99444
otu_24689 otu_52437 otu_146874 otu_22786 otu_109032 otu_138148 otu_97621
otu_28682 otu_82543 otu_893 otu_27877 otu_146604 otu_109711 otu_22339 otu_407
otu_80939 otu_80562 otu_132889 otu_163830 otu_97021 otu_79479 otu_3394 otu_28730
otu_102804 otu_153132 otu_153583 otu_150289 otu_132798 otu_149334 otu_157076
otu_82740 otu_106143 otu_141873 otu_143864 otu_27718 otu_146285 otu_30273
otu_24511 otu_4283 otu_126162 otu_104667 otu_81175 otu_91012 otu_143942 otu_27032
otu_109965 otu_24007 otu_105464 otu_686 otu_150330 otu_146959 otu_106344
otu_152428 otu_4729 otu_26852 otu_25294 otu_138327 otu_150946 otu_2115
otu_146557 otu_136728 otu_150581 otu_6673 otu_99623 otu_30969 otu_127623
otu_152877 otu_146685 otu_3226 otu_109326 otu_153897 otu_127773 otu_79973
otu_132595 otu_22738 otu_81208 otu_139818 otu_106294 otu_30349 otu_134344
otu_22935 otu_79200 otu_151497 otu_93920 otu_146770 otu_31241 otu_79981
otu_147894 otu_1189 otu_26827 otu_154254 otu_141917 otu_1321 otu_25735
otu_146644 otu_146520 otu_23354 otu_25788 otu_35251 otu_1544 otu_139800 otu_4042
otu_83623 otu_3926 otu_3612 otu_23303 otu_138123 otu_138153 otu_103505
otu_153943 otu_814 otu_141473 otu_6013 otu_24778 otu_37200 otu_10357 otu_2756
otu_126260 otu_147012 otu_163767 otu_151545 otu_33135 otu_109640 otu_147094
otu_150767 otu_97986 otu_3468 otu_4663 otu_152445 otu_6444 otu_104632 otu_106653
otu_144201 otu_148841 otu_79180 otu_150738 otu_25750 otu_27860 otu_26383
otu_4115 otu_109757 otu_28432 otu_23426 otu_24594 otu_4684 otu_19586 otu_5218
otu_96894 otu_138132 otu_4374 otu_22799 otu_156709 otu_136762 otu_29918
otu_132817 otu_32244 otu_82719 otu_102902 otu_22811 otu_153699 otu_106687
otu_30073 otu_121816 otu_27111 otu_27240 otu_107895 otu_2970 otu_108920 otu_33274
otu_144847 otu_141085 otu_36992 otu_2677 otu_105700 otu_702 otu_3606 otu_4009
otu_22911 otu_81252 otu_103025 otu_147071 otu_108780 otu_1916 otu_133512
otu_96724 otu_29682 otu_110473 otu_6637 otu_97707 otu_28479 otu_3212 otu_99488
otu_58932 otu_126377 otu_25655 otu_33345 otu_79018 otu_163285 otu_132934 otu_735
otu_24037 otu_153324 otu_2889 otu_102575 otu_151918 otu_126795 otu_6686
otu_28030 otu_146397 otu_150184 otu_25949 otu_23007 otu_24682 otu_103403
otu_147215 otu_81153 otu_634 otu_14524 otu_25274 otu_4259 otu_79391 otu_126226
otu_3793 otu_31078 otu_481 otu_97007 otu_25522 otu_11469 otu_140710 otu_37138
otu_27002 otu_80945 otu_3222 otu_118213 otu_140949 otu_126457 otu_139540
otu_2513 otu_126831 otu_150068 otu_25477 otu_30482 otu_151131 otu_141899
otu_139536 otu_150497 otu_132721 otu_106923 otu_5131 otu_4545 otu_30940
otu_86801 otu_4386 otu_22382 otu_146933 otu_81315 otu_117830 otu_154813
otu_52927 otu_29422 otu_103431 otu_1070 otu_132491 otu_33667 otu_134707
otu_26607 otu_154162 otu_127176 otu_140683 otu_253 otu_103251 otu_84105
otu_151787 otu_6559 otu_2585 otu_80220 otu_146262 otu_134366 otu_99538 otu_27302
otu_2532 otu_25954 otu_153224 otu_56903 otu_2950 otu_26199 otu_137857 otu_97249
otu_150490 otu_153916 otu_24701 otu_137283 otu_22747 otu_4018 otu_27657
otu_141877 otu_11790 otu_152985 otu_1482 otu_32862 otu_143643 otu_87084
otu_10036 otu_97017 otu_2714 otu_3885 otu_28323 otu_149596 otu_137073 otu_153523
otu_4414 otu_36121 otu_29276 otu_128 otu_1429 otu_99502 otu_1161 otu_33123
otu_80806 otu_146707 otu_23227 otu_78953 otu_144006 otu_134381 otu_2883
otu_147327 otu_27928 otu_80766 otu_32267 otu_23312 otu_2491 otu_22860 otu_140900
otu_132665 otu_105512 otu_133076 otu_25226 otu_29561 otu_33767 otu_81793
otu_23277 otu_3459 otu_110237 otu_103398 otu_2106 otu_5262 otu_12241 otu_146627
otu_79408 otu_137030 otu_79424 otu_17216 otu_82820 otu_1830 otu_98868 otu_146694
otu_150223 otu_141796 otu_146315 otu_26582 otu_25024 otu_144247 otu_137314
otu_79358 otu_32642 otu_36190 otu_140872 otu_6150 otu_107217 otu_99494 otu_82223
otu_137828 otu_145106 otu_2566 otu_126912 otu_79577 otu_2664 otu_137834
otu_127073 otu_108419 otu_2201 otu_104360 otu_103560 otu_36253 otu_18292
otu_28278 otu_134350 otu_153380 otu_151713 otu_29252 otu_151084 otu_5590
otu_137248 otu_3683 otu_496 otu_134529 otu_2179 otu_137298 otu_33498 otu_127668
otu_126766 otu_150547 otu_5270 otu_108202 otu_36065 otu_126679 otu_33602
otu_152670 otu_6967 otu_4768 otu_5629 otu_108531 otu_7227 otu_28233 otu_30326
otu_97868 otu_103089 otu_24021 otu_127589 otu_100146 otu_36000 otu_79311
otu_141073 otu_153664 otu_142218 otu_3622 otu_147477 otu_79662 otu_80901
otu_99796 otu_147285 otu_134718 otu_81308 otu_3922 otu_103211 otu_107924 otu_270
otu_145327 otu_80785 otu_152030 otu_49943 otu_163974 otu_36745 otu_151393
otu_140735 otu_106499 otu_7099 otu_28255 otu_23862 otu_28771 otu_29981 otu_7095
otu_23444 otu_118954 otu_99646 otu_22779 otu_35677 otu_139587 otu_93391
otu_23747 otu_105714 otu_146377 otu_144272 otu_4656 otu_109325 otu_138441
otu_150818 otu_146391 otu_84041 otu_118427 otu_99838 otu_36325 otu_28390
otu_26824 otu_154108 otu_146710 otu_103592 otu_11211 otu_22735 otu_13296
otu_11726 otu_26037 otu_22386 otu_106402 otu_96723 otu_9477 otu_134573 otu_80690
otu_138576 otu_142434 otu_102839 otu_79906 otu_79203 otu_29197 otu_7335
otu_144165 otu_80751 otu_104643 otu_99506 otu_99247 otu_33213 otu_146909
otu_140995 otu_152227 otu_136966 otu_26340 otu_6301 otu_138416 otu_2715
otu_30274 otu_141939 otu_62847 otu_3493 otu_137087 otu_145129 otu_99414
otu_99596 otu_105404 otu_6194 otu_25702 otu_108743 otu_104577 otu_35820
otu_32082 otu_6335 otu_3728 otu_110436 otu_26585 otu_4811 otu_26695 otu_81240
otu_28690 otu_2922 otu_104713 otu_153969 otu_4267 otu_146266 otu_139143
otu_30313 otu_142589 otu_79641 otu_81384 otu_134349 otu_103109 otu_136980
otu_3412 otu_83972 otu_4895 otu_5955 otu_63006 otu_27854 otu_30265 otu_18616

otu_150818 otu_146391 otu_84041 otu_118427 otu_99838 otu_36525 otu_28390
otu_26824 otu_154108 otu_146710 otu_103592 otu_11211 otu_22735 otu_13296
otu_11726 otu_26037 otu_22386 otu_106402 otu_96723 otu_9477 otu_134573 otu_80690
otu_138576 otu_142434 otu_102839 otu_79906 otu_79203 otu_29197 otu_7335
otu_144165 otu_80751 otu_104643 otu_99506 otu_99247 otu_33213 otu_146909
otu_140995 otu_152227 otu_136966 otu_26340 otu_6301 otu_138416 otu_2715
otu_30274 otu_141939 otu_62847 otu_3493 otu_137087 otu_145129 otu_99414
otu_99596 otu_105404 otu_6194 otu_25702 otu_108743 otu_104577 otu_35820
otu_32082 otu_6335 otu_3728 otu_110436 otu_26585 otu_4811 otu_26695 otu_81240
otu_28690 otu_2922 otu_104713 otu_153969 otu_4267 otu_146266 otu_139143
otu_30313 otu_142589 otu_79641 otu_81384 otu_134349 otu_103109 otu_136980
otu_3412 otu_83972 otu_4895 otu_5955 otu_63006 otu_27854 otu_30265 otu_18616
otu_140709 otu_83775 otu_31453 otu_83481 otu_82928 otu_2508 otu_5647 otu_48465
otu_80307 otu_150674 otu_144461 otu_146578 otu_30403 otu_126186 otu_2495
otu_151150 otu_146348 otu_36485 otu_137106 otu_33340 otu_3211 otu_81083 otu_3869
otu_26284 otu_23429 otu_79507 otu_110603 otu_553 otu_80458 otu_126563 otu_79034
otu_108346 otu_105760 otu_7057 otu_140861 otu_30773 otu_3042 otu_6667 otu_146440
otu_105414 otu_2751 otu_32457 otu_28177 otu_141977 otu_23627 otu_648 otu_24686
otu_2156 otu_14054 otu_103419 otu_143970 otu_149938 otu_3089 otu_104347 otu_549
otu_28469 otu_2517 otu_5312 otu_4618 otu_103490 otu_22909 otu_32595 otu_10062
otu_103294 otu_35508 otu_5524 otu_2620 otu_96830 otu_82017 otu_126346 otu_462
otu_64457 otu_97218 otu_106983 otu_31017 otu_3904 otu_2297 otu_81369 otu_26770
otu_133008 otu_2194 otu_30559 otu_108118 otu_3603 otu_149306 otu_32316
otu_140247 otu_144028 otu_32309 otu_35815 otu_24570 otu_803 otu_2699 otu_106471
otu_99528 otu_31902 otu_79254 otu_24543 otu_28321 otu_29256 otu_22889 otu_30723
otu_19170 otu_22436 otu_149434 otu_102942 otu_146407 otu_26178 otu_22899
otu_152484 otu_24752 otu_25242 otu_139699 otu_6080 otu_35584 otu_79643 otu_3949
otu_106068 otu_99612 otu_34022 otu_7134 otu_24252 otu_31170 otu_28531 otu_132443
otu_152112 otu_104780 otu_7261 otu_139645 otu_104212 otu_141817 otu_108810
otu_27429 otu_31572 otu_34651 otu_12173 otu_99487 otu_81331 otu_151008 otu_24572
otu_142471 otu_139839 otu_79521 otu_152235 otu_126634 otu_30851 otu_28266
otu_79014 otu_107767 otu_6501 otu_150178 otu_141828 otu_141086 otu_151442
otu_80693 otu_78949 otu_108418 otu_28796 otu_79383 otu_2806 otu_79033 otu_34231
otu_3456 otu_102853 otu_80950 otu_3710 otu_2523 otu_29598 otu_152632 otu_153287
otu_146259 otu_79172 otu_81386 otu_144342 otu_139407 otu_82357 otu_140785
otu_30103 otu_139723 otu_4667 otu_25990 otu_152579 otu_146468 otu_32027
otu_105754 otu_146544 otu_99340 otu_25859 otu_105907 otu_108283 otu_138438
otu_106700 otu_30568 otu_2131 otu_5672 otu_150259 otu_3154 otu_140424 otu_80753
otu_22986 otu_86852 otu_23384 otu_19332 otu_10622 otu_105394 otu_7182 otu_78979
otu_2700 otu_137611 otu_153872 otu_149210 otu_147231 otu_99366 otu_104641
otu_29026 otu_108783 otu_79655 otu_139190 otu_144334 otu_144853 otu_1241
otu_82530 otu_1063 otu_109867 otu_151043 otu_144200 otu_145099 otu_22440
otu_24995 otu_147140 otu_144135 otu_106562 otu_103472 otu_99356 otu_3393
otu_99903 otu_96949 otu_36781 otu_121392 otu_137854 otu_81147 otu_24696 otu_502
otu_82057 otu_99406 otu_22710 otu_146620 otu_81400 otu_80938 otu_142287
otu_99429 otu_79092 otu_2923 otu_102986 otu_138497 otu_126924 otu_99234 otu_2390
otu_4704 otu_133049 otu_153999 otu_5192 otu_25223 otu_147392 otu_24913 otu_526
otu_99656 otu_104208 otu_4823 otu_5166 otu_6585 otu_36753 otu_143853 otu_28888
otu_103359 otu_35060 otu_140732 otu_109559 otu_2277 otu_108629 otu_107084
otu_99507 otu_1036 otu_6790 otu_153835 otu_34420 otu_27499 otu_153870 otu_1411
otu_28314 otu_30206 otu_31174 otu_99783 otu_106006 otu_115972 otu_143823
otu_147034 otu_99312 otu_137878 otu_154318 otu_141957 otu_139147 otu_146958
otu_9975 otu_149659 otu_108357 otu_105711 otu_6759 otu_127474 otu_28654
otu_26723 otu_126520 otu_2627 otu_6634 otu_23144 otu_3846 otu_23650 otu_153316
otu_106502 otu_399 otu_140690 otu_106577 otu_99263 otu_183 otu_23293 otu_3229
otu_151274 otu_79140 otu_134 otu_3576 otu_24813 otu_150672 otu_109728 otu_22936
otu_6992 otu_25008 otu_27275 otu_103150 otu_152923 otu_108476 otu_29207
otu_106057 otu_117731 otu_2497 otu_146272 otu_154596 otu_6044 otu_133073
otu_25736 otu_153106 otu_1822 otu_28679 otu_139589 otu_20825 otu_150160
otu_103051 otu_44763 otu_80545 otu_11082 otu_151949 otu_79289 otu_132701
otu_80701 otu_25412 otu_133028 otu_152444 otu_104548 otu_2380 otu_134414
otu_1293 otu_139219 otu_25989 otu_146230 otu_110211 otu_29658 otu_137849 otu_4575
otu_1630 otu_79059 otu_109205 otu_25181 otu_139610 otu_26200 otu_100003
otu_34799 otu_108149 otu_25214 otu_153453 otu_81210 otu_138341 otu_151692
otu_34910 otu_2623 otu_108548 otu_133949 otu_2290 otu_255 otu_22378 otu_13922
otu_132907 otu_24817 otu_102975 otu_4342 otu_23684 otu_3166 otu_79861 otu_5585
otu_28924 otu_48582 otu_147257 otu_97000 otu_30654 otu_141147 otu_79131 otu_4089
otu_22759 otu_3655 otu_23073 otu_24253 otu_79504 otu_33373 otu_388 otu_7139
otu_106289 otu_96766 otu_3171 otu_2891 otu_129327 otu_24377 otu_27266 otu_152750
otu_3673 otu_23673 otu_134447 otu_152065 otu_130079 otu_140916 otu_129699
otu_107026 otu_474 otu_28246 otu_134777 otu_100108 otu_154505 otu_23109
otu_30607 otu_79024 otu_106206 otu_96985 otu_27367 otu_5171 otu_100152 otu_81714
otu_134689 otu_17081 otu_105479 otu_150347 otu_96945 otu_3684 otu_24713
otu_150132 otu_80087 otu_27333 otu_150267 otu_7398 otu_108726 otu_80184 otu_6951
otu_579 otu_154023 otu_152988 otu_127217 otu_147247 otu_106837 otu_139930
otu_4238 otu_24333 otu_5491 otu_35928 otu_29646 otu_81802 otu_23001 otu_5017
otu_3020 otu_102926 otu_82926 otu_28514 otu_134402 otu_154660 otu_10517 otu_3303
otu_426 otu_29955 otu_140712 otu_144068 otu_5552 otu_80239 otu_7330 otu_29370
otu_147368 otu_147205 otu_27194 otu_32102 otu_3508 otu_138219 otu_35802
otu_150924 otu_136973 otu_126193 otu_126849 otu_136437 otu_6715 otu_117358
otu_83873 otu_79753 otu_126412 otu_153257 otu_97091 otu_23624 otu_152618
otu_35821 otu_109064 otu_23860 otu_4835 otu_152308 otu_107698 otu_99419
otu_31247 otu_13683 otu_79300 otu_26506 otu_152878 otu_30048 otu_150566
otu_26983 otu_107105 otu_147486 otu_2559 otu_2185 otu_26526 otu_22791 otu_3828
otu_152011 otu_6817 otu_103308 otu_32090 otu_31354 otu_10265 otu_154357
otu_140849 otu_1154 otu_27982 otu_152067 otu_134362 otu_105110 otu_83086
otu_79975 otu_26545 otu_143888 otu_163244 otu_138196 otu_104186 otu_126583
otu_144349 otu_3981 otu_80448 otu_153508 otu_12096 otu_100060 otu_20264
otu_81593 otu_144181 otu_32483 otu_79639 otu_99934 otu_57315 otu_22831
otu_102824 otu_2730 otu_163988 otu_146342 otu_142269 otu_105451 otu_24764
otu_127497 otu_46034 otu_147294 otu_36622 otu_34394 otu_146951 otu_23253
otu_30324 otu_106972 otu_5703 otu_106992 otu_105069 otu_79208 otu_136943
otu_5419 otu_151350 otu_104932 otu_136233 otu_107317 otu_138501 otu_61040
otu_150320 otu_33583 otu_151582 otu_99616 otu_106846 otu_3691 otu_132526
otu_32677 otu_137774 otu_106062 otu_106017 otu_82889 otu_33272 otu_79020
otu_84226 otu_100519 otu_80524 otu_150315 otu_81915 otu_134546 otu_151913
otu_81840 otu_1244 otu_6654 otu_141087 otu_81588 otu_154427 otu_138529 otu_2221
otu_108975 otu_132541 otu_105455 otu_81297 otu_106131 otu_30527 otu_1106
otu_3309 otu_153742 otu_106360 otu_152666 otu_137405 otu_149137 otu_5728
otu_107426 otu_80313 otu_23172 otu_2866 otu_711 otu_79935 otu_147199 otu_2905
otu_146579 otu_110437 otu_4846 otu_13667 otu_153145 otu_31509 otu_44435
otu_106330 otu_129648 otu_5329 otu_127171 otu_2831 otu_4031 otu_136598
otu_142337 otu_143923 otu_89653 otu_30365 otu_151845 otu_105693 otu_29092
otu_79889 otu_146787 otu_79150 otu_96950 otu_5932 otu_148100 otu_105405 otu_3955
otu_154779 otu_33691 otu_97054 otu_79078 otu_104467 otu_153284 otu_107479
otu_4930 otu_26466 otu_103477 otu_100077 otu_147416 otu_16859 otu_106888
otu_136718 otu_6916 otu_98572 otu_22496 otu_147391 otu_139825 otu_675 otu_151343
otu_150625 otu_35425 otu_107420 otu_24366 otu_139900 otu_35629 otu_103099
otu_151662 otu_28143 otu_151637 otu_151446 otu_118737 otu_34029 otu_127830

otu_3309 otu_153742 otu_106360 otu_152666 otu_137405 otu_149137 otu_5728
otu_107426 otu_80313 otu_23172 otu_2866 otu_711 otu_79935 otu_147199 otu_2905
otu_146579 otu_110437 otu_4846 otu_13667 otu_153145 otu_31509 otu_44435
otu_106330 otu_129648 otu_5329 otu_127171 otu_2831 otu_4031 otu_136598
otu_142337 otu_143923 otu_89653 otu_30365 otu_151845 otu_105693 otu_29092
otu_79889 otu_146787 otu_79150 otu_96950 otu_5932 otu_148100 otu_105405 otu_3955
otu_154779 otu_33691 otu_97054 otu_79078 otu_104467 otu_153284 otu_107479
otu_4930 otu_26466 otu_103477 otu_100077 otu_147416 otu_16859 otu_106888
otu_136718 otu_6916 otu_98572 otu_22496 otu_147391 otu_139825 otu_675 otu_151343
otu_150625 otu_35425 otu_107420 otu_24366 otu_139900 otu_35629 otu_103099
otu_151662 otu_28143 otu_151637 otu_151446 otu_118737 otu_34029 otu_127830
otu_81541 otu_12316 otu_150696 otu_99527 otu_79629 otu_1511 otu_132584 otu_23735
otu_144364 otu_24302 otu_153718 otu_90219 otu_108628 otu_31494 otu_150756
otu_98757 otu_147436 otu_109655 otu_7011 otu_150256 otu_109910 otu_79109
otu_25626 otu_3360 otu_22387 otu_2784 otu_7135 otu_132489 otu_4447 otu_23475
otu_127326 otu_80892 otu_23391 otu_26944 otu_143900 otu_23695 otu_136758
otu_134451 otu_1206 otu_146840 otu_148586 otu_149859 otu_2111 otu_99363
otu_143988 otu_151731 otu_118086 otu_22939 otu_138165 otu_139560 otu_156886
otu_22673 otu_105813 otu_107206 otu_105885 otu_150114 otu_150180 otu_25152
otu_106620 otu_150179 otu_24704 otu_29872 otu_48755 otu_109158 otu_1414
otu_99353 otu_110163 otu_13632 otu_146539 otu_107329 otu_7283 otu_26645
otu_137623 otu_35980 otu_108205 otu_150354 otu_110043 otu_126971 otu_5025
otu_4020 otu_143869 otu_150413 otu_3868 otu_12201 otu_96854 otu_32807 otu_153831
otu_3125 otu_30180 otu_132642 otu_34724 otu_99260 otu_109260 otu_137253
otu_99351 otu_23987 otu_80598 otu_26341 otu_22709 otu_143820 otu_126558
otu_81934 otu_27494 otu_32077 otu_31548 otu_142063 otu_126675 otu_31620
otu_30829 otu_150546 otu_2675 otu_37206 otu_105531 otu_27732 otu_23861 otu_26067
otu_256 otu_142030 otu_5788 otu_143927 otu_132511 otu_97039 otu_24264 otu_80456
otu_137225 otu_134328 otu_119792 otu_32744 otu_106478 otu_2853 otu_24725
otu_7197 otu_138432 otu_151594 otu_153086 otu_106490 otu_79658 otu_25023
otu_146960 otu_141074 otu_27540 otu_138190 otu_2731 otu_146319 otu_25879
otu_34518 otu_5185 otu_96838 otu_28381 otu_640 otu_152856 otu_512 otu_126467
otu_126317 otu_106570 otu_25654 otu_126309 otu_106207 otu_106585 otu_147212
otu_81030 otu_146761 otu_139213 otu_24853 otu_25340 otu_147251 otu_30767
otu_2976 otu_104508 otu_87941 otu_22563 otu_102280 otu_24450 otu_777 otu_81091
otu_541 otu_146590 otu_153293 otu_146369 otu_126237 otu_152626 otu_150278
otu_414 otu_146775 otu_4154 otu_27133 otu_25165 otu_103022 otu_127991 otu_22817
otu_48713 otu_103510 otu_32245 otu_143889 otu_81334 otu_105875 otu_81219
otu_79185 otu_150982 otu_3059 otu_479 otu_154413 otu_4473 otu_99275 otu_133131
otu_31660 otu_83448 otu_764 otu_27059 otu_6209 otu_99254 otu_99306 otu_25497
otu_79027 otu_5253 otu_147903 otu_79734 otu_151115 otu_80921 otu_102889 otu_25934
otu_25942 otu_83767 otu_107541 otu_99269 otu_101088 otu_82615 otu_106711
otu_99573 otu_99885 otu_79581 otu_105444 otu_126608 otu_6547 otu_144117
otu_91922 otu_23898 otu_34247 otu_81598 otu_105722 otu_103430 otu_46874
otu_147447 otu_80697 otu_23548 otu_2998 otu_83743 otu_147388 otu_143985 otu_2761
otu_106911 otu_153348 otu_79431 otu_29116 otu_152852 otu_138469 otu_23577
otu_45512 otu_5109 otu_27427 otu_78999 otu_132508 otu_147293 otu_65209 otu_2465
otu_152972 otu_22401 otu_81664 otu_142229 otu_129619 otu_53516 otu_25902
otu_139182 otu_106387 otu_28370 otu_2420 otu_79745 otu_109976 otu_47673
otu_141941 otu_147125 otu_32480 otu_107593 otu_24247 otu_106863 otu_102939
otu_5178 otu_26039 otu_102827 otu_103203 otu_81447 otu_23617 otu_100116 otu_5790
otu_99241 otu_79729 otu_146633 otu_152161 otu_134397 otu_79486 otu_126906
otu_163247 otu_31000 otu_140978 otu_4853 otu_103412 otu_147326 otu_27769
otu_99618 otu_147401 otu_2395 otu_35917 otu_27453 otu_154842 otu_110364
otu_104392 otu_153491 otu_9527 otu_152906 otu_152279 otu_81480 otu_37143
otu_79476 otu_26867 otu_81708 otu_1802 otu_24947 otu_3677 otu_99649 otu_99944
otu_6349 otu_108684 otu_142391 otu_107857 otu_83135 otu_81475 otu_79157
otu_84138 otu_23051 otu_23775 otu_31653 otu_146 otu_147001 otu_150102 otu_103546
otu_153227 otu_96946 otu_2892 otu_105953 otu_4080 otu_151013 otu_137798
otu_29156 otu_6595 otu_137692 otu_146962 otu_79721 otu_141566 otu_136061
otu_79091 otu_35333 otu_79976 otu_80920 otu_79551 otu_139770 otu_106665
otu_136355 otu_137826 otu_81764 otu_6316 otu_81776 otu_30088 otu_78950 otu_22475
otu_106119 otu_144508 otu_103126 otu_106814 otu_2925 otu_9993 otu_107796
otu_105712 otu_79766 otu_132886 otu_98909 otu_82107 otu_150483 otu_6192
otu_28133 otu_150692 otu_30430 otu_25058 otu_126410 otu_6406 otu_99251
otu_157859 otu_99442 otu_126733 otu_163769 otu_108889 otu_148976 otu_152420
otu_28187 otu_80694 otu_126915 otu_31857 otu_147427 otu_134582 otu_141429
otu_34062 otu_140959 otu_105950 otu_27531 otu_3228 otu_139146 otu_4322
otu_107370 otu_127156 otu_139334 otu_3699 otu_126294 otu_150978 otu_80371
otu_31027 otu_99804 otu_55973 otu_99626 otu_89912 otu_141820 otu_138429
otu_163989 otu_23780 otu_145071 otu_146779 otu_150366 otu_25079 otu_171 otu_7342
otu_150638 otu_22665 otu_79744 otu_102821 otu_24243 otu_3967 otu_147363
otu_27741 otu_140475 otu_142535 otu_80835 otu_25014 otu_53680 otu_144529
otu_129278 otu_96773 otu_137803 otu_82815 otu_108706 otu_119252 otu_2865
otu_28955 otu_152751 otu_33470 otu_7388 otu_18741 otu_129696 otu_34650 otu_89224
otu_139549 otu_79130 otu_80092 otu_5273 otu_146202 otu_79032 otu_152702
otu_146276 otu_96763 otu_501 otu_1559 otu_3312 otu_13530 otu_106260 otu_24035
otu_140966 otu_108161 otu_108853 otu_34326 otu_146843 otu_146651 otu_97902
otu_160720 otu_2693 otu_2489 otu_3617 otu_153504 otu_2124 otu_151181 otu_110195
otu_136058 otu_107928 otu_4044 otu_4487 otu_134725 otu_132824 otu_4462
otu_127606 otu_83963 otu_3249 otu_127466 otu_48439 otu_2296 otu_34155 otu_154083
otu_82312 otu_126931 otu_103067 otu_103241 otu_29569 otu_142094 otu_24279
otu_138542 otu_127527 otu_97514 otu_24863 otu_136068 otu_23105 otu_24582
otu_110565 otu_28982 otu_103464 otu_2379 otu_142516 otu_3330 otu_138159
otu_103034 otu_151176 otu_87004 otu_146928 otu_54606 otu_25931 otu_146690
otu_26167 otu_105607 otu_151621 otu_139856 otu_109902 otu_31143 otu_150186
otu_35502 otu_105770 otu_22923 otu_99730 otu_107637 otu_99989 otu_134502
otu_138461 otu_63697 otu_148022 otu_27242 otu_132843 otu_29763 otu_22812
otu_2471 otu_36627 otu_145209 otu_144203 otu_109999 otu_440 otu_126900 otu_25531
otu_26467 otu_151121 otu_469 otu_97221 otu_79654 otu_97865 otu_152973 otu_79993
otu_83076 otu_146839 otu_139561 otu_82082 otu_102807 otu_16601 otu_99233
otu_22324 otu_14807 otu_105575 otu_6639 otu_105133 otu_30018 otu_150351
otu_82562 otu_153319 otu_78957 otu_126911 otu_106689 otu_82350 otu_49677
otu_23645 otu_3382 otu_2394 otu_30734 otu_126500 otu_102834 otu_5813 otu_24700
otu_80333 otu_24031 otu_110094 otu_147160 otu_23407 otu_104170 otu_146626
otu_3407 otu_6690 otu_53663 otu_54048 otu_3720 otu_103594 otu_139200 otu_138420
otu_146747 otu_122611 otu_106339 otu_140810 otu_108954 otu_83494 otu_127797
otu_80030 otu_36644 otu_99523 otu_142057 otu_149675 otu_82905 otu_81519
otu_27077 otu_11114 otu_2766 otu_6459 otu_18325 otu_2321 otu_105558 otu_1356
otu_99450 otu_80620 otu_3050 otu_26411 otu_106901 otu_106820 otu_100079
otu_36144 otu_99338 otu_126224 otu_146341 otu_104478 otu_18936 otu_96999
otu_33246 otu_3296 otu_2966 otu_105739 otu_82782 otu_136084 otu_98219 otu_150557
otu_139888 otu_99800 otu_33819 otu_36302 otu_151277 otu_126887 otu_82865
otu_138121 otu_152184 otu_99580 otu_146661 otu_583 otu_2105 otu_35648 otu_34673
otu_25632 otu_132765 otu_99357 otu_82678 otu_266 otu_99591 otu_109481 otu_24457
otu_1372 otu_107956 otu_136067 otu_24790 otu_80136 otu_25704 otu_31602 otu_33721
otu_3819 otu_742 otu_420 otu_134613 otu_3961 otu_3627 otu_28454 otu_106203
otu_27263 otu_33143 otu_106813 otu_103608 otu_25390 otu_132707 otu_145408
otu_81503 otu_127443 otu_134512 otu_151309 otu_81736 otu_2455 otu_147319

otu_80030 otu_36644 otu_99523 otu_142057 otu_149675 otu_82905 otu_81519
otu_27077 otu_11114 otu_2766 otu_6459 otu_18325 otu_2321 otu_105558 otu_1356
otu_99450 otu_80620 otu_3050 otu_26411 otu_106901 otu_106820 otu_100079
otu_36144 otu_99338 otu_126224 otu_146341 otu_104478 otu_18936 otu_96999
otu_33246 otu_3296 otu_2966 otu_105739 otu_82782 otu_136084 otu_98219 otu_150557
otu_139888 otu_99800 otu_33819 otu_36302 otu_151277 otu_126887 otu_82865
otu_138121 otu_152184 otu_99580 otu_146661 otu_583 otu_2105 otu_35648 otu_34673
otu_25632 otu_132765 otu_99357 otu_82678 otu_266 otu_99591 otu_109481 otu_24457
otu_1372 otu_107956 otu_136067 otu_24790 otu_80136 otu_25704 otu_31602 otu_33721
otu_3819 otu_742 otu_420 otu_134613 otu_3961 otu_3627 otu_28454 otu_106203
otu_27263 otu_33143 otu_106813 otu_103608 otu_25390 otu_132707 otu_145408
otu_81503 otu_127443 otu_134512 otu_151309 otu_81736 otu_2455 otu_147319
otu_82610 otu_149976 otu_138498 otu_6543 otu_131554 otu_143777 otu_30852
otu_79836 otu_147299 otu_104235 otu_5350 otu_47923 otu_127523 otu_137261
otu_28200 otu_46395 otu_27056 otu_29382 otu_247 otu_3322 otu_146410 otu_89335
otu_23430 otu_2896 otu_154764 otu_79292 otu_151936 otu_153073 otu_81996 otu_6818
otu_79390 otu_152145 otu_83091 otu_2639 otu_797 otu_136748 otu_2128 otu_141145
otu_153303 otu_142110 otu_1276 otu_105171 otu_137483 otu_138500 otu_138549
otu_2263 otu_134715 otu_142376 otu_19672 otu_146922 otu_126355 otu_2658
otu_105393 otu_82571 otu_97695 otu_33766 otu_10617 otu_98705 otu_6798 otu_7249
otu_494 otu_106093 otu_2437 otu_81589 otu_35645 otu_6048 otu_79116 otu_25501
otu_10417 otu_134762 otu_102498 otu_146819 otu_126759 otu_34178 otu_36734
otu_106561 otu_33316 otu_106701 otu_79366 otu_48392 otu_1247 otu_79302
otu_140841 otu_4944 otu_146733 otu_4066 otu_2138 otu_79931 otu_5714 otu_151335
otu_143809 otu_103298 otu_5667 otu_81180 otu_4801 otu_146463 otu_150583 otu_3701
otu_291 otu_5715 otu_25994 otu_98336 otu_99991 otu_148056 otu_3531 otu_31536
otu_81011 otu_32407 otu_106628 otu_147185 otu_140827 otu_26972 otu_152646
otu_140771 otu_31418 otu_100001 otu_82560 otu_23148 otu_105977 otu_129659
otu_103102 otu_146649 otu_154898 otu_150940 otu_151271 otu_79634 otu_3366
otu_127153 otu_83942 otu_81416 otu_146580 otu_14914 otu_144881 otu_4635
otu_23555 otu_27400 otu_27621 otu_84019 otu_138608 otu_139306 otu_31616
otu_27578 otu_3357 otu_6496 otu_150108 otu_4654 otu_103153 otu_140721 otu_33633
otu_134459 otu_99629 otu_97034 otu_694 otu_132784 otu_145162 otu_141486
otu_36878 otu_98134 otu_2113 otu_2373 otu_132544 otu_4818 otu_530 otu_109030
otu_82221 otu_150943 otu_79961 otu_100623 otu_127035 otu_107613 otu_5727
otu_96821 otu_10538 otu_142513 otu_104188 otu_33523 otu_109067 otu_154315
otu_3250 otu_150383 otu_126727 otu_126417 otu_142600 otu_2406 otu_703 otu_150282
otu_34346 otu_33883 otu_109772 otu_25121 otu_32648 otu_132718 otu_34200
otu_36807 otu_106116 otu_57434 otu_109083 otu_80165 otu_36381 otu_152302
otu_3733 otu_107608 otu_152745 otu_109548 otu_6223 otu_79767 otu_126723
otu_128003 otu_127562 otu_108965 otu_139150 otu_106426 otu_100085 otu_23418
otu_36045 otu_28384 otu_100154 otu_146465 otu_126797 otu_122453 otu_33334
otu_30347 otu_6177 otu_28482 otu_103341 otu_138978 otu_142409 otu_30877
otu_99833 otu_3906 otu_107016 otu_153070 otu_3584 otu_47839 otu_154300
otu_150073 otu_2758 otu_6096 otu_107772 otu_144152 otu_26639 otu_2506 otu_108020
otu_162873 otu_31221 otu_23583 otu_134471 otu_2136 otu_81498 otu_103027
otu_152273 otu_99907 otu_104672 otu_146567 otu_150736 otu_80685 otu_555
otu_79237 otu_29668 otu_108728 otu_152993 otu_26915 otu_106876 otu_134750
otu_99925 otu_28413 otu_23446 otu_103329 otu_35189 otu_116622 otu_21836
otu_100119 otu_3162 otu_136098 otu_3601 otu_79135 otu_35405 otu_32912 otu_4682
otu_99597 otu_722 otu_1088 otu_26927 otu_82019 otu_25213 otu_152025 otu_2928
otu_132563 otu_23671 otu_139601 otu_25809 otu_26800 otu_79516 otu_109579
otu_99384 otu_152627 otu_79502 otu_97068 otu_97618 otu_154612 otu_26224
otu_154794 otu_146499 otu_33113 otu_107278 otu_139221 otu_83429 otu_25155
otu_36789 otu_108910 otu_29925 otu_103235 otu_134417 otu_138448 otu_99396
otu_5763 otu_586 otu_136737 otu_31999 otu_6794 otu_6732 otu_150136 otu_80217
otu_137874 otu_152806 otu_150706 otu_148651 otu_83744 otu_146371 otu_83656
otu_154191 otu_154099 otu_103115 otu_2767 otu_147417 otu_23848 otu_79371
otu_6220 otu_151353 otu_1448 otu_134515 otu_2608 otu_30739 otu_132667 otu_136240
otu_96828 otu_1175 otu_146947 otu_151068 otu_1097 otu_480 otu_126987 otu_103131
otu_31275 otu_99941 otu_154784 otu_103322 otu_34715 otu_106223 otu_139672
otu_126505 otu_161397 otu_126980 otu_79345 otu_23123 otu_106809 otu_83879
otu_25336 otu_127030 otu_81288 otu_110461 otu_137071 otu_22341 otu_25049
otu_79968 otu_106248 otu_108105 otu_145124 otu_146554 otu_35367 otu_137622
otu_153056 otu_27000 otu_102876 otu_146516 otu_35715 otu_5150 otu_2328
otu_127223 otu_5165 otu_2140 otu_107562 otu_145006 otu_106410 otu_22708
otu_153836 otu_147000 otu_4244 otu_127697 otu_108287 otu_107879 otu_126497
otu_144269 otu_107259 otu_32568 otu_4423 otu_80204 otu_30060 otu_23314 otu_31788
otu_792 otu_4535 otu_6183 otu_2776 otu_4401 otu_151192 otu_143968 otu_106794
otu_25016 otu_115868 otu_148981 otu_107859 otu_33518 otu_151573 otu_152511
otu_146535 otu_14424 otu_146415 otu_106043 otu_6000 otu_78972 otu_22414
otu_154443 otu_30215 otu_10466 otu_23214 otu_99849 otu_119709 otu_45 otu_142028
otu_150512 otu_29027 otu_2458 otu_154140 otu_24127 otu_28265 otu_149034 otu_4573
otu_110625 otu_3074 otu_127031 otu_2580 otu_2744 otu_126251 otu_3505 otu_12272
otu_3021 otu_81725 otu_2634 otu_126820 otu_107039 otu_137291 otu_110531 otu_4793
otu_25 otu_47858 otu_49133 otu_96815 otu_138480 otu_27487 otu_25380 otu_141069
otu_6333 otu_6892 otu_29683 otu_103223 otu_26399 otu_132769 otu_6106 otu_23765
otu_108854 otu_31037 otu_23363 otu_28909 otu_3196 otu_144242 otu_28163
otu_118734 otu_152375 otu_126599 otu_107880 otu_109809 otu_24888 otu_9632
otu_108993 otu_31382 otu_127071 otu_132573 otu_147316 otu_99699 otu_23667
otu_129669 otu_146250 otu_105235 otu_4323 otu_6776 otu_147986 otu_5730 otu_23731
otu_79271 otu_107030 otu_147942 otu_24754 otu_432 otu_103157 otu_35520
otu_147296 otu_79061 otu_109042 otu_36693 otu_152604 otu_81433 otu_139631
otu_144102 otu_99794 otu_24526 otu_6546 otu_7333 otu_79119 otu_36087 otu_23567
otu_79582 otu_23433 otu_3954 otu_107549 otu_147059 otu_82862 otu_26546 otu_23435
otu_143964 otu_80116 otu_6242 otu_151765 otu_103195 otu_144541 otu_96933
otu_23441 otu_64180 otu_26830 otu_103559 otu_96836 otu_134343 otu_15372
otu_164054 otu_107057 otu_25656 otu_29827 otu_23107 otu_139744 otu_36904
otu_36474 otu_146409 otu_152523 otu_79737 otu_99249 otu_79192 otu_154550
otu_150247 otu_100727 otu_24391 otu_107948 otu_298 otu_79893 otu_30472
otu_118805 otu_79082 otu_144324 otu_24537 otu_26976 otu_3645 otu_147451
otu_106750 otu_36859 otu_25880 otu_30551 otu_3920 otu_5335 otu_82866 otu_80266
otu_136099 otu_22901 otu_2262 otu_99897 otu_23064 otu_80400 otu_31140 otu_34617
otu_30824 otu_106364 otu_80674 otu_134391 otu_146247 otu_147079 otu_2978
otu_1213 otu_144235 otu_99987 otu_126307 otu_17676 otu_22956 otu_83489
otu_141097 otu_80532 otu_5559 otu_126536 otu_109439 otu_604 otu_33249 otu_163286
otu_6016 otu_150388 otu_150574 otu_143392 otu_99887 otu_4004 otu_29115 otu_3424
otu_26137 otu_4358 otu_2340 otu_105701 otu_99226 otu_126738 otu_29060 otu_83661
otu_23931 otu_28508 otu_146286 otu_132849 otu_99844 otu_79925 otu_28069
otu_96987 otu_153093 otu_3991 otu_103116 otu_108436 otu_25326 otu_126375
otu_28344 otu_82796 otu_99511 otu_25884 otu_29426 otu_28991 otu_2254 otu_577
otu_107782 otu_2988 otu_141985 otu_132658 otu_138299 otu_151587 otu_139623
otu_79012 otu_144027 otu_80659 otu_25801 otu_4213 otu_107277 otu_107891
otu_26868 otu_80928 otu_882 otu_28520 otu_142143 otu_24654 otu_35828 otu_22717
otu_82810 otu_3675 otu_25295 otu_96717 otu_149983 otu_22823 otu_138444
otu_141478 otu_126394 otu_3264 otu_2139 otu_105944 otu_23489 otu_146299
otu_142428 otu_136090 otu_83649 otu_133124 otu_35742 otu_23497 otu_139643
otu_96700 otu_82603 otu_11026 otu_2169 otu_99704 otu_150390 otu_138499
otu_126741 otu_129516 otu_150469 otu_151527 otu_22603 otu_80053 otu_152685

[illegible]
otu_23931 otu_28508 otu_146286 otu_132849 otu_99844 otu_79925 otu_28069
otu_96987 otu_153093 otu_3991 otu_103116 otu_108436 otu_25326 otu_126375
otu_28344 otu_82796 otu_99511 otu_25884 otu_29426 otu_28991 otu_2254 otu_577
otu_107782 otu_2988 otu_141985 otu_132658 otu_138299 otu_151587 otu_139623
otu_79012 otu_144027 otu_80659 otu_25801 otu_4213 otu_107277 otu_107891
otu_26868 otu_80928 otu_882 otu_28520 otu_142143 otu_24654 otu_35828 otu_22717
otu_82810 otu_3675 otu_25295 otu_96717 otu_149983 otu_22823 otu_138444
otu_141478 otu_126394 otu_3264 otu_2139 otu_105944 otu_23489 otu_146299
otu_142428 otu_136090 otu_83649 otu_133124 otu_35742 otu_23497 otu_139643
otu_96700 otu_82603 otu_11026 otu_2169 otu_99704 otu_150390 otu_138499
otu_126741 otu_129516 otu_150469 otu_151527 otu_22603 otu_80053 otu_152685
otu_147410 otu_4110 otu_37037 otu_139860 otu_33247 otu_24088 otu_140728
otu_127350 otu_25971 otu_152998 otu_80744 otu_23847 otu_126216 otu_134473
otu_147370 otu_134419 otu_3696 otu_29634 otu_97498 otu_147431 otu_9525 otu_51615
otu_132620 otu_132905 otu_31985 otu_138126 otu_80379 otu_4913 otu_32856
otu_32067 otu_146533 otu_30594 otu_126592 otu_164080 otu_29140 otu_99687
otu_28264 otu_108617 otu_139837 otu_134450 otu_82253 otu_28395 otu_6800
otu_81347 otu_30497 otu_27634 otu_127616 otu_82989 otu_3925 otu_5034 otu_144022
otu_439 otu_2711 otu_15631 otu_146257 otu_105689 otu_34160 otu_117256 otu_58872
otu_146914 otu_23992 otu_5435 otu_80535 otu_109094 otu_31558 otu_2676 otu_105824
otu_140693 otu_146728 otu_137269 otu_135231 otu_24821 otu_126220 otu_132919
otu_25857 otu_138515 otu_147278 otu_1038 otu_23888 otu_134594 otu_79160
otu_134664 otu_163318 otu_23266 otu_150678 otu_4733 otu_4837 otu_79380
otu_143955 otu_23465 otu_147310 otu_36068 otu_126261 otu_29615 otu_29817
otu_148446 otu_145503 otu_151710 otu_148724 otu_109213 otu_146279 otu_99535
otu_99669 otu_153109 otu_106325 otu_81511 otu_108539 otu_96790 otu_2916
otu_152607 otu_163223 otu_151664 otu_107273 otu_23971 otu_127137 otu_28122
otu_136761 otu_29009 otu_26280 otu_13264 otu_152809 otu_151369 otu_110442
otu_26675 otu_109496 otu_3356 otu_137823 otu_6097 otu_150056 otu_30084
otu_150209 otu_150962 otu_34381 otu_146477 otu_1164 otu_153127 otu_26835
otu_80849 otu_22593 otu_151483 otu_2804 otu_138527 otu_80122 otu_24743 otu_6793
otu_134321 otu_5163 otu_107119 otu_5799 otu_99295 otu_147147 otu_35678 otu_29551
otu_35408 otu_618 otu_32952 otu_150890 otu_2564 otu_152641 otu_32661 otu_154843
otu_5397 otu_31430 otu_98535 otu_80978 otu_79587 otu_108129 otu_150453
otu_108643 otu_26197 otu_136872 otu_2526 otu_594 otu_2246 otu_83459 otu_105727
otu_83063 otu_31109 otu_106278 otu_3445 otu_23345 otu_412 otu_37069 otu_139165
otu_79110 otu_83357 otu_106598 otu_79552 otu_99566 otu_9736 otu_83412 otu_80319
otu_133580 otu_144036 otu_81831 otu_83641 otu_99320 otu_138456 otu_98937
otu_6631 otu_102874 otu_35169 otu_149542 otu_3553 otu_150797 otu_26277
otu_130843 otu_24859 otu_138410 otu_26012 otu_146282 otu_2234 otu_25365
otu_150473 otu_132808 otu_4420 otu_108611 otu_100117 otu_129507 otu_127786
otu_14238 otu_144107 otu_81539 otu_127205 otu_23327 otu_5862 otu_154468 otu_9213
otu_152695 otu_145161 otu_30407 otu_134569 otu_146596 otu_79630 otu_32984
otu_144260 otu_137303 otu_83143 otu_34138 otu_96936 otu_3568 otu_139740
otu_164053 otu_84116 otu_32017 otu_2425 otu_127906 otu_80174 otu_142601
otu_163354 otu_146400 otu_154375 otu_150275 otu_783 otu_79128 otu_79446
otu_153997 otu_83802 otu_79962 otu_6436 otu_27305 otu_23337 otu_141058 otu_80769
otu_81150 otu_30580 otu_104541 otu_156781 otu_31496 otu_80628 otu_139707
otu_99729 otu_83840 otu_563 otu_152934 otu_11541 otu_106055 otu_5027 otu_97002
otu_142668 otu_30589 otu_67943 otu_146350 otu_31993 otu_22483 otu_139155
otu_79362 otu_3737 otu_31409 otu_96866 otu_29373 otu_138192 otu_28 otu_146846
otu_127219 otu_5107 otu_127809 otu_2897 otu_81750 otu_147203 otu_134773
otu_25096 otu_146339 otu_130386 otu_126232 otu_79055 otu_31734 otu_6105
otu_131352 otu_29429 otu_143919 otu_35800 otu_23213 otu_26565 otu_100044
otu_2166 otu_152453 otu_146472 otu_51156 otu_79896 otu_81088 otu_79226
otu_152810 otu_142597 otu_141994 otu_7199 otu_3041 otu_138460 otu_139580
otu_108349 otu_82407 otu_103484 otu_136983 otu_81769 otu_147029 otu_127099
otu_132638 otu_29782 otu_4128 otu_2309 otu_6302 otu_30556 otu_35796 otu_193
otu_143647 otu_5278 otu_57251 otu_105442 otu_26077 otu_126235 otu_99785
otu_106630 otu_32919 otu_148472 otu_142580 otu_152031 otu_30765 otu_151919
otu_126697 otu_4577 otu_146236 otu_841 otu_33205 otu_149186 otu_139609 otu_22849
otu_150984 otu_102843 otu_68585 otu_1552 otu_2848 otu_79779 otu_62395 otu_22900
otu_23075 otu_152099 otu_137047 otu_134441 otu_22415 otu_3057 otu_148502
otu_15034 otu_31577 otu_145326 otu_30247 otu_2457 otu_141943 otu_108108
otu_150100 otu_152938 otu_99443 otu_24240 otu_99996 otu_99390 otu_28298
otu_106716 otu_2600 otu_151253 otu_6187 otu_82979 otu_150269 otu_4289 otu_109308
otu_150831 otu_25318 otu_80203 otu_142575 otu_27784 otu_110281 otu_1121
otu_138478 otu_104994 otu_28513 otu_122861 otu_3672 otu_88710 otu_108773
otu_27226 otu_141846 otu_2144 otu_107129 otu_102989 otu_146300 otu_150705
otu_33740 otu_152064 otu_81302 otu_152045 otu_82872 otu_80074 otu_137801
otu_97154 otu_34361 otu_80851 otu_23134 otu_132506 otu_56944 otu_140825
otu_23101 otu_5584 otu_147048 otu_80540 otu_105126 otu_99627 otu_33562
otu_141054 otu_154430 otu_99970 otu_5811 otu_79683 otu_23579 otu_105670
otu_87785 otu_60759 otu_132657 otu_150788 otu_33014 otu_29157 otu_108089
otu_11882 otu_25159 otu_126271 otu_98151 otu_139743 otu_103162 otu_1421
otu_146354 otu_150417 otu_23720 otu_79471 otu_3258 otu_2763 otu_80876 otu_109539
otu_152284 otu_30391 otu_83645 otu_79586 otu_4507 otu_51966 otu_31538 otu_142121
otu_151740 otu_134637 otu_138306 otu_153412 otu_26716 otu_22861 otu_82797
otu_2684 otu_81863 otu_30262 otu_27939 otu_97111 otu_146869 otu_137873 otu_12669
otu_1539 otu_150589 otu_2613 otu_141937 otu_46324 otu_51473 otu_18110 otu_32653
otu_105019 otu_35030 otu_163760 otu_100031 otu_30178 otu_31543 otu_153383
otu_5194 otu_153624 otu_151012 otu_99714 otu_7256 otu_1957 otu_2507 otu_84102
otu_83025 otu_3592 otu_24192 otu_105868 otu_137105 otu_23531 otu_79427
otu_146332 otu_80367 otu_97047 otu_127778 otu_23833 otu_22524 otu_142047
otu_104572 otu_33698 otu_109875 otu_146502 otu_22578 otu_80016 otu_151625
otu_516 otu_109515 otu_102896 otu_83458 otu_22844 otu_2960 otu_81490 otu_146432
otu_109099 otu_96707 otu_34299 otu_126259 otu_12732 otu_138544 otu_30100
otu_81882 otu_82214 otu_2335 otu_106362 otu_430 otu_80289 otu_33499 otu_35121
otu_151819 otu_126425 otu_79565 otu_2628 otu_6976 otu_137618 otu_154148
otu_83224 otu_79835 otu_4716 otu_6318 otu_138533 otu_108525 otu_132675
otu_109568 otu_134420 otu_150935 otu_80152 otu_79425 otu_150422 otu_99875
otu_106657 otu_4398 otu_22924 otu_90856 otu_127943 otu_99914 otu_106541
otu_22590 otu_31961 otu_10328 otu_4131 otu_100664 otu_3140 otu_5595 otu_104296
otu_83207 otu_139831 otu_82845 otu_154363 otu_4876 otu_134616 otu_26262
otu_83969 otu_107534 otu_24591 otu_3628 otu_4606 otu_36837 otu_22498 otu_105798
otu_24746 otu_84042 otu_134710 otu_149974 otu_22451 otu_128013 otu_97199
otu_153474 otu_25918 otu_23552 otu_151218 otu_25993 otu_81265 otu_131224
otu_34861 otu_127989 otu_143950 otu_126276 otu_2518 otu_132727 otu_4481 otu_5942
otu_127210 otu_144318 otu_60295 otu_29069 otu_6343 otu_503 otu_150845 otu_153770
otu_152860 otu_141787 otu_139678 otu_25410 otu_146492 otu_30784 otu_143848
otu_11126 otu_105897 otu_30087 otu_143836 otu_107801 otu_150153 otu_126293
otu_80707 otu_138566 otu_1052 otu_446 otu_2148 otu_108791 otu_103176 otu_80730
otu_421 otu_24934 otu_99529 otu_138575 otu_28221 otu_101134 otu_105862 otu_35053
otu_141798 otu_154704 otu_133651 otu_2691 otu_99515 otu_33088 otu_142514
otu_106253 otu_99722 otu_1783 otu_146387 otu_153204 otu_137553 otu_147063
otu_32668 otu_132623 otu_136514 otu_24384 otu_25246 otu_3351 otu_31392 otu_33614
otu_2800 otu_7133 otu_141 otu_4440 otu_154812 otu_9830 otu_79859 otu_151363
otu_24622 otu_25089 otu_25364 otu_28071 otu_99505 otu_107256 otu_14055 otu_3448
[illegible]

otu_34861 otu_127989 otu_143950 otu_126276 otu_2518 otu_132727 otu_4481 otu_5942
otu_127210 otu_144318 otu_60295 otu_29069 otu_6343 otu_503 otu_150845 otu_153770
otu_152860 otu_141787 otu_139678 otu_25410 otu_146492 otu_30784 otu_143848
otu_11126 otu_105897 otu_30087 otu_143836 otu_107801 otu_150153 otu_126293
otu_80707 otu_138566 otu_1052 otu_446 otu_2148 otu_108791 otu_103176 otu_80730
otu_421 otu_24934 otu_99529 otu_138575 otu_28221 otu_101134 otu_105862 otu_35053
otu_141798 otu_154704 otu_133651 otu_2691 otu_99515 otu_33088 otu_142514
otu_106253 otu_99722 otu_1783 otu_146387 otu_153204 otu_137553 otu_147063
otu_32668 otu_132623 otu_136514 otu_24384 otu_25246 otu_3351 otu_31392 otu_33614
otu_2800 otu_7133 otu_141 otu_4440 otu_154812 otu_9830 otu_79859 otu_151363
otu_24622 otu_25089 otu_25364 otu_28071 otu_99505 otu_107256 otu_14055 otu_3448
otu_7214 otu_99952 otu_79495 otu_143822 otu_84000 otu_2716 otu_29895 otu_139232
otu_35006 otu_153064 otu_28659 otu_108013 otu_1766 otu_146458 otu_147881
otu_4252 otu_140415 otu_151147 otu_30404 otu_26034 otu_5798 otu_139175 otu_81378
otu_127395 otu_109978 otu_23725 otu_136074 otu_138719 otu_18067 otu_1337
otu_23406 otu_2401 otu_25062 otu_16191 otu_79268 otu_79377 otu_149481 otu_139706
otu_4087 otu_81249 otu_87267 otu_146358 otu_1612 otu_134312 otu_5193 otu_149127
otu_110450 otu_107783 otu_26878 otu_109442 otu_105901 otu_6756 otu_150911
otu_35872 otu_103019 otu_81105 otu_105289 otu_2954 otu_31901 otu_137123
otu_107343 otu_30193 otu_150154 otu_4961 otu_3425 otu_81551 otu_142037 otu_52920
otu_146518 otu_23909 otu_13469 otu_23643 otu_102793 otu_134337 otu_141462
otu_99955 otu_32633 otu_3421 otu_80126 otu_35960 otu_5671 otu_105516 otu_23114
otu_149692 otu_99264 otu_91036 otu_99436 otu_147237 otu_3129 otu_26656
otu_126889 otu_31607 otu_137242 otu_146997 otu_106256 otu_147934 otu_154038
otu_158068 otu_79905 otu_151459 otu_4659 otu_146782 otu_6586 otu_6929 otu_106617
otu_37134 otu_135198 otu_103149 otu_32532 otu_5093 otu_143855 otu_6041
otu_146471 otu_142438 otu_154724 otu_82650 otu_6266 otu_101971 otu_153746
otu_103237 otu_143982 otu_78961 otu_3203 otu_134703 otu_2768 otu_150932
otu_36139 otu_2146 otu_150995 otu_27188 otu_127871 otu_143846 otu_137287
otu_29411 otu_79141 otu_142378 otu_140731 otu_27901 otu_23539 otu_83988
otu_33641 otu_86500 otu_139185 otu_33680 otu_105969 otu_84095 otu_99534 otu_4777
otu_25579 otu_4715 otu_150465 otu_126257 otu_26540 otu_60837 otu_141818
otu_137263 otu_7034 otu_34374 otu_110522 otu_135023 otu_5212 otu_141032
otu_158963 otu_2190 otu_23432 otu_3405 otu_3876 otu_24528 otu_99672 otu_102825
otu_82354 otu_81738 otu_99937 otu_11116 otu_144513 otu_132576 otu_79277
otu_146727 otu_2626 otu_139206 otu_103589 otu_143077 otu_115682 otu_126668
otu_415 otu_27428 otu_2798 otu_99290 otu_3077 otu_109628 otu_5066 otu_28157
otu_97193 otu_138523 otu_4368 otu_32380 otu_136082 otu_99341 otu_138270
otu_154348 otu_24536 otu_106755 otu_103493 otu_136957 otu_140869 otu_3722
otu_82027 otu_6712 otu_110229 otu_120697 otu_31838 otu_151487 otu_26756 otu_70
otu_276 otu_106816 otu_32893 otu_108481 otu_99542 otu_2646 otu_150374 otu_5856
otu_6174 otu_2759 otu_154448 otu_34824 otu_30854 otu_103392 otu_5661 otu_153286
otu_29790 otu_79420 otu_153645 otu_110611 otu_102818 otu_147275 otu_33399
otu_29175 otu_49774 otu_108466 otu_23970 otu_134681 otu_132680 otu_2946 otu_4190
otu_4924 otu_3930 otu_150436 otu_30361 otu_108192 otu_106676 otu_14083 otu_23738
otu_136428 otu_4003 otu_106834 otu_23836 otu_6364 otu_4357 otu_23085 otu_137612
otu_28274 otu_99367 otu_79144 otu_32946 otu_23211 otu_33584 otu_2391 otu_136944
otu_2819 otu_3761 otu_141832 otu_163245 otu_31066 otu_153040 otu_6190 otu_1532
otu_34280 otu_144198 otu_31932 otu_102871 otu_22391 otu_115749 otu_151454
otu_154807 otu_27094 otu_26699 otu_27932 otu_140688 otu_27743 otu_147873
otu_5438 otu_6601 otu_35039 otu_105881 otu_529 otu_54898 otu_139665 otu_34074
otu_35310 otu_45572 otu_5483 otu_104870 otu_102988 otu_2538 otu_22824 otu_1195
otu_152819 otu_2233 otu_152213 otu_103458 otu_109864 otu_32885 otu_3806
otu_26998 otu_23192 otu_110406 otu_99902 otu_106266 otu_110348 otu_578 otu_137634
otu_28607 otu_106546 otu_141896 otu_137844 otu_82473 otu_24241 otu_29179
otu_141135 otu_151866 otu_387 otu_163987 otu_53385 otu_154469 otu_99926
otu_142316 otu_97483 otu_146988 otu_139231 otu_106124 otu_23281 otu_150199
otu_101877 otu_26252 otu_104977 otu_83323 otu_82394 otu_108566 otu_79974
otu_127499 otu_106054 otu_137805 otu_139189 otu_81414 otu_127787 otu_1608
otu_164052 otu_24000 otu_30669 otu_134320 otu_2630 otu_109793 otu_5089 otu_79363
otu_750 otu_99615 otu_153733 otu_103539 otu_2029 otu_108452 otu_89493 otu_99256
otu_25117 otu_140957 otu_151259 otu_29736 otu_99759 otu_132478 otu_2153
otu_99318 otu_23119 otu_4148 otu_108001 otu_149061 otu_60273 otu_4343 otu_24547
otu_24816 otu_31584 otu_79482 otu_25757 otu_107883 otu_105569 otu_601 otu_103138
otu_80904 otu_80534 otu_144878 otu_139881 otu_33111 otu_109139 otu_152086 otu_8
otu_106890 otu_138557 otu_6906 otu_22905 otu_143995 otu_2823 otu_34245 otu_1409
otu_23164 otu_103423 otu_7086 otu_152473 otu_3435 otu_2695 otu_126642 otu_127556
otu_473 otu_34587 otu_45939 otu_31238 otu_3786 otu_153351 otu_24089 otu_154506
otu_3814 otu_126370 otu_138031 otu_82143 otu_34324 otu_99473 otu_134745
otu_108039 otu_24376 otu_4582 otu_3934 otu_36278 otu_2533 otu_146738 otu_137258
otu_141040 otu_133496 otu_23674 otu_151922 otu_23830 otu_27983 otu_146278
otu_80877 otu_27319 otu_99706 otu_83085 otu_79231 otu_136706 otu_5170 otu_105609
otu_22366 otu_154609 otu_79656 otu_23063 otu_93624 otu_103576 otu_22660
otu_34142 otu_23762 otu_29451 otu_150670 otu_81713 otu_31218 otu_2917 otu_22580
otu_97036 otu_6355 otu_126735 otu_107932 otu_6792 otu_23006 otu_103348 otu_1455
otu_138486 otu_22417 otu_98315 otu_46520 otu_152619 otu_145170 otu_106741
otu_140759 otu_20653 otu_82741 otu_27193 otu_81668 otu_150364 otu_120617
otu_150667 otu_110638 otu_6911 otu_132678 otu_134504 otu_106424 otu_20529
otu_99360 otu_152037 otu_25600 otu_108428 otu_81526 otu_3772 otu_25629
otu_108297 otu_2143 otu_81423 otu_26786 otu_31953 otu_29607 otu_23018 otu_79402
otu_144468 otu_5206 otu_151355 otu_151066 otu_4219 otu_4928 otu_138829
otu_126962 otu_7024 otu_1866 otu_140859 otu_108627 otu_148825 otu_126400
otu_82518 otu_769 otu_127560 otu_150683 otu_146301 otu_24233 otu_83345 otu_23121
otu_154561 otu_108338 otu_109143 otu_82580 otu_46965 otu_146226 otu_138166
otu_96931 otu_2947 otu_132617 otu_109159 otu_26118 otu_35193 otu_83880 otu_12486
otu_24352 otu_25018 otu_3511 otu_79050 otu_151739 otu_157139 otu_146475
otu_144347 otu_139864 otu_29542 otu_99377 otu_105592 otu_1592 otu_147387
otu_127009 otu_4551 otu_27313 otu_146353 otu_2540 otu_26371 otu_139627
otu_145480 otu_142089 otu_117126 otu_153864 otu_153554 otu_138571 otu_24958
otu_99326 otu_1339 otu_35945 otu_136755 otu_2280 otu_19276 otu_142099 otu_79811
otu_83951 otu_26940 otu_25417 otu_140198 otu_2462 otu_146889 otu_2498 otu_81950
otu_2423 otu_146511 otu_22801 otu_1942 otu_79416 otu_139567 otu_28898 otu_147501
otu_28258 otu_152285 otu_7344 otu_96667 otu_99652 otu_28521 otu_32132 otu_6034
otu_106960 otu_163370 otu_147229 otu_141870 otu_153020 otu_36146 otu_33717
otu_151398 otu_150412 otu_134375 otu_33954 otu_23459 otu_5113 otu_126550
otu_2685 otu_2152 otu_152667 otu_96905 otu_150820 otu_132446 otu_79627 otu_25860
otu_105741 otu_3311 otu_24678 otu_6764 otu_151400 otu_4806 otu_3503 otu_3300
otu_49344 otu_5803 otu_106506 otu_25233 otu_4564 otu_132571 otu_144426 otu_83505
otu_82807 otu_106889 otu_99662 otu_29620 otu_35628 otu_150255 otu_5108 otu_79354
otu_139751 otu_33845 otu_102929 otu_2854 otu_5681 otu_1151 otu_109952 otu_50611
otu_141995 otu_150590 otu_137863 otu_22843 otu_4341 otu_150168 otu_4334 otu_1364
otu_137617 otu_106591 otu_100045 otu_99393 otu_141813 otu_146968 otu_118287
otu_27116 otu_163267 otu_22572 otu_2913 otu_499 otu_129822 otu_1067 otu_28537
otu_105478 otu_36014 otu_22495 otu_29865 otu_136464 otu_23177 otu_109196
otu_80927 otu_23437 otu_28033 otu_32352 otu_99876 otu_35558 otu_104332
otu_106445 otu_22790 otu_25298 otu_81431 otu_22881 otu_104880 otu_144224
otu_136750 otu_139901 otu_97083 otu_26051 otu_82915 otu_107537 otu_139238
otu_4827 otu_32003 otu_132219 otu_108959 otu_142059 otu_64603 otu_108673
otu_60851 otu_146255 otu_34183 otu_141011 otu_30542 otu_149415 otu_135104

[illegible]
otu_82807 otu_106889 otu_99662 otu_29620 otu_35628 otu_150255 otu_5108 otu_79354
otu_139751 otu_33845 otu_102929 otu_2854 otu_5681 otu_1151 otu_109952 otu_50611
otu_141995 otu_150590 otu_137863 otu_22843 otu_4341 otu_150168 otu_4334 otu_1364
otu_137617 otu_106591 otu_100045 otu_99393 otu_141813 otu_146968 otu_118287
otu_27116 otu_163267 otu_22572 otu_2913 otu_499 otu_129822 otu_1067 otu_28537
otu_105478 otu_36014 otu_22495 otu_29865 otu_136464 otu_23177 otu_109196
otu_80927 otu_23437 otu_28033 otu_32352 otu_99876 otu_35558 otu_104332
otu_106445 otu_22790 otu_25298 otu_81431 otu_22881 otu_104880 otu_144224
otu_136750 otu_139901 otu_97083 otu_26051 otu_82915 otu_107537 otu_139238
otu_4827 otu_32003 otu_132219 otu_108959 otu_142059 otu_64603 otu_108673
otu_60851 otu_146255 otu_34183 otu_141011 otu_30542 otu_149415 otu_135104
otu_32266 otu_30830 otu_81345 otu_24112 otu_132565 otu_154204 otu_81991
otu_134661 otu_147314 otu_1177 otu_5432 otu_103527 otu_139810 otu_1474
otu_105854 otu_4189 otu_140850 otu_146289 otu_79223 otu_31976 otu_126881
otu_5691 otu_78947 otu_105952 otu_107547 otu_137080 otu_149883 otu_127432
otu_34461 otu_140727 otu_5750 otu_35042 otu_132782 otu_4976 otu_108638 otu_30399
otu_99253 otu_23694 otu_103193 otu_107079 otu_1147 otu_24660 otu_105708 otu_4160
otu_28358 otu_142534 otu_154538 otu_80838 otu_25208 otu_83351 otu_132599
otu_140706 otu_132516 otu_154020 otu_141130 otu_136063 otu_97733 otu_6075
otu_96743 otu_33349 otu_25903 otu_141582 otu_14280 otu_109961 otu_22921
otu_132570 otu_99310 otu_152890 otu_7367 otu_144366 otu_13107 otu_99521 otu_6247
otu_22531 otu_753 otu_26347 otu_82744 otu_146880 otu_32417 otu_154276 otu_133785
otu_32747 otu_106998 otu_23310 otu_2812 otu_136698 otu_36435 otu_105656 otu_2839
otu_98021 otu_4831 otu_32165 otu_591 otu_79689 otu_143852 otu_3053 otu_136131
otu_2134 otu_150149 otu_6297 otu_109720 otu_24638 otu_2375 otu_148756 otu_7375
otu_136075 otu_22870 otu_99011 otu_26314 otu_147398 otu_99768 otu_136439
otu_2410 otu_108208 otu_23558 otu_134970 otu_151863 otu_154810 otu_152832
otu_96823 otu_67068 otu_28460 otu_30421 otu_80336 otu_28666 otu_7081 otu_105795
otu_25442 otu_5236 otu_145002 otu_2728 otu_7193 otu_105606 otu_96957 otu_13428
otu_23872 otu_82597 otu_149504 otu_116722 otu_137056 otu_99284 otu_164057
otu_147128 otu_25933 otu_106818 otu_109115 otu_4315 otu_142146 otu_147241
otu_81909 otu_139934 otu_152857 otu_136472 otu_103125 otu_105230 otu_96719
otu_142447 otu_2348 otu_32629 otu_3274 otu_109590 otu_141803 otu_1490 otu_34627
otu_33118 otu_126523 otu_151322 otu_147153 otu_146331 otu_33752 otu_34721
otu_146800 otu_126825 otu_3240 otu_3373 otu_150711 otu_33270 otu_144070
otu_106307 otu_4647 otu_99300 otu_850 otu_137813 otu_650 otu_163289 otu_147438
otu_105524 otu_5383 otu_22505 otu_103168 otu_153172 otu_136522 otu_65062
otu_24724 otu_139792 otu_146290 otu_121153 otu_147211 otu_26358 otu_45107
otu_1588 otu_134313 otu_3236 otu_150217 otu_34365 otu_107794 otu_136447
otu_83337 otu_4680 otu_84077 otu_106168 otu_139162 otu_25266 otu_109836
otu_83349 otu_24086 otu_127379 otu_6342 otu_102848 otu_150936 otu_4312
otu_110248 otu_136948 otu_89773 otu_22482 otu_151744 otu_105498 otu_106363
otu_115704 otu_99735 otu_37198 otu_6877 otu_21871 otu_146239 otu_157044
otu_126331 otu_146569 otu_99602 otu_26766 otu_147335 otu_126628 otu_25255
otu_126609 otu_2269 otu_151256 otu_105537 otu_99842 otu_27946 otu_150617
otu_152407 otu_36356 otu_3762 otu_34615 otu_99466 otu_79199 otu_147406
otu_137797 otu_151245 otu_84211 otu_79940 otu_5252 otu_79251 otu_99389
otu_142259 otu_150277 otu_136155 otu_105822 otu_2173 otu_163229 otu_107047
otu_28131 otu_23174 otu_22459 otu_37199 otu_6341 otu_23562 otu_28169 otu_142083
otu_24828 otu_154072 otu_152966 otu_2592 otu_2940 otu_4743 otu_63438 otu_26329
otu_24117 otu_32283 otu_154533 otu_151930 otu_2317 otu_7357 otu_117863 otu_80010
otu_146589 otu_105485 otu_139556 otu_109176 otu_151917 otu_152059 otu_108607
otu_103282 otu_83005 otu_30661 otu_133122 otu_4583 otu_96846 otu_24359 otu_22746
otu_4747 otu_139600 otu_126554 otu_106884 otu_103413 otu_36831 otu_7038
otu_164131 otu_152157 otu_103177 otu_84225 otu_3069 otu_107575 otu_79335
otu_99826 otu_3753 otu_82952 otu_22595 otu_3075 otu_6697 otu_5886 otu_4227
otu_152201 otu_25289 otu_108460 otu_83213 otu_149005 otu_24465 otu_139532
otu_82966 otu_82502 otu_103370 otu_127638 otu_152021 otu_154646 otu_104962
otu_152131 otu_103043 otu_79833 otu_150078 otu_106709 otu_153121 otu_137825
otu_140687 otu_106567 otu_9545 otu_143908 otu_79376 otu_149734 otu_40 otu_150591
otu_99463 otu_132810 otu_106098 otu_17193 otu_34584 otu_99984 otu_16013
otu_36500 otu_2256 otu_140818 otu_100545 otu_1636 otu_140794 otu_139873
otu_106149 otu_2708 otu_100126 otu_102898 otu_81237 otu_6740 otu_4975 otu_102865
otu_22780 otu_82776 otu_6610 otu_83602 otu_115999 otu_150856 otu_14065
otu_137799 otu_1090 otu_35308 otu_64174 otu_22476 otu_136120 otu_128008
otu_147394 otu_150877 otu_3889 otu_140745 otu_122592 otu_153861 otu_10752
otu_152932 otu_82912 otu_24179 otu_33012 otu_110410 otu_32019 otu_31933
otu_22804 otu_139659 otu_105458 otu_151885 otu_26110 otu_106608 otu_163998
otu_146703 otu_146269 otu_137627 otu_138543 otu_23019 otu_592 otu_5155 otu_5616
otu_152840 otu_30736 otu_144118 otu_152214 otu_106246 otu_102872 otu_151552
otu_99560 otu_146382 otu_36117 otu_146386 otu_27258 otu_80941 otu_80112 otu_4117
otu_104669 otu_3114 otu_144307 otu_150779 otu_126549 otu_99459 otu_80478
otu_2967 otu_132986 otu_23818 otu_34465 otu_103074 otu_146374 otu_3281 otu_6451
otu_150303 otu_134341 otu_138161 otu_110649 otu_2021 otu_35140 otu_104151
otu_132588 otu_24057 otu_134358 otu_129970 otu_134315 otu_36224 otu_5021
otu_36241 otu_81277 otu_5692 otu_137638 otu_28812 otu_142536 otu_100160
otu_12733 otu_140725 otu_36127 otu_1981 otu_80763 otu_108295 otu_140522
otu_151269 otu_35966 otu_889 otu_26425 otu_29013 otu_2909 otu_31975 otu_147323
otu_2413 otu_22420 otu_134541 otu_5594 otu_418 otu_2963 otu_83343 otu_28767
otu_132484 otu_6480 otu_12966 otu_30712 otu_36372 otu_26986 otu_99272 otu_153974
otu_32637 otu_99968 otu_22635 otu_22813 otu_31579 otu_146594 otu_31003 otu_31359
otu_6216 otu_25571 otu_138138 otu_23892 otu_3310 otu_79190 otu_107096 otu_475
otu_99228 otu_28158 otu_36109 otu_24605 otu_150959 otu_27272 otu_154202
otu_150096 otu_134556 otu_63135 otu_79730 otu_145054 otu_151019 otu_22473
otu_80441 otu_151119 otu_62843 otu_33541 otu_148103 otu_22703 otu_127482
otu_22323 otu_3243 otu_79913 otu_146832 otu_26421 otu_83748 otu_132498 otu_80190
otu_49950 otu_10004 otu_150144 otu_32524 otu_106589 otu_80630 otu_5265
otu_140893 otu_106092 otu_138615 otu_45807 otu_80287 otu_11133 otu_4429 otu_4987
otu_102815 otu_81885 otu_1547 otu_154832 otu_29917 otu_138251 otu_22542
otu_27436 otu_5717 otu_5478 otu_81329 otu_146370 otu_107386 otu_150852 otu_5348
otu_33190 otu_26041 otu_1220 otu_106193 otu_163406 otu_23415 otu_151798
otu_31232 otu_1329 otu_22959 otu_146462 otu_36091 otu_25027 otu_81660 otu_83953
otu_151173 otu_143974 otu_146828 otu_4566 otu_126787 otu_82876 otu_27991
otu_146714 otu_141890 otu_142044 otu_3331 otu_22853 otu_5430 otu_26223 otu_9657
otu_3721 otu_107340 otu_32575 otu_139668 otu_48478 otu_5733 otu_102968 otu_26893
otu_509 otu_534 otu_147062 otu_146297 otu_2399 otu_99395 otu_159497 otu_80826
otu_82416 otu_80990 otu_4067 otu_132814 otu_34807 otu_104573 otu_144190
otu_153131 otu_106415 otu_30194 otu_110464 otu_147267 otu_153201 otu_23634
otu_93738 otu_2986 otu_52025 otu_25882 otu_106144 otu_99524 otu_149239 otu_24794
otu_82917 otu_2362 otu_32126 otu_5720 otu_2576 otu_136060 otu_106871 otu_6409
otu_105000 otu_2329 otu_147156 otu_68344 otu_2409 otu_141001 otu_127650
otu_24784 otu_148425 otu_106867 otu_109474 otu_836 otu_109097 otu_96715
otu_24824 otu_126928 otu_3200 otu_26990 otu_107836 otu_148302 otu_126396
otu_4284 otu_106379 otu_99530 otu_132504 otu_96653 otu_109457 otu_142426
otu_3288 otu_23088 otu_107677 otu_22373 otu_127952 otu_26666 otu_30 otu_81087
otu_24661 otu_106033 otu_82601 otu_27957 otu_31719 otu_137052 otu_25013
otu_153037 otu_146479 otu_24458 otu_143038 otu_3297 otu_82300 otu_138143
otu_22933 otu_24645 otu_138540 otu_146919 otu_17456 otu_106516 otu_153374
[illegible]

otu_153131 otu_106415 otu_30194 otu_110464 otu_147267 otu_153201 otu_23634
otu_93738 otu_2986 otu_52025 otu_25882 otu_106144 otu_99524 otu_149239 otu_24794
otu_82917 otu_2362 otu_32126 otu_5720 otu_2576 otu_136060 otu_106871 otu_6409
otu_105000 otu_2329 otu_147156 otu_68344 otu_2409 otu_141001 otu_127650
otu_24784 otu_148425 otu_106867 otu_109474 otu_836 otu_109097 otu_96715
otu_24824 otu_126928 otu_3200 otu_26990 otu_107836 otu_148302 otu_126396
otu_4284 otu_106379 otu_99530 otu_132504 otu_96653 otu_109457 otu_142426
otu_3288 otu_23088 otu_107677 otu_22373 otu_127952 otu_26666 otu_30 otu_81087
otu_24661 otu_106033 otu_82601 otu_27957 otu_31719 otu_137052 otu_25013
otu_153037 otu_146479 otu_24458 otu_143038 otu_3297 otu_82300 otu_138143
otu_22933 otu_24645 otu_138540 otu_146919 otu_17456 otu_106516 otu_153374
otu_79742 otu_27909 otu_132910 otu_86281 otu_134382 otu_150742 otu_25229
otu_23264 otu_138434 otu_736 otu_137094 otu_147076 otu_154477 otu_141702
otu_138485 otu_152894 otu_7106 otu_26994 otu_32690 otu_1428 otu_96735 otu_127542
otu_151793 otu_33309 otu_30622 otu_27175 otu_79423 otu_24432 otu_139173 otu_4796
otu_99727 otu_147845 otu_151712 otu_129986 otu_83456 otu_25464 otu_27330
otu_100022 otu_97103 otu_145196 otu_102735 otu_153876 otu_103167 otu_141834
otu_151085 otu_2046 otu_102880 otu_13648 otu_16969 otu_154548 otu_152440
otu_24290 otu_34980 otu_80187 otu_25550 otu_35957 otu_146464 otu_24739
otu_153784 otu_144083 otu_31210 otu_152480 otu_36134 otu_88871 otu_2838 otu_3029
otu_151686 otu_82929 otu_98983 otu_96916 otu_99544 otu_79210 otu_147347
otu_151326 otu_107427 otu_110486 otu_17490 otu_99402 otu_132589 otu_109805
otu_33603 otu_80839 otu_2567 otu_26094 otu_4052 otu_128035 otu_149095 otu_6130
otu_151933 otu_10215 otu_105806 otu_138493 otu_151094 otu_82737 otu_32385
otu_126999 otu_23517 otu_80403 otu_136947 otu_80907 otu_151342 otu_4937
otu_134425 otu_22588 otu_151244 otu_97161 otu_106355 otu_32739 otu_25868
otu_142297 otu_79387 otu_106024 otu_81152 otu_163322 otu_107868 otu_31448
otu_101038 otu_151394 otu_11769 otu_80627 otu_26248 otu_79879 otu_152182
otu_143059 otu_131995 otu_106158 otu_107165 otu_153735 otu_82787 otu_99809
otu_154709 otu_33576 otu_82943 otu_6617 otu_143248 otu_79444 otu_136089 otu_2355
otu_148143 otu_30360 otu_29233 otu_146288 otu_22796 otu_107110 otu_133559
otu_154580 otu_30039 otu_140726 otu_99858 otu_146977 otu_129602 otu_126158
otu_6264 otu_2376 otu_27255 otu_82655 otu_27687 otu_82398 otu_154163 otu_150420
otu_30943 otu_79525 otu_23283 otu_9683 otu_118083 otu_105465 otu_34836
otu_146718 otu_102833 otu_138179 otu_28816 otu_151053 otu_107276 otu_27516
otu_102914 otu_84222 otu_580 otu_1357 otu_81469 otu_22850 otu_107654 otu_150973
otu_151099 otu_83411 otu_80294 otu_99637 otu_5890 otu_26281 otu_31822 otu_6961
otu_79703 otu_137985 otu_34672 otu_137858 otu_132724 otu_82293 otu_146457
otu_140167 otu_80277 otu_137332 otu_137134 otu_140854 otu_36617 otu_99792
otu_96689 otu_29830 otu_103288 otu_137063 otu_126627 otu_99658 otu_92908
otu_99301 otu_78954 otu_132465 otu_83240 otu_127420 otu_79169 otu_4596 otu_4700
otu_36587 otu_146437 otu_3068 otu_132717 otu_147170 otu_22785 otu_35747 otu_522
otu_105626 otu_134355 otu_80270 otu_98086 otu_29229 otu_23646 otu_138199
otu_144030 otu_2727 otu_107605 otu_137933 otu_127923 otu_25120 otu_139318
otu_126501 otu_146742 otu_146797 otu_3716 otu_4151 otu_136132 otu_32647
otu_23856 otu_137682 otu_136112 otu_80380 otu_28971 otu_24835 otu_154373
otu_143827 otu_104172 otu_96989 otu_96898 otu_126386 otu_22543 otu_5175
otu_105226 otu_134507 otu_140675 otu_29914 otu_126393 otu_79984 otu_4826
otu_5554 otu_142417 otu_150615 otu_32234 otu_104145 otu_81785 otu_104474
otu_24337 otu_104863 otu_109246 otu_26821 otu_6945 otu_139756 otu_80794
otu_29374 otu_143810 otu_5333 otu_80821 otu_99819 otu_24220 otu_132569 otu_20590
otu_24717 otu_81622 otu_13366 otu_81459 otu_103515 otu_146234 otu_12165
otu_110122 otu_143897 otu_23730 otu_99440 otu_99262 otu_2239 otu_561 otu_143446
otu_146227 otu_28577 otu_2914 otu_23077 otu_151200 otu_30127 otu_82204 otu_6848
otu_818 otu_44465 otu_97839 otu_79080 otu_5013 otu_140961 otu_98685 otu_152299
otu_22437 otu_25210 otu_134748 otu_137075 otu_36542 otu_461 otu_126436
otu_103003 otu_163984 otu_140367 otu_548 otu_27221 otu_153979 otu_107945
otu_2825 otu_6942 otu_152623 otu_29762 otu_102837 otu_97933 otu_3246 otu_7094
otu_3230 otu_28242 otu_32702 otu_141859 otu_102943 otu_163281 otu_134728
otu_2486 otu_96759 otu_152889 otu_3853 otu_106336 otu_109690 otu_109132
otu_36835 otu_150109 otu_142123 otu_137088 otu_133111 otu_146857 otu_82413
otu_11019 otu_143932 otu_35914 otu_127469 otu_102870 otu_97152 otu_109324
otu_3695 otu_109448 otu_141809 otu_142573 otu_143858 otu_134805 otu_118352
otu_3602 otu_150246 otu_133077 otu_106376 otu_109340 otu_82365 otu_2661
otu_139514 otu_24120 otu_2519 otu_105425 otu_31710 otu_14561 otu_81456 otu_6193
otu_80516 otu_99570 otu_80750 otu_152766 otu_24668 otu_105993 otu_144225
otu_93223 otu_16335 otu_6185 otu_140874 otu_138476 otu_152944 otu_147301
otu_151279 otu_3830 otu_132671 otu_109776 otu_126799 otu_26610 otu_83952
otu_104907 otu_25279 otu_146955 otu_82648 otu_7334 otu_151476 otu_109250
otu_127576 otu_79579 otu_105932 otu_33958 otu_102792 otu_31567 otu_5149 otu_695
otu_134307 otu_146903 otu_99349 otu_31596 otu_126482 otu_31180 otu_137651
otu_27107 otu_146897 otu_153507 otu_150093 otu_150083 otu_4839 otu_138109
otu_22829 otu_24992 otu_35881 otu_24640 otu_34950 otu_32106 otu_79601 otu_96852
otu_146211 otu_5307 otu_28899 otu_23793 otu_25791 otu_4085 otu_22758 otu_146271
otu_54292 otu_598 otu_1640 otu_79780 otu_29460 otu_25766 otu_138290 otu_23118
otu_109613 otu_146356 otu_96862 otu_28664 otu_99408 otu_3010 otu_142079
otu_107866 otu_151370 otu_79558 otu_146483 otu_80306 otu_105411 otu_31417
otu_33900 otu_100063 otu_4379 otu_28609 otu_1637 otu_5234 otu_3379 otu_80301
otu_1084 otu_103012 otu_55518 otu_107711 otu_145405 otu_151553 otu_142246
otu_140973 otu_99508 otu_25040 otu_117997 otu_96781 otu_23288 otu_80896 otu_3410
otu_81731 otu_150757 otu_61471 otu_846 otu_14382 otu_148481 otu_141158
otu_104539 otu_152502 otu_127440 otu_1978 otu_99950 otu_6603 otu_5148 otu_28858
otu_7189 otu_127610 otu_6199 otu_152870 otu_4528 otu_139717 otu_31562 otu_99348
otu_133457 otu_99273 otu_23994 otu_146268 otu_159996 otu_79741 otu_80054
otu_152498 otu_132894 otu_32972 otu_99679 otu_82129 otu_134542 otu_22319
otu_106578 otu_109748 otu_106829 otu_5305 otu_3490 otu_23162 otu_99258
otu_127333 otu_27568 otu_5690 otu_99292 otu_103106 otu_33378 otu_142064
otu_27790 otu_147189 otu_142541 otu_81662 otu_25908 otu_151861 otu_138494
otu_147322 otu_153321 otu_1250 otu_126215 otu_126168 otu_26266 otu_27518
otu_23120 otu_146478 otu_13616 otu_107169 otu_146396 otu_81177 otu_79450
otu_102945 otu_99447 otu_45533 otu_80284 otu_108273 otu_23305 otu_143880
otu_127679 otu_27114 otu_32467 otu_67418 otu_105632 otu_22481 otu_151230
otu_98587 otu_107114 otu_6534 otu_28115 otu_118163 otu_96825 otu_83144 otu_3771
otu_148209 otu_31930 otu_4181 otu_150197 otu_137637 otu_12277 otu_79774
otu_140877 otu_28641 otu_106297 otu_6340 otu_163238 otu_98153 otu_134124
otu_24571 otu_10133 otu_99252 otu_81605 otu_107289 otu_22561 otu_151127
otu_81399 otu_22653 otu_24370 otu_137980 otu_23934 otu_146588 otu_99471
otu_141273 otu_22546 otu_140557 otu_33106 otu_133642 otu_106899 otu_107182
otu_11975 otu_142107 otu_23903 otu_24452 otu_99705 otu_152521 otu_13224
otu_97075 otu_146320 otu_25193 otu_28567 otu_10195 otu_122822 otu_2162
otu_147462 otu_24105 otu_4882 otu_139804 otu_83704 otu_151762 otu_143193
otu_20683 otu_410 otu_4395 otu_29680 otu_151746 otu_3205 otu_80161 otu_29878
otu_99236 otu_99799 otu_25217 otu_97049 otu_106166 otu_25970 otu_144041
otu_33919 otu_151588 otu_5558 otu_81705 otu_134392 otu_107692 otu_147854
otu_82756 otu_22873 otu_16526 otu_110467 otu_106046 otu_27952 otu_141825
otu_102938 otu_82996 otu_136091 otu_152132 otu_151102 otu_136884 otu_150077
otu_4081 otu_98376 otu_140674 otu_28966 otu_33248 otu_138903 otu_79361 otu_24267
otu_164059 otu_4666 otu_120266 otu_36703 otu_139821 otu_51443 otu_152617
otu_96804 otu_150861 otu_163274 otu_151038 otu_147472 otu_152614 otu_107458

otu_141273 otu_22546 otu_140557 otu_33106 otu_133642 otu_106899 otu_107182
otu_11975 otu_142107 otu_23903 otu_24452 otu_99705 otu_152521 otu_13224
otu_97075 otu_146320 otu_25193 otu_28567 otu_10195 otu_122822 otu_2162
otu_147462 otu_24105 otu_4882 otu_139804 otu_83704 otu_151762 otu_143193
otu_20683 otu_410 otu_4395 otu_29680 otu_151746 otu_3205 otu_80161 otu_29878
otu_99236 otu_99799 otu_25217 otu_97049 otu_106166 otu_25970 otu_144041
otu_33919 otu_151588 otu_5558 otu_81705 otu_134392 otu_107692 otu_147854
otu_82756 otu_22873 otu_16526 otu_110467 otu_106046 otu_27952 otu_141825
otu_102938 otu_82996 otu_136091 otu_152132 otu_151102 otu_136884 otu_150077
otu_4081 otu_98376 otu_140674 otu_28966 otu_33248 otu_138903 otu_79361 otu_24267
otu_164059 otu_4666 otu_120266 otu_36703 otu_139821 otu_51443 otu_152617
otu_96804 otu_150861 otu_163274 otu_151038 otu_147472 otu_152614 otu_107458
otu_160021 otu_103059 otu_98828 otu_1585 otu_146697 otu_28341 otu_139895
otu_5860 otu_132457 otu_136736 otu_1751 otu_22716 otu_36189 otu_153191
otu_104599 otu_34383 otu_139586 otu_99286 otu_146246 otu_105555 otu_3390
otu_34226 otu_98071 otu_84036 otu_152434 otu_126667 otu_82992 otu_106864
otu_163268 otu_150895 otu_107260 otu_151535 otu_67110 otu_133061 otu_108990
otu_31268 otu_25764 otu_22380 otu_105150 otu_79139 otu_150472 otu_146931
otu_107385 otu_24412 otu_140702 otu_22852 otu_27507 otu_146724 otu_144265
otu_32117 otu_79790 otu_139573 otu_150817 otu_151482 otu_134387 otu_33669
otu_97004 otu_143365 otu_81271 otu_110465 otu_30687 otu_106007 otu_163372
otu_13941 otu_79818 otu_110618 otu_81246 otu_79164 otu_12493 otu_23690 otu_46341
otu_110413 otu_138423 otu_107176 otu_99565 otu_27304 otu_146261 otu_132460
otu_3108 otu_17335 otu_80747 otu_142250 otu_23697 otu_141886 otu_139180
otu_24079 otu_6574 otu_82652 otu_7074 otu_685 otu_3201 otu_79445 otu_137321
otu_107162 otu_79613 otu_5028 otu_150597 otu_23346 otu_26106 otu_29929
otu_147035 otu_105469 otu_3116 otu_79191 otu_147329 otu_34037 otu_139154
otu_139676 otu_146895 otu_151528 otu_26871 otu_109671 otu_97600 otu_126960
otu_23040 otu_108792 otu_105764 otu_139653 otu_108470 otu_100070 otu_80128
otu_149964 otu_28286 otu_152756 otu_152001 otu_26554 otu_152154 otu_151977
otu_137943 otu_23127 otu_132447 otu_137609 otu_99967 otu_146692 otu_22484
otu_33461 otu_7113 otu_28474 otu_3792 otu_22725 otu_140982 otu_146256 otu_27396
otu_126325 otu_108098 otu_4746 otu_132499 otu_6530 otu_22457 otu_2847 otu_79523
otu_97087 otu_5436 otu_108175 otu_139247 otu_6102 otu_150571 otu_60475 otu_99766
otu_79331 otu_6143 otu_103080 otu_142507 otu_9355 otu_24342 otu_102909 otu_25388
otu_80653 otu_239 otu_144474 otu_134324 otu_31436 otu_5536 otu_147419 otu_23439
otu_81201 otu_149580 otu_153944 otu_154516 otu_158300 otu_150851 otu_147359
otu_2235 otu_26542 otu_23563 otu_3170 otu_81897 otu_2872 otu_150516 otu_146446
otu_49483 otu_127500 otu_36524 otu_34967 otu_143901 otu_108623 otu_2415
otu_106065 otu_1443 otu_139933 otu_103622 otu_148093 otu_117583 otu_163222
otu_27024 otu_147092 otu_139918 otu_99663 otu_134413 otu_84100 otu_143860
otu_151064 otu_150143 otu_79111 otu_106126 otu_150808 otu_32600 otu_34789 otu_29
otu_30896 otu_5264 otu_153405 otu_103191 otu_99536 otu_27332 otu_137812
otu_132682 otu_99343 otu_3718 otu_31286 otu_82250 otu_106494 otu_137614 otu_3031
otu_152869 otu_28713 otu_3214 otu_99296 otu_83740 otu_103376 otu_137700
otu_108486 otu_5961 otu_79715 otu_136711 otu_33069 otu_827 otu_153643 otu_22878
otu_82319 otu_5245 otu_103171 otu_104440 otu_132908 otu_30508 otu_105583
otu_133126 otu_26150 otu_4101 otu_23175 otu_146316 otu_2805 otu_35769 otu_3662
otu_149863 otu_151456 otu_27215 otu_96809 otu_150565 otu_154022 otu_47295
otu_148792 otu_97183 otu_150634 otu_6077 otu_131 otu_23523 otu_81615 otu_80410
otu_145046 otu_32552 otu_80071 otu_126883 otu_105654 otu_151884 otu_35017
otu_28297 otu_149770 otu_80445 otu_4288 otu_99808 otu_3224 otu_80395 otu_109911
otu_22851 otu_600 otu_22750 otu_106052 otu_107498 otu_28593 otu_139817 otu_80293
otu_22937 otu_33132 otu_105406 otu_5231 otu_99468 otu_109328 otu_108065
otu_96705 otu_83549 otu_31698 otu_104815 otu_28248 otu_3995 otu_99696 otu_151447
otu_35143 otu_106917 otu_79465 otu_140704 otu_31970 otu_127583 otu_116614
otu_147259 otu_152044 otu_146480 otu_23729 otu_83261 otu_138553 otu_141481
otu_28389 otu_103317 otu_80490 otu_152219 otu_28024 otu_97960 otu_81005
otu_109186 otu_105509 otu_34440 otu_31821 otu_401 otu_33222 otu_3263 otu_33480
otu_99757 otu_36822 otu_143198 otu_117102 otu_141843 otu_106669 otu_152928
otu_36610 otu_105176 otu_32286 otu_139748 otu_22375 otu_105668 otu_100099
otu_149922 otu_3824 otu_104552 otu_132672 otu_4879 otu_150221 otu_107599
otu_32419 otu_79494 otu_5143 otu_573 otu_154825 otu_145081 otu_82412 otu_33997
otu_27463 otu_25843 otu_80089 otu_34553 otu_138546 otu_23588 otu_3418 otu_146796
otu_144870 otu_886 otu_104991 otu_81559 otu_27199 otu_99840 otu_87259 otu_154426
otu_22439 otu_146906 otu_3308 otu_142090 otu_80086 otu_102811 otu_79698 otu_5555
otu_64127 otu_104388 otu_23605 otu_36244 otu_136210 otu_146442 otu_907 otu_82956
otu_137059 otu_4642 otu_151924 otu_103468 otu_87138 otu_27831 otu_34747
otu_81768 otu_150243 otu_133948 otu_147082 otu_106603 otu_96756 otu_138431
otu_6446 otu_99881 otu_6321 otu_127888 otu_5176 otu_14426 otu_106481 otu_1631
otu_55624 otu_80312 otu_3397 otu_3051 otu_24766 otu_22361 otu_152884 otu_142161
otu_55884 otu_99822 otu_103028 otu_146206 otu_99866 otu_4430 otu_144216 otu_460
otu_130098 otu_126696 otu_152222 otu_29777 otu_22650 otu_107027 otu_105673
otu_110388 otu_154087 otu_34356 otu_2112 otu_5940 otu_28751 otu_28975 otu_32631
otu_5821 otu_22752 otu_83295 otu_30981 otu_100000 otu_83761 otu_18644 otu_150318
otu_79740 otu_3161 otu_6 otu_1799 otu_136433 otu_96832 otu_139527 otu_136450
otu_106199 otu_22429 otu_4593 otu_127451 otu_3130 otu_134554 otu_24650 otu_22688
otu_141837 otu_106354 otu_12125 otu_2346 otu_22683 otu_26076 otu_138619
otu_13831 otu_151224 otu_140921 otu_26788 otu_37202 otu_152176 otu_23890
otu_36137 otu_26811 otu_147064 otu_5867 otu_2460 otu_152305 otu_154673 otu_99911
otu_108473 otu_127178 otu_154168 otu_119911 otu_151098 otu_137307 otu_136730
otu_127018 otu_116153 otu_1382 otu_132982 otu_5204 otu_47532 otu_149268
otu_79282 otu_30451 otu_79571 otu_33779 otu_4971 otu_2547 otu_139629 otu_134705
otu_138459 otu_107091 otu_150156 otu_99370 otu_23298 otu_102983 otu_103455
otu_154701 otu_151054 otu_5110 otu_151220 otu_3284 otu_13567 otu_4607 otu_106258
otu_127868 otu_80246 otu_154036 otu_106059 otu_4169 otu_23061 otu_396 otu_25747
otu_29332 otu_105706 otu_150400 otu_31422 otu_79400 otu_621 otu_80494 otu_22308
otu_29286 otu_110377 otu_99780 otu_36938 otu_2929 otu_27335 otu_102851
otu_109512 otu_27163 otu_97136 otu_24621 otu_109414 otu_152510 otu_27146
otu_26748 otu_142451 otu_153483 otu_4713 otu_127426 otu_146355 otu_126409
otu_107554 otu_23988 otu_3802 otu_153105 otu_145134 otu_106269 otu_2222
otu_24956 otu_152699 otu_107056 otu_23095 otu_108524 otu_102885 otu_5195
otu_80583 otu_142289 otu_36183 otu_30798 otu_132645 otu_134538 otu_99324
otu_106817 otu_3829 otu_22826 otu_2238 otu_32155 otu_6282 otu_97656 otu_108893
otu_147345 otu_140805 otu_23452 otu_6511 otu_151826 otu_2509 otu_79269 otu_99982
otu_80181 otu_107889 otu_78977 otu_24606 otu_150689 otu_2583 otu_37150
otu_106455 otu_2451 otu_32057 otu_81524 otu_137402 otu_97755 otu_138521
otu_10551 otu_32822 otu_33934 otu_151893 otu_706 otu_80271 otu_4758 otu_81319
otu_27005 otu_141113 otu_52767 otu_84192 otu_3034 otu_163958 otu_34577 otu_22441
otu_106565 otu_6980 otu_82487 otu_149513 otu_139233 otu_27309 otu_130798
otu_136515 otu_25086 otu_2852 otu_641 otu_81136 otu_102970 otu_106707 otu_107406
otu_99588 otu_5367 otu_106270 otu_146712 otu_146253 otu_34349 otu_424 otu_132471
otu_132732 otu_28555 otu_109882 otu_106953 otu_99674 otu_146678 otu_103524
otu_31459 otu_145257 otu_83500 otu_154473 otu_152229 otu_23463 otu_138616
otu_147283 otu_29851 otu_86135 otu_151849 otu_105825 otu_81671 otu_81992
otu_34934 otu_25507 otu_105919 otu_137076 otu_79394 otu_105482 otu_79242
otu_99916 otu_25394 otu_3063 otu_100167 otu_126459 otu_119290 otu_29813 otu_810
otu_22836 otu_81804 otu_82436 otu_139783 otu_107705 otu_97058 otu_140824
otu_143845 otu_127167 otu_106431 otu_24362 otu_152949 otu_26187 otu_153939

otu_27005 otu_141113 otu_52767 otu_84192 otu_3034 otu_163958 otu_34577 otu_22441
otu_106565 otu_6980 otu_82487 otu_149513 otu_139233 otu_27309 otu_130798
otu_136515 otu_25086 otu_2852 otu_641 otu_81136 otu_102970 otu_106707 otu_107406
otu_99588 otu_5367 otu_106270 otu_146712 otu_146253 otu_34349 otu_424 otu_132471
otu_132732 otu_28555 otu_109882 otu_106953 otu_99674 otu_146678 otu_103524
otu_31459 otu_145257 otu_83500 otu_154473 otu_152229 otu_23463 otu_138616
otu_147283 otu_29851 otu_86135 otu_151849 otu_105825 otu_81671 otu_81992
otu_34934 otu_25507 otu_105919 otu_137076 otu_79394 otu_105482 otu_79242
otu_99916 otu_25394 otu_3063 otu_100167 otu_126459 otu_119290 otu_29813 otu_810
otu_22836 otu_81804 otu_82436 otu_139783 otu_107705 otu_97058 otu_140824
otu_143845 otu_127167 otu_106431 otu_24362 otu_152949 otu_26187 otu_153939
otu_5452 otu_28316 otu_99409 otu_34081 otu_89206 otu_80091 otu_79506 otu_22569
otu_109165 otu_12978 otu_500 otu_30647 otu_99304 otu_109372 otu_6535 otu_34952
otu_79648 otu_104457 otu_150344 otu_59528 otu_151017 otu_150952 otu_99863
otu_127769 otu_25609 otu_138184 otu_79026 otu_136727 otu_102867 otu_107282
otu_135262 otu_27232 otu_137840 otu_607 otu_10096 otu_3143 otu_143976 otu_106582
otu_774 otu_151470 otu_104250 otu_99480 otu_27865 otu_1425 otu_18420 otu_28579
otu_143990 otu_7048 otu_110292 otu_2545 otu_4561 otu_2347 otu_79863 otu_31750
otu_3460 otu_27809 otu_3614 otu_24607 otu_22940 otu_13202 otu_36404 otu_79215
otu_99680 otu_81783 otu_136720 otu_96774 otu_151879 otu_142298 otu_25337
otu_163408 otu_23503 otu_134559 otu_31766 otu_6210 otu_22611 otu_103005 otu_2353
otu_132627 otu_102069 otu_80182 otu_102924 otu_25923 otu_109854 otu_126240
otu_101102 otu_24944 otu_146317 otu_142561 otu_153685 otu_26043 otu_149627
otu_51151 otu_150070 otu_127113 otu_27196 otu_108072 otu_152049 otu_82724
otu_80450 otu_139211 otu_81033 otu_30153 otu_1224 otu_23104 otu_134412
otu_138302 otu_147508 otu_142203 otu_153490 otu_82474 otu_24651 otu_4989
otu_57173 otu_4064 otu_26694 otu_25601 otu_106423 otu_99664 otu_25328 otu_83006
otu_96655 otu_29088 otu_81446 otu_5098 otu_36123 otu_142517 otu_4814 otu_147269
otu_140842 otu_2009 otu_35754 otu_132733 otu_144 otu_6672 otu_152697 otu_104291
otu_80344 otu_28058 otu_106865 otu_22396 otu_31 otu_83903 otu_26586 otu_79977
otu_134311 otu_12800 otu_5722 otu_2578 otu_419 otu_79687 otu_83186 otu_79989
otu_146783 otu_96850 otu_692 otu_35311 otu_27359 otu_146423 otu_140117 otu_126195
otu_107241 otu_812 otu_151198 otu_35370 otu_104306 otu_134633 otu_106697
otu_137650 otu_105608 otu_83982 otu_45363 otu_139719 otu_126385 otu_37228
otu_136069 otu_146640 otu_3370 otu_4095 otu_22583 otu_31949 otu_22666 otu_839
otu_36958 otu_141784 otu_150929 otu_126411 otu_79763 otu_151992 otu_139787
otu_5705 otu_24456 otu_144147 otu_28798 otu_82302 otu_34046 otu_28850 otu_146896
otu_60480 otu_26668 otu_5417 otu_149341 otu_13092 otu_33940 otu_105827
otu_153487 otu_138503 otu_136079 otu_23316 otu_140703 otu_1613 otu_5791
otu_80338 otu_138447 otu_27780 otu_139184 otu_103179 otu_99559 otu_4222
otu_83741 otu_30791 otu_107645 otu_1273 otu_22838 otu_26272 otu_2351 otu_163299
otu_134578 otu_27798 otu_117492 otu_81172 otu_142238 otu_100050 otu_132846
otu_30570 otu_7296 otu_163996 otu_103093 otu_127173 otu_79107 otu_107641
otu_127125 otu_5713 otu_7324 otu_26805 otu_84268 otu_36226 otu_152142 otu_139606
otu_139819 otu_23431 otu_108002 otu_34509 otu_127947 otu_144879 otu_3624
otu_132510 otu_34227 otu_142070 otu_28306 otu_81567 otu_3039 otu_146667
otu_126547 otu_146971 otu_126709 otu_132479 otu_152522 otu_103525 otu_2521
otu_139889 otu_154305 otu_23056 otu_4463 otu_31912 otu_164133 otu_4033 otu_729
otu_79042 otu_139205 otu_25479 otu_134511 otu_30516 otu_106522 otu_128056
otu_1679 otu_79657 otu_7026 otu_142178 otu_25914 otu_30327 otu_79079 otu_151037
otu_6811 otu_143960 otu_24209 otu_79232 otu_6133 otu_80158 otu_105399 otu_2898
otu_29799 otu_36670 otu_25240 otu_105615 otu_99265 otu_82224 otu_23182 otu_9836
otu_23663 otu_132637 otu_4114 otu_146310 otu_79536 otu_493 otu_15585 otu_29768
otu_127533 otu_4688 otu_20579 otu_106215 otu_33416 otu_146344 otu_79599 otu_678
otu_99798 otu_154773 otu_6514 otu_138458 otu_2360 otu_22528 otu_2753 otu_31878
otu_23535 otu_151733 otu_27311 otu_102937 otu_137727 otu_36181 otu_110230
otu_96812 otu_157462 otu_96683 otu_28621 otu_133876 otu_139166 otu_140161
otu_138487 otu_154752 otu_5888 otu_126561 otu_81229 otu_25332 otu_139910
otu_79561 otu_83513 otu_151644 otu_136747 otu_154470 otu_79367 otu_81026
otu_147083 otu_140748 otu_33838 otu_82452 otu_1432 otu_26819 otu_25066
otu_150789 otu_36305 otu_127903 otu_2514 otu_83133 otu_84170 otu_108253
otu_147004 otu_99654 otu_144282 otu_23546 otu_35062 otu_80330 otu_139758
otu_2122 otu_30186 otu_87022 otu_9679 otu_1311 otu_25432 otu_136451 otu_108640
otu_140930 otu_23012 otu_106263 otu_27852 otu_146512 otu_150931 otu_79019
otu_22615 otu_54045 otu_150237 otu_135163 otu_99562 otu_126430 otu_132546
otu_151059 otu_140739 otu_31497 otu_154379 otu_138579 otu_31067 otu_2369
otu_109703 otu_126526 otu_140715 otu_96998 otu_106464 otu_32562 otu_107978
otu_145064 otu_25956 otu_79115 otu_25628 otu_152463 otu_34569 otu_5848
otu_152212 otu_79090 otu_3387 otu_138518 otu_582 otu_23609 otu_22691 otu_81613
otu_151016 otu_150162 otu_30366 otu_139188 otu_137806 otu_2588 otu_46202
otu_26420 otu_83824 otu_100095 otu_459 otu_146438 otu_103446 otu_82723 otu_99567
otu_4695 otu_23759 otu_32870 otu_2163 otu_146990 otu_46209 otu_6326 otu_34850
otu_4298 otu_26170 otu_150998 otu_139721 otu_106777 otu_152564 otu_26249
otu_102117 otu_5659 otu_153332 otu_4361 otu_83690 otu_390 otu_25407 otu_176
otu_4171 otu_89260 otu_153208 otu_4702 otu_35384 otu_146858 otu_146307
otu_106107 otu_148961 otu_29687 otu_142111 otu_25311 otu_181 otu_151262 otu_22431
otu_153636 otu_24222 otu_25822 otu_31512 otu_141971 otu_80368 otu_2158 otu_6139
otu_126798 otu_6353 otu_5666 otu_137755 otu_25424 otu_81036 otu_5331 otu_136095
otu_6728 otu_23803 otu_1915 otu_55520 otu_83622 otu_52809 otu_16293 otu_134673
otu_81081 otu_79701 otu_134509 otu_55699 otu_107773 otu_26959 otu_29740
otu_143895 otu_3092 otu_1152 otu_104958 otu_141806 otu_149810 otu_87819 otu_5684
otu_101742 otu_2274 otu_3081 otu_25776 otu_141114 otu_146376 otu_5263 otu_26350
otu_24102 otu_102831 otu_80096 otu_99270 otu_23357 otu_27558 otu_7029 otu_22798
otu_105705 otu_79540 otu_3128 otu_140772 otu_82348 otu_151706 otu_141905
otu_26070 otu_163336 otu_4392 otu_109130 otu_30492 otu_89909 otu_109298
otu_22918 otu_81760 otu_106191 otu_139739 otu_29679 otu_139845 otu_150418
otu_153799 otu_2210 otu_53298 otu_126456 otu_79592 otu_24488 otu_142157
otu_109758 otu_146232 otu_110651 otu_106810 otu_83702 otu_107572 otu_22946
otu_152334 otu_27524 otu_5521 otu_138467 otu_136949 otu_82181 otu_84024
otu_34348 otu_15615 otu_34574 otu_142268 otu_79866 otu_151356 otu_138136
otu_82256 otu_153152 otu_25269 otu_2294 otu_144142 otu_79209 otu_145178
otu_24062 otu_81458 otu_81062 otu_150682 otu_122682 otu_2827 otu_103188
otu_132951 otu_99786 otu_106969 otu_2384 otu_103601 otu_25118 otu_6704 otu_83943
otu_2974 otu_29325 otu_28562 otu_100114 otu_30590 otu_29502 otu_151892 otu_5048
otu_126860 otu_22353 otu_23328 otu_132492 otu_105527 otu_23768 otu_134568
otu_2992 otu_146281 otu_31306 otu_22998 otu_2467 otu_151092 otu_31310 otu_97060
otu_110175 otu_2288 otu_31658 otu_52315 otu_127154 otu_27151 otu_5477 otu_79474
otu_105453 otu_99751 otu_137277 otu_153588 otu_127177 otu_144319 otu_105794
otu_146887 otu_99579 otu_32154 otu_79438 otu_28374 otu_26657 otu_2237 otu_6788
otu_106827 otu_37191 otu_139714 otu_84136 otu_102972 otu_389 otu_3016 otu_571
otu_24699 otu_2438 otu_68439 otu_26422 otu_102829 otu_96767 otu_146873
otu_143893 otu_30353 otu_80536 otu_139637 otu_35381 otu_23873 otu_106614
otu_33448 otu_139681 otu_126196 otu_22541 otu_3239 otu_146901 otu_135338
otu_146426 otu_6425 otu_697 otu_34221 otu_81876 otu_104698 otu_82833 otu_32093
otu_133058 otu_30678 otu_81103 otu_152799 otu_23122 otu_152107 otu_81780
otu_3251 otu_80956 otu_144007 otu_7427 otu_127442 otu_3546 otu_6914 otu_404
otu_29005 otu_87139 otu_18662 otu_34662 otu_3275 otu_25462 otu_126816 otu_29647
otu_11973 otu_3073 otu_103373 otu_79152 otu_136149 otu_2956 otu_103054 otu_3352
otu_24671 otu_24812 otu_96679 otu_109182 otu_22419 otu_154001 otu_7312

		otu_105453 otu_99751 otu_137277 otu_153588 otu_127177 otu_144319 otu_105794<br>otu_146887 otu_99579 otu_32154 otu_79438 otu_28374 otu_26657 otu_2237 otu_6788<br>otu_106827 otu_37191 otu_139714 otu_84136 otu_102972 otu_389 otu_3016 otu_571<br>otu_24699 otu_2438 otu_68439 otu_26422 otu_102829 otu_96767 otu_146873<br>otu_143893 otu_30353 otu_80536 otu_139637 otu_35381 otu_23873 otu_106614<br>otu_33448 otu_139681 otu_126196 otu_22541 otu_3239 otu_146901 otu_135338<br>otu_146426 otu_6425 otu_697 otu_34221 otu_81876 otu_104698 otu_82833 otu_32093<br>otu_133058 otu_30678 otu_81103 otu_152799 otu_23122 otu_152107 otu_81780<br>otu_3251 otu_80956 otu_144007 otu_7427 otu_127442 otu_3546 otu_6914 otu_404<br>otu_29005 otu_87139 otu_18662 otu_34662 otu_3275 otu_25462 otu_126816 otu_29647<br>otu_11973 otu_3073 otu_103373 otu_79152 otu_136149 otu_2956 otu_103054 otu_3352<br>otu_24671 otu_24812 otu_96679 otu_109182 otu_22419 otu_154001 otu_7312<br>otu_146444 otu_88184 otu_146577 otu_13347 otu_2808 otu_109956 otu_142343<br>otu_5424 otu_908 otu_80232 otu_132912 otu_136923 otu_103395 otu_29791 otu_154585<br>otu_140948 otu_24962 otu_2752 otu_4901 otu_52097 otu_28078 otu_5446 otu_152482<br>otu_52422 otu_24706 otu_26435 otu_36179 otu_3711 otu_80421 otu_146394 otu_2327<br>otu_98328 otu_146536 otu_2133 otu_151132 otu_154429 otu_102812 otu_154391<br>otu_5085 otu_152675 otu_152896 otu_83806 otu_153648 otu_3111 otu_27616 otu_139703<br>otu_9776 otu_16285 otu_153115 otu_79384 otu_131542 otu_141681 otu_79060<br>otu_144069 otu_148509 otu_25141 otu_141038 otu_29255 otu_134411 otu_126345<br>otu_147381 otu_29055 otu_5581 otu_146466 otu_23896 otu_132774 otu_23232<br>otu_139519 otu_80212 otu_36419 otu_82042 otu_106446 otu_139611 otu_1284<br>otu_140830 otu_1595 otu_103184 otu_150317 otu_24569 otu_152133 otu_82308<br>otu_103244 otu_28234 otu_163314 otu_156732 otu_147051 otu_105699 otu_35989<br>otu_3621 otu_26955 otu_713 otu_145562 otu_146789 otu_108814 otu_35514 otu_80894<br>otu_2654 otu_5280 otu_99465 otu_139935 otu_142183 otu_163231 otu_33455 otu_1073<br>otu_4677 otu_106684 otu_62385 otu_154834 otu_28549 otu_30231 otu_2257 otu_126622<br>otu_61909 otu_30223 otu_6920 otu_3709 otu_2942 otu_83089 otu_108615 otu_105303<br>otu_105554 otu_132813 otu_34812 otu_127087 otu_32447 otu_23882 otu_97843<br>otu_144103 otu_148627 otu_126941 otu_152981 otu_82023 otu_1059 otu_134416<br>otu_99762 otu_22832 otu_109990 otu_79498 otu_153273 otu_110105 otu_6378<br>otu_151567 otu_154046 otu_35340 otu_1513 otu_22530 otu_35712 otu_83214 otu_80539<br>otu_99900 otu_33544 otu_151506 otu_134900 otu_105511 otu_141960 otu_127265<br>otu_79877 otu_147434 otu_3536 otu_80391 otu_140699 otu_37183 otu_28841<br>otu_134483 otu_150285 otu_26700 otu_146345 otu_2607 otu_9989 otu_152583<br>otu_32118 otu_32516 otu_146907 otu_106678 otu_126876 otu_82533 otu_26473<br>otu_31466 otu_83996 otu_82622 otu_36311 otu_100125 otu_1578 otu_90411 otu_96859<br>otu_5187 otu_153821 otu_137070 otu_146515 otu_9259 otu_126789 otu_82276<br>otu_146267 otu_126324 otu_23916 otu_136443 otu_32038 otu_99238 otu_150491<br>otu_137851 otu_132703 otu_5405 otu_132740 otu_138918 otu_83490 otu_30042<br>otu_150370 otu_1879 otu_139394 otu_145126 otu_33223 otu_107513 otu_138495<br>otu_22702 otu_153392 otu_2878 otu_110110 otu_140983 otu_22335 otu_144259 otu_3837<br>otu_105890 otu_79653 otu_142625 otu_137060 otu_140708 otu_25143 otu_2230<br>otu_35694 otu_3103 otu_146547 otu_143851 otu_103316 otu_126773 otu_5726<br>otu_147262 otu_34928
mpalafl mpolyiv	3	otu_152568 otu_152811 otu_154298
mpalaiv mpolyfl	1	otu_40398
mpolyfl soils	1842	otu_86002 otu_155479 otu_129028 otu_21384 otu_8424 otu_111475 otu_147694<br>otu_115354 otu_37365 otu_18576 otu_111301 otu_38803 otu_110670 otu_39169<br>otu_84311 otu_85058 otu_139477 otu_60172 otu_114978 otu_9133 otu_85023 otu_40197<br>otu_112170 otu_155007 otu_112069 otu_103902 otu_39298 otu_37880 otu_37825<br>otu_8263 otu_128763 otu_111588 otu_21215 otu_41861 otu_103722 otu_84539<br>otu_100220 otu_105190 otu_114210 otu_111027 otu_113590 otu_113838 otu_84353<br>otu_112595 otu_103678 otu_40420 otu_38734 otu_8583 otu_84509 otu_114869 otu_38738<br>otu_147518 otu_104678 otu_43732 otu_114327 otu_84574 otu_84702 otu_144583<br>otu_114168 otu_114550 otu_59726 otu_7617 otu_43547 otu_111464 otu_1525 otu_155413<br>otu_202 otu_44041 otu_8062 otu_137903 otu_41424 otu_155291 otu_142763 otu_114944<br>otu_163442 otu_20519 otu_139275 otu_7783 otu_7739 otu_154999 otu_147745<br>otu_155152 otu_85014 otu_100264 otu_115180 otu_54203 otu_111958 otu_112312<br>otu_43062 otu_155869 otu_142723 otu_98739 otu_1680 otu_37932 otu_100217<br>otu_139938 otu_41098 otu_100364 otu_37949 otu_112579 otu_16919 otu_84333<br>otu_44102 otu_128182 otu_140075 otu_128151 otu_40274 otu_155857 otu_111100<br>otu_7713 otu_41739 otu_84921 otu_997 otu_105111 otu_84714 otu_39990 otu_37296<br>otu_39940 otu_40913 otu_111359 otu_104988 otu_84675 otu_8861 otu_147555<br>otu_100359 otu_38078 otu_38735 otu_97285 otu_41547 otu_100426 otu_113790<br>otu_40536 otu_40267 otu_104767 otu_155120 otu_50095 otu_43167 otu_42443<br>otu_111574 otu_104096 otu_111396 otu_156173 otu_156349 otu_112204 otu_155921<br>otu_111651 otu_7923 otu_104616 otu_138201 otu_39733 otu_147523 otu_100318<br>otu_41353 otu_113040 otu_85863 otu_155271 otu_110696 otu_19042 otu_16459<br>otu_98518 otu_55326 otu_155251 otu_7493 otu_142735 otu_113876 otu_84324 otu_8991<br>otu_155111 otu_111098 otu_103830 otu_155057 otu_115344 otu_128411 otu_41780<br>otu_155349 otu_37395 otu_41217 otu_41743 otu_85556 otu_140055 otu_41894<br>otu_86022 otu_115286 otu_37504 otu_141173 otu_39236 otu_133181 otu_111783<br>otu_40436 otu_115260 otu_100189 otu_17596 otu_142877 otu_140416 otu_38580<br>otu_37544 otu_155284 otu_141188 otu_155403 otu_39314 otu_7543 otu_128311<br>otu_155351 otu_39686 otu_7757 otu_155460 otu_133270 otu_43631 otu_112490<br>otu_111392 otu_40564 otu_111113 otu_112300 otu_140032 otu_154931 otu_155416<br>otu_114241 otu_7640 otu_160192 otu_105083 otu_112684 otu_38968 otu_133931<br>otu_41167 otu_1301 otu_128279 otu_155422 otu_37246 otu_111335 otu_8554 otu_84386<br>otu_39727 otu_11974 otu_128591 otu_110692 otu_104336 otu_103791 otu_41494<br>otu_112152 otu_111781 otu_7496 otu_84529 otu_128577 otu_156352 otu_142856<br>otu_37817 otu_113912 otu_155441 otu_8488 otu_141164 otu_38107 otu_84454 otu_41285<br>otu_8239 otu_133308 otu_41176 otu_8510 otu_37914 otu_110668 otu_965 otu_147546<br>otu_156533 otu_163699 otu_113024 otu_38199 otu_100416 otu_143687 otu_37347<br>otu_38604 otu_43016 otu_111450 otu_155211 otu_112107 otu_38651 otu_8609<br>otu_112563 otu_104110 otu_38748 otu_43644 otu_37584 otu_141240 otu_156192<br>otu_111584 otu_37539 otu_155428 otu_104086 otu_128838 otu_128532 otu_154921<br>otu_43092 otu_139961 otu_38510 otu_38132 otu_39078 otu_18630 otu_40421 otu_38925<br>otu_37309 otu_37479 otu_85888 otu_100412 otu_122283 otu_39485 otu_8407<br>otu_103831 otu_98204 otu_142750 otu_155097 otu_128527 otu_128617 otu_114408<br>otu_155976 otu_104035 otu_141258 otu_155719 otu_141746 otu_163454 otu_37610<br>otu_112021 otu_42982 otu_40201 otu_85612 otu_155090 otu_147755 otu_104903<br>otu_41166 otu_7625 otu_144573 otu_39867 otu_41751 otu_100342 otu_113573 otu_85225<br>otu_103728 otu_111778 otu_104833 otu_112085 otu_42357 otu_155053 otu_111025<br>otu_7516 otu_149677 otu_111057 otu_9031 otu_41312 otu_40593 otu_8063 otu_84530<br>otu_112940 otu_155104 otu_8447 otu_37929 otu_40972 otu_43812 otu_8497 otu_85095<br>otu_17494 otu_137920 otu_41697 otu_103748 otu_8429 otu_156297 otu_113692<br>otu_147764 otu_38012 otu_144566 otu_111297 otu_115285 otu_8110 otu_39471<br>otu_140056 otu_16756 otu_142828 otu_128118 otu_13283 otu_7912 otu_110960 otu_8054<br>otu_41064 otu_104074 otu_37511 otu_42989 otu_37414 otu_154959 otu_37680<br>otu_115351 otu_64253 otu_142802 otu_100396 otu_8411 otu_147692 otu_8048<br>otu_149446 otu_103991 otu_7831 otu_38642 otu_41170 otu_42096 otu_110814<br>otu_155511 otu_139949 otu_104722 otu_43882 otu_40991 otu_111185 otu_155017<br>otu_115313 otu_155527 otu_133288 otu_18125 otu_42753 otu_128302 otu_8245<br>otu_128204 otu_100327 otu_7601 otu_8028 otu_7579 otu_147718 otu_7594 otu_7897<br>otu_8328 otu_9021 otu_144618 otu_97295 otu_8140 otu_112865 otu_43107 otu_113970

otu_112940 otu_155104 otu_8447 otu_37929 otu_40972 otu_43812 otu_8497 otu_85095
otu_17494 otu_137920 otu_41697 otu_103748 otu_8429 otu_156297 otu_113692
otu_147764 otu_38012 otu_144566 otu_111297 otu_115285 otu_8110 otu_39471
otu_140056 otu_16756 otu_142828 otu_128118 otu_13283 otu_7912 otu_110960 otu_8054
otu_41064 otu_104074 otu_37511 otu_42989 otu_37414 otu_154959 otu_37680
otu_115351 otu_64253 otu_142802 otu_100396 otu_8411 otu_147692 otu_8048
otu_149446 otu_103991 otu_7831 otu_38642 otu_41170 otu_42096 otu_110814
otu_155511 otu_139949 otu_104722 otu_43882 otu_40991 otu_111185 otu_155017
otu_115313 otu_155527 otu_133288 otu_18125 otu_42753 otu_128302 otu_8245
otu_128204 otu_100327 otu_7601 otu_8028 otu_7579 otu_147718 otu_7594 otu_7897
otu_8328 otu_9021 otu_144618 otu_97295 otu_8140 otu_112865 otu_43107 otu_113970
otu_144682 otu_85236 otu_37879 otu_114268 otu_133191 otu_114335 otu_141680
otu_115446 otu_39041 otu_155329 otu_112558 otu_8173 otu_142954 otu_145381
otu_129058 otu_112814 otu_128142 otu_43108 otu_43549 otu_113113 otu_103962
otu_38504 otu_154949 otu_7474 otu_48653 otu_103844 otu_37534 otu_38575 otu_8887
otu_38880 otu_155690 otu_103827 otu_84677 otu_7910 otu_41704 otu_147572
otu_115301 otu_112039 otu_155368 otu_84704 otu_84341 otu_100194 otu_103802
otu_10134 otu_114946 otu_43785 otu_128242 otu_37248 otu_39071 otu_7435 otu_85205
otu_37836 otu_20593 otu_40969 otu_112243 otu_144608 otu_40907 otu_105090
otu_144599 otu_111748 otu_111096 otu_100435 otu_113207 otu_155564 otu_39566
otu_84402 otu_100258 otu_114090 otu_105153 otu_137918 otu_8176 otu_113023
otu_65800 otu_111043 otu_1742 otu_37418 otu_41886 otu_19583 otu_113667 otu_84724
otu_111108 otu_128758 otu_159444 otu_8224 otu_84294 otu_39656 otu_111005 otu_911
otu_134824 otu_113037 otu_155005 otu_37991 otu_42641 otu_154927 otu_147668
otu_37271 otu_115375 otu_114244 otu_38018 otu_85228 otu_40683 otu_7862 otu_128274
otu_100309 otu_116232 otu_110832 otu_85376 otu_112849 otu_18686 otu_139944
otu_115191 otu_42045 otu_110912 otu_8316 otu_39284 otu_38341 otu_155699
otu_112992 otu_155840 otu_128920 otu_43835 otu_84755 otu_104320 otu_147566
otu_85668 otu_113512 otu_147754 otu_155568 otu_7653 otu_128523 otu_113585
otu_142860 otu_145313 otu_137358 otu_155059 otu_119918 otu_38889 otu_7812
otu_39593 otu_113176 otu_84751 otu_137923 otu_105025 otu_114247 otu_104013
otu_19894 otu_154958 otu_134829 otu_7619 otu_43964 otu_155731 otu_155085
otu_39776 otu_139295 otu_111603 otu_114162 otu_41900 otu_84636 otu_128735
otu_39717 otu_8064 otu_138656 otu_142885 otu_1926 otu_40358 otu_155054 otu_8875
otu_103824 otu_41733 otu_9022 otu_155812 otu_149599 otu_37597 otu_41521
otu_111967 otu_42310 otu_9452 otu_141267 otu_142906 otu_113839 otu_84517
otu_85484 otu_2093 otu_38327 otu_7904 otu_84967 otu_39897 otu_38608 otu_988
otu_39783 otu_38616 otu_144580 otu_7489 otu_38021 otu_37738 otu_40303 otu_111770
otu_157677 otu_148911 otu_155964 otu_111592 otu_115453 otu_111541 otu_141224
otu_147652 otu_155266 otu_128743 otu_155204 otu_155236 otu_43726 otu_38673
otu_113895 otu_139964 otu_931 otu_113913 otu_86051 otu_41752 otu_116377
otu_103790 otu_42776 otu_147649 otu_113332 otu_40448 otu_42574 otu_37526
otu_110836 otu_147837 otu_37632 otu_100197 otu_110890 otu_160882 otu_112722
otu_40354 otu_39056 otu_141201 otu_85158 otu_7574 otu_141272 otu_144633
otu_110993 otu_18374 otu_145270 otu_38320 otu_40650 otu_41911 otu_7818 otu_1881
otu_128158 otu_155086 otu_39610 otu_38501 otu_37912 otu_37425 otu_84872
otu_100367 otu_42948 otu_42496 otu_41239 otu_139995 otu_147693 otu_103650
otu_103733 otu_84949 otu_42589 otu_85112 otu_1757 otu_37368 otu_147757 otu_114108
otu_139272 otu_7975 otu_9156 otu_155279 otu_1939 otu_38519 otu_139958 otu_100273
otu_113820 otu_38020 otu_128223 otu_111004 otu_111549 otu_147648 otu_128359
otu_1932 otu_7498 otu_112695 otu_112916 otu_103826 otu_114556 otu_38563
otu_134857 otu_155753 otu_37700 otu_37568 otu_39565 otu_111804 otu_155632
otu_155717 otu_942 otu_43996 otu_43527 otu_39318 otu_144659 otu_43548 otu_136788
otu_38233 otu_155442 otu_39369 otu_85549 otu_155260 otu_141193 otu_18509
otu_139966 otu_115511 otu_85248 otu_112683 otu_141736 otu_111011 otu_155584
otu_7505 otu_129149 otu_110779 otu_37732 otu_110723 otu_112000 otu_37387
otu_113856 otu_38659 otu_104093 otu_113285 otu_1394 otu_8710 otu_41967 otu_85170
otu_112823 otu_7603 otu_112097 otu_16702 otu_84318 otu_119455 otu_7611 otu_144728
otu_84450 otu_147749 otu_8665 otu_133242 otu_141172 otu_8041 otu_98895 otu_7776
otu_85159 otu_85002 otu_84346 otu_149365 otu_155864 otu_38987 otu_103717
otu_39502 otu_111187 otu_43908 otu_8527 otu_38831 otu_128197 otu_38703 otu_147556
otu_147575 otu_8633 otu_111370 otu_155004 otu_66781 otu_38528 otu_134839
otu_112036 otu_128630 otu_104751 otu_110806 otu_111912 otu_103785 otu_112963
otu_38299 otu_115030 otu_114337 otu_8006 otu_19135 otu_9127 otu_38129 otu_111546
otu_7476 otu_7584 otu_38353 otu_43668 otu_131817 otu_128166 otu_7529 otu_113533
otu_42206 otu_114870 otu_103723 otu_97834 otu_150001 otu_139269 otu_122332
otu_155147 otu_139265 otu_100265 otu_110738 otu_142745 otu_38167 otu_156255
otu_110898 otu_115294 otu_113926 otu_8097 otu_114300 otu_113984 otu_154932
otu_115108 otu_42612 otu_8598 otu_111902 otu_110702 otu_84650 otu_103823
otu_156292 otu_39358 otu_8810 otu_111734 otu_147676 otu_38059 otu_40644 otu_38962
otu_128138 otu_39852 otu_7716 otu_39022 otu_7542 otu_155032 otu_149378 otu_40746
otu_41587 otu_112105 otu_113793 otu_104859 otu_84804 otu_39322 otu_37980
otu_43757 otu_147643 otu_112620 otu_129179 otu_104885 otu_128416 otu_39125
otu_114000 otu_7798 otu_1016 otu_145283 otu_141269 otu_141185 otu_44068 otu_37586
otu_112339 otu_37513 otu_40046 otu_7437 otu_8850 otu_111094 otu_128578 otu_105115
otu_42690 otu_39384 otu_7919 otu_114109 otu_128078 otu_110778 otu_100235
otu_39390 otu_37552 otu_37250 otu_7508 otu_39168 otu_139266 otu_156387
otu_110776 otu_155323 otu_137889 otu_7620 otu_155020 otu_8740 otu_38043
otu_133218 otu_9168 otu_42634 otu_114033 otu_112388 otu_114650 otu_142956
otu_8867 otu_137371 otu_84610 otu_147686 otu_38991 otu_37434 otu_9124 otu_158009
otu_8857 otu_38506 otu_84502 otu_113831 otu_149408 otu_113192 otu_155015
otu_104036 otu_37602 otu_155275 otu_39124 otu_7536 otu_105276 otu_155798
otu_111986 otu_128148 otu_42415 otu_37264 otu_40699 otu_138642 otu_155220
otu_84790 otu_85315 otu_41344 otu_113298 otu_43770 otu_149861 otu_113075
otu_97257 otu_133207 otu_113017 otu_114557 otu_128452 otu_141199 otu_139271
otu_39548 otu_7659 otu_112688 otu_43050 otu_155814 otu_115330 otu_111289
otu_128206 otu_85944 otu_7875 otu_114294 otu_84602 otu_142911 otu_110769
otu_147841 otu_142810 otu_155725 otu_42559 otu_140025 otu_112588 otu_7575
otu_147700 otu_37426 otu_129180 otu_137350 otu_7588 otu_17082 otu_110875
otu_113556 otu_42103 otu_98614 otu_85849 otu_8040 otu_103928 otu_57886 otu_111571
otu_41681 otu_137897 otu_7928 otu_998 otu_7992 otu_114740 otu_100400 otu_129131
otu_104022 otu_38180 otu_97264 otu_110857 otu_110866 otu_128557 otu_85244
otu_110700 otu_41242 otu_37411 otu_110725 otu_43592 otu_114681 otu_113004
otu_111568 otu_40895 otu_115350 otu_155999 otu_129033 otu_138202 otu_85872
otu_8410 otu_38121 otu_43557 otu_8012 otu_155657 otu_128096 otu_145433 otu_38529
otu_113695 otu_147687 otu_103837 otu_8928 otu_44058 otu_156486 otu_112183
otu_110751 otu_8587 otu_85013 otu_155238 otu_103655 otu_144626 otu_37792
otu_84980 otu_42097 otu_100352 otu_37819 otu_113884 otu_105225 otu_115154
otu_84315 otu_100343 otu_111066 otu_145319 otu_114086 otu_39596 otu_84688
otu_111401 otu_85434 otu_39347 otu_7891 otu_137383 otu_8846 otu_110795 otu_147673
otu_84769 otu_39116 otu_113904 otu_100389 otu_104051 otu_142803 otu_39383
otu_120832 otu_147514 otu_84646 otu_7480 otu_111490 otu_40794 otu_147536 otu_1772
otu_37289 otu_103988 otu_155382 otu_1707 otu_145289 otu_39102 otu_128636
otu_113559 otu_8821 otu_120405 otu_155636 otu_41914 otu_43472 otu_114361
otu_43439 otu_8152 otu_40330 otu_917 otu_111204 otu_86055 otu_39515 otu_111125
otu_44091 otu_19518 otu_37605 otu_144565 otu_111272 otu_112332 otu_155134
otu_39923 otu_43255 otu_40400 otu_7593 otu_114695 otu_155342 otu_40431 otu_41413
otu_43953 otu_114808 otu_8099 otu_138207 otu_38340 otu_139289 otu_37824
otu_37318 otu_38788 otu_156489 otu_111330 otu_111293 otu_40976 otu_113927

otu_84980 otu_42097 otu_100352 otu_37819 otu_113884 otu_105225 otu_115154
otu_84315 otu_100343 otu_111066 otu_145319 otu_114086 otu_39596 otu_84688
otu_111401 otu_85434 otu_39347 otu_7891 otu_137383 otu_8846 otu_110795 otu_147673
otu_84769 otu_39116 otu_113904 otu_100389 otu_104051 otu_142803 otu_39383
otu_120832 otu_147514 otu_84646 otu_7480 otu_111490 otu_40794 otu_147536 otu_1772
otu_37289 otu_103988 otu_155382 otu_1707 otu_145289 otu_39102 otu_128636
otu_113559 otu_8821 otu_120405 otu_155636 otu_41914 otu_43472 otu_114361
otu_43439 otu_8152 otu_40330 otu_917 otu_111204 otu_86055 otu_39515 otu_111125
otu_44091 otu_19518 otu_37605 otu_144565 otu_111272 otu_112332 otu_155134
otu_39923 otu_43255 otu_40400 otu_7593 otu_114695 otu_155342 otu_40431 otu_41413
otu_43953 otu_114808 otu_8099 otu_138207 otu_38340 otu_139289 otu_37824
otu_37318 otu_38788 otu_156489 otu_111330 otu_111293 otu_40976 otu_113927
otu_155154 otu_144827 otu_40847 otu_154998 otu_112693 otu_138097 otu_128084
otu_39917 otu_142927 otu_42125 otu_41357 otu_9202 otu_85867 otu_105216
otu_112242 otu_38206 otu_110734 otu_41465 otu_137385 otu_42186 otu_113727
otu_37756 otu_84354 otu_41589 otu_8824 otu_111933 otu_41566 otu_128488 otu_104076
otu_155246 otu_128787 otu_104794 otu_110847 otu_111523 otu_9134 otu_147729
otu_8849 otu_139300 otu_41392 otu_8521 otu_156268 otu_1888 otu_84901 otu_97269
otu_8781 otu_44212 otu_85523 otu_38489 otu_8548 otu_103703 otu_8358 otu_128200
otu_8538 otu_8451 otu_37467 otu_128233 otu_8776 otu_104543 otu_145565 otu_937
otu_142726 otu_156231 otu_8544 otu_155947 otu_133235 otu_128263 otu_155302
otu_38714 otu_37488 otu_39714 otu_139945 otu_8604 otu_7519 otu_111118 otu_9368
otu_8828 otu_155908 otu_84713 otu_103753 otu_37540 otu_155343 otu_147529
otu_104106 otu_18500 otu_7468 otu_103995 otu_147695 otu_155170 otu_37551
otu_85709 otu_63958 otu_156426 otu_42878 otu_10664 otu_38032 otu_7626 otu_113334
otu_98867 otu_43140 otu_38672 otu_38822 otu_916 otu_113612 otu_155270 otu_155013
otu_41670 otu_91097 otu_111310 otu_39924 otu_38231 otu_141209 otu_144560
otu_37884 otu_144672 otu_39960 otu_40359 otu_8085 otu_112897 otu_84302 otu_37566
otu_7478 otu_84756 otu_100302 otu_148234 otu_8269 otu_43281 otu_137607 otu_38494
otu_141248 otu_84631 otu_128237 otu_60165 otu_38660 otu_37389 otu_38509
otu_113686 otu_115192 otu_103848 otu_84717 otu_142791 otu_7503 otu_114701
otu_104052 otu_156131 otu_112789 otu_100180 otu_97288 otu_55969 otu_43840
otu_112457 otu_38256 otu_155352 otu_114922 otu_19186 otu_155464 otu_111943
otu_38783 otu_100237 otu_139327 otu_42411 otu_7754 otu_155576 otu_39761
otu_147688 otu_38312 otu_118728 otu_112201 otu_41864 otu_7439 otu_110876
otu_142721 otu_37247 otu_85055 otu_112465 otu_39048 otu_41025 otu_128682
otu_84938 otu_41491 otu_111061 otu_134821 otu_113990 otu_8180 otu_8321 otu_39410
otu_104873 otu_8842 otu_111854 otu_133168 otu_137887 otu_41141 otu_38423
otu_39847 otu_112780 otu_41347 otu_111569 otu_42435 otu_43187 otu_114106
otu_39098 otu_140044 otu_114775 otu_113210 otu_43544 otu_128277 otu_111366
otu_128161 otu_84863 otu_37261 otu_19790 otu_42520 otu_42827 otu_42566
otu_113194 otu_37461 otu_141229 otu_39255 otu_155936 otu_40310 otu_147634
otu_985 otu_133173 otu_148286 otu_139959 otu_112030 otu_155318 otu_113296
otu_104511 otu_155174 otu_43360 otu_39340 otu_84369 otu_38544 otu_43214 otu_8221
otu_103820 otu_43271 otu_139941 otu_128094 otu_84873 otu_113617 otu_155200
otu_84830 otu_37580 otu_8272 otu_8167 otu_42571 otu_8129 otu_40107 otu_112186
otu_42252 otu_147625 otu_140023 otu_137899 otu_84552 otu_84975 otu_39918
otu_44105 otu_137378 otu_111102 otu_38179 otu_42327 otu_128671 otu_155230
otu_40865 otu_156158 otu_144680 otu_111654 otu_7642 otu_144817 otu_8468
otu_149531 otu_112176 otu_139952 otu_110694 otu_94244 otu_140035 otu_85869
otu_115274 otu_128081 otu_84815 otu_97613 otu_7823 otu_38792 otu_7467 otu_41905
otu_112682 otu_139990 otu_84655 otu_104828 otu_128119 otu_100192 otu_144753
otu_114687 otu_144781 otu_114802 otu_112113 otu_39767 otu_38436 otu_128194
otu_133163 otu_100272 otu_38840 otu_111294 otu_156296 otu_37818 otu_12185
otu_7681 otu_114537 otu_142931 otu_8391 otu_114005 otu_37957 otu_103980
otu_100222 otu_112403 otu_140063 otu_113220 otu_37501 otu_156213 otu_111713
otu_144808 otu_37567 otu_40808 otu_37520 otu_111810 otu_156117 otu_110889
otu_8101 otu_7750 otu_137664 otu_147645 otu_138648 otu_38002 otu_133183 otu_2059
otu_41708 otu_114842 otu_155222 otu_43659 otu_136789 otu_140057 otu_38378
otu_113060 otu_149109 otu_116716 otu_84630 otu_139943 otu_156208 otu_128773
otu_104089 otu_39339 otu_100402 otu_155855 otu_104914 otu_155959 otu_103927
otu_40129 otu_112510 otu_97294 otu_37774 otu_111562 otu_8467 otu_111701
otu_105121 otu_111265 otu_39570 otu_38238 otu_111083 otu_133169 otu_114658
otu_37944 otu_149805 otu_100390 otu_85638 otu_85289 otu_40562 otu_136170
otu_103772 otu_42101 otu_41455 otu_111866 otu_1510 otu_155560 otu_7995 otu_41289
otu_40158 otu_18898 otu_85069 otu_84416 otu_128268 otu_42746 otu_104252
otu_154944 otu_155183 otu_38166 otu_84641 otu_40843 otu_7502 otu_40393 otu_40009
otu_100369 otu_9419 otu_142825 otu_40022 otu_84772 otu_111834 otu_8730 otu_8719
otu_111127 otu_42841 otu_37342 otu_128451 otu_100295 otu_147639 otu_111935
otu_85629 otu_112091 otu_134830 otu_19344 otu_40670 otu_7448 otu_128576
otu_128971 otu_21036 otu_114873 otu_139971 otu_113674 otu_85074 otu_21538
otu_37965 otu_112456 otu_8312 otu_155273 otu_115553 otu_7925 otu_163912
otu_154977 otu_40227 otu_8580 otu_113238 otu_38075 otu_8268 otu_147540 otu_155113
otu_8981 otu_112044 otu_105223 otu_110893 otu_41896 otu_110899 otu_111031
otu_112159 otu_112464 otu_84520 otu_44015 otu_40966 otu_84651 otu_43818 otu_85094
otu_128248 otu_129026 otu_8508 otu_113991 otu_142772 otu_111412 otu_144677
otu_115102 otu_8473 otu_42332 otu_91337 otu_110716 otu_38444 otu_8901 otu_9059
otu_137370 otu_37663 otu_39619 otu_111208 otu_160646 otu_8575 otu_37941
otu_112234 otu_133321 otu_100216 otu_21268 otu_112621 otu_141177 otu_18114
otu_147526 otu_41709 otu_85532 otu_104121 otu_8903 otu_147898 otu_39629
otu_85555 otu_97302 otu_155529 otu_9123 otu_136779 otu_104990 otu_7514 otu_8512
otu_37394 otu_38707 otu_111875 otu_112163 otu_39957 otu_84732 otu_37499 otu_7917
otu_8720 otu_85824 otu_38209 otu_155335 otu_38956 otu_15929 otu_100276 otu_7665
otu_111859 otu_155506 otu_142920 otu_11842 otu_39373 otu_114518 otu_112535
otu_112469 otu_141207 otu_147690 otu_100204 otu_112543 otu_8933 otu_142765
otu_155009 otu_115476 otu_41287 otu_37659 otu_144805 otu_134850 otu_84846
otu_111059 otu_154954 otu_37667 otu_111796 otu_137351 otu_114339 otu_37254
otu_85177 otu_155952 otu_42962 otu_39290 otu_85770 otu_129211 otu_112437
otu_133174 otu_139977 otu_37776 otu_42005 otu_154915 otu_128579 otu_103983
otu_38801 otu_963 otu_7730 otu_154908 otu_111627 otu_63133 otu_139437 otu_128231
otu_39757 otu_8714 otu_155390 otu_155552 otu_103747 otu_8523 otu_103700
otu_16916 otu_38662 otu_67095 otu_155458 otu_62922 otu_154928 otu_147661
otu_85594 otu_40530 otu_8405 otu_112346 otu_136162 otu_8855 otu_111053 otu_58983
otu_9149 otu_8186 otu_38638 otu_43532 otu_129886 otu_42483 otu_128678 otu_38208
otu_142839 otu_104802 otu_110796 otu_85026 otu_155945 otu_7848 otu_43355
otu_60297 otu_39800 otu_1885 otu_42585 otu_113311 otu_141223 otu_8914 otu_110997
otu_147570 otu_147709 otu_9152 otu_85039 otu_100298 otu_111946 otu_163447
otu_8125 otu_114019 otu_39763 otu_38476 otu_40064 otu_112983 otu_155296
otu_103868 otu_8482 otu_97358 otu_37979 otu_155707 otu_104104 otu_7871
otu_128149 otu_142814 otu_140046 otu_7440 otu_37482 otu_128779 otu_37592
otu_142914 otu_41127 otu_111738 otu_141243 otu_155713 otu_7826 otu_104712
otu_43521 otu_983 otu_37380 otu_137667 otu_155491 otu_113657 otu_84328 otu_115186
otu_156594 otu_38419 otu_8423 otu_38150 otu_155267 otu_128388 otu_100406
otu_7501 otu_142793 otu_38893 otu_111278 otu_128082 otu_8409 otu_84389 otu_8147
otu_84845 otu_935 otu_111439 otu_104097 otu_128670 otu_112915 otu_44210
otu_140006 otu_84881 otu_110901 otu_38391 otu_128861 otu_110927 otu_128657
otu_7703 otu_37794 otu_133184 otu_156523 otu_128689 otu_156530 otu_8733
otu_37656 otu_8489 otu_138649

		[illegible] otu_103868 otu_8482 otu_97358 otu_37979 otu_155707 otu_104104 otu_7871 otu_128149 otu_142814 otu_140046 otu_7440 otu_37482 otu_128779 otu_37592 otu_142914 otu_41127 otu_111738 otu_141243 otu_155713 otu_7826 otu_104712 otu_43521 otu_983 otu_37380 otu_137667 otu_155491 otu_113657 otu_84328 otu_115186 otu_156594 otu_38419 otu_8423 otu_38150 otu_155267 otu_128388 otu_100406 otu_7501 otu_142793 otu_38893 otu_111278 otu_128082 otu_8409 otu_84389 otu_8147 otu_84845 otu_935 otu_111439 otu_104097 otu_128670 otu_112915 otu_44210 otu_140006 otu_84881 otu_110901 otu_38391 otu_128861 otu_110927 otu_128657 otu_7703 otu_37794 otu_133184 otu_156523 otu_128689 otu_156530 otu_8733 otu_37656 otu_8489 otu_138649
mpolyfl mpolyiv	8	otu_155136 otu_156411 otu_156245 otu_156494 otu_156180 otu_115369 otu_162968 otu_155151
mpalaiv soils	2	otu_86726 otu_48504
mpalaiv mpolyiv	172	otu_73656 otu_75424 otu_73630 otu_69438 otu_71469 otu_74100 otu_73050 otu_22294 otu_72767 otu_70997 otu_69313 otu_75182 otu_22289 otu_74320 otu_72542 otu_145872 otu_71141 otu_73682 otu_69901 otu_74732 otu_74697 otu_74436 otu_22073 otu_70843 otu_69747 otu_22285 otu_76313 otu_71282 otu_74837 otu_70027 otu_73433 otu_72180 otu_75677 otu_73947 otu_22260 otu_72286 otu_72132 otu_22136 otu_73904 otu_70564 otu_70038 otu_74156 otu_69455 otu_143766 otu_71691 otu_69874 otu_71225 otu_72183 otu_75839 otu_75162 otu_70702 otu_73361 otu_72090 otu_95498 otu_68901 otu_75980 otu_69007 otu_69349 otu_75890 otu_70186 otu_76185 otu_22087 otu_22296 otu_72623 otu_22278 otu_69637 otu_145666 otu_69237 otu_69459 otu_76600 otu_69337 otu_143735 otu_76512 otu_145785 otu_77537 otu_74267 otu_71079 otu_69255 otu_74685 otu_72316 otu_76376 otu_73912 otu_22256 otu_71592 otu_69271 otu_146183 otu_72112 otu_69090 otu_74506 otu_22299 otu_74771 otu_74484 otu_22290 otu_73955 otu_73295 otu_73659 otu_75454 otu_75082 otu_69304 otu_70487 otu_69737 otu_76383 otu_71119 otu_70144 otu_72774 otu_74427 otu_71091 otu_71815 otu_75154 otu_72710 otu_74770 otu_71627 otu_145827 otu_22153 otu_74165 otu_71775 otu_76064 otu_70414 otu_70854 otu_68941 otu_74743 otu_70300 otu_22169 otu_22279 otu_22269 otu_69613 otu_73052 otu_74176 otu_71205 otu_73877 otu_73259 otu_70417 otu_163155 otu_73653 otu_72539 otu_74354 otu_73165 otu_74998 otu_73462 otu_22263 otu_145806 otu_75250 otu_70201 otu_71177 otu_74333 otu_73909 otu_145687 otu_75569 otu_70128 otu_70032 otu_74002 otu_78404 otu_72952 otu_71274 otu_73622 otu_73471 otu_74767 otu_75516 otu_71734 otu_74236 otu_75437 otu_73566 otu_73728 otu_71607 otu_70504 otu_72796 otu_69162 otu_75090 otu_76204 otu_74560 otu_22270 otu_75584
mpolyiv soils	10	otu_159770 otu_129282 otu_45463 otu_159997 otu_52948 otu_54071 otu_162571 otu_57872 otu_161060 otu_2097
mpalafl	5362	otu_81481 otu_105782 otu_27706 otu_24272 otu_4798 otu_132621 otu_80658 otu_132734 otu_108808 otu_96902 otu_33454 otu_5282 otu_83492 otu_146416 otu_151050 otu_141860 otu_151984 otu_81055 otu_106416 otu_153665 otu_150843 otu_96887 otu_6816 otu_25323 otu_108621 otu_28100 otu_146381 otu_33850 otu_25366 otu_139563 otu_4533 otu_108610 otu_82583 otu_146298 otu_138162 otu_79858 otu_139764 otu_28402 otu_108227 otu_152289 otu_106609 otu_151351 otu_3982 otu_137628 otu_81072 otu_80168 otu_26873 otu_30317 otu_3690 otu_80844 otu_31994 otu_106513 otu_80477 otu_28750 otu_22792 otu_141856 otu_134629 otu_146992 otu_146821 otu_132709 otu_82932 otu_140698 otu_28716 otu_103254 otu_141785 otu_36787 otu_31117 otu_106475 otu_136962 otu_82273 otu_106437 otu_24125 otu_5242 otu_139755 otu_25382 otu_105663 otu_24187 otu_2719 otu_23717 otu_105960 otu_32252 otu_105842 otu_151697 otu_4757 otu_32539 otu_133040 otu_105898 otu_5020 otu_5022 otu_152565 otu_107431 otu_132513 otu_33067 otu_107150 otu_81998 otu_80155 otu_153485 otu_33639 otu_24116 otu_153110 otu_106795 otu_106559 otu_106781 otu_23509 otu_2245 otu_23981 otu_31581 otu_107477 otu_139705 otu_3002 otu_30718 otu_79381 otu_80780 otu_453 otu_106028 otu_150563 otu_150687 otu_126553 otu_7050 otu_657 otu_107754 otu_29478 otu_150720 otu_37013 otu_107723 otu_108496 otu_139234 otu_28553 otu_26324 otu_32657 otu_96847 otu_151062 otu_7047 otu_80069 otu_150824 otu_25031 otu_150832 otu_82725 otu_4477 otu_81290 otu_32559 otu_154028 otu_126404 otu_514 otu_5842 otu_127214 otu_36700 otu_79924 otu_146461 otu_30898 otu_152377 otu_132777 otu_4059 otu_107813 otu_29755 otu_2261 otu_22527 otu_139576 otu_26693 otu_4139 otu_106568 otu_28326 otu_109314 otu_106595 otu_6376 otu_23248 otu_106459 otu_4364 otu_139535 otu_105415 otu_136707 otu_22477 otu_23011 otu_154804 otu_31278 otu_35359 otu_142399 otu_22667 otu_27804 otu_691 otu_151143 otu_25568 otu_82438 otu_33533 otu_3255 otu_28113 otu_105459 otu_126473 otu_151138 otu_139772 otu_139554 otu_138413 otu_152576 otu_151520 otu_33442 otu_151551 otu_25135 otu_127436 otu_154310 otu_26310 otu_5232 otu_2551 otu_99628 otu_30045 otu_80453 otu_127190 otu_28604 otu_105908 otu_108294 otu_7208 otu_27792 otu_143775 otu_80397 otu_140723 otu_29741 otu_28548 otu_126878 otu_108887 otu_27969 otu_5861 otu_81278 otu_80582 otu_4325 otu_105446 otu_126731 otu_2788 otu_33435 otu_105553 otu_22372 otu_4457 otu_153364 otu_96747 otu_106519 otu_147496 otu_6683 otu_105647 otu_136725 otu_150468 otu_4622 otu_103328 otu_34994 otu_152775 otu_2217 otu_83405 otu_126306 otu_150976 otu_30675 otu_146991 otu_23447 otu_142543 otu_126296 otu_80348 otu_78996 otu_147040 otu_3045 otu_26660 otu_107208 otu_80374 otu_6086 otu_107377 otu_29039 otu_5476 otu_81134 otu_27915 otu_22619 otu_127068 otu_29901 otu_152373 otu_2298 otu_28224 otu_79086 otu_108210 otu_103343 otu_29816 otu_107003 otu_22681 otu_79642 otu_2907 otu_804 otu_152403 otu_141902 otu_108710 otu_105858 otu_147481 otu_4360 otu_81143 otu_82900 otu_150258 otu_105792 otu_107456 otu_96818 otu_33290 otu_34094 otu_4548 otu_32054 otu_109226 otu_679 otu_134500 otu_106216 otu_110126 otu_2868 otu_30764 otu_110329 otu_82875 otu_106324 otu_22461 otu_31625 otu_81043 otu_99280 otu_105831 otu_134642 otu_103036 otu_4569 otu_142518 otu_82888 otu_139802 otu_27833 otu_102813 otu_142306 otu_23485 otu_28429 otu_107412 otu_80002 otu_27954 otu_4224 otu_152313 otu_144096 otu_109822 otu_36304 otu_107305 otu_106907 otu_6045 otu_5992 otu_126157 otu_107995 otu_139181 otu_23329 otu_2142 otu_103064 otu_152562 otu_150839 otu_4145 otu_35454 otu_82818 otu_34608 otu_140980 otu_79098 otu_103453 otu_3486 otu_30583 otu_137608 otu_142482 otu_80974 otu_25249 otu_4709 otu_82552 otu_79443 otu_150452 otu_2764 otu_25659 otu_137309 otu_102952 otu_105436 otu_32591 otu_24902 otu_27380 otu_33889 otu_23340 otu_24489 otu_107502 otu_96919 otu_152767 otu_28061 otu_106025 otu_22740 otu_109977 otu_25063 otu_24166 otu_107846 otu_99334 otu_150324 otu_109151 otu_29261 otu_150662 otu_99793 otu_108263 otu_6633 otu_106543 otu_2454 otu_24472 otu_4028 otu_140691 otu_5508 otu_106155 otu_150915 otu_32173 otu_144013 otu_109010 otu_30010 otu_150897 otu_2464 otu_33001 otu_103609 otu_29002 otu_103336 otu_26497 otu_107482 otu_23915 otu_142026 otu_5676 otu_143831 otu_108868 otu_105410 otu_132549 otu_27148 otu_7213 otu_151741 otu_82532 otu_153774 otu_152501 otu_126197 otu_3887 otu_146434 otu_107741 otu_3987 otu_126282 otu_97128 otu_126357 otu_138534 otu_134486 otu_22579 otu_24444 otu_142048 otu_32229 otu_24876 otu_105766 otu_152153 otu_30119 otu_152495 otu_79028 otu_81168 otu_150969 otu_107131 otu_102861 otu_24141 otu_153263 otu_3193 otu_6810 otu_141884 otu_22455 otu_3591 otu_142232 otu_31434 otu_150595 otu_35830 otu_80167 otu_3473 otu_33308 otu_152977 otu_28064 otu_78970 otu_26958 otu_141835 otu_2248 otu_81285 otu_106979 otu_151265 otu_106383 otu_35165 otu_27025 otu_6434 otu_79609 otu_109804 otu_80531 otu_103475 otu_28689 otu_32901 otu_108573 otu_151934 otu_7009 otu_103522 otu_83636 otu_105799 otu_154554 otu_154867 otu_25125 otu_137267 otu_126519

otu_103609 otu_29002 otu_103336 otu_26497 otu_107482 otu_23915 otu_142026
otu_5676 otu_143831 otu_108868 otu_105410 otu_132549 otu_27148 otu_7213
otu_151741 otu_82532 otu_153774 otu_152501 otu_126197 otu_3887 otu_146434
otu_107741 otu_3987 otu_126282 otu_97128 otu_126357 otu_138534 otu_134486
otu_22579 otu_24444 otu_142048 otu_32229 otu_24876 otu_105766 otu_152153
otu_30119 otu_152495 otu_79028 otu_81168 otu_150969 otu_107131 otu_102861
otu_24141 otu_153263 otu_3193 otu_6810 otu_141884 otu_22455 otu_3591 otu_142232
otu_31434 otu_150595 otu_35830 otu_80167 otu_3473 otu_33308 otu_152977 otu_28064
otu_78970 otu_26958 otu_141835 otu_2248 otu_81285 otu_106979 otu_151265
otu_106383 otu_35165 otu_27025 otu_6434 otu_79609 otu_109804 otu_80531
otu_103475 otu_28689 otu_32901 otu_108573 otu_151934 otu_7009 otu_103522
otu_83636 otu_105799 otu_154554 otu_154867 otu_25125 otu_137267 otu_126519
otu_22797 otu_79166 otu_79526 otu_100105 otu_22793 otu_25483 otu_81625 otu_27573
otu_27298 otu_3671 otu_28184 otu_151517 otu_150577 otu_81561 otu_79043
otu_139550 otu_79386 otu_79066 otu_22943 otu_103552 otu_411 otu_79184 otu_134615
otu_24839 otu_5920 otu_4274 otu_82001 otu_105688 otu_2742 otu_30846 otu_106276
otu_29029 otu_23906 otu_2110 otu_127677 otu_103363 otu_132610 otu_22730
otu_152446 otu_106418 otu_4908 otu_163913 otu_31387 otu_103152 otu_152425
otu_27686 otu_137074 otu_107827 otu_83543 otu_33509 otu_127894 otu_151129
otu_4305 otu_81419 otu_2688 otu_81455 otu_103140 otu_106184 otu_132793
otu_141845 otu_28299 otu_3141 otu_22755 otu_82854 otu_151629 otu_106128
otu_127738 otu_24004 otu_79454 otu_146700 otu_79452 otu_126636 otu_150381
otu_106751 otu_150807 otu_33977 otu_27619 otu_2815 otu_32109 otu_80595 otu_23320
otu_35877 otu_5306 otu_108323 otu_105493 otu_106118 otu_23499 otu_151413
otu_126812 otu_33864 otu_32737 otu_146538 otu_132456 otu_31178 otu_25555
otu_152217 otu_4960 otu_2614 otu_126435 otu_24036 otu_107358 otu_150927 otu_6660
otu_22399 otu_4897 otu_126206 otu_146898 otu_34696 otu_150872 otu_33429
otu_29221 otu_37052 otu_106073 otu_163241 otu_150166 otu_105998 otu_128052
otu_30339 otu_24580 otu_24656 otu_29919 otu_143903 otu_103466 otu_151029
otu_24707 otu_31223 otu_32780 otu_107842 otu_99859 otu_6954 otu_146523 otu_82754
otu_24911 otu_3778 otu_23807 otu_150089 otu_108046 otu_29556 otu_99667 otu_79834
otu_27373 otu_27438 otu_107942 otu_150610 otu_126153 otu_79491 otu_134407
otu_153489 otu_26791 otu_81325 otu_29648 otu_150909 otu_34951 otu_126754
otu_32942 otu_134508 otu_23834 otu_36950 otu_106197 otu_80098 otu_154889
otu_2616 otu_83830 otu_32858 otu_23349 otu_4728 otu_83036 otu_5073 otu_153044
otu_126734 otu_79081 otu_82255 otu_25297 otu_150793 otu_28185 otu_151769
otu_25563 otu_106695 otu_150781 otu_142527 otu_2843 otu_127101 otu_144325
otu_3247 otu_25284 otu_29164 otu_151700 otu_22652 otu_127580 otu_477 otu_146241
otu_37125 otu_144223 otu_108731 otu_126248 otu_96988 otu_27013 otu_106021
otu_127601 otu_102984 otu_126690 otu_142497 otu_153901 otu_140717 otu_2186
otu_27600 otu_106072 otu_154649 otu_2443 otu_6126 otu_150795 otu_29854 otu_27414
otu_2692 otu_80423 otu_132483 otu_109924 otu_106195 otu_25665 otu_3958
otu_103197 otu_5272 otu_36346 otu_140905 otu_142189 otu_26557 otu_24268
otu_24620 otu_28279 otu_2191 otu_151937 otu_24345 otu_79040 otu_109620 otu_28599
otu_150768 otu_29222 otu_28201 otu_28698 otu_154859 otu_2745 otu_137262
otu_126747 otu_28254 otu_147053 otu_153234 otu_99671 otu_150641 otu_152050
otu_153942 otu_142293 otu_106936 otu_2200 otu_151390 otu_146726 otu_6685
otu_35873 otu_132606 otu_23397 otu_150964 otu_26782 otu_24633 otu_22715
otu_152660 otu_4296 otu_139555 otu_105981 otu_82137 otu_154393 otu_144173
otu_150697 otu_485 otu_83116 otu_25625 otu_151646 otu_107919 otu_142623
otu_35432 otu_133009 otu_4446 otu_2501 otu_109625 otu_80565 otu_37119 otu_23217
otu_28124 otu_2125 otu_22872 otu_151367 otu_7258 otu_153595 otu_142496
otu_107511 otu_28672 otu_7236 otu_99564 otu_150474 otu_134532 otu_105416
otu_28617 otu_79307 otu_106677 otu_107042 otu_151159 otu_2980 otu_139916
otu_96789 otu_147006 otu_82570 otu_126877 otu_103610 otu_26734 otu_150748
otu_22729 otu_154040 otu_28547 otu_143838 otu_146433 otu_32943 otu_3890
otu_146932 otu_152873 otu_127135 otu_106756 otu_32799 otu_79714 otu_102912
otu_24831 otu_24759 otu_106902 otu_151590 otu_127824 otu_105461 otu_2216
otu_126811 otu_126801 otu_81307 otu_126720 otu_150774 otu_3534 otu_3783
otu_26304 otu_29237 otu_178 otu_5507 otu_28473 otu_106105 otu_23163 otu_27326
otu_142106 otu_151818 otu_142012 otu_81138 otu_137243 otu_79875 otu_81852
otu_146998 otu_79980 otu_26033 otu_105968 otu_151717 otu_23287 otu_27729
otu_82043 otu_25305 otu_105866 otu_151260 otu_22887 otu_29247 otu_136708
otu_107484 otu_26518 otu_109282 otu_23740 otu_139673 otu_83101 otu_107820
otu_144246 otu_22960 otu_102844 otu_31321 otu_2822 otu_79891 otu_5447 otu_108090
otu_28953 otu_31601 otu_2466 otu_26143 otu_28146 otu_4883 otu_144322 otu_106914
otu_25192 otu_152136 otu_80008 otu_34944 otu_143793 otu_139599 otu_22433
otu_81922 otu_152703 otu_7276 otu_27279 otu_146447 otu_137259 otu_108402
otu_31510 otu_152960 otu_878 otu_26023 otu_32625 otu_26078 otu_4422 otu_24295
otu_96697 otu_33475 otu_109701 otu_109844 otu_99869 otu_154029 otu_96676
otu_146242 otu_25612 otu_141974 otu_141879 otu_33759 otu_25689 otu_30057
otu_151896 otu_24191 otu_151208 otu_153170 otu_82757 otu_108374 otu_80878
otu_147471 otu_79217 otu_27737 otu_108255 otu_7302 otu_78942 otu_99846
otu_152341 otu_103164 otu_3572 otu_31527 otu_28554 otu_83008 otu_24435 otu_34823
otu_28038 otu_5292 otu_36384 otu_96868 otu_26218 otu_28981 otu_127111 otu_126910
otu_80784 otu_81320 otu_105744 otu_109782 otu_80640 otu_34687 otu_27095
otu_102808 otu_150883 otu_108848 otu_4300 otu_150405 otu_31563 otu_143978
otu_142224 otu_150693 otu_22604 otu_140839 otu_3266 otu_5083 otu_27740
otu_127141 otu_3498 otu_79798 otu_22661 otu_3014 otu_136714 otu_138422 otu_29576
otu_81118 otu_2119 otu_150386 otu_33030 otu_109846 otu_32886 otu_26914 otu_29119
otu_3539 otu_31535 otu_151565 otu_3058 otu_26491 otu_153990 otu_23876 otu_27716
otu_2490 otu_26489 otu_152653 otu_24186 otu_4556 otu_5302 otu_23474 otu_105664
otu_106123 otu_134352 otu_82910 otu_3474 otu_146778 otu_25183 otu_132844
otu_3178 otu_24167 otu_106805 otu_6575 otu_36897 otu_35411 otu_108793 otu_109967
otu_5911 otu_24462 otu_6703 otu_28397 otu_429 otu_139617 otu_24298 otu_139749
otu_31614 otu_137079 otu_22979 otu_109749 otu_24428 otu_107499 otu_127183
otu_99319 otu_80135 otu_103459 otu_3386 otu_150189 otu_26164 otu_7267 otu_144398
otu_146443 otu_79339 otu_147175 otu_4108 otu_82458 otu_3210 otu_23606 otu_109549
otu_150837 otu_150961 otu_25285 otu_78978 otu_28178 otu_150074 otu_32255 otu_456
otu_79761 otu_3787 otu_33300 otu_126350 otu_6668 otu_109104 otu_141816 otu_79951
otu_108924 otu_24677 otu_105581 otu_6320 otu_151602 otu_82840 otu_22485
otu_107320 otu_108411 otu_152317 otu_33490 otu_80564 otu_126189 otu_24600
otu_29767 otu_35861 otu_126578 otu_99681 otu_152347 otu_140798 otu_109953
otu_158 otu_99980 otu_33488 otu_24873 otu_153681 otu_31300 otu_22522 otu_107077
otu_105398 otu_4333 otu_83294 otu_4084 otu_4620 otu_505 otu_105697 otu_25512
otu_150334 otu_22842 otu_81436 otu_4655 otu_36460 otu_103186 otu_24324 otu_23198
otu_126475 otu_81574 otu_126793 otu_136745 otu_150401 otu_79122 otu_30710
otu_147145 otu_27595 otu_127045 otu_139648 otu_150763 otu_127079 otu_28701
otu_29274 otu_4324 otu_151618 otu_28949 otu_151881 otu_82588 otu_163994
otu_150684 otu_152140 otu_99368 otu_152110 otu_107579 otu_82192 otu_133074
otu_106771 otu_127130 otu_22646 otu_150214 otu_139239 otu_106346 otu_81569
otu_5686 otu_2886 otu_107536 otu_4218 otu_6128 otu_27850 otu_96722 otu_105593
otu_110031 otu_4405 otu_79127 otu_37003 otu_28025 otu_139545 otu_103170
otu_152736 otu_163260 otu_108557 otu_24723 otu_30367 otu_32394 otu_6455
otu_80832 otu_151074 otu_23089 otu_127374 otu_29964 otu_80721 otu_139167
otu_152221 otu_27216 otu_146598 otu_152658 otu_138564 otu_106259 otu_134319
otu_152676 otu_103151 otu_134458 otu_99455 otu_105667 otu_33632 otu_768
otu_152232 otu_32437 otu_22424 otu_127089 otu_23510 otu_99760 otu_23950
otu_151738 otu_2659 otu_144011 otu_105440 otu_79568 otu_26036 otu_151347

otu_29274 otu_4324 otu_151618 otu_28949 otu_151881 otu_82588 otu_163994
otu_150684 otu_152140 otu_99368 otu_152110 otu_107579 otu_82192 otu_133074
otu_106771 otu_127130 otu_22646 otu_150214 otu_139239 otu_106346 otu_81569
otu_5686 otu_2886 otu_107536 otu_4218 otu_6128 otu_27850 otu_96722 otu_105593
otu_110031 otu_4405 otu_79127 otu_37003 otu_28025 otu_139545 otu_103170
otu_152736 otu_163260 otu_108557 otu_24723 otu_30367 otu_32394 otu_6455
otu_80832 otu_151074 otu_23089 otu_127374 otu_29964 otu_80721 otu_139167
otu_152221 otu_27216 otu_146598 otu_152658 otu_138564 otu_106259 otu_134319
otu_152676 otu_103151 otu_134458 otu_99455 otu_105667 otu_33632 otu_768
otu_152232 otu_32437 otu_22424 otu_127089 otu_23510 otu_99760 otu_23950
otu_151738 otu_2659 otu_144011 otu_105440 otu_79568 otu_26036 otu_151347
otu_146411 otu_28610 otu_34926 otu_24382 otu_84267 otu_2407 otu_29306 otu_3731
otu_139728 otu_133046 otu_3262 otu_105899 otu_146905 otu_106115 otu_83087
otu_82025 otu_139628 otu_31445 otu_2151 otu_153301 otu_28550 otu_27356
otu_109068 otu_80649 otu_136441 otu_138440 otu_7341 otu_29036 otu_127926
otu_27920 otu_82806 otu_106920 otu_80480 otu_34896 otu_110059 otu_3398 otu_2876
otu_142145 otu_30500 otu_25899 otu_96982 otu_79375 otu_79467 otu_80253
otu_105476 otu_81651 otu_2352 otu_109606 otu_25539 otu_141804 otu_106382
otu_79578 otu_7128 otu_4443 otu_6891 otu_3341 otu_150439 otu_107907 otu_25824
otu_99985 otu_34778 otu_154130 otu_24048 otu_137257 otu_146293 otu_109149
otu_22969 otu_106097 otu_126281 otu_32494 otu_79967 otu_24415 otu_103040
otu_127347 otu_80935 otu_150203 otu_4572 otu_150636 otu_146552 otu_34469
otu_150576 otu_109069 otu_79342 otu_25184 otu_150616 otu_107021 otu_150880
otu_26960 otu_2161 otu_132756 otu_153609 otu_23967 otu_25771 otu_136722
otu_151981 otu_34946 otu_31575 otu_139577 otu_127378 otu_3293 otu_26356
otu_127362 otu_25687 otu_33719 otu_154026 otu_22741 otu_132935 otu_142453
otu_163829 otu_80774 otu_146972 otu_29413 otu_151708 otu_3169 otu_134721
otu_151899 otu_2307 otu_99971 otu_137646 otu_106778 otu_80132 otu_132618
otu_80530 otu_126629 otu_25154 otu_84007 otu_105864 otu_4720 otu_24280 otu_78948
otu_31326 otu_150207 otu_107415 otu_137081 otu_127936 otu_108097 otu_29343
otu_154139 otu_24883 otu_35014 otu_106286 otu_82323 otu_96888 otu_23828
otu_28062 otu_7203 otu_151411 otu_105529 otu_132635 otu_83159 otu_105853
otu_80194 otu_35298 otu_126274 otu_150192 otu_110249 otu_103148 otu_153254
otu_23147 otu_142698 otu_6706 otu_142010 otu_24183 otu_143819 otu_139795
otu_4182 otu_154328 otu_3557 otu_4261 otu_31254 otu_33943 otu_146849 otu_96913
otu_132818 otu_29655 otu_81577 otu_83017 otu_29704 otu_108658 otu_139235
otu_102961 otu_5389 otu_29562 otu_22309 otu_150481 otu_106393 otu_4063 otu_79327
otu_6670 otu_638 otu_22318 otu_2813 otu_150224 otu_22493 otu_106249 otu_32655
otu_137814 otu_80942 otu_152920 otu_142029 otu_84134 otu_26480 otu_82096
otu_79584 otu_140746 otu_150511 otu_395 otu_3999 otu_23559 otu_25611 otu_79359
otu_83354 otu_81197 otu_143933 otu_163345 otu_28889 otu_667 otu_24002 otu_132597
otu_3801 otu_151158 otu_103574 otu_24204 otu_139851 otu_96977 otu_28528
otu_106802 otu_2403 otu_107394 otu_141110 otu_150124 otu_108863 otu_132660
otu_142669 otu_152215 otu_140997 otu_138453 otu_96693 otu_80170 otu_23254
otu_35175 otu_35045 otu_83062 otu_143835 otu_3746 otu_136466 otu_126677
otu_34117 otu_106984 otu_146422 otu_139533 otu_7002 otu_97093 otu_29957
otu_127212 otu_34734 otu_31006 otu_81555 otu_132592 otu_163990 otu_34688
otu_22763 otu_83395 otu_5074 otu_79848 otu_33586 otu_109835 otu_26121 otu_163279
otu_80898 otu_150733 otu_393 otu_151598 otu_140802 otu_80414 otu_3569 otu_152582
otu_79862 otu_23687 otu_4950 otu_102979 otu_109305 otu_31075 otu_146642
otu_35581 otu_150870 otu_100023 otu_6483 otu_144372 otu_127670 otu_36554
otu_105402 otu_3086 otu_144191 otu_31974 otu_109528 otu_151577 otu_5706
otu_79432 otu_83109 otu_140724 otu_26488 otu_35576 otu_83545 otu_28001 otu_32434
otu_82949 otu_154290 otu_28179 otu_32587 otu_105973 otu_27282 otu_30861
otu_80428 otu_152401 otu_81784 otu_153357 otu_5953 otu_152437 otu_107940
otu_152371 otu_79505 otu_83339 otu_22658 otu_4308 otu_23879 otu_2539 otu_151018
otu_105530 otu_132960 otu_150731 otu_27231 otu_6769 otu_32359 otu_109497
otu_31456 otu_144122 otu_3604 otu_22586 otu_109145 otu_25492 otu_79832
otu_150201 otu_105535 otu_109425 otu_22503 otu_26823 otu_142278 otu_5923
otu_4822 otu_132626 otu_35541 otu_144174 otu_559 otu_33339 otu_4096 otu_126233
otu_25346 otu_80451 otu_152385 otu_106961 otu_27027 otu_144407 otu_80337
otu_132921 otu_150614 otu_153594 otu_108069 otu_6570 otu_773 otu_28920 otu_2577
otu_150165 otu_24926 otu_27062 otu_36193 otu_106842 otu_4471 otu_25755
otu_153744 otu_126576 otu_22330 otu_127764 otu_34638 otu_96712 otu_24801
otu_81982 otu_5873 otu_152242 otu_106353 otu_154244 otu_4022 otu_28861 otu_28360
otu_31126 otu_163762 otu_154289 otu_109702 otu_82217 otu_2271 otu_139762
otu_151197 otu_146652 otu_25232 otu_150195 otu_151730 otu_30419 otu_25452
otu_29600 otu_154222 otu_100151 otu_23797 otu_2723 otu_134550 otu_32890
otu_146216 otu_22490 otu_108225 otu_151001 otu_96788 otu_153579 otu_27417
otu_2589 otu_34541 otu_7351 otu_127749 otu_150999 otu_6329 otu_150841 otu_140714
otu_36782 otu_132873 otu_146543 otu_25699 otu_30380 otu_78963 otu_134701
otu_84129 otu_82009 otu_24190 otu_5133 otu_28992 otu_133031 otu_36212 otu_2793
otu_80615 otu_99492 otu_2860 otu_3799 otu_103273 otu_126541 otu_7275 otu_107508
otu_36903 otu_138559 otu_152681 otu_5250 otu_4362 otu_22794 otu_4957 otu_28296
otu_151757 otu_107108 otu_31039 otu_81612 otu_80062 otu_32179 otu_140729
otu_150404 otu_27328 otu_144231 otu_105811 otu_26259 otu_107921 otu_153827
otu_79187 otu_151421 otu_142213 otu_82961 otu_3260 otu_108613 otu_146576
otu_28238 otu_140780 otu_2829 otu_4137 otu_4878 otu_79225 otu_3079 otu_27840
otu_140834 otu_24979 otu_79943 otu_99670 otu_30683 otu_105618 otu_81808
otu_23112 otu_105915 otu_29513 otu_106547 otu_127076 otu_30515 otu_24254
otu_29599 otu_31100 otu_3639 otu_109637 otu_126184 otu_30202 otu_107137 otu_2568
otu_79177 otu_106315 otu_83115 otu_152892 otu_150229 otu_127737 otu_146799
otu_96760 otu_109484 otu_23387 otu_139662 otu_25010 otu_29215 otu_107667
otu_154291 otu_78973 otu_140713 otu_34780 otu_126562 otu_141782 otu_139911
otu_142362 otu_142560 otu_109125 otu_5121 otu_23081 otu_103217 otu_103566
otu_79885 otu_154354 otu_80789 otu_32848 otu_81584 otu_22974 otu_105388 otu_4454
otu_27851 otu_146219 otu_33058 otu_107275 otu_79456 otu_79728 otu_23826
otu_28915 otu_4185 otu_3272 otu_138574 otu_107072 otu_132900 otu_22589
otu_140764 otu_82946 otu_79569 otu_110509 otu_106188 otu_100057 otu_22945
otu_29258 otu_126164 otu_2149 otu_142532 otu_81272 otu_25758 otu_6420 otu_22732
otu_146613 otu_82038 otu_142579 otu_108749 otu_151034 otu_150164 otu_140
otu_23166 otu_78991 otu_25153 otu_107829 otu_99545 otu_140977 otu_4074
otu_151903 otu_79621 otu_32151 otu_144226 otu_26616 otu_141851 otu_2686
otu_150675 otu_23571 otu_126939 otu_32787 otu_79437 otu_105500 otu_79726
otu_79264 otu_105785 otu_27861 otu_108842 otu_81540 otu_83181 otu_107446
otu_23117 otu_146531 otu_79793 otu_28373 otu_32571 otu_24225 otu_142307
otu_151191 otu_26866 otu_150875 otu_80648 otu_3495 otu_80381 otu_26580 otu_80145
otu_146421 otu_35002 otu_126312 otu_79487 otu_28546 otu_3985 otu_82104
otu_103035 otu_31804 otu_32041 otu_22968 otu_24657 otu_106584 otu_2416 otu_32529
otu_32493 otu_153427 otu_151005 otu_29558 otu_24334 otu_103401 otu_4966
otu_27980 otu_34425 otu_154170 otu_153153 otu_99717 otu_140678 otu_24193
otu_26176 otu_143816 otu_103055 otu_33275 otu_29291 otu_99229 otu_152345
otu_79788 otu_27773 otu_105779 otu_28764 otu_146375 otu_32506 otu_22712
otu_26061 otu_30662 otu_147243 otu_108401 otu_22883 otu_3919 otu_151179
otu_108212 otu_81650 otu_146814 otu_30126 otu_126372 otu_31057 otu_138468
otu_83445 otu_132596 otu_163234 otu_4482 otu_24575 otu_105924 otu_147479
otu_79890 otu_106730 otu_99362 otu_5045 otu_36866 otu_97198 otu_5346 otu_31951
otu_147027 otu_79865 otu_80660 otu_31182 otu_3812 otu_2826 otu_81335 otu_464
otu_132494 otu_27239 otu_132768 otu_153815 otu_109429 otu_108290 otu_146730

otu_146421 otu_35002 otu_126312 otu_79487 otu_28546 otu_3985 otu_82104
otu_103035 otu_31804 otu_32041 otu_22968 otu_24657 otu_106584 otu_2416 otu_32529
otu_32493 otu_153427 otu_151005 otu_29558 otu_24334 otu_103401 otu_4966
otu_27980 otu_34425 otu_154170 otu_153153 otu_99717 otu_140678 otu_24193
otu_26176 otu_143816 otu_103055 otu_33275 otu_29291 otu_99229 otu_152345
otu_79788 otu_27773 otu_105779 otu_28764 otu_146375 otu_32506 otu_22712
otu_26061 otu_30662 otu_147243 otu_108401 otu_22883 otu_3919 otu_151179
otu_108212 otu_81650 otu_146814 otu_30126 otu_126372 otu_31057 otu_138468
otu_83445 otu_132596 otu_163234 otu_4482 otu_24575 otu_105924 otu_147479
otu_79890 otu_106730 otu_99362 otu_5045 otu_36866 otu_97198 otu_5346 otu_31951
otu_147027 otu_79865 otu_80660 otu_31182 otu_3812 otu_2826 otu_81335 otu_464
otu_132494 otu_27239 otu_132768 otu_153815 otu_109429 otu_108290 otu_146730
otu_126859 otu_139513 otu_80764 otu_23342 otu_23549 otu_27181 otu_142473
otu_132812 otu_5117 otu_141882 otu_22693 otu_4120 otu_2118 otu_32701 otu_106290
otu_105441 otu_132532 otu_2441 otu_80943 otu_105450 otu_25751 otu_80140
otu_137325 otu_80114 otu_3852 otu_29490 otu_82550 otu_23917 otu_127133 otu_81866
otu_151953 otu_28021 otu_108146 otu_139531 otu_127160 otu_33229 otu_25825
otu_142615 otu_107240 otu_139542 otu_100043 otu_78951 otu_134373 otu_79682
otu_2529 otu_146870 otu_2258 otu_152249 otu_103338 otu_107807 otu_99650 otu_5626
otu_106332 otu_103545 otu_81933 otu_4257 otu_151585 otu_25060 otu_3009
otu_103315 otu_33607 otu_144258 otu_28687 otu_33658 otu_82277 otu_147198
otu_29061 otu_154528 otu_132567 otu_30665 otu_34815 otu_152718 otu_138512
otu_96897 otu_132833 otu_34785 otu_126577 otu_152098 otu_151049 otu_106381
otu_79904 otu_23382 otu_23669 otu_102817 otu_127663 otu_105974 otu_150082
otu_24416 otu_134595 otu_3112 otu_859 otu_23068 otu_80031 otu_151406 otu_29170
otu_127794 otu_139691 otu_80787 otu_29792 otu_81583 otu_150960 otu_26097
otu_151795 otu_146473 otu_22846 otu_5136 otu_24775 otu_30707 otu_4303 otu_23883
otu_2792 otu_24367 otu_25137 otu_150504 otu_127458 otu_79068 otu_27778 otu_3122
otu_128033 otu_139612 otu_34757 otu_106958 otu_2318 otu_79527 otu_134560
otu_24905 otu_755 otu_141125 otu_142102 otu_25905 otu_24626 otu_127044 otu_3620
otu_26507 otu_26476 otu_27080 otu_30538 otu_29795 otu_150679 otu_29781
otu_109194 otu_2648 otu_5902 otu_151114 otu_99255 otu_108636 otu_107915
otu_143791 otu_25578 otu_146555 otu_30868 otu_24541 otu_33558 otu_97077 otu_5824
otu_23980 otu_29879 otu_29582 otu_152378 otu_97208 otu_105812 otu_27878
otu_27249 otu_28706 otu_25186 otu_81975 otu_81504 otu_107520 otu_106652
otu_103139 otu_132486 otu_24371 otu_22545 otu_4367 otu_25164 otu_24529
otu_150326 otu_150601 otu_81704 otu_5318 otu_126156 otu_150081 otu_31095
otu_152613 otu_126535 otu_132467 otu_34005 otu_102891 otu_79519 otu_4890
otu_25278 otu_99770 otu_81188 otu_22973 otu_154317 otu_3023 otu_106548
otu_107938 otu_110005 otu_107522 otu_103229 otu_140758 otu_151103 otu_140976
otu_105746 otu_126685 otu_163953 otu_23247 otu_24806 otu_151777 otu_163262
otu_29578 otu_5267 otu_24049 otu_5800 otu_151009 otu_32037 otu_5593 otu_141857
otu_97035 otu_107017 otu_2229 otu_152835 otu_106896 otu_127295 otu_26354
otu_107732 otu_81476 otu_4475 otu_137273 otu_108633 otu_152069 otu_79421
otu_146748 otu_153771 otu_22668 otu_80895 otu_37151 otu_108247 otu_5283 otu_5814
otu_139685 otu_36742 otu_4263 otu_79470 otu_3944 otu_32709 otu_80519 otu_103548
otu_5857 otu_126344 otu_30489 otu_35070 otu_31125 otu_27109 otu_26943 otu_28461
otu_5662 otu_32088 otu_152550 otu_28048 otu_79680 otu_153842 otu_536 otu_146217
otu_2861 otu_105939 otu_24331 otu_83060 otu_151231 otu_152888 otu_96729
otu_151935 otu_105995 otu_106388 otu_106643 otu_28118 otu_103321 otu_80213
otu_5460 otu_150513 otu_6184 otu_151911 otu_35102 otu_26129 otu_83947 otu_23616
otu_24106 otu_35011 otu_107181 otu_153118 otu_99347 otu_22316 otu_3349 otu_132786
otu_81411 otu_81590 otu_28225 otu_99832 otu_142031 otu_25900 otu_109492
otu_25891 otu_23538 otu_102998 otu_107432 otu_150099 otu_80948 otu_107188
otu_5362 otu_5875 otu_103190 otu_26907 otu_96848 otu_2207 otu_3454 otu_24401
otu_3268 otu_817 otu_4963 otu_81202 otu_30858 otu_22895 otu_81895 otu_80004
otu_106161 otu_30728 otu_109262 otu_3368 otu_132579 otu_126471 otu_79398
otu_22447 otu_79274 otu_146587 otu_146753 otu_96895 otu_24510 otu_152374
otu_107531 otu_139224 otu_23949 otu_24139 otu_106538 otu_80467 otu_23560
otu_163227 otu_137836 otu_24029 otu_146418 otu_24011 otu_22504 otu_32907
otu_105819 otu_23467 otu_758 otu_28273 otu_142504 otu_151063 otu_3715 otu_109571
otu_137252 otu_408 otu_127984 otu_144003 otu_760 otu_24988 otu_34966 otu_3279
otu_23273 otu_25402 otu_103058 otu_106848 otu_151719 otu_2770 otu_23212
otu_26166 otu_79795 otu_103391 otu_80362 otu_30509 otu_140829 otu_23176
otu_152662 otu_151108 otu_102994 otu_146541 otu_24846 otu_151433 otu_134545
otu_79839 otu_106274 otu_27788 otu_3032 otu_5428 otu_99607 otu_105653 otu_2315
otu_31525 otu_154425 otu_96855 otu_147069 otu_108500 otu_151521 otu_152104
otu_24565 otu_146380 otu_141874 otu_106008 otu_106029 otu_26593 otu_146705
otu_105961 otu_150903 otu_80651 otu_152265 otu_152085 otu_151270 otu_35302
otu_107910 otu_151339 otu_79610 otu_6477 otu_103330 otu_108976 otu_105625
otu_24322 otu_144156 otu_2402 otu_26999 otu_137843 otu_81169 otu_126177 otu_4258
otu_151139 otu_81279 otu_108114 otu_27331 otu_25046 otu_7268 otu_138466 otu_34645
otu_30615 otu_103575 otu_24625 otu_127328 otu_103156 otu_82173 otu_27513
otu_26837 otu_126397 otu_25307 otu_164278 otu_140730 otu_27474 otu_142133
otu_34905 otu_25414 otu_142531 otu_80415 otu_26457 otu_107640 otu_134687
otu_109939 otu_35112 otu_150090 otu_81107 otu_151045 otu_102991 otu_25787
otu_106293 otu_126658 otu_132463 otu_106891 otu_2374 otu_32468 otu_146349
otu_79464 otu_26872 otu_2114 otu_136971 otu_29146 otu_4205 otu_24559 otu_4123
otu_107566 otu_26058 otu_79873 otu_2218 otu_147440 otu_106505 otu_2934
otu_151285 otu_142545 otu_24860 otu_3048 otu_102928 otu_109615 otu_79697
otu_22880 otu_103001 otu_103349 otu_146367 otu_105765 otu_31899 otu_30641
otu_30052 otu_36032 otu_146592 otu_146550 otu_140867 otu_132453 otu_5177
otu_142147 otu_138443 otu_3158 otu_106839 otu_81432 otu_3396 otu_79373
otu_142419 otu_150710 otu_139854 otu_79284 otu_32732 otu_6522 otu_29244 otu_4273
otu_152404 otu_102887 otu_150840 otu_126438 otu_151691 otu_24721 otu_29684
otu_3543 otu_32288 otu_83322 otu_99867 otu_29065 otu_151701 otu_146314 otu_25936
otu_138144 otu_80682 otu_22565 otu_34716 otu_81395 otu_127053 otu_140734
otu_106718 otu_127142 otu_22326 otu_83000 otu_80612 otu_127444 otu_30964
otu_80936 otu_24085 otu_143997 otu_24553 otu_109488 otu_152709 otu_6723
otu_27265 otu_4970 otu_27663 otu_23108 otu_79368 otu_79748 otu_83392 otu_126588
otu_4456 otu_30599 otu_106066 otu_3176 otu_108555 otu_22430 otu_5697 otu_3610
otu_26841 otu_126885 otu_30986 otu_134346 otu_126498 otu_132792 otu_4435
otu_83760 otu_103129 otu_3826 otu_151495 otu_635 otu_23619 otu_26493 otu_716
otu_30795 otu_141136 otu_108352 otu_105955 otu_34657 otu_106120 otu_151448
otu_3996 otu_6163 otu_105983 otu_106994 otu_22331 otu_137324 otu_150369 otu_129
otu_25721 otu_107874 otu_24921 otu_81907 otu_81100 otu_140996 otu_27329
otu_126606 otu_26016 otu_28680 otu_137084 otu_2531 otu_138558 otu_141898
otu_139660 otu_132710 otu_30604 otu_82551 otu_33645 otu_109187 otu_132655
otu_5380 otu_147458 otu_80699 otu_107972 otu_107144 otu_4411 otu_32172
otu_140967 otu_24844 otu_80340 otu_146639 otu_99501 otu_4559 otu_82195 otu_4229
otu_132 otu_26215 otu_24441 otu_27026 otu_5736 otu_3976 otu_126522 otu_140718
otu_23009 otu_3451 otu_105579 otu_150828 otu_105470 otu_138524 otu_2488
otu_110002 otu_29178 otu_150159 otu_80807 otu_106706 otu_134389 otu_97105
otu_4200 otu_29220 otu_109586 otu_144132 otu_142081 otu_96831 otu_79409
otu_134623 otu_106773 otu_28787 otu_79353 otu_27704 otu_26931 otu_143912
otu_30077 otu_81318 otu_139148 otu_5241 otu_126302 otu_5615 otu_22863 otu_99540
otu_105808 otu_140898 otu_28763 otu_24363 otu_97059 otu_153169 otu_2107
otu_80485 otu_81879 otu_152056 otu_620 otu_99673 otu_139602 otu_2884 otu_32215
otu_4681 otu_152082 otu_25850 otu_146791 otu_152024 otu_103284 otu_142276

[illegible]
otu_5380 otu_147458 otu_80699 otu_107972 otu_107144 otu_4411 otu_32172
otu_140967 otu_24844 otu_80340 otu_146639 otu_99501 otu_4559 otu_82195 otu_4229
otu_132 otu_26215 otu_24441 otu_27026 otu_5736 otu_3976 otu_126522 otu_140718
otu_23009 otu_3451 otu_105579 otu_150828 otu_105470 otu_138524 otu_2488
otu_110002 otu_29178 otu_150159 otu_80807 otu_106706 otu_134389 otu_97105
otu_4200 otu_29220 otu_109586 otu_144132 otu_142081 otu_96831 otu_79409
otu_134623 otu_106773 otu_28787 otu_79353 otu_27704 otu_26931 otu_143912
otu_30077 otu_81318 otu_139148 otu_5241 otu_126302 otu_5615 otu_22863 otu_99540
otu_105808 otu_140898 otu_28763 otu_24363 otu_97059 otu_153169 otu_2107
otu_80485 otu_81879 otu_152056 otu_620 otu_99673 otu_139602 otu_2884 otu_32215
otu_4681 otu_152082 otu_25850 otu_146791 otu_152024 otu_103284 otu_142276
otu_23680 otu_147476 otu_2175 otu_26430 otu_150642 otu_109736 otu_102985
otu_140919 otu_29141 otu_23462 otu_102866 otu_3902 otu_140851 otu_106526
otu_4118 otu_144184 otu_103409 otu_35230 otu_31260 otu_106623 otu_144076
otu_4945 otu_79572 otu_151902 otu_150607 otu_23477 otu_151272 otu_133015
otu_5998 otu_36939 otu_151097 otu_153745 otu_138140 otu_79772 otu_6553 otu_29425
otu_83828 otu_80547 otu_6127 otu_151305 otu_6262 otu_103076 otu_151183
otu_102982 otu_24028 otu_23043 otu_132760 otu_153335 otu_4917 otu_23816 otu_3752
otu_103214 otu_31520 otu_143939 otu_25095 otu_81299 otu_30182 otu_31426
otu_102955 otu_96820 otu_4027 otu_28088 otu_105447 otu_152007 otu_22723
otu_80608 otu_27334 otu_105572 otu_24439 otu_83422 otu_96971 otu_36687 otu_30204
otu_24997 otu_108973 otu_26743 otu_26969 otu_151548 otu_862 otu_81332 otu_108207
otu_132827 otu_26663 otu_23451 otu_25126 otu_25505 otu_137069 otu_153015
otu_24173 otu_102886 otu_143963 otu_3012 otu_27162 otu_3095 otu_2990 otu_79573
otu_2435 otu_80802 otu_4013 otu_26576 otu_6880 otu_35753 otu_152193 otu_24476
otu_102911 otu_143931 otu_23799 otu_150470 otu_2721 otu_152199 otu_142431
otu_6457 otu_132445 otu_107745 otu_126665 otu_23189 otu_22679 otu_6375 otu_22616
otu_26856 otu_32156 otu_99394 otu_132545 otu_7017 otu_150110 otu_4828 otu_26345
otu_110100 otu_34543 otu_25800 otu_105571 otu_34766 otu_83591 otu_107354
otu_140816 otu_152456 otu_2680 otu_126527 otu_25812 otu_109281 otu_152491
otu_139861 otu_126460 otu_126407 otu_35726 otu_26556 otu_81139 otu_23042
otu_30586 otu_29583 otu_109476 otu_3581 otu_79851 otu_150319 otu_138409
otu_26722 otu_150747 otu_137097 otu_35106 otu_106252 otu_107673 otu_106409
otu_4141 otu_105395 otu_35538 otu_103351 otu_24604 otu_139588 otu_3324 otu_25591
otu_127094 otu_152532 otu_26089 otu_107187 otu_106903 otu_103118 otu_28847
otu_23678 otu_140928 otu_4045 otu_146329 otu_127172 otu_105614 otu_140952
otu_23810 otu_154882 otu_3354 otu_109098 otu_29761 otu_27415 otu_138451
otu_106334 otu_23660 otu_28486 otu_24015 otu_22987 otu_26885 otu_109164
otu_28768 otu_105867 otu_33540 otu_108739 otu_29238 otu_25856 otu_144374
otu_106552 otu_643 otu_34351 otu_96978 otu_146390 otu_24455 otu_24136 otu_2619
otu_126515 otu_151658 otu_80141 otu_136086 otu_30213 otu_27821 otu_109937
otu_79522 otu_139786 otu_152306 otu_80759 otu_25519 otu_127134 otu_151441
otu_29025 otu_142186 otu_26777 otu_5946 otu_3455 otu_107530 otu_109359
otu_139633 otu_79114 otu_151174 otu_4968 otu_140689 otu_25503 otu_2845 otu_30137
otu_27082 otu_30705 otu_30569 otu_25028 otu_153001 otu_108190 otu_141888
otu_103248 otu_34171 otu_127664 otu_107665 otu_22575 otu_3144 otu_22468
otu_152672 otu_29217 otu_154833 otu_79562 otu_4104 otu_107034 otu_141984
otu_26099 otu_26276 otu_26799 otu_81962 otu_84276 otu_3734 otu_142679 otu_4664
otu_5386 otu_139591 otu_3724 otu_108634 otu_81603 otu_5303 otu_26527 otu_105931
otu_80643 otu_143977 otu_150700 otu_106556 otu_24963 otu_35746 otu_24610
otu_26913 otu_24924 otu_80880 otu_80778 otu_140855 otu_79638 otu_4815 otu_136436
otu_29695 otu_80373 otu_81148 otu_143830 otu_6649 otu_4513 otu_106660 otu_27441
otu_151886 otu_107876 otu_24083 otu_146704 otu_146373 otu_106374 otu_23440
otu_25920 otu_30571 otu_132882 otu_106924 otu_27154 otu_136731 otu_28376
otu_163320 otu_132985 otu_662 otu_391 otu_83477 otu_127061 otu_150632 otu_109764
otu_100029 otu_24238 otu_138175 otu_5046 otu_81112 otu_22751 otu_99558 otu_26627
otu_83541 otu_127100 otu_26736 otu_139746 otu_150241 otu_22622 otu_82832
otu_142252 otu_126342 otu_28448 otu_24030 otu_153294 otu_466 otu_27976 otu_84063
otu_80604 otu_150220 otu_79669 otu_152573 otu_27106 otu_106691 otu_154259
otu_28973 otu_23721 otu_24714 otu_107023 otu_84242 otu_34232 otu_7399 otu_134446
otu_142168 otu_81466 otu_146564 otu_24400 otu_105821 otu_32527 otu_109020
otu_143792 otu_144238 otu_151752 otu_4232 otu_33903 otu_728 otu_22432 otu_3157
otu_126698 otu_26513 otu_29131 otu_3964 otu_126701 otu_29669 otu_82012
otu_147332 otu_142176 otu_79085 otu_30027 otu_24760 otu_163272 otu_143907
otu_3767 otu_150811 otu_136146 otu_127296 otu_26581 otu_2504 otu_22718 otu_4644
otu_27631 otu_126452 otu_79287 otu_82907 otu_146767 otu_27138 otu_151643
otu_153258 otu_26243 otu_25422 otu_3339 otu_147084 otu_79691 otu_97130 otu_99308
otu_139172 otu_31091 otu_141808 otu_152911 otu_29915 otu_6114 otu_26653
otu_152595 otu_107130 otu_142555 otu_126838 otu_24303 otu_150992 otu_23220
otu_34413 otu_106462 otu_152412 otu_146829 otu_2550 otu_99375 otu_26423
otu_141892 otu_139912 otu_3694 otu_5161 otu_34717 otu_3321 otu_140875 otu_106768
otu_27972 otu_139546 otu_127470 otu_151278 otu_2642 otu_142576 otu_27795
otu_30807 otu_29935 otu_82503 otu_127369 otu_139511 otu_80740 otu_139670
otu_81294 otu_24309 otu_32047 otu_26452 otu_150213 otu_81303 otu_30987 otu_22444
otu_79778 otu_24061 otu_143936 otu_29405 otu_81232 otu_2678 otu_134536 otu_79557
otu_80555 otu_5969 otu_25458 otu_29586 otu_106495 otu_36649 otu_134323
otu_134636 otu_26302 otu_79837 otu_150478 otu_151748 otu_126784 otu_150115
otu_32808 otu_137061 otu_25144 otu_151759 otu_83365 otu_132605 otu_133059
otu_790 otu_107631 otu_34052 otu_31982 otu_27550 otu_532 otu_33024 otu_146892
otu_151832 otu_96835 otu_107610 otu_147091 otu_147144 otu_3788 otu_82798
otu_144046 otu_142080 otu_31898 otu_139647 otu_108197 otu_34176 otu_151146
otu_82215 otu_139775 otu_3650 otu_80369 otu_106194 otu_153563 otu_80040
otu_153018 otu_32904 otu_35236 otu_105456 otu_27303 otu_79073 otu_152549
otu_106422 otu_27085 otu_144338 otu_22523 otu_136746 otu_150803 otu_139614
otu_136984 otu_23993 otu_106412 otu_79803 otu_33830 otu_25035 otu_105985
otu_107368 otu_110154 otu_139572 otu_25081 otu_3640 otu_2703 otu_81270 otu_3956
otu_23951 otu_152533 otu_24884 otu_5838 otu_105616 otu_27220 otu_33424 otu_5288
otu_137260 otu_102888 otu_23521 otu_105692 otu_103225 otu_102841 otu_108952
otu_23511 otu_23393 otu_127266 otu_143847 otu_28933 otu_37100 otu_106443
otu_27841 otu_105948 otu_35732 otu_79917 otu_106369 otu_106811 otu_147067
otu_28783 otu_434 otu_24396 otu_107094 otu_105468 otu_83944 otu_84223 otu_29718
otu_80749 otu_132694 otu_153610 otu_80199 otu_2997 otu_79843 otu_30889 otu_30148
otu_151685 otu_153368 otu_80696 otu_3067 otu_110290 otu_24072 otu_622 otu_31847
otu_3660 otu_542 otu_141934 otu_23618 otu_78990 otu_143807 otu_150637 otu_4516
otu_22776 otu_150585 otu_22407 otu_106529 otu_140766 otu_849 otu_6978 otu_39
otu_79636 otu_6705 otu_26563 otu_136111 otu_34635 otu_152733 otu_24832 otu_109060
otu_150854 otu_105643 otu_26064 otu_25446 otu_150188 otu_35587 otu_107569
otu_25088 otu_107638 otu_81908 otu_30306 otu_4816 otu_24799 otu_34018 otu_139592
otu_23157 otu_28405 otu_81108 otu_27683 otu_403 otu_79583 otu_32785 otu_2400
otu_142109 otu_132512 otu_28407 otu_22976 otu_28637 otu_24095 otu_30145
otu_79336 otu_134327 otu_29549 otu_108370 otu_126933 otu_25709 otu_142506
otu_80163 otu_3306 otu_22904 otu_27958 otu_110323 otu_108504 otu_24768 otu_3501
otu_140756 otu_26026 otu_127066 otu_144301 otu_126477 otu_150263 otu_4201
otu_143818 otu_106328 otu_99715 otu_23263 otu_2224 otu_25878 otu_4501 otu_25203
otu_567 otu_32207 otu_100021 otu_106895 otu_146837 otu_152611 otu_25945
otu_127642 otu_6131 otu_31447 otu_34886 otu_107022 otu_109202 otu_106038
otu_28496 otu_151426 otu_152625 otu_108036 otu_24103 otu_105526 otu_24289
otu_139702 otu_151267 otu_106872 otu_4127 otu_29779 otu_126492 otu_81871
[illegible]

otu_25088 otu_107638 otu_81908 otu_30306 otu_4816 otu_24799 otu_34018 otu_139592
otu_23157 otu_28405 otu_81108 otu_27683 otu_403 otu_79583 otu_32785 otu_2400
otu_142109 otu_132512 otu_28407 otu_22976 otu_28637 otu_24095 otu_30145
otu_79336 otu_134327 otu_29549 otu_108370 otu_126933 otu_25709 otu_142506
otu_80163 otu_3306 otu_22904 otu_27958 otu_110323 otu_108504 otu_24768 otu_3501
otu_140756 otu_26026 otu_127066 otu_144301 otu_126477 otu_150263 otu_4201
otu_143818 otu_106328 otu_99715 otu_23263 otu_2224 otu_25878 otu_4501 otu_25203
otu_567 otu_32207 otu_100021 otu_106895 otu_146837 otu_152611 otu_25945
otu_127642 otu_6131 otu_31447 otu_34886 otu_107022 otu_109202 otu_106038
otu_28496 otu_151426 otu_152625 otu_108036 otu_24103 otu_105526 otu_24289
otu_139702 otu_151267 otu_106872 otu_4127 otu_29779 otu_126492 otu_81871
otu_79511 otu_134426 otu_31546 otu_3544 otu_3464 otu_23100 otu_27159 otu_79895
otu_107214 otu_3678 otu_105820 otu_29040 otu_81617 otu_152091 otu_134742
otu_80862 otu_153753 otu_132687 otu_33471 otu_142520 otu_25978 otu_150331
otu_24033 otu_99432 otu_5354 otu_36527 otu_150097 otu_109883 otu_147220
otu_151753 otu_34343 otu_23460 otu_25660 otu_29943 otu_150338 otu_80156
otu_163327 otu_7223 otu_22388 otu_127077 otu_138174 otu_106759 otu_30802
otu_81929 otu_79538 otu_152144 otu_33311 otu_24398 otu_36098 otu_83201 otu_82822
otu_5836 otu_79944 otu_79062 otu_36465 otu_134552 otu_7326 otu_4461 otu_140751
otu_107493 otu_4597 otu_81357 otu_141786 otu_29479 otu_152372 otu_127724
otu_142540 otu_4043 otu_4249 otu_109959 otu_138150 otu_126354 otu_79074
otu_146826 otu_24418 otu_23210 otu_26654 otu_151281 otu_22416 otu_126263
otu_27764 otu_25093 otu_82454 otu_132533 otu_150200 otu_79279 otu_140743
otu_106135 otu_137317 otu_136143 otu_2232 otu_152824 otu_25048 otu_406 otu_97220
otu_624 otu_81428 otu_79395 otu_139694 otu_448 otu_147149 otu_23082 otu_150666
otu_141947 otu_35527 otu_6438 otu_4651 otu_34075 otu_25436 otu_81930 otu_31890
otu_32466 otu_34647 otu_24207 otu_132629 otu_34412 otu_666 otu_29181 otu_147023
otu_151811 otu_153098 otu_151940 otu_3234 otu_28361 otu_80808 otu_23191
otu_27926 otu_105895 otu_132468 otu_150210 otu_30423 otu_150889 otu_2528
otu_5199 otu_140911 otu_107407 otu_110425 otu_25434 otu_5385 otu_24613 otu_80576
otu_22698 otu_28715 otu_83128 otu_26323 otu_109146 otu_633 otu_127359 otu_4196
otu_35450 otu_150454 otu_154030 otu_81815 otu_80962 otu_150092 otu_24097
otu_106686 otu_23944 otu_22515 otu_163264 otu_153329 otu_22949 otu_29144
otu_137305 otu_5395 otu_103303 otu_35575 otu_79105 otu_108386 otu_127292
otu_96822 otu_106083 otu_140697 otu_108565 otu_34907 otu_139230 otu_80708
otu_153275 otu_24971 otu_4382 otu_26905 otu_108209 otu_152601 otu_102822 otu_845
otu_35607 otu_24554 otu_107284 otu_3406 otu_30270 otu_100020 otu_7160 otu_29357
otu_107597 otu_106654 otu_150728 otu_81360 otu_142523 otu_2264 otu_79518
otu_83685 otu_6087 otu_143981 otu_132566 otu_7273 otu_3514 otu_82342 otu_80433
otu_25631 otu_33138 otu_150228 otu_164056 otu_105758 otu_153634 otu_108735
otu_99975 otu_146337 otu_126202 otu_23413 otu_103008 otu_150367 otu_141872
otu_127355 otu_79747 otu_24227 otu_26053 otu_127067 otu_154688 otu_127492
otu_22997 otu_146615 otu_24100 otu_141826 otu_28596 otu_29246 otu_4453 otu_22912
otu_79501 otu_7241 otu_33099 otu_81870 otu_147133 otu_108120 otu_126836
otu_106723 otu_103033 otu_150435 otu_31142 otu_146655 otu_23423 otu_79784
otu_3345 otu_32740 otu_141925 otu_106574 otu_134775 otu_30240 otu_23241
otu_28714 otu_30763 otu_4770 otu_26759 otu_30670 otu_107146 otu_28075 otu_82439
otu_25059 otu_33715 otu_79844 otu_28737 otu_151760 otu_33990 otu_139763
otu_28329 otu_24522 otu_25832 otu_82629 otu_32854 otu_25794 otu_4412 otu_105559
otu_138530 otu_142 otu_5233 otu_33019 otu_139179 otu_26941 otu_28052 otu_4140
otu_103265 otu_146605 otu_151589 otu_31519 otu_126338 otu_132888 otu_108366
otu_880 otu_80874 otu_29708 otu_80853 otu_99243 otu_107509 otu_107210 otu_2145
otu_128045 otu_139186 otu_35072 otu_105884 otu_139193 otu_25076 otu_150630
otu_150196 otu_150945 otu_3277 otu_137800 otu_107061 otu_105857 otu_127671
otu_4078 otu_144405 otu_31681 otu_31979 otu_107652 otu_80746 otu_4116 otu_3177
otu_144456 otu_127804 otu_28116 otu_23823 otu_25743 otu_99625 otu_3046 otu_31136
otu_2765 otu_151623 otu_134599 otu_144097 otu_143922 otu_82963 otu_79245
otu_29734 otu_143971 otu_144304 otu_5063 otu_22865 otu_106704 otu_96711
otu_102802 otu_32953 otu_106509 otu_29416 otu_134717 otu_756 otu_99316
otu_126358 otu_23336 otu_106242 otu_132686 otu_26459 otu_29031 otu_150644
otu_28338 otu_146758 otu_22711 otu_3189 otu_107360 otu_146330 otu_107935
otu_2958 otu_22340 otu_78983 otu_137292 otu_105568 otu_23022 otu_33799 otu_22975
otu_4942 otu_142326 otu_103221 otu_23722 otu_142049 otu_150626 otu_23845
otu_25124 otu_4570 otu_154761 otu_79880 otu_27353 otu_36967 otu_126678 otu_23554
otu_152054 otu_103212 otu_151876 otu_2696 otu_26013 otu_105925 otu_152092
otu_33753 otu_32729 otu_25560 otu_36706 otu_25596 otu_32682 otu_25275 otu_126630
otu_22592 otu_80809 otu_24593 otu_106167 otu_27206 otu_127839 otu_106554
otu_82151 otu_5820 otu_108996 otu_25267 otu_147026 otu_33616 otu_141852
otu_26328 otu_132953 otu_79004 otu_106273 otu_31522 otu_31647 otu_5852
otu_150496 otu_25635 otu_146389 otu_2609 otu_140779 otu_140694 otu_80200
otu_26450 otu_80124 otu_126408 otu_152597 otu_80469 otu_3807 otu_108004
otu_146540 otu_26418 otu_79854 otu_34923 otu_140953 otu_23359 otu_4671
otu_152927 otu_106053 otu_26836 otu_151900 otu_126593 otu_150451 otu_32187
otu_105556 otu_25912 otu_106968 otu_29438 otu_28673 otu_29162 otu_81109
otu_106748 otu_22337 otu_139709 otu_146606 otu_105513 otu_151190 otu_619
otu_107817 otu_132783 otu_107233 otu_3884 otu_79466 otu_103577 otu_146565
otu_31669 otu_142283 otu_79341 otu_82222 otu_34731 otu_79162 otu_3986 otu_146343
otu_154094 otu_136076 otu_35588 otu_136952 otu_142627 otu_109524 otu_105966
otu_3754 otu_108763 otu_79543 otu_29649 otu_23595 otu_80318 otu_3988 otu_144284
otu_106002 otu_27872 otu_137315 otu_22721 otu_32296 otu_153028 otu_3894
otu_26348 otu_150933 otu_146513 otu_107295 otu_80144 otu_26498 otu_80884
otu_3269 otu_99503 otu_140711 otu_82274 otu_23914 otu_107335 otu_137886
otu_24373 otu_3651 otu_4563 otu_81718 otu_80435 otu_30286 otu_126695 otu_139843
otu_132713 otu_144120 otu_3180 otu_106641 otu_141992 otu_150908 otu_4047
otu_139582 otu_142101 otu_25405 otu_23024 otu_36055 otu_103482 otu_126159
otu_99454 otu_31297 otu_25393 otu_24598 otu_150328 otu_154048 otu_28510
otu_105492 otu_27556 otu_150707 otu_4271 otu_23705 otu_144176 otu_105740
otu_126566 otu_3494 otu_126316 otu_139884 otu_142165 otu_151972 otu_106787
otu_99517 otu_27317 otu_140924 otu_24755 otu_24397 otu_23488 otu_107474
otu_24551 otu_81214 otu_5682 otu_35071 otu_32939 otu_3348 otu_153157 otu_4329
otu_103491 otu_126742 otu_138188 otu_28383 otu_36681 otu_22916 otu_2647 otu_552
otu_5901 otu_163338 otu_34083 otu_107881 otu_108117 otu_151211 otu_107009
otu_151490 otu_29541 otu_153796 otu_107397 otu_32882 otu_6579 otu_163914
otu_141967 otu_31002 otu_100087 otu_126804 otu_143884 otu_25695 otu_108711
otu_5322 otu_34435 otu_106746 otu_107304 otu_108282 otu_140858 otu_2339
otu_151354 otu_4295 otu_3221 otu_23124 otu_152228 otu_142138 otu_103323
otu_23036 otu_4604 otu_142559 otu_731 otu_126461 otu_27786 otu_28004 otu_150560
otu_32471 otu_24805 otu_126214 otu_6332 otu_80210 otu_153341 otu_30490
otu_151332 otu_146641 otu_36248 otu_6594 otu_6300 otu_126637 otu_5087 otu_151601
otu_108322 otu_152999 otu_35272 otu_99325 otu_107262 otu_28943 otu_80401
otu_102964 otu_106148 otu_2418 otu_146489 otu_147008 otu_150650 otu_102852
otu_109389 otu_137275 otu_34971 otu_190 otu_81887 otu_33153 otu_26709 otu_26890
otu_146602 otu_3560 otu_105409 otu_24830 otu_83377 otu_126420 otu_24996
otu_83047 otu_99601 otu_82640 otu_576 otu_164266 otu_30043 otu_126624 otu_139667
otu_36698 otu_25588 otu_32074 otu_151963 otu_184 otu_29554 otu_31342 otu_23900
otu_24188 otu_35083 otu_23575 otu_150387 otu_3726 otu_108564 otu_24314 otu_31940
otu_36290 otu_108992 otu_3235 otu_126343 otu_134371 otu_140686 otu_144281
otu_31755 otu_2276 otu_109588 otu_34135 otu_25998 otu_22822 otu_138430 otu_23591
otu_139798 otu_22350 otu_35387 otu_107943 otu_4854 otu_25749 otu_139204

[illegible]
otu_151332 otu_146641 otu_36248 otu_6594 otu_6300 otu_126637 otu_5087 otu_151601
otu_108322 otu_152999 otu_35272 otu_99325 otu_107262 otu_28943 otu_80401
otu_102964 otu_106148 otu_2418 otu_146489 otu_147008 otu_150650 otu_102852
otu_109389 otu_137275 otu_34971 otu_190 otu_81887 otu_33153 otu_26709 otu_26890
otu_146602 otu_3560 otu_105409 otu_24830 otu_83377 otu_126420 otu_24996
otu_83047 otu_99601 otu_82640 otu_576 otu_164266 otu_30043 otu_126624 otu_139667
otu_36698 otu_25588 otu_32074 otu_151963 otu_184 otu_29554 otu_31342 otu_23900
otu_24188 otu_35083 otu_23575 otu_150387 otu_3726 otu_108564 otu_24314 otu_31940
otu_36290 otu_108992 otu_3235 otu_126343 otu_134371 otu_140686 otu_144281
otu_31755 otu_2276 otu_109588 otu_34135 otu_25998 otu_22822 otu_138430 otu_23591
otu_139798 otu_22350 otu_35387 otu_107943 otu_4854 otu_25749 otu_139204
otu_152259 otu_4503 otu_80113 otu_81707 otu_23498 otu_82777 otu_102798
otu_105994 otu_102806 otu_6336 otu_31022 otu_81631 otu_81015 otu_27691
otu_151257 otu_108296 otu_5100 otu_22570 otu_102999 otu_99297 otu_4518 otu_36475
otu_82694 otu_2370 otu_29631 otu_142487 otu_30278 otu_106085 otu_2496 otu_36912
otu_150532 otu_27012 otu_28997 otu_79182 otu_132801 otu_29597 otu_108165
otu_154689 otu_79910 otu_143790 otu_79233 otu_2187 otu_82842 otu_28091
otu_109944 otu_136760 otu_5370 otu_476 otu_27300 otu_24688 otu_109475 otu_97132
otu_4226 otu_144166 otu_142150 otu_107885 otu_152158 otu_80081 otu_105910
otu_551 otu_30491 otu_109817 otu_126616 otu_82480 otu_137877 otu_144323
otu_106774 otu_143909 otu_36269 otu_140863 otu_27437 otu_22607 otu_106600
otu_137816 otu_147010 otu_108040 otu_140757 otu_137311 otu_83216 otu_35263
otu_28343 otu_153633 otu_3342 otu_126752 otu_22642 otu_105753 otu_151282
otu_28404 otu_133098 otu_108461 otu_126450 otu_6137 otu_36360 otu_152068
otu_31276 otu_105790 otu_144004 otu_106288 otu_152277 otu_146900 otu_127586
otu_22364 otu_79640 otu_7373 otu_470 otu_24807 otu_80237 otu_3437 otu_80566
otu_107363 otu_80274 otu_3563 otu_107699 otu_153066 otu_126486 otu_107855
otu_99745 otu_163255 otu_152820 otu_153242 otu_152980 otu_32802 otu_79686
otu_27351 otu_107616 otu_143826 otu_25572 otu_142357 otu_83015 otu_2910
otu_137626 otu_26574 otu_23584 otu_81479 otu_150519 otu_5589 otu_139199
otu_30175 otu_100054 otu_23710 otu_83574 otu_127180 otu_106940 otu_154647
otu_81832 otu_105927 otu_2171 otu_132723 otu_23145 otu_108047 otu_24811
otu_134456 otu_110117 otu_108977 otu_105438 otu_152678 otu_3179 otu_36162
otu_4248 otu_133032 otu_100107 otu_4949 otu_29588 otu_80726 otu_759 otu_143844
otu_134476 otu_163263 otu_109879 otu_33988 otu_150119 otu_107621 otu_141962
otu_153690 otu_36668 otu_142140 otu_25744 otu_24973 otu_79633 otu_152185
otu_36952 otu_32664 otu_97001 otu_154403 otu_24758 otu_5099 otu_24646 otu_106132
otu_29862 otu_96661 otu_126472 otu_106358 otu_132994 otu_34051 otu_26850
otu_26152 otu_142249 otu_29651 otu_7052 otu_25523 otu_5876 otu_146706 otu_23512
otu_106954 otu_2208 otu_108332 otu_109316 otu_436 otu_26779 otu_5448 otu_133135
otu_79275 otu_154680 otu_106156 otu_79216 otu_23804 otu_26400 otu_97019
otu_108927 otu_150655 otu_146729 otu_23527 otu_2999 otu_3510 otu_4927 otu_126852
otu_24138 otu_34257 otu_106629 otu_134380 otu_108111 otu_150131 otu_106937
otu_150086 otu_3381 otu_127439 otu_495 otu_27376 otu_27789 otu_103163 otu_36136
otu_4335 otu_137251 otu_32324 otu_137850 otu_670 otu_150434 otu_106245
otu_137295 otu_96794 otu_25327 otu_25829 otu_138439 otu_27826 otu_5162
otu_126255 otu_142184 otu_150508 otu_6653 otu_6969 otu_22310 otu_23946
otu_106367 otu_31046 otu_134535 otu_28250 otu_82131 otu_127423 otu_28854
otu_82891 otu_31578 otu_22594 otu_3327 otu_126290 otu_136471 otu_83234 otu_32671
otu_29951 otu_2338 otu_127629 otu_25790 otu_26629 otu_139585 otu_2783 otu_152129
otu_150985 otu_151298 otu_143940 otu_31158 otu_30320 otu_152520 otu_4436
otu_35026 otu_4974 otu_2769 otu_151252 otu_127319 otu_28392 otu_106646
otu_126621 otu_5605 otu_80875 otu_108012 otu_23572 otu_3375 otu_151004 otu_5644
otu_25103 otu_28661 otu_107328 otu_80515 otu_26529 otu_147250 otu_30337
otu_83248 otu_6841 otu_2182 otu_99684 otu_147271 otu_103122 otu_31258 otu_27806
otu_79338 otu_4107 otu_30304 otu_150580 otu_22329 otu_81093 otu_31121 otu_107475
otu_109657 otu_133047 otu_136064 otu_139596 otu_22784 otu_150363 otu_102901
otu_103287 otu_28137 otu_99995 otu_103460 otu_97147 otu_24440 otu_107583
otu_141915 otu_151648 otu_79762 otu_99708 otu_140947 otu_126468 otu_144421
otu_29259 otu_108348 otu_150394 otu_144051 otu_109141 otu_144303 otu_3491
otu_23420 otu_105991 otu_3760 otu_109752 otu_2308 otu_7122 otu_23855 otu_6074
otu_82168 otu_80343 otu_99307 otu_140752 otu_24084 otu_137083 otu_152348
otu_110541 otu_106681 otu_159 otu_29726 otu_133093 otu_83805 otu_151613
otu_22951 otu_127251 otu_27646 otu_153193 otu_6523 otu_4661 otu_108987 otu_27989
otu_24249 otu_81258 otu_30970 otu_31712 otu_25836 otu_3245 otu_2697 otu_147060
otu_146525 otu_139216 otu_5211 otu_152380 otu_152093 otu_80772 otu_83272
otu_33825 otu_132581 otu_106023 otu_107944 otu_106347 otu_79036 otu_81575
otu_7042 otu_2535 otu_30659 otu_108325 otu_109489 otu_153477 otu_82569 otu_25782
otu_80937 otu_34055 otu_79181 otu_146237 otu_23966 otu_103069 otu_79513
otu_140962 otu_5019 otu_26714 otu_27235 otu_22978 otu_133119 otu_106254
otu_103269 otu_134766 otu_5023 otu_36716 otu_126277 otu_25419 otu_139183
otu_80742 otu_3231 otu_152238 otu_24970 otu_143833 otu_134683 otu_4143 otu_32542
otu_153637 otu_25835 otu_79544 otu_107812 otu_139196 otu_99434 otu_23246
otu_103075 otu_151857 otu_150865 otu_36720 otu_80846 otu_27118 otu_31061
otu_126462 otu_28675 otu_33045 otu_6186 otu_103494 otu_139523 otu_5710
otu_150372 otu_27099 otu_6393 otu_3220 otu_79306 otu_140985 otu_102923
otu_151148 otu_152795 otu_139608 otu_22724 otu_2989 otu_26159 otu_146212
otu_139565 otu_126173 otu_81254 otu_109388 otu_105460 otu_138424 otu_32404
otu_29437 otu_150432 otu_27922 otu_137285 otu_24160 otu_25363 otu_146890
otu_108797 otu_28697 otu_152838 otu_102958 otu_97126 otu_105412 otu_139766
otu_106036 otu_33908 otu_142495 otu_153418 otu_137068 otu_127122 otu_99407
otu_105573 otu_7136 otu_3593 otu_5510 otu_27480 otu_3238 otu_4547 otu_163421
otu_26541 otu_138452 otu_27108 otu_140747 otu_83920 otu_25627 otu_4057 otu_4829
otu_82136 otu_82575 otu_4330 otu_29622 otu_31041 otu_27994 otu_22421 otu_152088
otu_139208 otu_151000 otu_35765 otu_25898 otu_23471 otu_153532 otu_152027
otu_79426 otu_126351 otu_150467 otu_126674 otu_3700 otu_144131 otu_79531
otu_83585 otu_106145 otu_23153 otu_36388 otu_153764 otu_6762 otu_5759 otu_80357
otu_134617 otu_126287 otu_151519 otu_80169 otu_99964 otu_152578 otu_24420
otu_150680 otu_139885 otu_147106 otu_140969 otu_80151 otu_27229 otu_146762
otu_28450 otu_107037 otu_4192 otu_103098 otu_5938 otu_29540 otu_154524 otu_504
otu_79520 otu_146792 otu_141811 otu_23600 otu_163993 otu_144245 otu_147475
otu_26917 otu_32218 otu_150643 otu_22659 otu_3879 otu_32240 otu_32319 otu_142379
otu_134789 otu_4870 otu_707 otu_31267 otu_36862 otu_24293 otu_142248 otu_126204
otu_28760 otu_106551 otu_100165 otu_99789 otu_24081 otu_29366 otu_137055
otu_79732 otu_82977 otu_3085 otu_132922 otu_82314 otu_22931 otu_106745
otu_109477 otu_4105 otu_22859 otu_138131 otu_2844 otu_31159 otu_26083 otu_25167
otu_35501 otu_84079 otu_106729 otu_106986 otu_151959 otu_109287 otu_30715
otu_25421 otu_6846 otu_151705 otu_142158 otu_22680 otu_141903 otu_78959
otu_126579 otu_105918 otu_23626 otu_109108 otu_25930 otu_144335 otu_22509
otu_108048 otu_146431 otu_79664 otu_108788 otu_140775 otu_24530 otu_152386
otu_143915 otu_28983 otu_110301 otu_79051 otu_141919 otu_24862 otu_126785
otu_143812 otu_4840 otu_152174 otu_35190 otu_36942 otu_107010 otu_83165
otu_106095 otu_3217 otu_103347 otu_24698 otu_2197 otu_83678 otu_32089 otu_106244
otu_84261 otu_80256 otu_83733 otu_24162 otu_81441 otu_143902 otu_23551 otu_25646
otu_27348 otu_141998 otu_107810 otu_105988 otu_30136 otu_24447 otu_5313
otu_144275 otu_83530 otu_23366 otu_27832 otu_106142 otu_107870 otu_106058
otu_102836 otu_26981 otu_106662 otu_3254 otu_22376 otu_24599 otu_24460
otu_106417 otu_79724 otu_25725 otu_79138 otu_134364 otu_4532 otu_35034
[illegible]

		otu_25421 otu_6846 otu_151705 otu_142158 otu_22680 otu_141903 otu_78959<br>otu_126579 otu_105918 otu_23626 otu_109108 otu_25930 otu_144335 otu_22509<br>otu_108048 otu_146431 otu_79664 otu_108788 otu_140775 otu_24530 otu_152386<br>otu_143915 otu_28983 otu_110301 otu_79051 otu_141919 otu_24862 otu_126785<br>otu_143812 otu_4840 otu_152174 otu_35190 otu_36942 otu_107010 otu_83165<br>otu_106095 otu_3217 otu_103347 otu_24698 otu_2197 otu_83678 otu_32089 otu_106244<br>otu_84261 otu_80256 otu_83733 otu_24162 otu_81441 otu_143902 otu_23551 otu_25646<br>otu_27348 otu_141998 otu_107810 otu_105988 otu_30136 otu_24447 otu_5313<br>otu_144275 otu_83530 otu_23366 otu_27832 otu_106142 otu_107870 otu_106058<br>otu_102836 otu_26981 otu_106662 otu_3254 otu_22376 otu_24599 otu_24460<br>otu_106417 otu_79724 otu_25725 otu_79138 otu_134364 otu_4532 otu_35034<br>otu_143843 otu_34689 otu_3723 otu_28188 otu_3413 otu_132601 otu_2571 otu_103332<br>otu_108874 otu_27971 otu_108796 otu_136958 otu_5081 otu_147135 otu_107774<br>otu_4268 otu_25741 otu_106228 otu_80796 otu_137086 otu_79849 otu_108328<br>otu_150202 otu_108471 otu_33075 otu_4439 otu_31690 otu_80505 otu_80897 otu_99225<br>otu_96892 otu_107012 otu_24184 otu_108083 otu_80642 otu_25202 otu_4510 otu_99518<br>otu_35183 otu_126540 otu_106113 otu_79481 otu_80855 otu_102826 otu_154253<br>otu_32101 otu_108662 otu_99490 otu_108905 otu_29993 otu_107068 otu_99622<br>otu_23421 otu_82582 otu_142430 otu_106823 otu_540 otu_103199 otu_7288 otu_150848<br>otu_80070 otu_83174 otu_82397 otu_23142 otu_107252 otu_36344 otu_30800<br>otu_137065 otu_28480 otu_81642 otu_154565 otu_24910 otu_107538 otu_28568<br>otu_153952 otu_28139 otu_108274 otu_81135 otu_29711 otu_99870 otu_33769 otu_99424<br>otu_134737 otu_151392 otu_136087 otu_82073 otu_665 otu_4309 otu_152245<br>otu_142054 otu_26886 otu_150859 otu_152716 otu_4051 otu_150426 otu_3165<br>otu_133103 otu_154765 otu_147349 otu_140822 otu_126822 otu_22920 otu_110332<br>otu_26315 otu_3102 otu_81349 otu_108598 otu_106434 otu_97041 otu_79878 otu_33291<br>otu_31408 otu_153822 otu_23995 otu_79807 otu_139562 otu_26762 otu_107983<br>otu_24731 otu_34418 otu_33893 otu_132737 otu_2202 otu_110079 otu_2445 otu_22771<br>otu_80677 otu_139171 otu_32767 otu_108546 otu_82139 otu_144423 otu_25486<br>otu_96703 otu_21 otu_147013 otu_26891 otu_83114 otu_150464 otu_4797 otu_28939<br>otu_25819 otu_6659 otu_106702 otu_5122 otu_146861 otu_107819 otu_141991<br>otu_154547 otu_34834 otu_151883 otu_3725 otu_33851 otu_3420 otu_110074 otu_24993<br>otu_136954 otu_153060 otu_146744 otu_109397 otu_27497 otu_28606 otu_109239<br>otu_24694 otu_151688 otu_151002 otu_102805 otu_151754 otu_107501 otu_141895<br>otu_106675 otu_132663 otu_23737 otu_82527 otu_142498 otu_28786 otu_23910<br>otu_106769 otu_28028 otu_32280 otu_107220 otu_528 otu_146508 otu_2949 otu_2392<br>otu_27654 otu_25997 otu_151983 otu_30269 otu_36119 otu_27161 otu_134680<br>otu_23814 otu_2957 otu_26720 otu_108373 otu_24276 otu_83256 otu_24051 otu_81685<br>otu_28261 otu_105610 otu_29235 otu_153007 otu_152802 otu_83852 otu_151615<br>otu_152196 otu_143776 otu_4427 otu_99879 otu_107468 otu_80761 otu_127069<br>otu_26384 otu_23284 otu_22938 otu_25003 otu_110455 otu_81647 otu_141940<br>otu_152281 otu_26093 otu_32922 otu_26267 otu_2859 otu_80396 otu_2653 otu_150975<br>otu_81558 otu_2965 otu_25703 otu_3096 otu_142128 otu_5453 otu_3798 otu_29337<br>otu_103078 otu_108086 otu_80164 otu_26568 otu_30648 otu_139769 otu_103402<br>otu_143817 otu_3863 otu_2300 otu_146716 otu_22508 otu_127404 otu_142387<br>otu_30476 otu_4502 otu_22369 otu_150182 otu_107185 otu_3295 otu_127038<br>otu_150548 otu_106110 otu_4094 otu_151773 otu_154237 otu_110193 otu_99493<br>otu_24262 otu_106579 otu_107369 otu_2500 otu_105883 otu_5804 otu_27379 otu_80251<br>otu_150618 otu_30394 otu_32852 otu_146934 otu_34049 otu_24825 otu_6545 otu_99479<br>otu_83303 otu_31149 otu_164029 otu_153298 otu_146657 otu_105525 otu_126722<br>otu_132540 otu_143795 otu_28453 otu_29018 otu_107526 otu_28782 otu_27069<br>otu_84013 otu_105499 otu_144328 otu_143825 otu_3136 otu_153154 otu_81336<br>otu_99381 otu_105940 otu_82751 otu_139256 otu_151965 otu_134561 otu_25291<br>otu_132470 otu_4191 otu_99883 otu_24421 otu_146527 otu_108163 otu_28183<br>otu_34378 otu_26732 otu_81212 otu_102842 otu_25395 otu_150341 otu_30714<br>otu_152166 otu_99294 otu_137077 otu_126856 otu_147142 otu_33386 otu_24666<br>otu_3223 otu_152956 otu_83057 otu_140876 otu_397 otu_150095 otu_139797 otu_81520<br>otu_105400 otu_24338 otu_36872 otu_152768 otu_32928 otu_2240 otu_126463<br>otu_36962 otu_23825 otu_79236 otu_147503 otu_28524 otu_150584 otu_865 otu_78943<br>otu_23843 otu_25258 otu_32125 otu_29759 otu_106487 otu_24885 otu_36470 otu_82617<br>otu_79058 otu_126945 otu_109320 otu_34243 otu_107163 otu_109455 otu_139620<br>otu_31981 otu_27749 otu_146595 otu_154173 otu_28627 otu_2249 otu_26996 otu_2570<br>otu_26674 otu_134367 otu_23795 otu_27935 otu_108385 otu_5810 otu_30311 otu_79113<br>otu_146448 otu_142382 otu_22537 otu_23778 otu_4819 otu_83954 otu_22612 otu_24161<br>otu_127388 otu_134310 otu_830 otu_23313 otu_79972 otu_22404 otu_3115 otu_144256<br>otu_79950 otu_28876 otu_4998 otu_151788 otu_36120 otu_134333 otu_4985 otu_4988<br>otu_27340 otu_147190 otu_25041 otu_83251 otu_34345 otu_5767 otu_507 otu_23666<br>otu_3151 otu_23481 otu_5930 otu_140931 otu_99539 otu_80700 otu_34697 otu_25177<br>otu_3918 otu_150176 otu_151321 otu_142406 otu_4775 otu_99496 otu_126516<br>otu_23020 otu_152250 otu_146763 otu_96801 otu_33552 otu_22571 otu_107899<br>otu_78971 otu_5635 otu_3658 otu_152769 otu_136080 otu_3631 otu_26638 otu_26652<br>otu_153163 otu_3689 otu_103032 otu_97018 otu_34341 otu_144469 otu_3480 otu_81913<br>otu_490 otu_103119 otu_106151 otu_79179 otu_3847 otu_107031 otu_103047 otu_79492<br>otu_23067 otu_147353 otu_107442 otu_4794 otu_78952 otu_5341 otu_84262 otu_25739<br>otu_34843 otu_25514 otu_126596 otu_79270 otu_134553 otu_26715 otu_126718<br>otu_34858 otu_132474 otu_27411 otu_2743 otu_96688 otu_107586 otu_30849<br>otu_151041 otu_7149 otu_28166 otu_151486 otu_102849 otu_80189 otu_79072<br>otu_143959 otu_2635 otu_2555 otu_3541 otu_80117 otu_103011 otu_27606 otu_79035<br>otu_141797 otu_133113 otu_150989 otu_139220 otu_29808 otu_99906 otu_23854<br>otu_25910 otu_29941 otu_27274 otu_107227 otu_140786 otu_151067 otu_28719<br>otu_97205 otu_2698 otu_30619 otu_26601 otu_23285 otu_32439 otu_26903 otu_29926<br>otu_173 otu_4738 otu_23746 otu_79852 otu_32239 otu_33987 otu_22349 otu_103194<br>otu_151455 otu_103092 otu_96918 otu_6828 otu_24855 otu_132582 otu_80723<br>otu_143829 otu_105713 otu_99378 otu_102949 otu_30157 otu_31756 otu_154406<br>otu_27836 otu_81550 otu_28652 otu_2325 otu_134427 otu_110431 otu_106015<br>otu_133043 otu_108740 otu_27668 otu_34566 otu_35806 otu_32941 otu_23641<br>otu_107300 otu_24679 otu_150746 otu_142459 otu_7083 otu_26379 otu_32478<br>otu_151239 otu_152872 otu_106797 otu_2560 otu_31680 otu_4996 otu_153045<br>otu_153055 otu_34782 otu_106322 otu_2459 otu_2740 otu_134384 otu_126813<br>otu_24250 otu_24466 otu_33661 otu_103275 otu_30893 otu_106178 otu_151389<br>otu_105467 otu_152494 otu_34467 otu_27301 otu_108064 otu_141978
mpolyfl	2893	otu_100393 otu_147776 otu_128746 otu_128622 otu_7706 otu_41903 otu_103953<br>otu_141233 otu_139996 otu_7443 otu_111133 otu_38483 otu_7547 otu_40095 otu_103818<br>otu_110802 otu_155185 otu_8051 otu_111564 otu_84431 otu_155288 otu_37653<br>otu_97268 otu_41824 otu_128259 otu_129135 otu_40581 otu_39730 otu_112607<br>otu_112178 otu_38016 otu_40549 otu_147782 otu_38708 otu_155396 otu_142969<br>otu_7465 otu_38366 otu_139950 otu_136496 otu_115213 otu_163450 otu_38197<br>otu_84560 otu_42017 otu_41845 otu_128136 otu_100300 otu_154992 otu_156253<br>otu_155374 otu_54 otu_40940 otu_110739 otu_103939 otu_8015 otu_144559 otu_147551<br>otu_155876 otu_142741 otu_155135 otu_113643 otu_155161 otu_112453 otu_84801<br>otu_40608 otu_111620 otu_39371 otu_111217 otu_142798 otu_142783 otu_155860<br>otu_155143 otu_111963 otu_43072 otu_37634 otu_85196 otu_37313 otu_111812<br>otu_114414 otu_111841 otu_112092 otu_111648 otu_142928 otu_39275 otu_39721<br>otu_144791 otu_39880 otu_912 otu_84329 otu_110733 otu_8910 otu_84928 otu_155598<br>otu_38221 otu_139978 otu_112949 otu_154942 otu_84825 otu_85188 otu_7861<br>otu_40844 otu_111904 otu_112699 otu_103807 otu_103984 otu_85871 otu_37805<br>otu_128108 otu_147664 otu_37719 otu_40090 otu_7675 otu_7837 otu_38710 otu_8184<br>otu_41372 otu_103667 otu_8372 otu_112988 otu_8332 otu_42366 otu_155514

otu_7465 otu_38366 otu_139950 otu_136496 otu_115213 otu_163450 otu_38197
otu_84560 otu_42017 otu_41845 otu_128136 otu_100300 otu_154992 otu_156253
otu_155374 otu_54 otu_40940 otu_110739 otu_103939 otu_8015 otu_144559 otu_147551
otu_155876 otu_142741 otu_155135 otu_113643 otu_155161 otu_112453 otu_84801
otu_40608 otu_111620 otu_39371 otu_111217 otu_142798 otu_142783 otu_155860
otu_155143 otu_111963 otu_43072 otu_37634 otu_85196 otu_37313 otu_111812
otu_114414 otu_111841 otu_112092 otu_111648 otu_142928 otu_39275 otu_39721
otu_144791 otu_39880 otu_912 otu_84329 otu_110733 otu_8910 otu_84928 otu_155598
otu_38221 otu_139978 otu_112949 otu_154942 otu_84825 otu_85188 otu_7861
otu_40844 otu_111904 otu_112699 otu_103807 otu_103984 otu_85871 otu_37805
otu_128108 otu_147664 otu_37719 otu_40090 otu_7675 otu_7837 otu_38710 otu_8184
otu_41372 otu_103667 otu_8372 otu_112988 otu_8332 otu_42366 otu_155514
otu_155038 otu_142832 otu_156102 otu_155055 otu_41881 otu_111823 otu_110886
otu_38338 otu_110910 otu_7585 otu_111890 otu_100199 otu_85857 otu_41391 otu_84944
otu_84979 otu_155249 otu_111966 otu_39031 otu_144569 otu_113171 otu_111121
otu_133225 otu_137890 otu_155901 otu_39557 otu_155585 otu_112762 otu_142725
otu_42112 otu_8568 otu_84788 otu_38686 otu_155071 otu_138211 otu_103719 otu_38713
otu_42470 otu_113323 otu_144772 otu_39580 otu_111978 otu_38762 otu_128201
otu_37466 otu_155683 otu_144643 otu_139969 otu_164314 otu_38593 otu_7578
otu_110841 otu_155227 otu_137365 otu_39853 otu_112517 otu_84594 otu_40015
otu_147637 otu_84951 otu_136175 otu_141166 otu_111630 otu_113606 otu_7898
otu_112395 otu_155548 otu_41840 otu_112419 otu_100239 otu_38853 otu_41516
otu_38669 otu_84735 otu_110936 otu_8007 otu_155131 otu_111597 otu_37517
otu_155453 otu_8208 otu_111044 otu_84292 otu_112016 otu_147627 otu_111937
otu_38845 otu_147567 otu_8194 otu_112494 otu_38514 otu_38823 otu_84596 otu_111545
otu_8362 otu_111387 otu_7627 otu_110869 otu_155961 otu_155307 otu_111184
otu_42529 otu_115009 otu_37628 otu_137355 otu_37763 otu_111049 otu_147720
otu_128067 otu_155265 otu_144645 otu_84451 otu_111877 otu_139262 otu_144579
otu_38984 otu_110730 otu_85781 otu_112589 otu_155512 otu_37878 otu_8775
otu_142786 otu_42381 otu_110815 otu_111585 otu_38288 otu_37810 otu_112389
otu_42303 otu_40391 otu_147561 otu_7742 otu_112500 otu_37639 otu_39997 otu_84896
otu_112462 otu_97397 otu_40169 otu_110823 otu_41348 otu_155563 otu_38433
otu_84360 otu_111445 otu_41183 otu_7655 otu_39018 otu_84561 otu_110842 otu_38390
otu_38376 otu_155213 otu_37268 otu_113767 otu_41331 otu_43409 otu_39869
otu_141162 otu_112781 otu_41140 otu_139297 otu_111537 otu_110902 otu_111307
otu_115307 otu_39439 otu_110746 otu_84880 otu_147802 otu_114744 otu_112869
otu_142892 otu_111759 otu_38073 otu_147522 otu_128389 otu_133178 otu_111982
otu_8229 otu_37498 otu_100195 otu_140043 otu_113689 otu_155794 otu_110919
otu_136783 otu_103804 otu_7940 otu_38602 otu_113996 otu_111779 otu_142758
otu_8301 otu_154964 otu_154981 otu_8078 otu_111600 otu_110879 otu_103940
otu_39367 otu_7515 otu_42250 otu_7532 otu_103839 otu_84305 otu_100380 otu_111237
otu_113574 otu_8153 otu_37751 otu_142858 otu_39704 otu_114451 otu_7793 otu_155875
otu_147632 otu_111641 otu_128690 otu_103933 otu_7879 otu_7997 otu_38844
otu_104068 otu_112200 otu_38117 otu_154972 otu_155650 otu_85606 otu_85216
otu_40550 otu_41633 otu_141205 otu_112533 otu_103803 otu_155332 otu_41542
otu_110953 otu_38473 otu_154906 otu_128098 otu_85220 otu_40106 otu_8001
otu_147641 otu_40992 otu_112930 otu_142749 otu_39050 otu_8463 otu_103692
otu_41657 otu_154982 otu_155602 otu_112236 otu_85051 otu_110754 otu_37968
otu_37708 otu_154952 otu_142747 otu_155101 otu_42875 otu_110665 otu_42673
otu_110821 otu_37433 otu_139985 otu_147746 otu_41994 otu_84501 otu_114167
otu_7582 otu_112264 otu_39492 otu_37846 otu_7729 otu_84390 otu_139267 otu_84660
otu_155444 otu_128357 otu_38091 otu_103808 otu_137360 otu_38053 otu_133175
otu_134827 otu_111111 otu_155107 otu_111093 otu_110714 otu_38093 otu_155541
otu_8059 otu_42013 otu_147630 otu_7436 otu_37638 otu_111559 otu_39241 otu_8333
otu_100383 otu_7623 otu_37620 otu_155889 otu_97318 otu_144621 otu_128087
otu_155069 otu_39036 otu_104090 otu_38664 otu_142829 otu_128720 otu_142732
otu_111721 otu_114222 otu_155668 otu_112002 otu_155065 otu_144678 otu_37747
otu_37385 otu_84652 otu_85440 otu_112360 otu_40167 otu_155386 otu_115122
otu_142912 otu_103925 otu_8975 otu_7643 otu_110759 otu_100221 otu_112573
otu_85192 otu_133206 otu_38427 otu_41772 otu_134875 otu_7576 otu_39451
otu_110991 otu_114452 otu_110872 otu_155346 otu_7691 otu_155197 otu_113597
otu_113870 otu_84344 otu_110946 otu_110676 otu_43724 otu_38214 otu_42387
otu_39360 otu_38607 otu_38310 otu_155208 otu_41433 otu_39426 otu_43048 otu_44122
otu_999 otu_40460 otu_103865 otu_85068 otu_41500 otu_111689 otu_7510 otu_40098
otu_112887 otu_111625 otu_155241 otu_41417 otu_147592 otu_115208 otu_147737
otu_38797 otu_1003 otu_37549 otu_37702 otu_128130 otu_112845 otu_37282 otu_111357
otu_37265 otu_110981 otu_41853 otu_155705 otu_38182 otu_8335 otu_41562 otu_43711
otu_154973 otu_7902 otu_37698 otu_43760 otu_155559 otu_37333 otu_113457
otu_38322 otu_128069 otu_38478 otu_103913 otu_84734 otu_155175 otu_111315
otu_38066 otu_39100 otu_8256 otu_137349 otu_164293 otu_9097 otu_156454
otu_103721 otu_114971 otu_113921 otu_103853 otu_37752 otu_155325 otu_112358
otu_103783 otu_155990 otu_40871 otu_112142 otu_128215 otu_103903 otu_111486
otu_110752 otu_144588 otu_111319 otu_154965 otu_144663 otu_111400 otu_41461
otu_155043 otu_37377 otu_38176 otu_139968 otu_38165 otu_155842 otu_39020
otu_40304 otu_155133 otu_111806 otu_40368 otu_8454 otu_144710 otu_42176 otu_39406
otu_128549 otu_147740 otu_38674 otu_110867 otu_38474 otu_155333 otu_41321
otu_155311 otu_155537 otu_113584 otu_156179 otu_37658 otu_7964 otu_155781
otu_110709 otu_8890 otu_110726 otu_38363 otu_128661 otu_41049 otu_84824 otu_39300
otu_111679 otu_111114 otu_85447 otu_104113 otu_7554 otu_40813 otu_147519 otu_8170
otu_84428 otu_42974 otu_7591 otu_100198 otu_112216 otu_155884 otu_39891
otu_37608 otu_7544 otu_85160 otu_155487 otu_8318 otu_41901 otu_141163 otu_42314
otu_112337 otu_111361 otu_137665 otu_114472 otu_110856 otu_111470 otu_147813
otu_38596 otu_37240 otu_103924 otu_155509 otu_44083 otu_38571 otu_110783
otu_111075 otu_37710 otu_111799 otu_8299 otu_155904 otu_42588 otu_39583 otu_42119
otu_156155 otu_85100 otu_38289 otu_155360 otu_37367 otu_155471 otu_113954
otu_37239 otu_39712 otu_110697 otu_38749 otu_37833 otu_7736 otu_155205 otu_42105
otu_42348 otu_147541 otu_142796 otu_155144 otu_138657 otu_8689 otu_84820
otu_128362 otu_111131 otu_111312 otu_128299 otu_41931 otu_40636 otu_110794
otu_128066 otu_147724 otu_139994 otu_39806 otu_38030 otu_8193 otu_147617
otu_39597 otu_155165 otu_42059 otu_155710 otu_111575 otu_41426 otu_154990
otu_114696 otu_38155 otu_155228 otu_7639 otu_8791 otu_84488 otu_7451 otu_38502
otu_8008 otu_163970 otu_133170 otu_38958 otu_37852 otu_7731 otu_39325 otu_40962
otu_42229 otu_97279 otu_43561 otu_39779 otu_84783 otu_37989 otu_112226
otu_155732 otu_155852 otu_112354 otu_85530 otu_112232 otu_39861 otu_7464
otu_40314 otu_7708 otu_38543 otu_111029 otu_111720 otu_7866 otu_100200 otu_37748
otu_141270 otu_147515 otu_134826 otu_947 otu_147538 otu_43513 otu_100183
otu_37480 otu_147800 otu_155859 otu_110926 otu_8151 otu_155277 otu_115342
otu_8636 otu_39433 otu_110677 otu_7534 otu_41863 otu_84659 otu_38067 otu_7637
otu_43429 otu_42817 otu_7905 otu_8171 otu_38870 otu_8046 otu_37363 otu_37393
otu_85253 otu_154940 otu_39958 otu_113525 otu_38712 otu_38825 otu_110685
otu_128347 otu_8139 otu_110982 otu_84686 otu_111181 otu_112796 otu_37621
otu_142771 otu_113406 otu_112108 otu_100325 otu_8278 otu_111068 otu_84446
otu_155075 otu_155418 otu_41868 otu_147767 otu_39574 otu_113789 otu_40989
otu_111964 otu_39397 otu_41133 otu_128303 otu_41014 otu_112534 otu_37778
otu_112747 otu_37761 otu_134833 otu_103712 otu_8789 otu_39618 otu_38082
otu_111205 otu_155571 otu_970 otu_85864 otu_112088 otu_38234 otu_111135
otu_111638 otu_144550 otu_113097 otu_113064 otu_110947 otu_41190 otu_37940
otu_156225 otu_41557 otu_138651 otu_100215 otu_103764 otu_100266 otu_137892
otu_42048 otu_142812 otu_110681 otu_37515 otu_111744 otu_111607 otu_111722

otu_43429 otu_42817 otu_7905 otu_8171 otu_38870 otu_8046 otu_37363 otu_37393
otu_85253 otu_154940 otu_39958 otu_113525 otu_38712 otu_38825 otu_110685
otu_128347 otu_8139 otu_110982 otu_84686 otu_111181 otu_112796 otu_37621
otu_142771 otu_113406 otu_112108 otu_100325 otu_8278 otu_111068 otu_84446
otu_155075 otu_155418 otu_41868 otu_147767 otu_39574 otu_113789 otu_40989
otu_111964 otu_39397 otu_41133 otu_128303 otu_41014 otu_112534 otu_37778
otu_112747 otu_37761 otu_134833 otu_103712 otu_8789 otu_39618 otu_38082
otu_111205 otu_155571 otu_970 otu_85864 otu_112088 otu_38234 otu_111135
otu_111638 otu_144550 otu_113097 otu_113064 otu_110947 otu_41190 otu_37940
otu_156225 otu_41557 otu_138651 otu_100215 otu_103764 otu_100266 otu_137892
otu_42048 otu_142812 otu_110681 otu_37515 otu_111744 otu_111607 otu_111722
otu_110938 otu_128660 otu_111522 otu_8618 otu_111788 otu_41614 otu_155986
otu_141235 otu_962 otu_111454 otu_111423 otu_111389 otu_113276 otu_40926
otu_112356 otu_155581 otu_7595 otu_128805 otu_110818 otu_155723 otu_42404
otu_37278 otu_103746 otu_8603 otu_8715 otu_113712 otu_40340 otu_84747 otu_112519
otu_39538 otu_7624 otu_111566 otu_37685 otu_155140 otu_142821 otu_38364
otu_155553 otu_41488 otu_39402 otu_142847 otu_113640 otu_103890 otu_113547
otu_112522 otu_128700 otu_142766 otu_40979 otu_154993 otu_103965 otu_155863
otu_37887 otu_8934 otu_114445 otu_37430 otu_112725 otu_111765 otu_41436 otu_84516
otu_156385 otu_38356 otu_144660 otu_39227 otu_914 otu_154907 otu_38300 otu_40215
otu_110862 otu_97357 otu_112593 otu_37890 otu_113894 otu_41618 otu_155257
otu_155505 otu_111250 otu_39484 otu_163446 otu_103908 otu_42268 otu_7921
otu_37571 otu_115069 otu_147598 otu_39166 otu_128715 otu_39903 otu_950 otu_85683
otu_37969 otu_7455 otu_128124 otu_41219 otu_113639 otu_85460 otu_113816 otu_39546
otu_154966 otu_84958 otu_40468 otu_147785 otu_111730 otu_144613 otu_112446
otu_155708 otu_113073 otu_113391 otu_84468 otu_100232 otu_37542 otu_39588
otu_100181 otu_113304 otu_113216 otu_141186 otu_37481 otu_138639 otu_128797
otu_41063 otu_103841 otu_38874 otu_111718 otu_38068 otu_155596 otu_85140
otu_37525 otu_8076 otu_155701 otu_111928 otu_155096 otu_41763 otu_111362
otu_39152 otu_113199 otu_155122 otu_84330 otu_111026 otu_144596 otu_128423
otu_111054 otu_114826 otu_110712 otu_137895 otu_38400 otu_38839 otu_43883
otu_7766 otu_975 otu_85161 otu_38548 otu_113437 otu_111777 otu_84858 otu_103674
otu_38025 otu_112997 otu_38639 otu_139936 otu_97256 otu_39312 otu_43531
otu_144563 otu_41804 otu_155916 otu_7725 otu_110998 otu_40588 otu_128365
otu_155530 otu_110874 otu_112135 otu_85208 otu_42438 otu_111693 otu_37919
otu_38038 otu_40261 otu_128260 otu_7482 otu_147629 otu_156194 otu_8049
otu_155394 otu_8018 otu_112373 otu_112289 otu_9053 otu_37695 otu_38413 otu_8329
otu_110727 otu_155050 otu_112569 otu_147557 otu_112431 otu_133179 otu_934
otu_37238 otu_37672 otu_147603 otu_147657 otu_112605 otu_38368 otu_141190
otu_85679 otu_111046 otu_37686 otu_37510 otu_38384 otu_112227 otu_111576
otu_111521 otu_128503 otu_37447 otu_39243 otu_100357 otu_100208 otu_103992
otu_39261 otu_41007 otu_7463 otu_7827 otu_155305 otu_111226 otu_7819 otu_155411
otu_38264 otu_38924 otu_41749 otu_8215 otu_110943 otu_112483 otu_39454 otu_41795
otu_38041 otu_38456 otu_41467 otu_113370 otu_42639 otu_111839 otu_128493
otu_39126 otu_37961 otu_38460 otu_112670 otu_7495 otu_110793 otu_84314 otu_7840
otu_142833 otu_112281 otu_37814 otu_37655 otu_7500 otu_147616 otu_7744
otu_142737 otu_111692 otu_38864 otu_85423 otu_113935 otu_84727 otu_112947
otu_43006 otu_141168 otu_111422 otu_128241 otu_112220 otu_7551 otu_111130
otu_37457 otu_84648 otu_110833 otu_155203 otu_142728 otu_37975 otu_85808
otu_43610 otu_133222 otu_155199 otu_38741 otu_155141 otu_85035 otu_38756
otu_147626 otu_39927 otu_37802 otu_144688 otu_85486 otu_128284 otu_111147
otu_111613 otu_112724 otu_155019 otu_114238 otu_37769 otu_41794 otu_40534
otu_37641 otu_41387 otu_8289 otu_37570 otu_113146 otu_37477 otu_8182 otu_110801
otu_128461 otu_142752 otu_41478 otu_144558 otu_155056 otu_37383 otu_155304
otu_41264 otu_155792 otu_38271 otu_141196 otu_114986 otu_111543 otu_40786
otu_37716 otu_84667 otu_156376 otu_112302 otu_115215 otu_7614 otu_43164 otu_41538
otu_84823 otu_139290 otu_111787 otu_113191 otu_155727 otu_104037 otu_37528
otu_147735 otu_155157 otu_155280 otu_8039 otu_38894 otu_39863 otu_43766
otu_154963 otu_110988 otu_110826 otu_155173 otu_40068 otu_128079 otu_128812
otu_110838 otu_42642 otu_112526 otu_38337 otu_84368 otu_141252 otu_103911
otu_112759 otu_39250 otu_147609 otu_8644 otu_43330 otu_139284 otu_142734
otu_155248 otu_113929 otu_37645 otu_113297 otu_113163 otu_111604 otu_128243
otu_39409 otu_37598 otu_134851 otu_142740 otu_37355 otu_38329 otu_114673
otu_144634 otu_110868 otu_37500 otu_155406 otu_110663 otu_103955 otu_154975
otu_97429 otu_97373 otu_7486 otu_7728 otu_111134 otu_40807 otu_37777 otu_7466
otu_139983 otu_84397 otu_155624 otu_139991 otu_42471 otu_41310 otu_144571
otu_40603 otu_37951 otu_40366 otu_85083 otu_111229 otu_110791 otu_128444 otu_8150
otu_7698 otu_38071 otu_142976 otu_113031 otu_112266 otu_39123 otu_112948
otu_111154 otu_114147 otu_958 otu_43438 otu_110965 otu_112728 otu_85383 otu_39248
otu_37410 otu_38512 otu_111172 otu_84654 otu_7847 otu_84564 otu_37615 otu_110930
otu_128648 otu_164294 otu_42762 otu_128556 otu_128414 otu_40828 otu_41923
otu_111683 otu_142742 otu_40546 otu_38594 otu_8192 otu_155030 otu_111056
otu_155397 otu_7524 otu_41675 otu_112114 otu_42211 otu_112372 otu_111508
otu_110703 otu_110887 otu_939 otu_84637 otu_97347 otu_85818 otu_84766 otu_42417
otu_38440 otu_85107 otu_113896 otu_137356 otu_147721 otu_40361 otu_133273
otu_7841 otu_38345 otu_38630 otu_7863 otu_111242 otu_144557 otu_7577 otu_113401
otu_37306 otu_38290 otu_41047 otu_42747 otu_110837 otu_128973 otu_38116 otu_7787
otu_128068 otu_128528 otu_155423 otu_39991 otu_7738 otu_44000 otu_111090
otu_40632 otu_142776 otu_37945 otu_37360 otu_112857 otu_155678 otu_42561
otu_7669 otu_37267 otu_8840 otu_42153 otu_41598 otu_144630 otu_37237 otu_7518
otu_7714 otu_39930 otu_128723 otu_39613 otu_114029 otu_113862 otu_155110 otu_7561
otu_97351 otu_8678 otu_136499 otu_8441 otu_37743 otu_129000 otu_7470 otu_155561
otu_37299 otu_39457 otu_8829 otu_154918 otu_7859 otu_43518 otu_97290 otu_38242
otu_100228 otu_84707 otu_111556 otu_147653 otu_37704 otu_38008 otu_85115
otu_156128 otu_114333 otu_155524 otu_112154 otu_38307 otu_39061 otu_110722
otu_84693 otu_147683 otu_39103 otu_128609 otu_39535 otu_84581 otu_103799
otu_103973 otu_40220 otu_100347 otu_112144 otu_156273 otu_110865 otu_37821
otu_38227 otu_112259 otu_111032 otu_155023 otu_8671 otu_103884 otu_114597
otu_111754 otu_137114 otu_7556 otu_42693 otu_142730 otu_104109 otu_8782 otu_37536
otu_84849 otu_84321 otu_155106 otu_147599 otu_100423 otu_111124 otu_37808
otu_154905 otu_129096 otu_41848 otu_7888 otu_84297 otu_85443 otu_144703
otu_39142 otu_142759 otu_37678 otu_114563 otu_111246 otu_112115 otu_111662
otu_41082 otu_112065 otu_7933 otu_128190 otu_7893 otu_8847 otu_946 otu_112004
otu_84687 otu_40236 otu_37997 otu_37349 otu_139973 otu_85301 otu_110678
otu_142804 otu_84865 otu_7720 otu_111176 otu_155181 otu_155489 otu_7966 otu_7948
otu_154957 otu_155743 otu_114011 otu_40928 otu_111509 otu_144679 otu_84806
otu_37889 otu_112542 otu_147655 otu_147512 otu_112235 otu_155338 otu_111817
otu_111072 otu_142806 otu_104046 otu_37416 otu_147547 otu_37707 otu_147528
otu_8659 otu_111040 otu_155601 otu_164297 otu_144620 otu_155191 otu_128086
otu_137361 otu_155837 otu_85314 otu_41222 otu_8204 otu_128250 otu_38258
otu_142755 otu_7883 otu_100376 otu_128991 otu_936 otu_115258 otu_155551
otu_111667 otu_37705 otu_115103 otu_155138 otu_111442 otu_37978 otu_104027
otu_142768 otu_113005 otu_134822 otu_84300 otu_8022 otu_8094 otu_84941 otu_8487
otu_39560 otu_97355 otu_40213 otu_42532 otu_41006 otu_155066 otu_142731
otu_111152 otu_40190 otu_155627 otu_128702 otu_100209 otu_7497 otu_110811
otu_114058 otu_113504 otu_112441 otu_9113 otu_155011 otu_154995 otu_7968
otu_156124 otu_113035 otu_949 otu_84622 otu_111110 otu_112705 otu_993 otu_38198
otu_112401 otu_84710 otu_84736 otu_37897 otu_84518 otu_111489 otu_155235
otu_110954 otu_41793 otu_142853 otu_163132 otu_155164 otu_39136 otu_39498

otu_111072 otu_142806 otu_104046 otu_37416 otu_147547 otu_37707 otu_147528
otu_8659 otu_111040 otu_155601 otu_164297 otu_144620 otu_155191 otu_128086
otu_137361 otu_155837 otu_85314 otu_41222 otu_8204 otu_128250 otu_38258
otu_142755 otu_7883 otu_100376 otu_128991 otu_936 otu_115258 otu_155551
otu_111667 otu_37705 otu_115103 otu_155138 otu_111442 otu_37978 otu_104027
otu_142768 otu_113005 otu_134822 otu_84300 otu_8022 otu_8094 otu_84941 otu_8487
otu_39560 otu_97355 otu_40213 otu_42532 otu_41006 otu_155066 otu_142731
otu_111152 otu_40190 otu_155627 otu_128702 otu_100209 otu_7497 otu_110811
otu_114058 otu_113504 otu_112441 otu_9113 otu_155011 otu_154995 otu_7968
otu_156124 otu_113035 otu_949 otu_84622 otu_111110 otu_112705 otu_993 otu_38198
otu_112401 otu_84710 otu_84736 otu_37897 otu_84518 otu_111489 otu_155235
otu_110954 otu_41793 otu_142853 otu_163432 otu_155164 otu_39136 otu_39498
otu_111533 otu_163776 otu_113900 otu_110675 otu_112254 otu_111144 otu_142792
otu_110688 otu_85541 otu_37283 otu_40071 otu_155209 otu_137111 otu_8122 otu_42961
otu_40011 otu_39307 otu_111368 otu_40791 otu_155125 otu_38282 otu_111316
otu_128101 otu_40200 otu_37339 otu_110929 otu_139975 otu_39095 otu_113610
otu_41852 otu_37614 otu_156110 otu_85255 otu_113324 otu_41165 otu_155671
otu_113763 otu_8711 otu_85672 otu_147728 otu_38409 otu_111465 otu_128853 otu_925
otu_163429 otu_141176 otu_141210 otu_155217 otu_112499 otu_155619 otu_37334
otu_155429 otu_140008 otu_38426 otu_114475 otu_7773 otu_7803 otu_39211 otu_39380
otu_39700 otu_112056 otu_38291 otu_38113 otu_112630 otu_38125 otu_84343 otu_84413
otu_155080 otu_111037 otu_7727 otu_141181 otu_38980 otu_37356 otu_42901 otu_37494
otu_111161 otu_110790 otu_111379 otu_112369 otu_38070 otu_84913 otu_112507
otu_8181 otu_113992 otu_155637 otu_37399 otu_37868 otu_144561 otu_114657
otu_134815 otu_142850 otu_113090 otu_39514 otu_7633 otu_42862 otu_8294 otu_111199
otu_155105 otu_38392 otu_100375 otu_38362 otu_8126 otu_112064 otu_38325
otu_111527 otu_155966 otu_7856 otu_7663 otu_8025 otu_43499 otu_38907 otu_111324
otu_39088 otu_39011 otu_112174 otu_128447 otu_8891 otu_111494 otu_8327 otu_42987
otu_111129 otu_38994 otu_114911 otu_40099 otu_112433 otu_85127 otu_111590
otu_40780 otu_37630 otu_44012 otu_44152 otu_37440 otu_128239 otu_111858
otu_112336 otu_38371 otu_139981 otu_112791 otu_112435 otu_112371 otu_37609
otu_128717 otu_40049 otu_114234 otu_111610 otu_40900 otu_113049 otu_37573
otu_112990 otu_110720 otu_154929 otu_114724 otu_38100 otu_111973 otu_40189
otu_40225 otu_956 otu_84770 otu_97400 otu_42788 otu_7461 otu_84523 otu_42129
otu_38133 otu_112659 otu_114349 otu_110860 otu_111485 otu_8310 otu_38306
otu_111944 otu_41773 otu_85003 otu_7979 otu_1015 otu_112582 otu_40419 otu_7889
otu_111279 otu_37350 otu_39377 otu_37892 otu_111671 otu_111699 otu_139962
otu_39874 otu_111609 otu_154938 otu_112049 otu_155328 otu_8641 otu_41624
otu_147840 otu_40919 otu_42091 otu_84365 otu_111089 otu_110683 otu_111932
otu_37556 otu_110882 otu_8302 otu_38330 otu_147809 otu_7589 otu_111707 otu_142777
otu_39297 otu_155245 otu_155171 otu_9030 otu_8889 otu_111894 otu_84879 otu_104120
otu_7658 otu_8734 otu_110976 otu_97276 otu_38793 otu_147574 otu_142754 otu_111311
otu_128707 otu_155975 otu_103734 otu_38951 otu_147660 otu_111605 otu_112627
otu_42000 otu_110928 otu_147581 otu_139974 otu_155060 otu_154956 otu_981
otu_42665 otu_38866 otu_37793 otu_38550 otu_39283 otu_8754 otu_84418 otu_84757
otu_112773 otu_37642 otu_84375 otu_7521 otu_38766 otu_154924 otu_909 otu_8280
otu_84545 otu_155907 otu_8670 otu_38036 otu_128903 otu_155470 otu_113082
otu_7972 otu_8818 otu_37291 otu_40124 otu_156135 otu_37883 otu_142729 otu_38740
otu_7602 otu_128367 otu_142819 otu_8047 otu_113011 otu_8029 otu_112275 otu_39528
otu_40637 otu_8493 otu_155651 otu_39441 otu_7558 otu_84447 otu_115127 otu_8081
otu_42272 otu_7985 otu_142846 otu_42132 otu_103976 otu_42397 otu_112918 otu_7802
otu_142797 otu_155472 otu_113949 otu_84303 otu_103898 otu_38277 otu_103857
otu_8271 otu_85091 otu_115273 otu_38671 otu_137661 otu_84618 otu_155493
otu_112185 otu_37579 otu_84326 otu_38145 otu_8165 otu_136772 otu_39160
otu_103725 otu_38999 otu_112167 otu_84522 otu_39304 otu_114130 otu_39316
otu_103643 otu_111451 otu_38709 otu_38892 otu_7743 otu_38455 otu_39832 otu_40732
otu_85354 otu_8888 otu_38739 otu_103845 otu_164307 otu_111132 otu_113806 otu_8161
otu_7477 otu_7779 otu_84812 otu_37999 otu_155047 otu_7548 otu_154913 otu_112333
otu_110803 otu_37469 otu_133187 otu_128344 otu_43177 otu_42213 otu_100330
otu_39341 otu_38849 otu_128945 otu_104048 otu_85363 otu_155061 otu_40968
otu_100253 otu_37456 otu_112040 otu_110925 otu_38103 otu_112378 otu_114332
otu_41968 otu_112660 otu_110701 otu_142824 otu_8249 otu_147578 otu_103690
otu_41819 otu_8761 otu_43250 otu_40284 otu_112082 otu_39151 otu_38971 otu_111281
otu_38349 otu_110742 otu_84332 otu_41513 otu_7566 otu_39442 otu_7572 otu_111013
otu_42254 otu_155290 otu_113356 otu_155072 otu_133180 otu_84412 otu_41831
otu_154917 otu_111907 otu_39276 otu_111101 otu_112632 otu_104142 otu_7763
otu_136985 otu_113417 otu_111818 otu_100218 otu_112313 otu_7886 otu_111079
otu_8656 otu_111615 otu_128397 otu_40788 otu_111653 otu_104056 otu_84573
otu_154968 otu_37665 otu_142779 otu_110963 otu_112421 otu_155070 otu_97265
otu_155752 otu_155137 otu_128150 otu_155933 otu_41591 otu_103731 otu_40702
otu_85148 otu_8117 otu_111550 otu_154941 otu_40933 otu_41048 otu_37741 otu_142898
otu_137900 otu_37994 otu_39179 otu_8230 otu_142762 otu_8074 otu_111261 otu_7645
otu_111360 otu_38315 otu_114304 otu_85044 otu_141184 otu_8277 otu_111636
otu_103740 otu_40149 otu_37647 otu_111826 otu_8913 otu_8631 otu_155764 otu_164230
otu_155448 otu_111652 otu_41361 otu_144656 otu_84458 otu_147834 otu_40041
otu_100391 otu_147596 otu_37964 otu_142736 otu_38918 otu_40423 otu_147524
otu_39133 otu_85570 otu_37255 otu_133304 otu_84405 otu_154986 otu_37953
otu_84323 otu_40260 otu_7942 otu_40959 otu_40008 otu_37958 otu_110957 otu_110767
otu_128756 otu_141214 otu_154945 otu_155404 otu_8197 otu_113924 otu_84533
otu_7884 otu_85726 otu_41936 otu_84357 otu_110894 otu_38229 otu_103649
otu_147613 otu_43877 otu_155149 otu_39509 otu_113165 otu_43415 otu_7492
otu_142820 otu_37811 otu_37624 otu_7931 otu_37437 otu_7895 otu_42304 otu_37276
otu_103706 otu_155722 otu_155834 otu_128719 otu_41929 otu_115209 otu_39478
otu_103664 otu_7778 otu_39225 otu_1006 otu_40870 otu_38901 otu_84867 otu_103842
otu_133171 otu_38343 otu_38468 otu_111578 otu_7481 otu_155450 otu_100206
otu_110967 otu_969 otu_113061 otu_141187 otu_111304 otu_114258 otu_111624
otu_154950 otu_103646 otu_111210 otu_37516 otu_38846 otu_84929 otu_155254
otu_42647 otu_41256 otu_38042 otu_41283 otu_155538 otu_115167 otu_110740
otu_112058 otu_37423 otu_155077 otu_137109 otu_43928 otu_39790 otu_111035
otu_114237 otu_114240 otu_40338 otu_40226 otu_960 otu_8784 otu_37405 otu_112710
otu_112723 otu_38914 otu_110854 otu_128704 otu_38118 otu_112348 otu_155680
otu_112730 otu_8478 otu_128240 otu_85706 otu_7748 otu_38603 otu_142811 otu_103766
otu_142757 otu_154989 otu_41776 otu_920 otu_40146 otu_112813 otu_38334 otu_8017
otu_41686 otu_84773 otu_155058 otu_40720 otu_37392 otu_110804 otu_7712 otu_84672
otu_164239 otu_41122 otu_113135 otu_155676 otu_40125 otu_37269 otu_40205
otu_84483 otu_111427 otu_37396 otu_37694 otu_8357 otu_112792 otu_40911 otu_155361
otu_38758 otu_110760 otu_155012 otu_110949 otu_104030 otu_38555 otu_39445
otu_7531 otu_141254 otu_111536 otu_40840 otu_128097 otu_8106 otu_144739 otu_38294
otu_113551 otu_128104 otu_136786 otu_112841 otu_155592 otu_7709 otu_37546
otu_155123 otu_155344 otu_8608 otu_40778 otu_154914 otu_137901 otu_37529
otu_112256 otu_140030 otu_38297 otu_41539 otu_37332 otu_41145 otu_37729
otu_111112 otu_142761 otu_155108 otu_40707 otu_163426 otu_112576 otu_85499
otu_7791 otu_113158 otu_7459 otu_110987 otu_38679 otu_84997 otu_112713 otu_39579
otu_39251 otu_7673 otu_85618 otu_144709 otu_155293 otu_128822 otu_155225
otu_128813 otu_8553 otu_142830 otu_7986 otu_142727 otu_133221 otu_133246
otu_111820 otu_41778 otu_142917 otu_155100 otu_142817 otu_154974 otu_37976
otu_111348 otu_37408 otu_142751 otu_37358 otu_155905 otu_155427 otu_128285
otu_37872 otu_133209 otu_39630 otu_111709 otu_38522 otu_111143 otu_111123
otu_140003 otu_40030 otu_155046 otu_84335 otu_8735 otu_155492 otu_38826

		[illegible]<br>otu_113551 otu_128104 otu_136786 otu_112841 otu_155592 otu_7709 otu_37546<br>otu_155123 otu_155344 otu_8608 otu_40778 otu_154914 otu_137901 otu_37529<br>otu_112256 otu_140030 otu_38297 otu_41539 otu_37332 otu_41145 otu_37729<br>otu_111112 otu_142761 otu_155108 otu_40707 otu_163426 otu_112576 otu_85499<br>otu_7791 otu_113158 otu_7459 otu_110987 otu_38679 otu_84997 otu_112713 otu_39579<br>otu_39251 otu_7673 otu_85618 otu_144709 otu_155293 otu_128822 otu_155225<br>otu_128813 otu_8553 otu_142830 otu_7986 otu_142727 otu_133221 otu_133246<br>otu_111820 otu_41778 otu_142917 otu_155100 otu_142817 otu_154974 otu_37976<br>otu_111348 otu_37408 otu_142751 otu_37358 otu_155905 otu_155427 otu_128285<br>otu_37872 otu_133209 otu_39630 otu_111709 otu_38522 otu_111143 otu_111123<br>otu_140003 otu_40030 otu_155046 otu_84335 otu_8735 otu_155492 otu_38826<br>otu_43910 otu_111433 otu_100366 otu_112686 otu_43750 otu_38688 otu_7569<br>otu_100283 otu_38235 otu_112639 otu_40733 otu_37424 otu_37715 otu_103926<br>otu_42092 otu_111119 otu_141161 otu_142864 otu_142837 otu_112334 otu_38243<br>otu_139967 otu_41940 otu_42055 otu_38316 otu_38560 otu_84819 otu_128553<br>otu_42824 otu_40703 otu_155583 otu_40336 otu_40944 otu_38947 otu_104049<br>otu_100353 otu_112067 otu_42426 otu_129092 otu_37272 otu_38111 otu_128676<br>otu_37453 otu_111249 otu_111684 otu_38178 otu_7916 otu_110704 otu_137368<br>otu_42282 otu_37699 otu_7767 otu_39007 otu_38151 otu_38989 otu_7934 otu_7978<br>otu_103919 otu_7499 otu_128358 otu_114303 otu_114718 otu_139285 otu_155247<br>otu_113105 otu_37930 otu_7598 otu_8258 otu_8798 otu_113125 otu_8024 otu_142733<br>otu_37352 otu_103782 otu_110797 otu_8859 otu_156346 otu_38239 otu_144581<br>otu_147553 otu_41997 otu_155443 otu_38260 otu_41388 otu_42189 otu_155118<br>otu_110964 otu_114204 otu_128987 otu_7922 otu_128879 otu_7525 otu_40676<br>otu_103858 otu_112386 otu_8200 otu_112740 otu_37541 otu_40206 otu_41394 otu_85145<br>otu_144638 otu_97304 otu_84489 otu_128191 otu_38187 otu_39224 otu_7678<br>otu_133237 otu_37625 otu_147731 otu_39381 otu_39039 otu_147520 otu_110785<br>otu_111821 otu_7471 otu_84990 otu_927 otu_39239 otu_85078 otu_37327 otu_38469<br>otu_38624 otu_128926 otu_113844 otu_7592 otu_103883 otu_155885 otu_7530<br>otu_103756 otu_8111 otu_115549 otu_38123 otu_39614 otu_155150 otu_38115<br>otu_111209 otu_84377 otu_44184 otu_84767 otu_85753 otu_113455 otu_7737 otu_8785<br>otu_37996 otu_85969 otu_110689 otu_41683 otu_111139 otu_42659 otu_134817<br>otu_100219 otu_100187 otu_155233 otu_142722 otu_84345 otu_84307 otu_37910<br>otu_147712 otu_155405 otu_114878 otu_41235 otu_137902 otu_84673 otu_112037<br>otu_7560 otu_7943 otu_111349 otu_114453 otu_41464 otu_155625 otu_104001<br>otu_128227 otu_110717 otu_39718 otu_155677 otu_147635 otu_114488 otu_7877<br>otu_115129 otu_110880 otu_85031 otu_112417 otu_104077 otu_155166 otu_144704<br>otu_114415 otu_8843 otu_155457 otu_7528 otu_128955 otu_85823 otu_85526 otu_111247<br>otu_128137 otu_103869 otu_133194 otu_111175 otu_37832 otu_111385 otu_134835<br>otu_110707 otu_84716 otu_155895 otu_84782 otu_7820 otu_37348 otu_156171<br>otu_112557 otu_38836 otu_42394 otu_7441 otu_137110 otu_85136 otu_7707 otu_147511<br>otu_112233 otu_84983 otu_111808 otu_111896 otu_7599 otu_38352 otu_84351 otu_85284<br>otu_85269 otu_7479 otu_110784 otu_38706 otu_114074 otu_37894 otu_7753 otu_39288<br>otu_103682 otu_136500 otu_111047 otu_7583 otu_923 otu_111998 otu_38374 otu_128608<br>otu_142801 otu_84534 otu_7839 otu_111036 otu_111801 otu_111726 otu_7947<br>otu_114170 otu_39731 otu_37587 otu_84320 otu_37519 otu_40892 otu_84634<br>otu_100305 otu_115110 otu_155628 otu_136490 otu_133216 otu_7438 otu_128620<br>otu_85001 otu_39871 otu_139264 otu_84598 otu_85365 otu_41004 otu_38728 otu_41651<br>otu_84298 otu_144700 otu_155180 otu_37243 otu_38074 otu_85562 otu_38973 otu_7872<br>otu_39945 otu_84340 otu_43237 otu_38029 otu_103653 otu_37364 otu_84601<br>otu_155010 otu_43623 otu_112672 otu_155299 otu_8045 otu_37835 otu_111842<br>otu_84571 otu_8658 otu_111970 otu_84374 otu_7638 otu_8438 otu_39274 otu_111425<br>otu_111538 otu_7475 otu_38404 otu_41984 otu_39357 otu_111714 otu_8057 otu_41318<br>otu_37370 otu_128919 otu_114762 otu_112415 otu_111374 otu_971 otu_8188 otu_110939<br>otu_84796 otu_37798 otu_113114 otu_7444 otu_103963 otu_155784 otu_154948<br>otu_114579 otu_128395 otu_38480 otu_114381 otu_85321 otu_138635 otu_85131<br>otu_37263 otu_128564 otu_147563 otu_39040 otu_40487 otu_40818 otu_40059<br>otu_111076 otu_111429 otu_142770 otu_113443 otu_41230 otu_38215 otu_115432<br>otu_7836 otu_128265 otu_41952 otu_40741 otu_128169 otu_84899 otu_112324<br>otu_37722 otu_112311 otu_139972 otu_112038 otu_39282 otu_142795 otu_7777<br>otu_38588 otu_128063 otu_110817 otu_139955 otu_155762 otu_155480 otu_37581<br>otu_100308 otu_144578 otu_114444 otu_43002 otu_42270 otu_111345 otu_110951<br>otu_156228 otu_42804 otu_129165 otu_155167 otu_155520 otu_110687 otu_84988<br>otu_39043 otu_38357 otu_111623 otu_110863 otu_103645 otu_39135 otu_111639 otu_201<br>otu_155025 otu_7860 otu_37547 otu_133211 otu_990 otu_40047 otu_38081 otu_111478<br>otu_110706 otu_110906 otu_1004 otu_155109 otu_40753 otu_147548 otu_156172<br>otu_38365 otu_155326 otu_39395 otu_111212 otu_113611 otu_112015 otu_37443<br>otu_155968 otu_84698 otu_155216 otu_155883 otu_110699 otu_7962 otu_110772<br>otu_113028 otu_112008 otu_100394 otu_112700 otu_155821 otu_44054 otu_103709<br>otu_155226 otu_142822 otu_147633 otu_155646 otu_110781 otu_7774 otu_147517<br>otu_85084 otu_155452 otu_112777 otu_42678 otu_84621 otu_113960 otu_112125<br>otu_111201 otu_128405 otu_139982 otu_7765 otu_38387 otu_112308 otu_128180<br>otu_128653 otu_37421 otu_42479 otu_111407 otu_144646 otu_39950 otu_141178<br>otu_38767 otu_37806 otu_84443 otu_40837 otu_147697 otu_111048 otu_139963 otu_7568<br>otu_7469 otu_155354 otu_97274 otu_134869 otu_155906 otu_111073 otu_155682<br>otu_100269 otu_103768 otu_155252 otu_41214 otu_113875 otu_156356 otu_147726<br>otu_8878 otu_142862 otu_112319 otu_112136 otu_129069 otu_7483 otu_147819<br>otu_41652 otu_38997 otu_38912 otu_41801 otu_41890 otu_128271 otu_154937<br>otu_85243 otu_38838 otu_155815 otu_8532 otu_141192 otu_100282 otu_111672 otu_8565<br>otu_154967 otu_85461 otu_7664 otu_40611 otu_155177 otu_38370 otu_114817 otu_7580<br>otu_114974 otu_38592 otu_133166 otu_111484 otu_154984 otu_42193 otu_38154<br>otu_84600 otu_100231 otu_113869 otu_38230 otu_155569 otu_85173 otu_155345<br>otu_38515 otu_38317 otu_111000 otu_110892 otu_155099 otu_137357 otu_144798<br>otu_111829 otu_38275 otu_7538 otu_38047 otu_37379 otu_972 otu_115008 otu_40293<br>otu_155139 otu_97306 otu_112942 otu_110851 otu_111960 otu_128116 otu_112652<br>otu_139940 otu_40157 otu_111138 otu_111863 otu_128961 otu_103793 otu_85482<br>otu_128982 otu_8490 otu_39882 otu_128440 otu_44006 otu_8958 otu_112381<br>otu_155045 otu_103851 otu_111849 otu_39127 otu_111225 otu_114487 otu_8154<br>otu_155259 otu_111402 otu_111018 otu_141169 otu_128173 otu_111215 otu_142782<br>otu_128472 otu_111761 otu_112739 otu_8273 otu_39215 otu_38520 otu_39149 otu_38049<br>otu_84415 otu_112291 otu_40257 otu_37375 otu_7552 otu_40166 otu_114654 otu_142738<br>otu_154971 otu_155500 otu_38220 otu_155693 otu_147713 otu_3 otu_139287 otu_38598<br>otu_37256 otu_155666 otu_38945 otu_147521 otu_42573 otu_85621 otu_97396<br>otu_84310 otu_144665 otu_41339 otu_139951 otu_163458 otu_86102 otu_111065<br>otu_84959 otu_7662 otu_112012 otu_7677 otu_37622 otu_41414 otu_111299 otu_128222<br>otu_39854 otu_38828 otu_8750 otu_111306 otu_40708 otu_141264 otu_142891 otu_7452<br>otu_112342 otu_85141 otu_42140 otu_142915 otu_111784 otu_37723 otu_141227<br>otu_129081 otu_40888 otu_147766 otu_38484 otu_155309 otu_164298 otu_128391<br>otu_111084 otu_155588
mpalaiv	393	otu_78411 otu_78081 otu_76917 otu_22265 otu_96374 otu_95601 otu_96094 otu_76646<br>otu_95808 otu_78660 otu_96444 otu_76762 otu_95745 otu_95703 otu_96225 otu_96007<br>otu_77415 otu_78154 otu_96207 otu_78118 otu_96076 otu_95587 otu_77132 otu_96230<br>otu_76745 otu_77867 otu_76692 otu_77961 otu_77213 otu_77929 otu_95676 otu_76710<br>otu_95887 otu_96099 otu_143763 otu_95933 otu_95635 otu_95842 otu_78121 otu_22305<br>otu_77493 otu_78579 otu_95725 otu_95768 otu_95987 otu_95674 otu_78280 otu_77851<br>otu_22268 otu_95876 otu_141780 otu_76862 otu_96081 otu_78663 otu_76931 otu_78182<br>otu_95660 otu_163752 otu_96574 otu_78702 otu_96299 otu_96250 otu_22259 otu_78102<br>[illegible]

		otu_129081 otu_40888 otu_147766 otu_38484 otu_155309 otu_164298 otu_128391 otu_111084 otu_155588
mpalaiv	393	otu_78411 otu_78081 otu_76917 otu_22265 otu_96374 otu_95601 otu_96094 otu_76646 otu_95808 otu_78660 otu_96444 otu_76762 otu_95745 otu_95703 otu_96225 otu_96007 otu_77415 otu_78154 otu_96207 otu_78118 otu_96076 otu_95587 otu_77132 otu_96230 otu_76745 otu_77867 otu_76692 otu_77961 otu_77213 otu_77929 otu_95676 otu_76710 otu_95887 otu_96099 otu_143763 otu_95933 otu_95635 otu_95842 otu_78121 otu_22305 otu_77493 otu_78579 otu_95725 otu_95768 otu_95987 otu_95674 otu_78280 otu_77851 otu_22268 otu_95876 otu_141780 otu_76862 otu_96081 otu_78663 otu_76931 otu_78182 otu_95660 otu_163752 otu_96574 otu_78702 otu_96299 otu_96250 otu_22259 otu_78102 otu_78023 otu_96025 otu_95623 otu_22276 otu_76769 otu_78239 otu_95734 otu_78559 otu_77941 otu_95792 otu_77109 otu_96086 otu_96436 otu_95841 otu_95763 otu_95736 otu_77203 otu_76882 otu_77700 otu_77121 otu_96361 otu_78625 otu_96323 otu_95602 otu_77089 otu_95848 otu_150050 otu_76900 otu_77381 otu_77091 otu_95702 otu_77013 otu_96167 otu_164201 otu_77994 otu_77031 otu_365 otu_96395 otu_95582 otu_22297 otu_96369 otu_96087 otu_95634 otu_78351 otu_96045 otu_96014 otu_95840 otu_76703 otu_76778 otu_76624 otu_95815 otu_96120 otu_96198 otu_78535 otu_140668 otu_96352 otu_95844 otu_77130 otu_77715 otu_77480 otu_76742 otu_77026 otu_77864 otu_96037 otu_96131 otu_95684 otu_77093 otu_95612 otu_76859 otu_78222 otu_96142 otu_22287 otu_76881 otu_361 otu_95764 otu_78866 otu_77075 otu_76704 otu_78044 otu_76752 otu_96166 otu_78256 otu_95814 otu_95847 otu_95904 otu_95675 otu_78007 otu_145996 otu_22261 otu_95818 otu_78013 otu_96065 otu_146016 otu_77134 otu_77810 otu_95780 otu_95633 otu_78422 otu_95618 otu_140667 otu_77117 otu_76890 otu_95958 otu_76795 otu_77542 otu_95559 otu_96114 otu_96331 otu_96519 otu_22281 otu_95836 otu_78224 otu_95704 otu_95560 otu_78858 otu_95710 otu_77107 otu_95700 otu_76807 otu_95790 otu_96208 otu_77096 otu_95550 otu_78339 otu_96069 otu_96383 otu_95627 otu_78614 otu_77841 otu_95965 otu_96305 otu_77285 otu_78034 otu_95650 otu_78386 otu_78251 otu_78542 otu_96172 otu_22277 otu_78367 otu_96138 otu_77400 otu_95639 otu_95624 otu_76903 otu_95729 otu_96000 otu_77273 otu_95791 otu_95689 otu_95983 otu_95781 otu_78139 otu_76773 otu_95827 otu_77224 otu_77554 otu_78456 otu_95597 otu_77410 otu_78111 otu_76765 otu_77068 otu_76670 otu_76962 otu_76998 otu_77278 otu_95737 otu_95648 otu_76991 otu_95903 otu_77855 otu_77503 otu_78516 otu_96396 otu_76983 otu_95845 otu_366 otu_96070 otu_77578 otu_95614 otu_95629 otu_77481 otu_96132 otu_95758 otu_95911 otu_95722 otu_77824 otu_95693 otu_76739 otu_96143 otu_76889 otu_77655 otu_95553 otu_95586 otu_95800 otu_96093 otu_76837 otu_95692 otu_96189 otu_95779 otu_77401 otu_76730 otu_96194 otu_95934 otu_96492 otu_95620 otu_95912 otu_95744 otu_96339 otu_95619 otu_77105 otu_96424 otu_77232 otu_95565 otu_95864 otu_95595 otu_95988 otu_95608 otu_96394 otu_95667 otu_96067 otu_95945 otu_95873 otu_78586 otu_96285 otu_77101 otu_22286 otu_95731 otu_95625 otu_96256 otu_95606 otu_96110 otu_77642 otu_163210 otu_76794 otu_95711 otu_77344 otu_77853 otu_77466 otu_95931 otu_95953 otu_163204 otu_95585 otu_96268 otu_95709 otu_76764 otu_95747 otu_95599 otu_78933 otu_77543 otu_76636 otu_76806 otu_77914 otu_96020 otu_77015 otu_95895 otu_95566 otu_77002 otu_95609 otu_95621 otu_95571 otu_78387 otu_96613 otu_77549 otu_95626 otu_77215 otu_96314 otu_95671 otu_77027 otu_96288 otu_95607 otu_78594 otu_78236 otu_77865 otu_95971 otu_78874 otu_95708 otu_78543 otu_95794 otu_78518 otu_95777 otu_96346 otu_95687 otu_76690 otu_76660 otu_78383 otu_96119 otu_96058 otu_96247 otu_77698 otu_78255 otu_78131 otu_78885 otu_95564 otu_95641 otu_95795 otu_77080 otu_77201 otu_95723 otu_77282 otu_77070 otu_96306 otu_96211 otu_95573 otu_77975 otu_78584 otu_96262 otu_96354 otu_96325 otu_77412 otu_95775 otu_76627 otu_78334 otu_95889 otu_95968 otu_76687 otu_77059 otu_95867 otu_96145 otu_78850 otu_95668 otu_77257 otu_96043 otu_95615 otu_77476 otu_76776 otu_95986 otu_76912
soils	11709	otu_1205 otu_64865 otu_157687 otu_1173 otu_91119 otu_149236 otu_87473 otu_65802 otu_65362 otu_16369 otu_9913 otu_16542 otu_20223 otu_13990 otu_52278 otu_62217 otu_115687 otu_140402 otu_102515 otu_52975 otu_121755 otu_91237 otu_119267 otu_11317 otu_141717 otu_87088 otu_88635 otu_90937 otu_145455 otu_92670 otu_98670 otu_46229 otu_138745 otu_87605 otu_100508 otu_14829 otu_118261 otu_116608 otu_131647 otu_65486 otu_11556 otu_158441 otu_87664 otu_159282 otu_50680 otu_19612 otu_19303 otu_119651 otu_116804 otu_54821 otu_140240 otu_9429 otu_162384 otu_120822 otu_19833 otu_122982 otu_19088 otu_58356 otu_51614 otu_64745 otu_62346 otu_58309 otu_120149 otu_47179 otu_52541 otu_158950 otu_161373 otu_1098 otu_19102 otu_66233 otu_50712 otu_45306 otu_131410 otu_67123 otu_58185 otu_116447 otu_88987 otu_46453 otu_59873 otu_15202 otu_119009 otu_16264 otu_135773 otu_59973 otu_88392 otu_162405 otu_100701 otu_86307 otu_60945 otu_1091 otu_1632 otu_58259 otu_88377 otu_118591 otu_1709 otu_130675 otu_89555 otu_47770 otu_12194 otu_157556 otu_12473 otu_86362 otu_53048 otu_139454 otu_117058 otu_57501 otu_60194 otu_160552 otu_47298 otu_11001 otu_45917 otu_10525 otu_101683 otu_91691 otu_119719 otu_13545 otu_138753 otu_58227 otu_140150 otu_120759 otu_19098 otu_87230 otu_101203 otu_93014 otu_118627 otu_149547 otu_91044 otu_1675 otu_148147 otu_94105 otu_134947 otu_87492 otu_101452 otu_101342 otu_95409 otu_9928 otu_157713 otu_17727 otu_18505 otu_1419 otu_58885 otu_161398 otu_57642 otu_10288 otu_9216 otu_148578 otu_104668 otu_63195 otu_101257 otu_52520 otu_122963 otu_55586 otu_131007 otu_144888 otu_148339 otu_131804 otu_50160 otu_130850 otu_45686 otu_115646 otu_115905 otu_87237 otu_87008 otu_54416 otu_88029 otu_59416 otu_117322 otu_1030 otu_117629 otu_101457 otu_158120 otu_87322 otu_47365 otu_54748 otu_46930 otu_47441 otu_145185 otu_9945 otu_86817 otu_61511 otu_123426 otu_141345 otu_162017 otu_129844 otu_9450 otu_159369 otu_143002 otu_120388 otu_100776 otu_93804 otu_105054 otu_48418 otu_86505 otu_58643 otu_16491 otu_20780 otu_48765 otu_45023 otu_129606 otu_14276 otu_54540 otu_122928 otu_90706 otu_134143 otu_16309 otu_57352 otu_101879 otu_56115 otu_16864 otu_160344 otu_50775 otu_61318 otu_100725 otu_46245 otu_17846 otu_121566 otu_62483 otu_134023 otu_115722 otu_163909 otu_143043 otu_54227 otu_98420 otu_116990 otu_86163 otu_160550 otu_47101 otu_105201 otu_104687 otu_87281 otu_143041 otu_160126 otu_10211 otu_16836 otu_116475 otu_131614 otu_47156 otu_149361 otu_61684 otu_161255 otu_116366 otu_159887 otu_159028 otu_64191 otu_140547 otu_17829 otu_46940 otu_50273 otu_102224 otu_46055 otu_51857 otu_115742 otu_130397 otu_61312 otu_88817 otu_93164 otu_135009 otu_60491 otu_44375 otu_52255 otu_87714 otu_53627 otu_89886 otu_50361 otu_117817 otu_138946 otu_93295 otu_10733 otu_65454 otu_100854 otu_118814 otu_47471 otu_58609 otu_88013 otu_149490 otu_100539 otu_129471 otu_9264 otu_159718 otu_158994 otu_149718 otu_58844 otu_1693 otu_141659 otu_46110 otu_157307 otu_148110 otu_137712 otu_158311 otu_9357 otu_12220 otu_64397 otu_100477 otu_45874 otu_46790 otu_118922 otu_59081 otu_136226 otu_116760 otu_45546 otu_159897 otu_90693 otu_129341 otu_16669 otu_11582 otu_17219 otu_1991 otu_117692 otu_119885 otu_120485 otu_104359 otu_49499 otu_157962 otu_86355 otu_91918 otu_48153 otu_116794 otu_10415 otu_49230 otu_99132 otu_14793 otu_2030 otu_120954 otu_60695 otu_55266 otu_91343 otu_57891 otu_47554 otu_1899 otu_133793 otu_148744 otu_52349 otu_133973 otu_86123 otu_9300 otu_60899 otu_45940 otu_57213 otu_116540 otu_104330 otu_47784 otu_16284 otu_57486 otu_136337 otu_48690 otu_9705 otu_1155 otu_149246 otu_90794 otu_87877 otu_102528 otu_18136 otu_55803 otu_18842 otu_17625 otu_47281 otu_9372 otu_149928 otu_91553 otu_92771 otu_157185 otu_10552 otu_145595 otu_98957 otu_122940 otu_62199 otu_21992 otu_64882 otu_16217 otu_48085 otu_102367 otu_101997 otu_118143 otu_16895 otu_44811 otu_10707 otu_134082 otu_53348 otu_61249 otu_90680 otu_47811 otu_20074 otu_104251 otu_13838 otu_102229 otu_48106 otu_120989 otu_17263 otu_139488 otu_63366 otu_64291 otu_61281 otu_18990 otu_138730 otu_120057 otu_140380 otu_44500 otu_13418 otu_148175 otu_120651 otu_52209 otu_50847 otu_44585

[illegible]
otu_120954 otu_60695 otu_55266 otu_91343 otu_57891 otu_47554 otu_1899 otu_133793
otu_148744 otu_52349 otu_133973 otu_86123 otu_9300 otu_60899 otu_45940 otu_57213
otu_116540 otu_104330 otu_47784 otu_16284 otu_57486 otu_136337 otu_48690
otu_9705 otu_1155 otu_149246 otu_90794 otu_87877 otu_102528 otu_18136 otu_55803
otu_18842 otu_17625 otu_47281 otu_9372 otu_149928 otu_91553 otu_92771 otu_157185
otu_10552 otu_145595 otu_98957 otu_122940 otu_62199 otu_21992 otu_64882
otu_16217 otu_48085 otu_102367 otu_101997 otu_118143 otu_16895 otu_44811
otu_10707 otu_134082 otu_53348 otu_61249 otu_90680 otu_47811 otu_20074
otu_104251 otu_13838 otu_102229 otu_48106 otu_120989 otu_17263 otu_139488
otu_63366 otu_64291 otu_61281 otu_18990 otu_138730 otu_120057 otu_140380
otu_44500 otu_13418 otu_148175 otu_120651 otu_52209 otu_50847 otu_44585
otu_123146 otu_145294 otu_115639 otu_160265 otu_92000 otu_86824 otu_11049
otu_52373 otu_157497 otu_45195 otu_119728 otu_61420 otu_64844 otu_86154
otu_61545 otu_116561 otu_64737 otu_54552 otu_121910 otu_56511 otu_66121
otu_143384 otu_53937 otu_138007 otu_129975 otu_48468 otu_138715 otu_21776
otu_50835 otu_95081 otu_160400 otu_16651 otu_59267 otu_63261 otu_20936 otu_91516
otu_94708 otu_90903 otu_119636 otu_47334 otu_14653 otu_44670 otu_91024 otu_115971
otu_119683 otu_64261 otu_160662 otu_139474 otu_10257 otu_86666 otu_86419
otu_10974 otu_87354 otu_143582 otu_86252 otu_45536 otu_161518 otu_91376
otu_62008 otu_50123 otu_55762 otu_105178 otu_14770 otu_59288 otu_134963
otu_62681 otu_9553 otu_15489 otu_161590 otu_145012 otu_148722 otu_86683
otu_100671 otu_149508 otu_119074 otu_133754 otu_162160 otu_130777 otu_138848
otu_119055 otu_20141 otu_47191 otu_123801 otu_16757 otu_145315 otu_59822
otu_145333 otu_138080 otu_47491 otu_160259 otu_20094 otu_159821 otu_97945
otu_87584 otu_119019 otu_49601 otu_104218 otu_65666 otu_11085 otu_157407
otu_11872 otu_86699 otu_119494 otu_16875 otu_53587 otu_49071 otu_140214 otu_13737
otu_101958 otu_139438 otu_115826 otu_60350 otu_293 otu_159612 otu_88188
otu_102087 otu_18854 otu_2089 otu_161137 otu_9227 otu_50635 otu_9634 otu_48125
otu_121089 otu_120371 otu_16537 otu_118136 otu_139406 otu_98150 otu_140319
otu_121649 otu_92958 otu_119211 otu_50037 otu_62668 otu_156542 otu_67716
otu_88571 otu_17408 otu_163884 otu_100579 otu_12881 otu_58178 otu_158451
otu_158212 otu_157144 otu_141586 otu_9435 otu_129283 otu_119513 otu_143505
otu_16844 otu_121129 otu_129945 otu_88901 otu_121157 otu_141393 otu_50960
otu_55249 otu_135072 otu_156639 otu_16740 otu_160040 otu_161959 otu_131407
otu_46817 otu_11865 otu_18540 otu_121534 otu_51584 otu_131909 otu_102749
otu_156759 otu_100683 otu_118190 otu_53194 otu_9869 otu_63957 otu_136905
otu_123831 otu_13230 otu_52591 otu_121598 otu_90614 otu_45235 otu_20448
otu_88440 otu_48335 otu_163904 otu_117130 otu_16565 otu_116399 otu_157613
otu_116957 otu_21750 otu_52167 otu_119876 otu_51020 otu_12045 otu_57541 otu_59486
otu_101914 otu_90641 otu_64566 otu_46808 otu_135532 otu_10504 otu_17536
otu_157031 otu_46877 otu_64928 otu_104383 otu_1904 otu_131197 otu_130725
otu_17895 otu_11425 otu_156968 otu_147981 otu_121251 otu_16502 otu_143039
otu_143454 otu_15269 otu_58385 otu_115832 otu_65423 otu_138724 otu_48592
otu_91589 otu_46188 otu_101075 otu_66367 otu_9888 otu_93266 otu_104777
otu_129772 otu_104255 otu_159709 otu_134943 otu_50700 otu_62098 otu_58502
otu_160023 otu_148082 otu_58727 otu_21840 otu_46591 otu_142996 otu_62091
otu_18869 otu_160447 otu_160370 otu_130638 otu_44744 otu_48265 otu_12225
otu_100978 otu_136269 otu_17744 otu_65606 otu_136687 otu_163541 otu_148138
otu_157367 otu_140300 otu_157378 otu_160218 otu_51800 otu_121343 otu_131274
otu_145339 otu_117533 otu_141275 otu_121723 otu_87863 otu_57693 otu_61321
otu_9536 otu_15373 otu_102381 otu_1217 otu_105044 otu_115949 otu_45527
otu_161072 otu_115847 otu_93117 otu_92169 otu_46091 otu_15827 otu_138690
otu_45103 otu_51835 otu_44706 otu_98396 otu_90777 otu_123661 otu_137953
otu_102619 otu_149131 otu_65181 otu_119459 otu_119153 otu_47418 otu_50540
otu_140391 otu_87002 otu_51610 otu_65842 otu_130664 otu_120479 otu_141564
otu_130302 otu_45628 otu_1461 otu_44629 otu_61866 otu_9420 otu_46314 otu_131136
otu_87069 otu_117210 otu_65004 otu_55615 otu_16313 otu_94114 otu_94082 otu_16533
otu_92748 otu_141567 otu_88310 otu_157382 otu_115694 otu_14414 otu_129869
otu_148364 otu_56885 otu_92098 otu_61260 otu_46973 otu_44460 otu_13917
otu_141553 otu_11140 otu_120709 otu_86704 otu_46063 otu_119390 otu_157337
otu_16584 otu_50287 otu_87228 otu_60911 otu_48023 otu_130200 otu_14609 otu_10630
otu_119017 otu_87257 otu_140178 otu_1221 otu_62813 otu_12794 otu_90815 otu_47596
otu_60890 otu_130126 otu_60215 otu_86190 otu_116677 otu_21354 otu_19900
otu_62848 otu_19431 otu_91741 otu_51877 otu_48202 otu_90775 otu_158100
otu_116318 otu_100484 otu_10743 otu_62131 otu_87218 otu_55021 otu_104736
otu_159360 otu_20245 otu_140106 otu_120045 otu_63714 otu_86740 otu_45343
otu_1033 otu_129261 otu_49638 otu_100669 otu_62881 otu_119465 otu_116348
otu_140081 otu_87668 otu_147938 otu_59203 otu_14369 otu_88054 otu_12568
otu_47267 otu_135672 otu_1745 otu_116845 otu_64681 otu_86729 otu_60889 otu_9684
otu_122030 otu_52810 otu_90769 otu_1884 otu_51184 otu_97554 otu_92518 otu_160129
otu_156538 otu_119814 otu_101282 otu_45421 otu_21362 otu_53927 otu_123175
otu_89961 otu_44812 otu_15018 otu_18918 otu_48463 otu_162211 otu_46959 otu_91561
otu_119688 otu_53763 otu_86989 otu_141300 otu_140183 otu_140474 otu_92364
otu_86692 otu_121056 otu_164090 otu_121040 otu_156825 otu_47894 otu_135350
otu_9489 otu_13542 otu_57806 otu_13071 otu_98511 otu_89757 otu_88935 otu_20716
otu_15735 otu_53665 otu_20536 otu_158160 otu_59451 otu_115939 otu_140156
otu_97654 otu_89855 otu_13993 otu_119337 otu_93350 otu_130655 otu_94037
otu_148406 otu_50999 otu_143535 otu_143620 otu_88872 otu_15496 otu_45987
otu_143005 otu_91798 otu_137211 otu_86184 otu_12935 otu_161874 otu_105266
otu_119603 otu_92877 otu_59854 otu_102257 otu_14880 otu_46865 otu_10184
otu_122792 otu_1570 otu_117932 otu_18056 otu_53283 otu_157066 otu_91094
otu_159755 otu_66953 otu_101436 otu_11613 otu_119424 otu_98475 otu_160742
otu_139317 otu_45145 otu_148946 otu_51662 otu_98655 otu_57681 otu_89345
otu_115794 otu_61852 otu_89512 otu_1694 otu_11666 otu_92084 otu_9749 otu_19411
otu_19140 otu_137116 otu_158333 otu_18140 otu_129818 otu_47015 otu_90951
otu_89892 otu_160156 otu_21410 otu_158418 otu_17117 otu_12553 otu_139401
otu_12688 otu_9697 otu_120366 otu_64161 otu_66147 otu_18227 otu_11568 otu_61540
otu_14247 otu_104718 otu_46620 otu_45019 otu_12345 otu_47004 otu_157662
otu_156587 otu_104970 otu_20801 otu_55467 otu_67505 otu_10313 otu_55483
otu_50058 otu_87148 otu_58995 otu_90869 otu_160171 otu_145009 otu_58854
otu_98169 otu_272 otu_138913 otu_64623 otu_121747 otu_13444 otu_148080
otu_160167 otu_161002 otu_104901 otu_11004 otu_9871 otu_57517 otu_156845
otu_100604 otu_16240 otu_1936 otu_59925 otu_119531 otu_93894 otu_149630
otu_162181 otu_13487 otu_20907 otu_61735 otu_138809 otu_10934 otu_138370
otu_122044 otu_148366 otu_12300 otu_9927 otu_148386 otu_51191 otu_141703
otu_130957 otu_49970 otu_12778 otu_131430 otu_147902 otu_12175 otu_137194
otu_161452 otu_143073 otu_62557 otu_59590 otu_1616 otu_121233 otu_59514
otu_16345 otu_147949 otu_122482 otu_45260 otu_90624 otu_51402 otu_118020
otu_10402 otu_52779 otu_116081 otu_120671 otu_12154 otu_161306 otu_66308
otu_87024 otu_58036 otu_53580 otu_131928 otu_57974 otu_45297 otu_95075 otu_9370
otu_52275 otu_58095 otu_133812 otu_117444 otu_45815 otu_18939 otu_94468
otu_137543 otu_48084 otu_49494 otu_162086 otu_158822 otu_16573 otu_137461
otu_53913 otu_101955 otu_50354 otu_157355 otu_10990 otu_91122 otu_46219
otu_20255 otu_143104 otu_137482 otu_160746 otu_49512 otu_130946 otu_58325
otu_149684 otu_92660 otu_91357 otu_90598 otu_157813 otu_52723 otu_100582
otu_105259 otu_16591 otu_161619 otu_104750 otu_9561 otu_60298 otu_58363
otu_101639 otu_93163 otu_49383 otu_46695 otu_104788 otu_137216 otu_65856
otu_137182 otu_143330 otu_10873 otu_161092 otu_14314 otu_116993 otu_17489
[illegible]

otu_16345 otu_147949 otu_122482 otu_45260 otu_90624 otu_51402 otu_118020
otu_10402 otu_52779 otu_116081 otu_120671 otu_12154 otu_161306 otu_66308
otu_87024 otu_58036 otu_53580 otu_131928 otu_57974 otu_45297 otu_95075 otu_9370
otu_52275 otu_58095 otu_133812 otu_117444 otu_45815 otu_18939 otu_94468
otu_137543 otu_48084 otu_49494 otu_162086 otu_158822 otu_16573 otu_137461
otu_53913 otu_101955 otu_50354 otu_157355 otu_10990 otu_91122 otu_46219
otu_20255 otu_143104 otu_137482 otu_160746 otu_49512 otu_130946 otu_58325
otu_149684 otu_92660 otu_91357 otu_90598 otu_157813 otu_52723 otu_100582
otu_105259 otu_16591 otu_161619 otu_104750 otu_9561 otu_60298 otu_58363
otu_101639 otu_93163 otu_49383 otu_46695 otu_104788 otu_137216 otu_65856
otu_137182 otu_143330 otu_10873 otu_161092 otu_14314 otu_116993 otu_17489
otu_120299 otu_16956 otu_120568 otu_157210 otu_87623 otu_97715 otu_118061
otu_90797 otu_119089 otu_100718 otu_63486 otu_100772 otu_140493 otu_148131
otu_91144 otu_45875 otu_16365 otu_13263 otu_116235 otu_121064 otu_92037 otu_13270
otu_87054 otu_148488 otu_129643 otu_104241 otu_130788 otu_45973 otu_9514
otu_129581 otu_10891 otu_122811 otu_156727 otu_12087 otu_163623 otu_119091
otu_54249 otu_159868 otu_19426 otu_59841 otu_59733 otu_65793 otu_135067
otu_46706 otu_133348 otu_9397 otu_101369 otu_92916 otu_45110 otu_139362
otu_45428 otu_55989 otu_46198 otu_60079 otu_140386 otu_137761 otu_61843
otu_60088 otu_67268 otu_60797 otu_16722 otu_63109 otu_163983 otu_13052
otu_121583 otu_141418 otu_101990 otu_50080 otu_46073 otu_143558 otu_57784
otu_100918 otu_14045 otu_12877 otu_59877 otu_148471 otu_45002 otu_93646
otu_57462 otu_129754 otu_149261 otu_57674 otu_45592 otu_93727 otu_9829 otu_17246
otu_52060 otu_147899 otu_104798 otu_45345 otu_129273 otu_116624 otu_97811
otu_87389 otu_98412 otu_86457 otu_144855 otu_56780 otu_59129 otu_60096 otu_18100
otu_131201 otu_90663 otu_90566 otu_163535 otu_158343 otu_87109 otu_160394
otu_44443 otu_149195 otu_1604 otu_9846 otu_157831 otu_60337 otu_86893 otu_148304
otu_88088 otu_17249 otu_10205 otu_129247 otu_52081 otu_61634 otu_16330
otu_131747 otu_135455 otu_50417 otu_11777 otu_159742 otu_9972 otu_9517
otu_120018 otu_47752 otu_21342 otu_13653 otu_160062 otu_119098 otu_58897
otu_116351 otu_16433 otu_163790 otu_104587 otu_91267 otu_62354 otu_47686
otu_102618 otu_133519 otu_13415 otu_11686 otu_9988 otu_89515 otu_119077
otu_148118 otu_89890 otu_98935 otu_140116 otu_94362 otu_87828 otu_149138
otu_122664 otu_11714 otu_119771 otu_104185 otu_159982 otu_1975 otu_88333 otu_1185
otu_1646 otu_136899 otu_145154 otu_130591 otu_53245 otu_163553 otu_156682
otu_16277 otu_19666 otu_102250 otu_91507 otu_104194 otu_135218 otu_60041
otu_120503 otu_44406 otu_57436 otu_118883 otu_9817 otu_16190 otu_49186 otu_47938
otu_90520 otu_137674 otu_10602 otu_161315 otu_116220 otu_62546 otu_86205
otu_137735 otu_9273 otu_90895 otu_118171 otu_9364 otu_12385 otu_63971 otu_130829
otu_87910 otu_67049 otu_98761 otu_140237 otu_162333 otu_9655 otu_115631
otu_57328 otu_48961 otu_50101 otu_122965 otu_59636 otu_61057 otu_50492
otu_161346 otu_157697 otu_98136 otu_1615 otu_143624 otu_138008 otu_20435
otu_1933 otu_129383 otu_122848 otu_86177 otu_45735 otu_102559 otu_131366
otu_61887 otu_11981 otu_63216 otu_115623 otu_52918 otu_137495 otu_11145 otu_11869
otu_15143 otu_129318 otu_48705 otu_93097 otu_116581 otu_148417 otu_130703
otu_161271 otu_86850 otu_122384 otu_50731 otu_45071 otu_9979 otu_140253
otu_138704 otu_147853 otu_15014 otu_88053 otu_138886 otu_48080 otu_48403
otu_156798 otu_91885 otu_16249 otu_138775 otu_10407 otu_54479 otu_140260
otu_129938 otu_137171 otu_86156 otu_120818 otu_148070 otu_159568 otu_62190
otu_104159 otu_149273 otu_54733 otu_20276 otu_65 otu_59333 otu_93611 otu_123178
otu_20284 otu_17397 otu_149271 otu_129305 otu_20673 otu_17448 otu_58175
otu_58086 otu_57638 otu_131980 otu_148361 otu_118302 otu_118697 otu_148378
otu_86759 otu_50797 otu_97512 otu_58630 otu_63372 otu_138734 otu_138695
otu_44856 otu_18078 otu_141575 otu_118753 otu_1226 otu_48028 otu_58937
otu_121635 otu_9689 otu_45631 otu_51033 otu_104906 otu_20998 otu_18518 otu_59119
otu_119591 otu_101029 otu_100902 otu_11480 otu_45847 otu_57158 otu_55018
otu_19123 otu_143242 otu_62808 otu_104803 otu_158299 otu_143633 otu_118934
otu_138785 otu_17775 otu_16205 otu_12971 otu_44501 otu_129455 otu_100525
otu_46224 otu_9905 otu_19523 otu_104248 otu_159408 otu_14855 otu_44889
otu_135688 otu_16576 otu_100654 otu_45799 otu_100787 otu_45101 otu_117543
otu_58534 otu_159171 otu_91868 otu_143101 otu_120488 otu_51406 otu_123634
otu_149183 otu_46680 otu_57437 otu_61001 otu_118340 otu_15423 otu_158155
otu_102147 otu_117822 otu_117396 otu_17229 otu_94740 otu_14105 otu_90837
otu_98588 otu_9342 otu_19388 otu_93398 otu_17101 otu_119785 otu_158635
otu_131816 otu_91733 otu_104321 otu_129400 otu_92506 otu_141339 otu_138674
otu_149297 otu_62194 otu_58211 otu_92317 otu_119085 otu_52756 otu_46153 otu_45278
otu_121520 otu_91385 otu_105278 otu_46366 otu_90398 otu_144861 otu_143337
otu_156700 otu_158053 otu_135233 otu_105188 otu_52467 otu_105062 otu_17378
otu_158516 otu_118852 otu_102125 otu_13248 otu_50828 otu_58470 otu_149507
otu_129334 otu_158222 otu_20821 otu_20206 otu_56144 otu_160219 otu_66 otu_116996
otu_57625 otu_158765 otu_149457 otu_10360 otu_87771 otu_53432 otu_50180
otu_88882 otu_86207 otu_129767 otu_157923 otu_118720 otu_18667 otu_140453
otu_58295 otu_101549 otu_97825 otu_48546 otu_58459 otu_45426 otu_54691
otu_156683 otu_104848 otu_9937 otu_1567 otu_141615 otu_54564 otu_144897
otu_92109 otu_46569 otu_16222 otu_54846 otu_129358 otu_47233 otu_101350
otu_91128 otu_12504 otu_120600 otu_61243 otu_149424 otu_92654 otu_90762
otu_10127 otu_59054 otu_53719 otu_13310 otu_149242 otu_88349 otu_117298
otu_160190 otu_61826 otu_51095 otu_49386 otu_163927 otu_49759 otu_15368
otu_143095 otu_119018 otu_148116 otu_98370 otu_58870 otu_159122 otu_91273
otu_46642 otu_44265 otu_118688 otu_49979 otu_162186 otu_98406 otu_136830
otu_44826 otu_94744 otu_91440 otu_158072 otu_104402 otu_12743 otu_139446
otu_18766 otu_44463 otu_17717 otu_14930 otu_138716 otu_120216 otu_160549
otu_45357 otu_92168 otu_135791 otu_47419 otu_86510 otu_157730 otu_88360
otu_11270 otu_137758 otu_21545 otu_148268 otu_88841 otu_91155 otu_132070 otu_9
otu_116238 otu_140602 otu_116119 otu_133527 otu_57423 otu_158165 otu_18331
otu_100666 otu_10063 otu_9711 otu_86495 otu_46711 otu_51421 otu_45217 otu_104165
otu_19450 otu_163515 otu_16989 otu_116695 otu_9566 otu_62246 otu_87107
otu_105257 otu_10143 otu_87965 otu_115697 otu_58951 otu_89317 otu_160946
otu_143375 otu_159144 otu_47137 otu_149288 otu_116213 otu_120350 otu_52838
otu_62411 otu_46271 otu_118867 otu_117795 otu_157419 otu_58751 otu_51328
otu_49889 otu_158966 otu_11075 otu_11486 otu_59063 otu_149044 otu_119080
otu_101225 otu_132054 otu_20872 otu_123700 otu_90798 otu_130250 otu_120710
otu_11756 otu_18438 otu_89805 otu_19901 otu_67144 otu_92610 otu_58441 otu_44439
otu_98526 otu_9734 otu_87212 otu_50425 otu_58061 otu_164346 otu_98145 otu_130138
otu_120518 otu_44260 otu_161313 otu_88548 otu_116508 otu_10789 otu_60318
otu_61055 otu_97771 otu_133485 otu_65106 otu_131739 otu_91513 otu_130796
otu_140126 otu_129490 otu_148064 otu_86325 otu_142988 otu_1335 otu_130914
otu_17083 otu_11766 otu_68520 otu_95007 otu_58919 otu_65527 otu_10434 otu_160262
otu_48453 otu_100711 otu_49254 otu_86367 otu_52270 otu_18182 otu_87554
otu_143385 otu_16236 otu_57235 otu_10305 otu_160070 otu_119385 otu_129817
otu_1181 otu_48019 otu_94606 otu_48221 otu_86720 otu_145482 otu_20699 otu_89196
otu_100810 otu_55988 otu_102684 otu_119659 otu_60663 otu_90977 otu_55402
otu_61902 otu_90363 otu_145293 otu_13169 otu_45976 otu_14630 otu_92094 otu_98478
otu_143357 otu_13687 otu_89642 otu_68518 otu_46948 otu_159047 otu_16244
otu_100489 otu_118790 otu_131211 otu_156822 otu_118239 otu_102235 otu_129264
otu_13563 otu_129223 otu_159752 otu_147978 otu_59662 otu_46846 otu_45431
otu_139441 otu_12262 otu_56859 otu_162463 otu_16889 otu_118911 otu_61764
otu_90103 otu_117753 otu_18731 otu_116604 otu_17960 otu_87504 otu_44759
otu_147933 otu_147863 otu_86225 otu_10899 otu_13975 otu_116292 otu_88881

[illegible]
otu_48453 otu_100711 otu_49254 otu_86367 otu_52270 otu_18182 otu_87554
otu_143385 otu_16236 otu_57235 otu_10305 otu_160070 otu_119385 otu_129817
otu_1181 otu_48019 otu_94606 otu_48221 otu_86720 otu_145482 otu_20699 otu_89196
otu_100810 otu_55988 otu_102684 otu_119659 otu_60663 otu_90977 otu_55402
otu_61902 otu_90363 otu_145293 otu_13169 otu_45976 otu_14630 otu_92094 otu_98478
otu_143357 otu_13687 otu_89642 otu_68518 otu_46948 otu_159047 otu_16244
otu_100489 otu_118790 otu_131211 otu_156822 otu_118239 otu_102235 otu_129264
otu_13563 otu_129223 otu_159752 otu_147978 otu_59662 otu_46846 otu_45431
otu_139441 otu_12262 otu_56859 otu_162463 otu_16889 otu_118911 otu_61764
otu_90103 otu_117753 otu_18731 otu_116604 otu_17960 otu_87504 otu_44759
otu_147933 otu_147863 otu_86225 otu_10899 otu_13975 otu_116292 otu_88881
otu_97954 otu_86299 otu_156638 otu_138092 otu_47090 otu_91053 otu_115660
otu_101909 otu_119974 otu_1188 otu_115786 otu_159894 otu_136906 otu_62321
otu_63113 otu_101876 otu_101165 otu_149311 otu_143076 otu_131682 otu_90580
otu_102088 otu_48215 otu_138362 otu_16686 otu_119024 otu_130642 otu_90803
otu_149252 otu_17516 otu_55584 otu_9866 otu_16552 otu_140107 otu_97907 otu_55730
otu_101185 otu_140146 otu_94315 otu_160378 otu_90139 otu_61834 otu_62676
otu_148652 otu_159252 otu_18695 otu_16180 otu_145530 otu_133936 otu_130719
otu_13529 otu_19425 otu_156616 otu_137512 otu_115963 otu_50074 otu_48233
otu_86912 otu_12001 otu_133924 otu_161800 otu_118430 otu_20649 otu_59994
otu_157018 otu_13148 otu_15232 otu_160457 otu_159748 otu_61676 otu_160113
otu_138374 otu_161601 otu_13476 otu_11911 otu_58514 otu_136997 otu_131166
otu_53519 otu_46390 otu_89638 otu_122298 otu_157501 otu_16406 otu_18742
otu_59087 otu_100861 otu_52244 otu_49082 otu_60138 otu_148030 otu_102275
otu_97583 otu_1132 otu_58272 otu_61297 otu_15139 otu_59396 otu_86138 otu_50792
otu_138334 otu_17527 otu_20544 otu_87758 otu_15746 otu_161114 otu_13852 otu_21549
otu_116871 otu_104764 otu_118646 otu_47937 otu_14866 otu_21848 otu_48848
otu_57770 otu_44469 otu_60983 otu_121862 otu_93165 otu_134915 otu_119668
otu_54375 otu_61860 otu_98540 otu_50554 otu_17076 otu_12572 otu_118636
otu_121527 otu_44471 otu_116489 otu_51058 otu_10422 otu_87297 otu_161065
otu_115878 otu_160738 otu_65097 otu_44601 otu_97451 otu_15939 otu_1463
otu_138772 otu_115594 otu_115750 otu_143136 otu_160354 otu_19182 otu_62771
otu_119766 otu_91318 otu_63072 otu_57531 otu_119356 otu_50768 otu_89177
otu_140527 otu_148217 otu_58048 otu_141302 otu_44621 otu_18550 otu_17787
otu_162139 otu_91192 otu_147870 otu_143016 otu_120013 otu_62402 otu_14589
otu_136799 otu_87028 otu_100462 otu_58837 otu_140403 otu_86878 otu_90809
otu_92112 otu_148486 otu_64487 otu_132183 otu_12307 otu_117325 otu_137181
otu_10721 otu_11921 otu_141316 otu_67018 otu_93616 otu_129522 otu_134889
otu_9363 otu_129505 otu_45904 otu_44563 otu_62626 otu_58464 otu_13479 otu_53617
otu_67914 otu_89009 otu_89695 otu_115775 otu_63239 otu_145522 otu_145171
otu_60778 otu_130388 otu_158233 otu_91058 otu_63789 otu_47666 otu_140119
otu_120466 otu_86959 otu_45887 otu_160481 otu_59698 otu_138737 otu_87568
otu_116029 otu_10106 otu_159660 otu_159618 otu_101947 otu_117729 otu_9233
otu_137754 otu_46221 otu_156925 otu_101354 otu_21333 otu_46490 otu_1703
otu_90553 otu_139326 otu_98654 otu_86395 otu_1743 otu_16954 otu_64269 otu_45971
otu_117686 otu_116039 otu_59815 otu_131022 otu_100805 otu_45155 otu_54115
otu_157426 otu_120307 otu_121469 otu_55108 otu_11341 otu_98464 otu_91925
otu_129835 otu_137459 otu_67061 otu_120581 otu_92491 otu_115988 otu_11498
otu_18719 otu_13749 otu_149914 otu_94243 otu_122867 otu_65023 otu_54386
otu_46445 otu_21366 otu_91625 otu_120673 otu_9337 otu_57965 otu_16819 otu_19310
otu_130082 otu_62306 otu_87687 otu_14686 otu_17928 otu_45011 otu_138701
otu_10037 otu_12025 otu_55602 otu_141447 otu_20645 otu_11571 otu_149194
otu_120555 otu_64997 otu_45420 otu_156581 otu_156561 otu_118196 otu_46300
otu_145451 otu_130688 otu_141292 otu_9795 otu_61443 otu_45117 otu_100742
otu_44544 otu_1762 otu_13049 otu_119196 otu_46328 otu_87520 otu_102148
otu_159315 otu_92767 otu_62216 otu_13154 otu_51665 otu_119149 otu_129403
otu_159673 otu_45955 otu_64785 otu_21513 otu_51869 otu_89200 otu_156751
otu_148148 otu_148914 otu_1938 otu_63687 otu_60798 otu_51174 otu_17985 otu_161121
otu_158241 otu_87444 otu_158816 otu_136626 otu_57633 otu_137026 otu_101493
otu_87401 otu_19659 otu_162196 otu_143649 otu_87427 otu_13883 otu_12050
otu_87029 otu_9505 otu_158340 otu_131264 otu_49867 otu_157089 otu_129398
otu_48036 otu_63279 otu_140086 otu_161533 otu_48120 otu_58072 otu_119413
otu_87954 otu_143398 otu_116519 otu_119483 otu_50581 otu_94543 otu_88886
otu_141619 otu_133451 otu_129979 otu_45705 otu_148041 otu_101673 otu_47184
otu_121779 otu_140591 otu_57415 otu_89531 otu_86535 otu_130901 otu_156771
otu_129229 otu_116685 otu_61573 otu_60163 otu_159888 otu_119274 otu_65076
otu_13436 otu_157950 otu_104221 otu_92068 otu_59617 otu_45022 otu_160048
otu_63907 otu_13784 otu_57400 otu_163918 otu_159136 otu_60575 otu_58428
otu_18286 otu_12840 otu_91093 otu_45746 otu_91274 otu_117628 otu_102174
otu_92212 otu_91163 otu_47299 otu_15353 otu_121807 otu_121592 otu_157600
otu_9378 otu_158405 otu_90871 otu_65549 otu_66389 otu_56169 otu_90752 otu_59373
otu_101906 otu_93896 otu_89470 otu_17357 otu_101217 otu_98907 otu_158854
otu_16545 otu_117588 otu_45007 otu_46960 otu_11432 otu_52035 otu_86126 otu_12044
otu_57790 otu_119539 otu_98603 otu_143312 otu_156817 otu_44880 otu_18145
otu_52785 otu_44736 otu_90726 otu_116091 otu_90549 otu_1066 otu_60551 otu_159799
otu_9938 otu_160890 otu_97640 otu_46450 otu_19915 otu_54911 otu_131127 otu_2050
otu_157932 otu_149460 otu_121308 otu_141552 otu_129347 otu_161797 otu_157965
otu_14080 otu_130120 otu_122205 otu_102187 otu_130044 otu_21830 otu_49371
otu_145087 otu_17070 otu_118613 otu_143020 otu_148039 otu_119995 otu_135650
otu_136002 otu_59105 otu_119078 otu_117254 otu_164032 otu_97537 otu_157714
otu_59982 otu_157469 otu_59859 otu_101881 otu_141545 otu_135844 otu_164068
otu_161822 otu_92177 otu_143175 otu_17349 otu_87233 otu_21685 otu_145155
otu_53195 otu_54977 otu_160949 otu_121279 otu_92002 otu_58014 otu_137938
otu_59068 otu_92150 otu_115573 otu_92822 otu_130977 otu_86391 otu_121408
otu_16769 otu_88776 otu_138942 otu_1363 otu_156641 otu_44780 otu_93807 otu_48702
otu_140560 otu_15919 otu_88798 otu_14986 otu_9847 otu_138042 otu_2082 otu_19898
otu_122238 otu_143509 otu_159874 otu_92603 otu_48666 otu_156704 otu_115871
otu_44758 otu_159536 otu_53307 otu_12110 otu_20439 otu_104333 otu_100655
otu_121047 otu_86584 otu_86250 otu_98876 otu_87372 otu_278 otu_147913 otu_98961
otu_119068 otu_61083 otu_9666 otu_131628 otu_87072 otu_14153 otu_143048
otu_50823 otu_65990 otu_50860 otu_86166 otu_160413 otu_101478 otu_87734
otu_100877 otu_57647 otu_20853 otu_104749 otu_149148 otu_50867 otu_90654
otu_91840 otu_118819 otu_159841 otu_117476 otu_162346 otu_58374 otu_46568
otu_161576 otu_61890 otu_157737 otu_55579 otu_160137 otu_137711 otu_50364
otu_57323 otu_141612 otu_123156 otu_143092 otu_102709 otu_14959 otu_157229
otu_116881 otu_159737 otu_10196 otu_140483 otu_63859 otu_14906 otu_17173
otu_149375 otu_55655 otu_88918 otu_90415 otu_18365 otu_115822 otu_58685
otu_87545 otu_118618 otu_86587 otu_90590 otu_46614 otu_67612 otu_16333 otu_9478
otu_140408 otu_101991 otu_141557 otu_50191 otu_91603 otu_1438 otu_20116
otu_66750 otu_145144 otu_136314 otu_44371 otu_104277 otu_130036 otu_92510
otu_116816 otu_9729 otu_58082 otu_48888 otu_14281 otu_1178 otu_51573 otu_148055
otu_53018 otu_14798 otu_95031 otu_50257 otu_134902 otu_147864 otu_9321
otu_130672 otu_135736 otu_17716 otu_91382 otu_10492 otu_48136 otu_18626 otu_1289
otu_119446 otu_159788 otu_148184 otu_87561 otu_116843 otu_58771 otu_18115
otu_86507 otu_44692 otu_119971 otu_138708 otu_157176 otu_44540 otu_101528
otu_129974 otu_61359 otu_54360 otu_60750 otu_10638 otu_10110 otu_93645 otu_45208
otu_57826 otu_9839 otu_134113 otu_18900 otu_58865 otu_48514 otu_9453 otu_119917
otu_116664 otu_19481 otu_16946 otu_45429 otu_45610 otu_148731 otu_136603
[illegible]

otu_87545 otu_118618 otu_86587 otu_90590 otu_46614 otu_67612 otu_16333 otu_9478
otu_140408 otu_101991 otu_141557 otu_50191 otu_91603 otu_1438 otu_20116
otu_66750 otu_145144 otu_136314 otu_44371 otu_104277 otu_130036 otu_92510
otu_116816 otu_9729 otu_58082 otu_48888 otu_14281 otu_1178 otu_51573 otu_148055
otu_53018 otu_14798 otu_95031 otu_50257 otu_134902 otu_147864 otu_9321
otu_130672 otu_135736 otu_17716 otu_91382 otu_10492 otu_48136 otu_18626 otu_1289
otu_119446 otu_159788 otu_148184 otu_87561 otu_116843 otu_58771 otu_18115
otu_86507 otu_44692 otu_119971 otu_138708 otu_157176 otu_44540 otu_101528
otu_129974 otu_61359 otu_54360 otu_60750 otu_10638 otu_10110 otu_93645 otu_45208
otu_57826 otu_9839 otu_134113 otu_18900 otu_58865 otu_48514 otu_9453 otu_119917
otu_116664 otu_19481 otu_16946 otu_45429 otu_45610 otu_148731 otu_136603
otu_129495 otu_59224 otu_61835 otu_87922 otu_11789 otu_148003 otu_135962
otu_129244 otu_117068 otu_149797 otu_1852 otu_59459 otu_158375 otu_123088
otu_104827 otu_145417 otu_19079 otu_119745 otu_159462 otu_45892 otu_57134
otu_92207 otu_86482 otu_20046 otu_159412 otu_12542 otu_91626 otu_14184 otu_55464
otu_60399 otu_67965 otu_44411 otu_52340 otu_87493 otu_86667 otu_47056 otu_93586
otu_11314 otu_116985 otu_90934 otu_101664 otu_58581 otu_1964 otu_142983
otu_140374 otu_117141 otu_101956 otu_54878 otu_101500 otu_59295 otu_86246
otu_138905 otu_90833 otu_135679 otu_61925 otu_136348 otu_58404 otu_156923
otu_145481 otu_138369 otu_60762 otu_117127 otu_130863 otu_48266 otu_88024
otu_138982 otu_57481 otu_116170 otu_53775 otu_10222 otu_102195 otu_16742
otu_159966 otu_61744 otu_65674 otu_105212 otu_94027 otu_57062 otu_88698
otu_148012 otu_48132 otu_18952 otu_18183 otu_19407 otu_137551 otu_49932
otu_143276 otu_138726 otu_130297 otu_46891 otu_60833 otu_55277 otu_87551
otu_143416 otu_11753 otu_130736 otu_16876 otu_119158 otu_90345 otu_1752 otu_57422
otu_93924 otu_133403 otu_119943 otu_148822 otu_16683 otu_58901 otu_129810
otu_49158 otu_58286 otu_156651 otu_163882 otu_88112 otu_138039 otu_9987
otu_147984 otu_48345 otu_44262 otu_120087 otu_141593 otu_138952 otu_138870
otu_59479 otu_46776 otu_163661 otu_138813 otu_66603 otu_129367 otu_16960
otu_58638 otu_47859 otu_157124 otu_90573 otu_54105 otu_122461 otu_87533
otu_45935 otu_97798 otu_160602 otu_67882 otu_137546 otu_46822 otu_86829
otu_55885 otu_12622 otu_64314 otu_143697 otu_119458 otu_157491 otu_86977
otu_104941 otu_10515 otu_59433 otu_66134 otu_116004 otu_91908 otu_115671
otu_16957 otu_55697 otu_51099 otu_140316 otu_55156 otu_157619 otu_129930
otu_133358 otu_130790 otu_159911 otu_115895 otu_136568 otu_129644 otu_138916
otu_45164 otu_17562 otu_101903 otu_104841 otu_57882 otu_98253 otu_92593
otu_60742 otu_59752 otu_157734 otu_44422 otu_116480 otu_148073 otu_55376
otu_59032 otu_100476 otu_10736 otu_148833 otu_86807 otu_10373 otu_57377
otu_116587 otu_102358 otu_158109 otu_59568 otu_9870 otu_159950 otu_121737
otu_116948 otu_159726 otu_44381 otu_46623 otu_140463 otu_98405 otu_157741
otu_140268 otu_116166 otu_160444 otu_159060 otu_48334 otu_52768 otu_10312
otu_9406 otu_120879 otu_1849 otu_60010 otu_156848 otu_139425 otu_160751
otu_116830 otu_15406 otu_44846 otu_45304 otu_89010 otu_90969 otu_93298 otu_50902
otu_135444 otu_130808 otu_51022 otu_44821 otu_59260 otu_10268 otu_47787
otu_118801 otu_121346 otu_18371 otu_64322 otu_67499 otu_63734 otu_87144
otu_46059 otu_157853 otu_94279 otu_17768 otu_158192 otu_92418 otu_104761
otu_13065 otu_10621 otu_17737 otu_119854 otu_15149 otu_55926 otu_50329 otu_116952
otu_140185 otu_64640 otu_88517 otu_14270 otu_141638 otu_97497 otu_56646
otu_163577 otu_91411 otu_64505 otu_119431 otu_17421 otu_91117 otu_66392 otu_9926
otu_91641 otu_86860 otu_88017 otu_15340 otu_119359 otu_161233 otu_12026
otu_129580 otu_157404 otu_91663 otu_49120 otu_44342 otu_129466 otu_89358
otu_45528 otu_161168 otu_148244 otu_57892 otu_47271 otu_66543 otu_46263
otu_52973 otu_145058 otu_62983 otu_45626 otu_46248 otu_54097 otu_88168
otu_133373 otu_15254 otu_240 otu_19178 otu_100836 otu_14923 otu_140136 otu_67423
otu_160924 otu_135099 otu_46331 otu_115816 otu_58378 otu_88959 otu_1322
otu_90645 otu_162806 otu_97669 otu_149780 otu_10341 otu_45729 otu_119758
otu_65286 otu_118773 otu_92138 otu_131312 otu_59463 otu_98728 otu_129650
otu_131085 otu_148040 otu_19190 otu_137508 otu_17606 otu_118118 otu_129840
otu_18015 otu_17220 otu_1708 otu_66728 otu_100984 otu_60272 otu_17415 otu_97728
otu_19673 otu_119829 otu_140473 otu_161559 otu_88531 otu_45329 otu_58842
otu_53746 otu_135257 otu_62337 otu_120552 otu_61738 otu_11172 otu_122083
otu_90770 otu_9374 otu_87894 otu_141565 otu_136903 otu_101859 otu_158528
otu_55193 otu_117215 otu_149250 otu_64732 otu_88857 otu_51980 otu_120414
otu_118764 otu_89554 otu_160053 otu_135675 otu_87710 otu_48422 otu_45701
otu_89758 otu_116283 otu_156546 otu_161238 otu_17951 otu_123013 otu_141315
otu_87934 otu_61282 otu_13880 otu_156959 otu_102222 otu_16990 otu_97732
otu_14795 otu_17586 otu_64280 otu_52833 otu_9444 otu_160177 otu_94085 otu_143354
otu_20248 otu_17452 otu_12425 otu_130555 otu_87186 otu_48948 otu_101894
otu_52099 otu_9898 otu_16679 otu_10001 otu_98443 otu_100480 otu_149224 otu_45389
otu_86958 otu_119808 otu_120093 otu_46326 otu_134918 otu_50118 otu_62477
otu_14194 otu_138668 otu_54357 otu_10797 otu_131549 otu_86765 otu_162202
otu_143523 otu_100636 otu_18651 otu_59603 otu_89140 otu_11935 otu_145520
otu_90751 otu_88266 otu_52351 otu_53213 otu_134054 otu_116734 otu_18589
otu_91631 otu_90098 otu_16187 otu_13629 otu_20951 otu_56253 otu_149235
otu_135841 otu_137769 otu_98483 otu_137204 otu_54817 otu_44223 otu_105004
otu_147967 otu_61460 otu_138067 otu_138683 otu_131354 otu_58269 otu_139338
otu_10875 otu_52347 otu_61557 otu_143393 otu_91673 otu_160407 otu_160511
otu_86752 otu_104939 otu_16368 otu_44306 otu_14826 otu_87243 otu_91289 otu_16463
otu_135518 otu_48031 otu_46093 otu_10094 otu_129504 otu_87756 otu_86769
otu_14235 otu_48985 otu_156979 otu_122284 otu_20042 otu_160660 otu_10351
otu_67055 otu_53143 otu_46598 otu_164319 otu_300 otu_13690 otu_164112 otu_65699
otu_54812 otu_117087 otu_87686 otu_62611 otu_135550 otu_116427 otu_98634
otu_57911 otu_90511 otu_61365 otu_62888 otu_20187 otu_86664 otu_136296 otu_101432
otu_17144 otu_46031 otu_14441 otu_160271 otu_49715 otu_163514 otu_47160
otu_115950 otu_16519 otu_145096 otu_58440 otu_119199 otu_61595 otu_130056
otu_58314 otu_133503 otu_121750 otu_119783 otu_115657 otu_148346 otu_60277
otu_16654 otu_88711 otu_51070 otu_11346 otu_158565 otu_93106 otu_140382
otu_119173 otu_17564 otu_115969 otu_159981 otu_133926 otu_44286 otu_9982
otu_53274 otu_135171 otu_143530 otu_59042 otu_11896 otu_120699 otu_86218
otu_102161 otu_13168 otu_157504 otu_2008 otu_49303 otu_140118 otu_10799 otu_1326
otu_160064 otu_119953 otu_46998 otu_163967 otu_141580 otu_10834 otu_98440
otu_17261 otu_47541 otu_18209 otu_121717 otu_13388 otu_160907 otu_160277
otu_11384 otu_157029 otu_48426 otu_52380 otu_18730 otu_65860 otu_131773
otu_121300 otu_123455 otu_13874 otu_121082 otu_14507 otu_61918 otu_58480
otu_133767 otu_158754 otu_88573 otu_140335 otu_97522 otu_143035 otu_102172
otu_62140 otu_61139 otu_148016 otu_54703 otu_117579 otu_14190 otu_10669
otu_130940 otu_102025 otu_46870 otu_136535 otu_157005 otu_87505 otu_87424
otu_94644 otu_16720 otu_92545 otu_51896 otu_104589 otu_119647 otu_129947
otu_19275 otu_65283 otu_158697 otu_91224 otu_90827 otu_141440 otu_13364 otu_1819
otu_129954 otu_92026 otu_11460 otu_19418 otu_122626 otu_140312 otu_45112
otu_17241 otu_10667 otu_45412 otu_59525 otu_9399 otu_9334 otu_92342 otu_140368
otu_12007 otu_100673 otu_57817 otu_45059 otu_160790 otu_62466 otu_55073
otu_60795 otu_47614 otu_158908 otu_100621 otu_59975 otu_50292 otu_58737 otu_9433
otu_12678 otu_61755 otu_10107 otu_163560 otu_9636 otu_45595 otu_16243 otu_9954
otu_160266 otu_45277 otu_100985 otu_135232 otu_104192 otu_46739 otu_91651
otu_159014 otu_162035 otu_158092 otu_53620 otu_62096 otu_135200 otu_86702
otu_161770 otu_58349 otu_61892 otu_66804 otu_20618 otu_86949 otu_156911
otu_86598 otu_86271 otu_47360 otu_12174 otu_16400 otu_18169 otu_63270 otu_15151
otu_9275 otu_119297 otu_53118 otu_46781 otu_116316 otu_133492 otu_93154 otu_20647

otu_94644 otu_16720 otu_92545 otu_51896 otu_104589 otu_119647 otu_129947
otu_19275 otu_65283 otu_158697 otu_91224 otu_90827 otu_141440 otu_13364 otu_1819
otu_129954 otu_92026 otu_11460 otu_19418 otu_122626 otu_140312 otu_45112
otu_17241 otu_10667 otu_45412 otu_59525 otu_9399 otu_9334 otu_92342 otu_140368
otu_12007 otu_100673 otu_57817 otu_45059 otu_160790 otu_62466 otu_55073
otu_60795 otu_47614 otu_158908 otu_100621 otu_59975 otu_50292 otu_58737 otu_9433
otu_12678 otu_61755 otu_10107 otu_163560 otu_9636 otu_45595 otu_16243 otu_9954
otu_160266 otu_45277 otu_100985 otu_135232 otu_104192 otu_46739 otu_91651
otu_159014 otu_162035 otu_158092 otu_53620 otu_62096 otu_135200 otu_86702
otu_161770 otu_58349 otu_61892 otu_66804 otu_20618 otu_86949 otu_156911
otu_86598 otu_86271 otu_47360 otu_12174 otu_16400 otu_18169 otu_63270 otu_15151
otu_9275 otu_119297 otu_53118 otu_46781 otu_116316 otu_133492 otu_93154 otu_20647
otu_20563 otu_46976 otu_98272 otu_140457 otu_52337 otu_46746 otu_90646 otu_50322
otu_121341 otu_92985 otu_15443 otu_89496 otu_118866 otu_93891 otu_45786
otu_10984 otu_67066 otu_58539 otu_57876 otu_48934 otu_118656 otu_118505 otu_60192
otu_17040 otu_148035 otu_104349 otu_54895 otu_86925 otu_45910 otu_13323
otu_161045 otu_67002 otu_149245 otu_104804 otu_158105 otu_104256 otu_101912
otu_10506 otu_16841 otu_145427 otu_160101 otu_157376 otu_129256 otu_57738
otu_1294 otu_9416 otu_87153 otu_140504 otu_120213 otu_122651 otu_47406
otu_163800 otu_48583 otu_87394 otu_1100 otu_66659 otu_53353 otu_161113 otu_104211
otu_129357 otu_45244 otu_115920 otu_1721 otu_136528 otu_118304 otu_56370
otu_115728 otu_89084 otu_56152 otu_133571 otu_45054 otu_19154 otu_52488
otu_100935 otu_104164 otu_160282 otu_58831 otu_135031 otu_162273 otu_53652
otu_159548 otu_158406 otu_93290 otu_1049 otu_137759 otu_63251 otu_60410
otu_17251 otu_51587 otu_148195 otu_88352 otu_60200 otu_131229 otu_17425
otu_92446 otu_9529 otu_120027 otu_104743 otu_148619 otu_148115 otu_97710
otu_45443 otu_90546 otu_45370 otu_136512 otu_115737 otu_87226 otu_120731
otu_87530 otu_16765 otu_98263 otu_62410 otu_135714 otu_19715 otu_10676 otu_17702
otu_63076 otu_52516 otu_158002 otu_52066 otu_149444 otu_17919 otu_105079
otu_104904 otu_19570 otu_47663 otu_156741 otu_90983 otu_62040 otu_15378
otu_63131 otu_160035 otu_133353 otu_101392 otu_133993 otu_86550 otu_59347
otu_49206 otu_158658 otu_143445 otu_102308 otu_141572 otu_58978 otu_44515
otu_138259 otu_129489 otu_116730 otu_9794 otu_16685 otu_100564 otu_148168
otu_90913 otu_88327 otu_100649 otu_68062 otu_144842 otu_92474 otu_52548
otu_98494 otu_60258 otu_10847 otu_116011 otu_115797 otu_1572 otu_10304 otu_52484
otu_163717 otu_1858 otu_59411 otu_65726 otu_129242 otu_115978 otu_101008
otu_122707 otu_148310 otu_58250 otu_46658 otu_62524 otu_10239 otu_141555
otu_121133 otu_89135 otu_66360 otu_57503 otu_130751 otu_143007 otu_10471
otu_149238 otu_55394 otu_61464 otu_62305 otu_135521 otu_119938 otu_11310
otu_60886 otu_45423 otu_135718 otu_104836 otu_1761 otu_161300 otu_51352
otu_10855 otu_138743 otu_91230 otu_1075 otu_60363 otu_118957 otu_13519
otu_162204 otu_15843 otu_140087 otu_18618 otu_17167 otu_45121 otu_87688
otu_121391 otu_60472 otu_143542 otu_48990 otu_50502 otu_130137 otu_98121
otu_143399 otu_57838 otu_14137 otu_88567 otu_157061 otu_129668 otu_58867
otu_10401 otu_163926 otu_47563 otu_50669 otu_130013 otu_44803 otu_133896
otu_47377 otu_129561 otu_159925 otu_115626 otu_86433 otu_147869 otu_101870
otu_156915 otu_159194 otu_59816 otu_46957 otu_122277 otu_62731 otu_143489
otu_120536 otu_58772 otu_137389 otu_144895 otu_130870 otu_137492 otu_158240
otu_50485 otu_59690 otu_148350 otu_90523 otu_93895 otu_116478 otu_20463
otu_158111 otu_57350 otu_159145 otu_139323 otu_57442 otu_1400 otu_91563 otu_50614
otu_121070 otu_118615 otu_12270 otu_1032 otu_57935 otu_100883 otu_116260
otu_49794 otu_17764 otu_119187 otu_121628 otu_98662 otu_157900 otu_94206
otu_101907 otu_61353 otu_17303 otu_104631 otu_148962 otu_91422 otu_118707
otu_102138 otu_156907 otu_66625 otu_140428 otu_141525 otu_14156 otu_159735
otu_52282 otu_157170 otu_54458 otu_48600 otu_46400 otu_59293 otu_147975
otu_129492 otu_1435 otu_45302 otu_44840 otu_160868 otu_45360 otu_133951
otu_60924 otu_143167 otu_13870 otu_97922 otu_50843 otu_66559 otu_15171 otu_57179
otu_88191 otu_86707 otu_160302 otu_90656 otu_130993 otu_149206 otu_140436
otu_59846 otu_17015 otu_9837 otu_17733 otu_49951 otu_101006 otu_158926 otu_13703
otu_121687 otu_50403 otu_129931 otu_119629 otu_143335 otu_119277 otu_14298
otu_47869 otu_98899 otu_157890 otu_1591 otu_93574 otu_143516 otu_12157
otu_131733 otu_149711 otu_45813 otu_97670 otu_143088 otu_63915 otu_120051
otu_92218 otu_93016 otu_46984 otu_11152 otu_100543 otu_92525 otu_157561 otu_45849
otu_129917 otu_116961 otu_21604 otu_105246 otu_17294 otu_92751 otu_61654
otu_49690 otu_58532 otu_67608 otu_137210 otu_161566 otu_57166 otu_1984 otu_49077
otu_15464 otu_120298 otu_44236 otu_50972 otu_143371 otu_16881 otu_158357
otu_86675 otu_47084 otu_50169 otu_120097 otu_54080 otu_94527 otu_86357 otu_13072
otu_148243 otu_135738 otu_86791 otu_120373 otu_100726 otu_60496 otu_120851
otu_57752 otu_87090 otu_86245 otu_159807 otu_87405 otu_115658 otu_90321
otu_13318 otu_59757 otu_101045 otu_158052 otu_101663 otu_90738 otu_57325
otu_102340 otu_53962 otu_115889 otu_147884 otu_63348 otu_59707 otu_141540
otu_158987 otu_53153 otu_97578 otu_44620 otu_138922 otu_16216 otu_1749 otu_17275
otu_101221 otu_90799 otu_49464 otu_65937 otu_157252 otu_97998 otu_86864
otu_13153 otu_130884 otu_161482 otu_163960 otu_87342 otu_135987 otu_86803
otu_86320 otu_66100 otu_104748 otu_58612 otu_12513 otu_57248 otu_149322
otu_158345 otu_19878 otu_143342 otu_115706 otu_98994 otu_117352 otu_17873
otu_66839 otu_10559 otu_123086 otu_100479 otu_49335 otu_118733 otu_9346
otu_62169 otu_136519 otu_98094 otu_134114 otu_19464 otu_19484 otu_60788
otu_47824 otu_47288 otu_94423 otu_100605 otu_161520 otu_45660 otu_98163
otu_14932 otu_1481 otu_136563 otu_50624 otu_91443 otu_97639 otu_53575 otu_10339
otu_135032 otu_120175 otu_18666 otu_19040 otu_92550 otu_49872 otu_59629
otu_148709 otu_14113 otu_120020 otu_130794 otu_129552 otu_14368 otu_17120
otu_163954 otu_136613 otu_60550 otu_149291 otu_160133 otu_48766 otu_58835
otu_18168 otu_17956 otu_18392 otu_86258 otu_20240 otu_90128 otu_16488 otu_139310
otu_11724 otu_67111 otu_13298 otu_148201 otu_47283 otu_90923 otu_57565 otu_86826
otu_100670 otu_143091 otu_45510 otu_58109 otu_1602 otu_64441 otu_98492
otu_148308 otu_15691 otu_1308 otu_105064 otu_131826 otu_60484 otu_46089
otu_156804 otu_122280 otu_9260 otu_18745 otu_104721 otu_1179 otu_9402 otu_137396
otu_44520 otu_63665 otu_45633 otu_90612 otu_1853 otu_86424 otu_102080 otu_119486
otu_9778 otu_117060 otu_148013 otu_64211 otu_51761 otu_101872 otu_130781
otu_10424 otu_97499 otu_10917 otu_143106 otu_149104 otu_62146 otu_21622
otu_88250 otu_93428 otu_53619 otu_141416 otu_16006 otu_59114 otu_50512 otu_46871
otu_134927 otu_100574 otu_52939 otu_135787 otu_62277 otu_119488 otu_64061
otu_50374 otu_66928 otu_143299 otu_58618 otu_14772 otu_61077 otu_50286
otu_162315 otu_157317 otu_115633 otu_9819 otu_141354 otu_98017 otu_61970
otu_143082 otu_139359 otu_98660 otu_104791 otu_49184 otu_148368 otu_14183
otu_52336 otu_90696 otu_138732 otu_52656 otu_158461 otu_91404 otu_101154
otu_63973 otu_13417 otu_9967 otu_13971 otu_138936 otu_10162 otu_119194 otu_2075
otu_60280 otu_149227 otu_104438 otu_12463 otu_15074 otu_121161 otu_137505
otu_90538 otu_100703 otu_49409 otu_160448 otu_59047 otu_156580 otu_58899
otu_159339 otu_143360 otu_123539 otu_97593 otu_101234 otu_161513 otu_102319
otu_149496 otu_59520 otu_11681 otu_45099 otu_14191 otu_91172 otu_140375 otu_86234
otu_119139 otu_143053 otu_9970 otu_159992 otu_51509 otu_46441 otu_21171
otu_133787 otu_147983 otu_160614 otu_137133 otu_90518 otu_55091 otu_47523
otu_122988 otu_91923 otu_130816 otu_60704 otu_122927 otu_17591 otu_134194
otu_143210 otu_131435 otu_89940 otu_49277 otu_118779 otu_121259 otu_14341
otu_131266 otu_138742 otu_117906 otu_94030 otu_20759 otu_52575 otu_140323
otu_121361 otu_140411 otu_88100 otu_87013 otu_119122 otu_121118 otu_47472
otu_156783 otu_129234 otu_134957 otu_161427 otu_136614 otu_64806 otu_12060

otu_60280 otu_149227 otu_104438 otu_12463 otu_15074 otu_121161 otu_137505
otu_90538 otu_100703 otu_49409 otu_160448 otu_59047 otu_156580 otu_58899
otu_159339 otu_143360 otu_123539 otu_97593 otu_101234 otu_161513 otu_102319
otu_149496 otu_59520 otu_11681 otu_45099 otu_14191 otu_91172 otu_140375 otu_86234
otu_119139 otu_143053 otu_9970 otu_159992 otu_51509 otu_46441 otu_21171
otu_133787 otu_147983 otu_160614 otu_137133 otu_90518 otu_55091 otu_47523
otu_122988 otu_91923 otu_130816 otu_60704 otu_122927 otu_17591 otu_134194
otu_143210 otu_131435 otu_89940 otu_49277 otu_118779 otu_121259 otu_14341
otu_131266 otu_138742 otu_117906 otu_94030 otu_20759 otu_52575 otu_140323
otu_121361 otu_140411 otu_88100 otu_87013 otu_119122 otu_121118 otu_47472
otu_156783 otu_129234 otu_134957 otu_161427 otu_136614 otu_64806 otu_12060
otu_1669 otu_86120 otu_121661 otu_20723 otu_60503 otu_13463 otu_89257 otu_45008
otu_88438 otu_48181 otu_131954 otu_92540 otu_89076 otu_115773 otu_133892
otu_120123 otu_17405 otu_1291 otu_46060 otu_160095 otu_117674 otu_64986
otu_160791 otu_10549 otu_101189 otu_50658 otu_129416 otu_88331 otu_91632
otu_15956 otu_21760 otu_92176 otu_18107 otu_16251 otu_94998 otu_140235 otu_94599
otu_100557 otu_115802 otu_101272 otu_19199 otu_93060 otu_148254 otu_315
otu_130232 otu_91726 otu_129448 otu_140363 otu_1691 otu_100592 otu_160008
otu_48703 otu_147858 otu_15416 otu_140255 otu_100511 otu_161982 otu_45643
otu_134945 otu_101615 otu_94035 otu_56210 otu_48075 otu_20489 otu_137209
otu_61373 otu_159083 otu_147846 otu_53922 otu_92684 otu_88314 otu_88289
otu_53957 otu_62316 otu_58729 otu_12029 otu_44480 otu_116397 otu_135334
otu_164365 otu_149022 otu_102633 otu_91927 otu_141589 otu_115864 otu_45283
otu_86251 otu_135203 otu_15894 otu_44340 otu_51934 otu_115933 otu_48048
otu_21928 otu_148038 otu_18584 otu_60608 otu_134895 otu_115725 otu_17008
otu_160361 otu_161951 otu_119320 otu_54152 otu_116925 otu_136216 otu_160843
otu_104741 otu_47211 otu_163527 otu_53696 otu_63851 otu_131453 otu_56928
otu_61636 otu_64540 otu_158274 otu_101602 otu_131389 otu_47697 otu_18146
otu_88973 otu_91474 otu_161492 otu_46339 otu_11076 otu_133929 otu_121883
otu_15162 otu_119318 otu_115821 otu_15638 otu_148494 otu_104354 otu_117515
otu_101049 otu_119637 otu_100859 otu_164046 otu_11399 otu_120331 otu_10093
otu_11039 otu_10456 otu_88782 otu_130809 otu_130581 otu_45845 otu_120995
otu_136595 otu_116369 otu_130086 otu_55168 otu_120676 otu_121559 otu_115702
otu_13023 otu_92512 otu_44243 otu_130669 otu_140372 otu_92282 otu_136797
otu_135174 otu_63806 otu_45230 otu_122575 otu_159851 otu_9210 otu_119258
otu_118456 otu_1058 otu_1146 otu_92764 otu_97818 otu_140322 otu_130673 otu_9390
otu_62707 otu_11788 otu_136247 otu_64218 otu_160078 otu_57742 otu_20260
otu_157059 otu_157432 otu_90383 otu_92729 otu_9943 otu_160876 otu_97651 otu_1197
otu_47806 otu_10502 otu_147847 otu_100501 otu_101405 otu_61105 otu_88326
otu_143129 otu_66886 otu_16558 otu_1122 otu_16481 otu_9470 otu_57854 otu_141368
otu_118743 otu_158391 otu_64118 otu_121938 otu_11695 otu_57540 otu_44414
otu_120533 otu_87647 otu_58948 otu_49310 otu_18244 otu_86450 otu_133602 otu_1072
otu_54699 otu_53021 otu_62715 otu_160327 otu_116278 otu_20445 otu_91995
otu_161032 otu_50291 otu_89768 otu_141651 otu_158297 otu_51267 otu_93946
otu_135754 otu_118629 otu_143475 otu_135369 otu_162427 otu_160918 otu_65943
otu_160321 otu_119947 otu_141598 otu_59934 otu_91293 otu_160029 otu_131309
otu_17228 otu_11847 otu_156763 otu_118913 otu_46863 otu_58935 otu_119889
otu_118936 otu_98657 otu_60511 otu_136403 otu_18186 otu_13637 otu_89759
otu_163881 otu_119625 otu_130077 otu_117464 otu_50710 otu_88051 otu_86278
otu_160169 otu_157913 otu_65091 otu_120923 otu_119677 otu_141669 otu_138248
otu_118593 otu_61850 otu_61745 otu_157559 otu_143369 otu_148323 otu_115584
otu_117296 otu_157849 otu_137545 otu_13082 otu_132257 otu_90494 otu_89975
otu_87309 otu_137145 otu_100755 otu_45129 otu_148340 otu_143012 otu_145167
otu_86490 otu_88149 otu_9690 otu_13997 otu_45836 otu_87509 otu_12820 otu_48991
otu_158472 otu_149901 otu_66347 otu_59262 otu_162207 otu_159284 otu_135890
otu_140217 otu_157564 otu_47007 otu_13455 otu_9963 otu_121394 otu_48968
otu_10169 otu_149313 otu_46345 otu_149225 otu_157171 otu_17178 otu_49871
otu_157653 otu_90930 otu_148628 otu_49069 otu_48758 otu_98772 otu_49855
otu_129731 otu_57443 otu_86287 otu_104146 otu_58852 otu_90866 otu_12673
otu_143394 otu_135300 otu_158138 otu_9207 otu_143233 otu_59057 otu_61651
otu_118829 otu_44800 otu_58588 otu_134121 otu_62029 otu_98604 otu_91111 otu_11944
otu_136223 otu_118081 otu_19424 otu_158499 otu_58873 otu_119516 otu_134929
otu_17623 otu_1065 otu_53410 otu_57976 otu_1949 otu_63338 otu_120338 otu_53884
otu_104659 otu_46383 otu_56758 otu_149733 otu_129824 otu_118068 otu_129641
otu_46590 otu_47313 otu_1110 otu_97720 otu_16811 otu_141596 otu_48164 otu_59386
otu_94104 otu_118775 otu_16469 otu_54435 otu_17792 otu_119828 otu_66983 otu_86129
otu_54760 otu_50997 otu_15752 otu_18348 otu_86399 otu_49883 otu_87699 otu_59065
otu_140273 otu_47578 otu_50945 otu_121955 otu_10286 otu_164035 otu_89391
otu_141705 otu_158849 otu_118970 otu_58956 otu_98554 otu_120005 otu_143341
otu_148145 otu_141609 otu_9238 otu_63676 otu_148638 otu_101009 otu_18452
otu_91435 otu_18066 otu_140148 otu_119146 otu_157997 otu_47413 otu_54057
otu_59556 otu_149327 otu_15277 otu_1135 otu_118799 otu_135683 otu_18029
otu_141412 otu_92555 otu_59183 otu_55283 otu_148524 otu_145460 otu_49256
otu_50824 otu_14148 otu_46562 otu_53338 otu_44648 otu_46535 otu_138045
otu_116768 otu_52526 otu_49791 otu_164123 otu_118831 otu_160332 otu_102294
otu_286 otu_15185 otu_64620 otu_160464 otu_104853 otu_58105 otu_135068 otu_90659
otu_51223 otu_133920 otu_13810 otu_14312 otu_10372 otu_91441 otu_1841 otu_92193
otu_101343 otu_45228 otu_149284 otu_17199 otu_279 otu_120295 otu_130908
otu_121606 otu_52476 otu_102247 otu_129377 otu_148445 otu_46184 otu_134019
otu_117280 otu_156731 otu_13209 otu_50342 otu_121109 otu_61253 otu_11420
otu_10507 otu_157887 otu_88720 otu_135128 otu_137998 otu_20608 otu_12055
otu_121956 otu_21754 otu_149436 otu_130108 otu_93176 otu_145069 otu_60989
otu_47626 otu_46144 otu_44623 otu_45554 otu_19832 otu_56694 otu_132176 otu_45508
otu_267 otu_92192 otu_46379 otu_46246 otu_47683 otu_121238 otu_44385 otu_119308
otu_65630 otu_148101 otu_143134 otu_9446 otu_11745 otu_47144 otu_65074 otu_44248
otu_129477 otu_44809 otu_58749 otu_102398 otu_157990 otu_58366 otu_10144
otu_19962 otu_158498 otu_117532 otu_118322 otu_147982 otu_137713 otu_62746
otu_53465 otu_57864 otu_148460 otu_46564 otu_93083 otu_140193 otu_61374
otu_66679 otu_16501 otu_121200 otu_12221 otu_134244 otu_13928 otu_86144
otu_60076 otu_141632 otu_60764 otu_13958 otu_119856 otu_100598 otu_62323
otu_119725 otu_50850 otu_117548 otu_98619 otu_148193 otu_68759 otu_148477
otu_132180 otu_121518 otu_101249 otu_132189 otu_117825 otu_51552 otu_64674
otu_87720 otu_46252 otu_143562 otu_117610 otu_15358 otu_140464 otu_93765
otu_116585 otu_47968 otu_19743 otu_131948 otu_10939 otu_149275 otu_104735
otu_148242 otu_91537 otu_48193 otu_117435 otu_115570 otu_94088 otu_57028
otu_19013 otu_102076 otu_51180 otu_117007 otu_9239 otu_17030 otu_101120 otu_56703
otu_19382 otu_54270 otu_61391 otu_101810 otu_44390 otu_92620 otu_18273 otu_53210
otu_52010 otu_12433 otu_9952 otu_63621 otu_10324 otu_46784 otu_17016 otu_161628
otu_14624 otu_87525 otu_140485 otu_60547 otu_130867 otu_59746 otu_100659
otu_47785 otu_11963 otu_141376 otu_61981 otu_119040 otu_98455 otu_11044 otu_65595
otu_140170 otu_11453 otu_137557 otu_138691 otu_118648 otu_17649 otu_64050
otu_160356 otu_93340 otu_119924 otu_45016 otu_157249 otu_87446 otu_64943
otu_129364 otu_10044 otu_18632 otu_161254 otu_1750 otu_130305 otu_16987
otu_121483 otu_143541 otu_86730 otu_45396 otu_121019 otu_92085 otu_120289
otu_18484 otu_57963 otu_143594 otu_50631 otu_105016 otu_45496 otu_9942
otu_138269 otu_145038 otu_9627 otu_121869 otu_141459 otu_117707 otu_118634
otu_140093 otu_100493 otu_45406 otu_135229 otu_13693 otu_118738 otu_100744
otu_104303 otu_59084 otu_137540 otu_163544 otu_48148 otu_16932 otu_140555
otu_101762 otu_58507 otu_44226 otu_145446 otu_61060 otu_94265 otu_17883

otu_52010 otu_12433 otu_9952 otu_63621 otu_10324 otu_46784 otu_17016 otu_161628
otu_14624 otu_87525 otu_140485 otu_60547 otu_130867 otu_59746 otu_100659
otu_47785 otu_11963 otu_141376 otu_61981 otu_119040 otu_98455 otu_11044 otu_65595
otu_140170 otu_11453 otu_137557 otu_138691 otu_118648 otu_17649 otu_64050
otu_160356 otu_93340 otu_119924 otu_45016 otu_157249 otu_87446 otu_64943
otu_129364 otu_10044 otu_18632 otu_161254 otu_1750 otu_130305 otu_16987
otu_121483 otu_143541 otu_86730 otu_45396 otu_121019 otu_92085 otu_120289
otu_18484 otu_57963 otu_143594 otu_50631 otu_105016 otu_45496 otu_9942
otu_138269 otu_145038 otu_9627 otu_121869 otu_141459 otu_117707 otu_118634
otu_140093 otu_100493 otu_45406 otu_135229 otu_13693 otu_118738 otu_100744
otu_104303 otu_59084 otu_137540 otu_163544 otu_48148 otu_16932 otu_140555
otu_101762 otu_58507 otu_44226 otu_145446 otu_61060 otu_94265 otu_17883
otu_160337 otu_130743 otu_149364 otu_86808 otu_91025 otu_54985 otu_21535
otu_148696 otu_1526 otu_133764 otu_163777 otu_134980 otu_65025 otu_158140
otu_45999 otu_120347 otu_92072 otu_97884 otu_60622 otu_100458 otu_16793
otu_67115 otu_46469 otu_54307 otu_156652 otu_119962 otu_120334 otu_87206
otu_55155 otu_49272 otu_135661 otu_120000 otu_138718 otu_141389 otu_16579
otu_87067 otu_145284 otu_162026 otu_137475 otu_21145 otu_16392 otu_133753
otu_46489 otu_59754 otu_133429 otu_2000 otu_122731 otu_156920 otu_138735
otu_115979 otu_21176 otu_18728 otu_9615 otu_94332 otu_16527 otu_20291 otu_18717
otu_118998 otu_47569 otu_91477 otu_161177 otu_47679 otu_138072 otu_60898
otu_18407 otu_86719 otu_87091 otu_121599 otu_131565 otu_65300 otu_17453
otu_10587 otu_45638 otu_149196 otu_53501 otu_163505 otu_59935 otu_91372
otu_46709 otu_136501 otu_18230 otu_101970 otu_100516 otu_66591 otu_55849
otu_57513 otu_117077 otu_104878 otu_9786 otu_89556 otu_66923 otu_137187
otu_62650 otu_138081 otu_159665 otu_19650 otu_162743 otu_62230 otu_11778
otu_45580 otu_147891 otu_143308 otu_1731 otu_52433 otu_90710 otu_93610
otu_140451 otu_60136 otu_15420 otu_60577 otu_129249 otu_137772 otu_16204
otu_105112 otu_118784 otu_61571 otu_133566 otu_48099 otu_58732 otu_89167
otu_145611 otu_59095 otu_67122 otu_158871 otu_159820 otu_64480 otu_97507
otu_98290 otu_10026 otu_86336 otu_1144 otu_116562 otu_9315 otu_20266 otu_17203
otu_57364 otu_116425 otu_1349 otu_20009 otu_92463 otu_93193 otu_157438 otu_57740
otu_160884 otu_116642 otu_47186 otu_16637 otu_13079 otu_143408 otu_119400
otu_46505 otu_45163 otu_57441 otu_17072 otu_135210 otu_145184 otu_58074
otu_129375 otu_117080 otu_59817 otu_46161 otu_93698 otu_1251 otu_138672
otu_119058 otu_51631 otu_104640 otu_51506 otu_48677 otu_10296 otu_157918
otu_160347 otu_149093 otu_157882 otu_97565 otu_50279 otu_285 otu_87152 otu_61771
otu_91121 otu_58849 otu_45309 otu_51188 otu_50161 otu_53954 otu_86183 otu_48586
otu_149717 otu_59932 otu_160051 otu_13062 otu_13606 otu_104783 otu_54076
otu_53628 otu_139319 otu_141390 otu_139002 otu_148383 otu_102105 otu_120271
otu_145275 otu_53985 otu_130345 otu_87742 otu_100512 otu_20129 otu_16781
otu_49003 otu_117725 otu_64749 otu_140154 otu_119752 otu_47500 otu_21311
otu_122457 otu_131336 otu_120712 otu_104689 otu_119573 otu_102176 otu_87045
otu_141337 otu_54361 otu_141658 otu_91591 otu_17128 otu_119997 otu_16982
otu_87173 otu_1288 otu_156866 otu_149360 otu_1068 otu_1928 otu_158475 otu_57729
otu_61315 otu_143040 otu_9576 otu_105096 otu_19267 otu_136630 otu_14228
otu_62182 otu_119112 otu_12676 otu_91316 otu_86701 otu_18536 otu_148888 otu_87037
otu_157785 otu_137965 otu_17196 otu_160411 otu_61121 otu_44716 otu_86603
otu_164023 otu_21107 otu_133638 otu_118880 otu_16894 otu_51053 otu_18783
otu_149455 otu_116059 otu_149204 otu_116900 otu_56716 otu_10739 otu_9283
otu_135435 otu_117963 otu_122121 otu_104167 otu_59858 otu_16953 otu_143074
otu_44833 otu_58953 otu_44298 otu_131863 otu_60612 otu_135645 otu_16725
otu_98951 otu_135652 otu_48536 otu_123049 otu_93182 otu_134153 otu_141591
otu_162242 otu_149412 otu_117860 otu_136370 otu_100965 otu_104149 otu_147947
otu_47816 otu_158281 otu_18363 otu_140101 otu_94325 otu_16383 otu_45417
otu_148255 otu_49017 otu_121402 otu_129821 otu_157270 otu_87053 otu_156644
otu_44448 otu_13736 otu_20188 otu_137955 otu_48378 otu_47420 otu_98284 otu_57955
otu_156878 otu_115881 otu_48501 otu_52968 otu_53893 otu_60771 otu_52724
otu_119174 otu_87117 otu_19014 otu_48708 otu_51155 otu_104328 otu_55370 otu_87973
otu_91285 otu_133425 otu_57171 otu_68329 otu_49207 otu_9586 otu_45325 otu_131345
otu_56292 otu_120309 otu_16281 otu_18433 otu_45666 otu_9620 otu_116761 otu_117114
otu_143080 otu_9400 otu_98609 otu_161872 otu_129738 otu_9476 otu_98675
otu_117274 otu_148266 otu_45699 otu_140179 otu_19292 otu_93949 otu_19689
otu_118387 otu_11048 otu_46862 otu_119038 otu_122110 otu_137463 otu_159984
otu_161080 otu_87339 otu_45034 otu_159777 otu_135461 otu_156953 otu_18925
otu_49916 otu_17646 otu_101631 otu_92077 otu_145541 otu_120988 otu_157874
otu_20043 otu_132350 otu_102047 otu_48998 otu_148328 otu_119136 otu_54297
otu_292 otu_157178 otu_44251 otu_121545 otu_148096 otu_9293 otu_60420 otu_161023
otu_46436 otu_145043 otu_2019 otu_93850 otu_1350 otu_49287 otu_15115 otu_59394
otu_61472 otu_9930 otu_61067 otu_49111 otu_148763 otu_65488 otu_11664 otu_164367
otu_91708 otu_63269 otu_87583 otu_17618 otu_9919 otu_161888 otu_119622 otu_16347
otu_141559 otu_10283 otu_18191 otu_157069 otu_17714 otu_134047 otu_18723
otu_130476 otu_48575 otu_91515 otu_89922 otu_11305 otu_157802 otu_9563 otu_61875
otu_1966 otu_10574 otu_138854 otu_158284 otu_1517 otu_64801 otu_57940 otu_56637
otu_129600 otu_97515 otu_94308 otu_57865 otu_66232 otu_159614 otu_87517
otu_86883 otu_149961 otu_62347 otu_12783 otu_123322 otu_55440 otu_44530
otu_160242 otu_159796 otu_87291 otu_59132 otu_89286 otu_16523 otu_129819
otu_49213 otu_118074 otu_145308 otu_97751 otu_1111 otu_16189 otu_139314 otu_11249
otu_48425 otu_105028 otu_138104 otu_86682 otu_51567 otu_92721 otu_132207
otu_11907 otu_120204 otu_59339 otu_93036 otu_120382 otu_97965 otu_45962
otu_58093 otu_19326 otu_87232 otu_131097 otu_119349 otu_9552 otu_119524
otu_159971 otu_50552 otu_88161 otu_98893 otu_66053 otu_61142 otu_104363
otu_141625 otu_130355 otu_97500 otu_59593 otu_12624 otu_66826 otu_130782
otu_87640 otu_9432 otu_90512 otu_122831 otu_93435 otu_89404 otu_116385 otu_118624
otu_58804 otu_134904 otu_118744 otu_97725 otu_129926 otu_120801 otu_47231
otu_9785 otu_119610 otu_9696 otu_46115 otu_45433 otu_44660 otu_137213 otu_100892
otu_147928 otu_91992 otu_61780 otu_134955 otu_12682 otu_91019 otu_59026
otu_53777 otu_100887 otu_49716 otu_120761 otu_117387 otu_46924 otu_12955
otu_58681 otu_120141 otu_157828 otu_115959 otu_57924 otu_129311 otu_60199
otu_119276 otu_48027 otu_136518 otu_98735 otu_117204 otu_16972 otu_47072
otu_120062 otu_48787 otu_18633 otu_94963 otu_130847 otu_137931 otu_66733
otu_133329 otu_17755 otu_57375 otu_19969 otu_1216 otu_115843 otu_62461 otu_45300
otu_44650 otu_123264 otu_160409 otu_115980 otu_10582 otu_14550 otu_9556
otu_97627 otu_86569 otu_122971 otu_140419 otu_140394 otu_100765 otu_12324
otu_60895 otu_9404 otu_18579 otu_118328 otu_86872 otu_159276 otu_133604
otu_53581 otu_156648 otu_55883 otu_61120 otu_156879 otu_116582 otu_45106
otu_53565 otu_46771 otu_11853 otu_129351 otu_119045 otu_86132 otu_10463
otu_119011 otu_101612 otu_48083 otu_63896 otu_91214 otu_87064 otu_16182 otu_16764
otu_160513 otu_61691 otu_141741 otu_157237 otu_120275 otu_57950 otu_11898
otu_138907 otu_10513 otu_47314 otu_137484 otu_17863 otu_91010 otu_18756
otu_18257 otu_46104 otu_157406 otu_149787 otu_93055 otu_137193 otu_49395
otu_58037 otu_45624 otu_115914 otu_140365 otu_59407 otu_157220 otu_65427
otu_45792 otu_86654 otu_159439 otu_64769 otu_121860 otu_53342 otu_47857
otu_54058 otu_135682 otu_17138 otu_123011 otu_136846 otu_90480 otu_51728
otu_101086 otu_13498 otu_129582 otu_45487 otu_47638 otu_46468 otu_16228
otu_67679 otu_115814 otu_122406 otu_100867 otu_10885 otu_95060 otu_97814
otu_10396 otu_86318 otu_91196 otu_135292 otu_130016 otu_87526 otu_57812
otu_157300 otu_118106 otu_131137 otu_93307 otu_129918 otu_116027 otu_121329
otu_102156 otu_60100 otu_51555 otu_61678 otu_16314 otu_140529 otu_135730

[illegible]
otu_160513 otu_61691 otu_141741 otu_157237 otu_120275 otu_57950 otu_11898
otu_138907 otu_10513 otu_47314 otu_137484 otu_17863 otu_91010 otu_18756
otu_18257 otu_46104 otu_157406 otu_149787 otu_93055 otu_137193 otu_49395
otu_58037 otu_45624 otu_115914 otu_140365 otu_59407 otu_157220 otu_65427
otu_45792 otu_86654 otu_159439 otu_64769 otu_121860 otu_53342 otu_47857
otu_54058 otu_135682 otu_17138 otu_123011 otu_136846 otu_90480 otu_51728
otu_101086 otu_13498 otu_129582 otu_45487 otu_47638 otu_46468 otu_16228
otu_67679 otu_115814 otu_122406 otu_100867 otu_10885 otu_95060 otu_97814
otu_10396 otu_86318 otu_91196 otu_135292 otu_130016 otu_87526 otu_57812
otu_157300 otu_118106 otu_131137 otu_93307 otu_129918 otu_116027 otu_121329
otu_102156 otu_60100 otu_51555 otu_61678 otu_16314 otu_140529 otu_135730
otu_45937 otu_91695 otu_140492 otu_159428 otu_86614 otu_48263 otu_133648
otu_59490 otu_141281 otu_120754 otu_136374 otu_94241 otu_59306 otu_16604
otu_19976 otu_116932 otu_135451 otu_50781 otu_148224 otu_59881 otu_129789
otu_156895 otu_138828 otu_129604 otu_68511 otu_130909 otu_49679 otu_57521
otu_135757 otu_116133 otu_92338 otu_18775 otu_105161 otu_101516 otu_59029
otu_136356 otu_90892 otu_53149 otu_117397 otu_138712 otu_66758 otu_149345
otu_56163 otu_91874 otu_89491 otu_47183 otu_44568 otu_2027 otu_159867 otu_116340
otu_92253 otu_116141 otu_54700 otu_62618 otu_159739 otu_97734 otu_1082 otu_61042
otu_102742 otu_137234 otu_9951 otu_63809 otu_143352 otu_88155 otu_89660
otu_97706 otu_11179 otu_156962 otu_115756 otu_136538 otu_94221 otu_115839
otu_9570 otu_18453 otu_13439 otu_101033 otu_48555 otu_135470 otu_17362 otu_90567
otu_119480 otu_86641 otu_1825 otu_104376 otu_94478 otu_92363 otu_160297
otu_163650 otu_62867 otu_157542 otu_16641 otu_47545 otu_57451 otu_62671
otu_116717 otu_86754 otu_143148 otu_47959 otu_11408 otu_65888 otu_61688
otu_135051 otu_66361 otu_93253 otu_121306 otu_10104 otu_89500 otu_94149
otu_45274 otu_88503 otu_156548 otu_162238 otu_45342 otu_59330 otu_45013
otu_10486 otu_88685 otu_59474 otu_19770 otu_49728 otu_92438 otu_15390 otu_163694
otu_138666 otu_86987 otu_52218 otu_48464 otu_87421 otu_157475 otu_68450
otu_158884 otu_140488 otu_148008 otu_93245 otu_57492 otu_98461 otu_9394 otu_81
otu_9739 otu_141513 otu_56781 otu_149387 otu_55396 otu_49705 otu_157941
otu_57691 otu_46218 otu_44877 otu_21475 otu_97876 otu_143546 otu_90401
otu_149486 otu_161078 otu_16297 otu_9614 otu_16292 otu_61133 otu_55248 otu_93357
otu_130742 otu_51737 otu_57989 otu_48969 otu_145345 otu_18250 otu_44437
otu_64417 otu_10989 otu_141721 otu_64677 otu_10354 otu_49076 otu_48112 otu_118452
otu_17914 otu_91287 otu_161139 otu_163536 otu_11256 otu_89800 otu_9299 otu_115690
otu_158878 otu_117284 otu_52934 otu_16373 otu_44582 otu_46358 otu_116630
otu_46159 otu_45994 otu_104180 otu_46447 otu_12617 otu_149621 otu_14881
otu_92906 otu_16677 otu_117764 otu_161154 otu_131758 otu_117167 otu_61106
otu_164162 otu_49990 otu_158589 otu_47654 otu_20535 otu_136389 otu_11569
otu_115986 otu_14111 otu_130238 otu_86974 otu_143218 otu_57982 otu_48259
otu_46145 otu_47883 otu_104705 otu_148043 otu_89239 otu_160011 otu_157330
otu_49373 otu_143232 otu_58567 otu_62371 otu_93277 otu_102315 otu_157201
otu_140430 otu_58026 otu_51234 otu_140425 otu_46258 otu_16384 otu_15905
otu_18416 otu_116347 otu_91915 otu_137694 otu_157466 otu_65277 otu_51620
otu_118140 otu_92179 otu_60120 otu_88818 otu_45190 otu_122028 otu_62055
otu_135413 otu_11625 otu_159142 otu_129535 otu_17079 otu_51257 otu_51712
otu_119701 otu_139452 otu_161205 otu_59704 otu_149663 otu_104316 otu_54000
otu_88062 otu_13445 otu_19074 otu_45079 otu_117439 otu_16275 otu_161975
otu_53679 otu_135642 otu_51430 otu_134946 otu_18933 otu_11708 otu_91156 otu_87855
otu_49155 otu_45063 otu_158463 otu_9758 otu_91029 otu_53570 otu_86728 otu_138703
otu_149474 otu_164101 otu_58087 otu_90651 otu_157284 otu_48194 otu_59051
otu_118195 otu_148037 otu_48102 otu_86560 otu_18180 otu_101424 otu_130954
otu_62588 otu_52647 otu_67373 otu_159094 otu_160307 otu_64255 otu_13373
otu_57336 otu_118641 otu_136688 otu_64368 otu_134991 otu_17517 otu_148363
otu_105293 otu_135079 otu_149724 otu_55487 otu_160116 otu_91520 otu_87119
otu_50692 otu_118839 otu_101069 otu_137578 otu_18090 otu_61116 otu_149247
otu_90505 otu_157212 otu_97535 otu_156736 otu_86483 otu_18026 otu_88031
otu_46403 otu_59957 otu_61896 otu_309 otu_136394 otu_133525 otu_66417 otu_14746
otu_149716 otu_65574 otu_158730 otu_50334 otu_158224 otu_140140 otu_149299
otu_133707 otu_164003 otu_116814 otu_137219 otu_138758 otu_280 otu_1521
otu_149212 otu_49290 otu_58345 otu_49641 otu_101083 otu_141579 otu_10213
otu_90587 otu_11476 otu_144890 otu_93316 otu_97503 otu_138056 otu_86856
otu_160124 otu_160582 otu_118553 otu_93973 otu_58267 otu_50620 otu_97470
otu_15338 otu_100914 otu_149400 otu_119526 otu_89547 otu_51009 otu_58533
otu_119543 otu_101986 otu_158167 otu_157025 otu_161231 otu_136835 otu_17062
otu_129587 otu_56924 otu_164105 otu_158495 otu_147939 otu_104825 otu_118077
otu_57318 otu_133658 otu_12267 otu_52004 otu_98124 otu_10154 otu_157716
otu_137005 otu_115810 otu_86223 otu_44729 otu_47264 otu_141628 otu_122737
otu_93590 otu_157630 otu_135713 otu_138262 otu_50763 otu_45227 otu_89367
otu_120238 otu_119317 otu_10429 otu_93828 otu_19017 otu_160638 otu_56808
otu_120826 otu_66490 otu_105071 otu_45460 otu_156637 otu_16721 otu_157440
otu_16331 otu_91590 otu_50749 otu_116899 otu_93019 otu_62862 otu_13685 otu_63323
otu_51886 otu_97551 otu_160393 otu_45569 otu_14014 otu_104153 otu_135005
otu_58796 otu_160842 otu_122099 otu_160557 otu_53349 otu_1980 otu_129246
otu_89587 otu_48175 otu_122953 otu_45025 otu_45969 otu_102436 otu_120933
otu_20715 otu_18391 otu_59195 otu_119182 otu_44373 otu_92505 otu_157576
otu_15038 otu_47197 otu_147957 otu_15404 otu_139347 otu_130716 otu_44512
otu_157325 otu_147910 otu_60167 otu_117230 otu_45912 otu_158407 otu_138318
otu_139396 otu_53002 otu_149391 otu_145263 otu_145098 otu_44664 otu_56675
otu_15118 otu_45684 otu_129315 otu_120164 otu_143004 otu_63429 otu_1186
otu_116382 otu_91279 otu_49648 otu_131394 otu_161295 otu_102444 otu_60705
otu_131427 otu_88261 otu_61857 otu_101862 otu_93043 otu_53660 otu_16600
otu_59731 otu_88394 otu_65796 otu_13588 otu_1900 otu_138752 otu_101949 otu_17741
otu_62353 otu_163945 otu_90860 otu_163907 otu_91958 otu_143551 otu_138241
otu_116448 otu_12312 otu_44254 otu_11316 otu_98505 otu_144860 otu_60442 otu_51817
otu_139320 otu_48960 otu_9441 otu_98171 otu_130828 otu_1657 otu_20229 otu_13620
otu_46154 otu_65020 otu_44893 otu_16688 otu_140540 otu_123638 otu_163924
otu_148517 otu_121039 otu_11894 otu_61076 otu_118992 otu_59598 otu_64985
otu_50128 otu_44561 otu_157179 otu_141371 otu_48999 otu_1611 otu_145008
otu_135542 otu_52697 otu_62435 otu_157233 otu_17824 otu_12584 otu_130831
otu_101929 otu_138284 otu_157088 otu_102239 otu_149228 otu_105014 otu_102137
otu_116574 otu_148315 otu_62303 otu_88039 otu_1967 otu_91939 otu_14203 otu_45483
otu_54054 otu_58411 otu_9291 otu_63371 otu_13192 otu_98454 otu_86475 otu_46283
otu_21470 otu_51648 otu_147927 otu_102566 otu_138057 otu_13299 otu_90722
otu_118942 otu_133911 otu_95250 otu_87870 otu_90528 otu_101962 otu_67079
otu_18832 otu_60927 otu_86605 otu_87202 otu_86200 otu_11671 otu_52182 otu_61778
otu_67448 otu_9628 otu_148516 otu_148701 otu_160596 otu_45448 otu_62857
otu_64809 otu_48638 otu_51243 otu_61856 otu_47935 otu_122367 otu_100492
otu_129487 otu_104919 otu_86338 otu_58624 otu_158446 otu_47862 otu_44590
otu_136833 otu_158662 otu_97666 otu_48499 otu_89619 otu_135895 otu_95037
otu_59149 otu_10289 otu_123191 otu_11537 otu_92902 otu_58944 otu_48229 otu_16715
otu_62589 otu_97947 otu_11008 otu_49703 otu_61748 otu_15245 otu_98496 otu_120015
otu_11650 otu_86993 otu_14097 otu_16235 otu_156655 otu_162812 otu_118868
otu_139433 otu_50377 otu_51929 otu_61303 otu_101487 otu_20411 otu_119272
otu_63142 otu_145514 otu_91083 otu_116918 otu_90201 otu_65135 otu_121052
otu_119461 otu_164020 otu_140085 otu_48714 otu_62434 otu_65877 otu_56536
otu_57573 otu_55574 otu_104207 otu_18526 otu_53846 otu_91424 otu_104476
[illegible]

otu_67448 otu_9628 otu_148516 otu_148701 otu_160596 otu_45448 otu_62857
otu_64809 otu_48638 otu_51243 otu_61856 otu_47935 otu_122367 otu_100492
otu_129487 otu_104919 otu_86338 otu_58624 otu_158446 otu_47862 otu_44590
otu_136833 otu_158662 otu_97666 otu_48499 otu_89619 otu_135895 otu_95037
otu_59149 otu_10289 otu_123191 otu_11537 otu_92902 otu_58944 otu_48229 otu_16715
otu_62589 otu_97947 otu_11008 otu_49703 otu_61748 otu_15245 otu_98496 otu_120015
otu_11650 otu_86993 otu_14097 otu_16235 otu_156655 otu_162812 otu_118868
otu_139433 otu_50377 otu_51929 otu_61303 otu_101487 otu_20411 otu_119272
otu_63142 otu_145514 otu_91083 otu_116918 otu_90201 otu_65135 otu_121052
otu_119461 otu_164020 otu_140085 otu_48714 otu_62434 otu_65877 otu_56536
otu_57573 otu_55574 otu_104207 otu_18526 otu_53846 otu_91424 otu_104476
otu_98580 otu_87912 otu_1102 otu_98479 otu_86342 otu_46278 otu_86744 otu_60063
otu_1037 otu_137006 otu_105097 otu_11465 otu_86435 otu_47033 otu_12407 otu_10153
otu_64989 otu_90742 otu_14705 otu_158048 otu_12768 otu_130309 otu_89452
otu_44359 otu_105078 otu_158054 otu_122760 otu_50893 otu_130371 otu_134043
otu_60430 otu_15731 otu_158673 otu_16771 otu_100881 otu_46162 otu_91110
otu_119346 otu_101417 otu_16269 otu_20155 otu_101184 otu_57787 otu_140147
otu_147906 otu_138673 otu_129563 otu_147950 otu_18378 otu_92368 otu_50483
otu_68536 otu_12582 otu_12404 otu_117031 otu_57414 otu_156913 otu_91460
otu_12101 otu_164200 otu_133454 otu_61570 otu_16966 otu_100923 otu_55701
otu_86298 otu_119973 otu_57937 otu_100562 otu_102272 otu_117064 otu_136249
otu_118405 otu_115661 otu_115787 otu_101504 otu_143485 otu_10740 otu_101366
otu_119326 otu_145462 otu_45407 otu_157924 otu_59545 otu_46495 otu_47949
otu_46111 otu_14343 otu_50827 otu_104784 otu_119873 otu_19387 otu_141661
otu_44300 otu_17947 otu_12448 otu_58212 otu_149182 otu_66190 otu_164006
otu_97719 otu_14352 otu_101043 otu_143336 otu_57459 otu_48062 otu_87627
otu_59059 otu_149728 otu_20828 otu_159805 otu_140309 otu_57796 otu_135211
otu_9344 otu_12993 otu_100586 otu_86714 otu_139440 otu_91599 otu_130411
otu_115917 otu_49398 otu_149582 otu_18688 otu_86573 otu_58648 otu_157948
otu_141338 otu_133434 otu_47232 otu_17212 otu_51295 otu_57371 otu_21150 otu_9588
otu_19053 otu_16888 otu_60130 otu_86705 otu_119684 otu_162001 otu_101262
otu_118914 otu_58056 otu_51446 otu_119478 otu_50695 otu_123308 otu_10902
otu_121526 otu_119497 otu_119181 otu_1117 otu_1593 otu_19629 otu_163627 otu_45662
otu_129548 otu_137417 otu_129465 otu_149208 otu_50278 otu_13466 otu_88847
otu_9936 otu_12521 otu_115715 otu_93558 otu_20249 otu_135680 otu_90619 otu_52299
otu_101460 otu_95079 otu_157192 otu_133989 otu_117006 otu_10806 otu_134948
otu_163934 otu_19333 otu_93002 otu_92967 otu_160975 otu_86851 otu_65953
otu_102005 otu_20289 otu_67523 otu_130951 otu_53573 otu_10597 otu_158036
otu_130761 otu_100600 otu_120637 otu_139445 otu_145479 otu_15367 otu_121695
otu_98400 otu_17343 otu_16823 otu_17169 otu_90609 otu_122397 otu_102061
otu_137408 otu_148059 otu_91744 otu_65778 otu_53528 otu_18461 otu_156852
otu_16909 otu_17822 otu_130251 otu_133522 otu_50175 otu_138229 otu_63997
otu_116632 otu_47525 otu_148128 otu_88050 otu_94663 otu_16470 otu_17709 otu_11850
otu_91129 otu_116668 otu_145136 otu_100795 otu_98952 otu_87302 otu_45255
otu_49753 otu_136697 otu_92579 otu_15264 otu_135669 otu_148180 otu_48394
otu_141574 otu_10687 otu_92857 otu_149211 otu_116598 otu_20520 otu_122155
otu_10619 otu_87169 otu_46785 otu_102730 otu_20576 otu_64344 otu_18847 otu_60425
otu_164038 otu_58021 otu_60464 otu_92753 otu_87886 otu_58131 otu_118947
otu_16210 otu_105036 otu_158071 otu_45088 otu_63549 otu_157288 otu_60379
otu_57545 otu_12299 otu_59709 otu_67989 otu_131759 otu_15467 otu_11258
otu_100804 otu_45635 otu_44791 otu_105010 otu_58180 otu_130180 otu_159628
otu_120891 otu_163557 otu_49680 otu_86954 otu_59040 otu_115823 otu_143347
otu_90961 otu_137739 otu_89410 otu_1214 otu_50910 otu_59566 otu_148044
otu_160239 otu_86755 otu_49159 otu_97918 otu_137135 otu_45828 otu_116068
otu_17745 otu_116116 otu_120363 otu_14832 otu_63975 otu_12113 otu_119355
otu_58950 otu_143205 otu_21301 otu_104993 otu_160964 otu_104797 otu_19203
otu_16931 otu_135943 otu_156564 otu_20955 otu_61698 otu_91263 otu_86767
otu_98575 otu_15279 otu_47330 otu_119148 otu_10362 otu_98132 otu_149386
otu_44227 otu_117999 otu_1346 otu_135034 otu_11997 otu_91445 otu_133490 otu_86173
otu_158518 otu_105118 otu_139363 otu_121800 otu_12236 otu_94686 otu_116512
otu_158384 otu_157581 otu_122973 otu_116328 otu_149265 otu_9716 otu_17647
otu_140434 otu_99061 otu_100668 otu_19644 otu_48033 otu_122182 otu_141645
otu_97609 otu_86262 otu_47800 otu_157287 otu_100736 otu_159202 otu_14477
otu_59009 otu_137200 otu_17697 otu_49372 otu_139413 otu_12683 otu_47945
otu_46327 otu_16303 otu_10724 otu_16245 otu_18945 otu_97591 otu_1891 otu_137753
otu_14283 otu_46077 otu_116384 otu_56775 otu_13145 otu_131083 otu_100572
otu_156752 otu_49222 otu_48486 otu_133563 otu_16358 otu_17339 otu_86848
otu_64872 otu_149646 otu_120118 otu_58482 otu_122297 otu_66405 otu_10201
otu_144851 otu_90753 otu_115944 otu_10751 otu_47484 otu_116282 otu_17223
otu_67264 otu_57316 otu_147882 otu_98807 otu_135173 otu_141319 otu_48944
otu_117711 otu_115964 otu_17059 otu_163938 otu_51043 otu_102074 otu_119847
otu_20848 otu_9287 otu_117263 otu_156772 otu_15225 otu_157050 otu_148299
otu_133790 otu_92323 otu_148571 otu_18508 otu_86426 otu_59312 otu_101543
otu_143358 otu_66525 otu_50047 otu_9504 otu_55109 otu_60439 otu_92457 otu_65385
otu_90396 otu_54141 otu_11050 otu_16253 otu_118804 otu_138789 otu_59038 otu_57771
otu_117842 otu_100597 otu_18862 otu_16395 otu_98719 otu_44245 otu_45226
otu_116836 otu_133831 otu_93348 otu_149430 otu_51457 otu_91087 otu_16828
otu_53393 otu_131040 otu_16474 otu_136913 otu_148033 otu_98894 otu_163676
otu_14265 otu_86301 otu_58264 otu_115858 otu_16631 otu_87514 otu_10458 otu_14400
otu_133935 otu_131786 otu_136537 otu_10049 otu_10815 otu_48585 otu_143443
otu_102297 otu_10933 otu_159963 otu_86935 otu_97586 otu_101721 otu_10306
otu_14144 otu_119765 otu_18395 otu_9807 otu_136996 otu_46465 otu_97792 otu_10607
otu_53466 otu_49938 otu_61122 otu_45899 otu_98487 otu_100821 otu_100993
otu_86944 otu_91399 otu_148476 otu_61779 otu_135386 otu_44386 otu_130011
otu_92414 otu_102175 otu_17118 otu_116205 otu_133665 otu_115678 otu_17236
otu_117417 otu_58602 otu_48073 otu_138687 otu_57727 otu_11237 otu_156859
otu_48006 otu_16181 otu_63673 otu_104608 otu_10516 otu_94775 otu_52954 otu_12131
otu_117422 otu_135653 otu_148024 otu_158620 otu_118682 otu_98668 otu_57866
otu_133907 otu_131387 otu_140568 otu_66067 otu_61883 otu_1806 otu_48788
otu_158221 otu_12329 otu_91291 otu_140194 otu_15033 otu_52551 otu_140257
otu_118959 otu_157450 otu_123878 otu_138699 otu_116152 otu_145590 otu_19809
otu_90985 otu_91284 otu_89126 otu_44271 otu_1464 otu_59766 otu_160739 otu_97452
otu_18302 otu_92011 otu_58336 otu_12444 otu_116373 otu_58508 otu_138771
otu_162037 otu_149778 otu_98409 otu_53266 otu_117559 otu_157526 otu_142993
otu_141461 otu_57939 otu_138295 otu_137175 otu_11104 otu_136358 otu_148267
otu_88862 otu_1676 otu_117633 otu_12590 otu_55419 otu_18696 otu_160840 otu_48925
otu_129301 otu_89060 otu_67101 otu_116534 otu_47431 otu_97749 otu_104726
otu_52482 otu_62788 otu_49422 otu_105196 otu_48834 otu_16910 otu_100767
otu_118783 otu_51279 otu_11746 otu_10461 otu_47695 otu_133422 otu_59378
otu_138700 otu_46641 otu_122374 otu_90138 otu_44599 otu_60672 otu_135185
otu_129903 otu_135748 otu_136505 otu_16906 otu_11507 otu_13471 otu_48187
otu_135450 otu_98692 otu_19050 otu_19785 otu_49412 otu_9771 otu_18444 otu_92095
otu_102070 otu_116898 otu_268 otu_62446 otu_16556 otu_61760 otu_59078 otu_157073
otu_94752 otu_147867 otu_97716 otu_1826 otu_120530 otu_46950 otu_160143
otu_60158 otu_58889 otu_67974 otu_129747 otu_149051 otu_119710 otu_91199
otu_17488 otu_44795 otu_44899 otu_121316 otu_21266 otu_136292 otu_16260
otu_148163 otu_138851 otu_20918 otu_57898 otu_64615 otu_20346 otu_9212
otu_157717 otu_66751 otu_61337 otu_120152 otu_129848 otu_147856 otu_53183
otu_86988 otu_88954 otu_144840 otu_58445 otu_90037 otu_149414 otu_17760

[illegible]
otu_118783 otu_51279 otu_11746 otu_10461 otu_47695 otu_133422 otu_59378
otu_138700 otu_46641 otu_122374 otu_90138 otu_44599 otu_60672 otu_135185
otu_129903 otu_135748 otu_136505 otu_16906 otu_11507 otu_13471 otu_48187
otu_135450 otu_98692 otu_19050 otu_19785 otu_49412 otu_9771 otu_18444 otu_92095
otu_102070 otu_116898 otu_268 otu_62446 otu_16556 otu_61760 otu_59078 otu_157073
otu_94752 otu_147867 otu_97716 otu_1826 otu_120530 otu_46950 otu_160143
otu_60158 otu_58889 otu_67974 otu_129747 otu_149051 otu_119710 otu_91199
otu_17488 otu_44795 otu_44899 otu_121316 otu_21266 otu_136292 otu_16260
otu_148163 otu_138851 otu_20918 otu_57898 otu_64615 otu_20346 otu_9212
otu_157717 otu_66751 otu_61337 otu_120152 otu_129848 otu_147856 otu_53183
otu_86988 otu_88954 otu_144840 otu_58445 otu_90037 otu_149414 otu_17760
otu_86787 otu_47712 otu_11608 otu_58098 otu_148000 otu_161467 otu_11240 otu_59412
otu_10594 otu_119687 otu_160047 otu_60239 otu_158016 otu_160340 otu_49650
otu_93923 otu_44709 otu_90788 otu_134112 otu_141394 otu_55218 otu_16441
otu_136573 otu_86195 otu_63180 otu_91935 otu_141708 otu_9578 otu_59388
otu_163847 otu_101861 otu_134013 otu_87590 otu_10575 otu_143176 otu_149232
otu_140097 otu_9762 otu_130626 otu_100757 otu_118748 otu_89249 otu_86180
otu_115691 otu_1077 otu_61468 otu_51578 otu_157820 otu_115799 otu_129432
otu_61479 otu_51678 otu_19084 otu_145360 otu_137015 otu_11313 otu_11331
otu_119289 otu_141677 otu_1963 otu_1120 otu_53900 otu_54544 otu_20450 otu_121278
otu_101661 otu_46343 otu_158004 otu_64782 otu_86321 otu_104198 otu_117038
otu_138725 otu_119313 otu_145528 otu_58968 otu_101180 otu_118453 otu_58629
otu_91179 otu_91321 otu_161042 otu_92308 otu_120398 otu_59199 otu_133864
otu_15612 otu_54214 otu_115909 otu_62372 otu_120423 otu_100751 otu_64423
otu_116739 otu_101461 otu_47388 otu_61741 otu_90529 otu_19439 otu_20326
otu_120323 otu_119443 otu_139458 otu_20745 otu_118894 otu_88320 otu_117344
otu_46438 otu_47942 otu_163971 otu_13125 otu_92527 otu_120457 otu_138876
otu_10370 otu_131860 otu_117161 otu_93628 otu_115840 otu_156855 otu_147945
otu_9520 otu_57340 otu_120603 otu_60099 otu_116631 otu_11014 otu_104949 otu_50059
otu_91749 otu_140338 otu_21578 otu_55118 otu_164037 otu_20233 otu_64408
otu_18734 otu_117069 otu_135140 otu_122311 otu_133621 otu_11645 otu_162254
otu_57209 otu_161432 otu_48157 otu_59483 otu_140103 otu_19682 otu_57820
otu_14745 otu_120586 otu_131327 otu_9330 otu_9246 otu_102225 otu_135627
otu_130337 otu_87490 otu_138366 otu_53152 otu_159261 otu_131039 otu_97853
otu_47285 otu_97571 otu_159411 otu_12337 otu_131126 otu_60820 otu_141549 otu_1837
otu_9949 otu_141546 otu_48089 otu_143333 otu_55285 otu_13535 otu_137404
otu_161923 otu_58102 otu_123042 otu_157468 otu_141349 otu_130647 otu_119003
otu_49853 otu_47133 otu_119782 otu_119800 otu_16701 otu_92631 otu_115708
otu_160408 otu_49337 otu_164033 otu_45097 otu_157273 otu_61051 otu_140138
otu_97934 otu_52464 otu_44805 otu_57734 otu_15253 otu_91780 otu_46645 otu_115629
otu_143047 otu_68083 otu_101071 otu_1029 otu_12894 otu_92008 otu_52784 otu_12756
otu_117005 otu_130284 otu_122254 otu_13155 otu_9694 otu_66524 otu_98799
otu_149428 otu_50365 otu_162495 otu_134960 otu_104701 otu_57879 otu_101708
otu_61457 otu_129933 otu_50916 otu_100662 otu_16546 otu_13256 otu_121861
otu_92758 otu_159764 otu_9347 otu_101919 otu_119802 otu_11527 otu_88207 otu_1370
otu_148239 otu_283 otu_65509 otu_87915 otu_17172 otu_156806 otu_119738 otu_1912
otu_45466 otu_148376 otu_120176 otu_118818 otu_47152 otu_118736 otu_44379
otu_12351 otu_14647 otu_157035 otu_56194 otu_58605 otu_89387 otu_133440
otu_130661 otu_149615 otu_120303 otu_59326 otu_12502 otu_116766 otu_18005
otu_159847 otu_104162 otu_19095 otu_122594 otu_10150 otu_21256 otu_119479
otu_48590 otu_53999 otu_44609 otu_118928 otu_13207 otu_86764 otu_87290 otu_9223
otu_11291 otu_164071 otu_101874 otu_94121 otu_61453 otu_44305 otu_140480
otu_58540 otu_131004 otu_143119 otu_135364 otu_118874 otu_89691 otu_18961
otu_161734 otu_160969 otu_53226 otu_46432 otu_120825 otu_143362 otu_134897
otu_50615 otu_91104 otu_63001 otu_13173 otu_48032 otu_55047 otu_160795 otu_21479
otu_50966 otu_1406 otu_57779 otu_100509 otu_10637 otu_19674 otu_148260
otu_148203 otu_66729 otu_141340 otu_51306 otu_11771 otu_100983 otu_45519
otu_145475 otu_156733 otu_53959 otu_156832 otu_100869 otu_9316 otu_121823
otu_19357 otu_158613 otu_10536 otu_145103 otu_89400 otu_18409 otu_164110
otu_91167 otu_136302 otu_117023 otu_56285 otu_93422 otu_57645 otu_59785 otu_60366
otu_18544 otu_100837 otu_143033 otu_91814 otu_19008 otu_58575 otu_10648 otu_89
otu_89791 otu_105305 otu_140407 otu_17353 otu_116313 otu_157161 otu_91421
otu_47674 otu_48317 otu_129337 otu_104569 otu_140458 otu_157472 otu_92669
otu_48659 otu_157942 otu_45700 otu_44522 otu_65615 otu_53435 otu_44343
otu_118221 otu_148384 otu_1538 otu_10118 otu_9661 otu_115731 otu_134987 otu_97727
otu_53716 otu_148196 otu_44391 otu_102317 otu_122076 otu_149498 otu_119959
otu_92312 otu_102091 otu_130890 otu_156692 otu_145521 otu_92499 otu_47102
otu_116381 otu_59582 otu_140377 otu_48288 otu_88133 otu_44284 otu_97625
otu_139470 otu_13705 otu_149190 otu_148422 otu_53682 otu_21381 otu_89302
otu_52341 otu_13926 otu_61519 otu_47617 otu_121888 otu_104433 otu_129460
otu_104288 otu_117108 otu_89841 otu_56012 otu_121145 otu_18721 otu_55523
otu_115900 otu_104793 otu_1369 otu_130837 otu_16649 otu_10009 otu_140537
otu_59886 otu_97888 otu_104385 otu_142985 otu_86751 otu_135834 otu_18710
otu_58417 otu_16691 otu_49709 otu_18390 otu_140521 otu_130803 otu_10095
otu_129721 otu_104280 otu_135722 otu_9639 otu_122373 otu_134055 otu_129407
otu_14694 otu_1531 otu_92236 otu_68315 otu_63829 otu_160255 otu_88614 otu_14463
otu_130310 otu_46356 otu_141483 otu_48561 otu_156993 otu_92520 otu_122534
otu_16869 otu_20049 otu_145118 otu_136357 otu_46667 otu_131320 otu_86529
otu_119490 otu_16767 otu_147968 otu_87968 otu_91724 otu_121424 otu_158265
otu_141372 otu_47352 otu_59747 otu_53157 otu_129880 otu_149629 otu_149229
otu_18757 otu_11220 otu_44632 otu_48495 otu_55886 otu_98819 otu_157085
otu_133337 otu_61943 otu_121982 otu_58784 otu_11231 otu_64120 otu_10178
otu_120678 otu_119298 otu_137568 otu_129427 otu_97799 otu_46056 otu_52246
otu_136347 otu_87990 otu_49834 otu_149701 otu_121697 otu_94150 otu_13761
otu_59494 otu_91502 otu_117961 otu_121097 otu_139426 otu_120800 otu_101518
otu_135081 otu_20322 otu_50206 otu_149278 otu_11893 otu_136274 otu_119358
otu_133352 otu_9916 otu_100688 otu_45089 otu_51159 otu_121803 otu_16580
otu_16520 otu_145197 otu_117522 otu_163538 otu_116910 otu_102023 otu_159937
otu_120476 otu_48067 otu_58639 otu_130791 otu_16961 otu_157617 otu_56328
otu_92010 otu_160151 otu_1736 otu_10382 otu_141312 otu_160458 otu_56931
otu_101902 otu_44332 otu_129682 otu_137736 otu_97835 otu_137162 otu_62982
otu_87367 otu_120902 otu_137002 otu_19826 otu_100857 otu_20408 otu_45323
otu_131139 otu_16477 otu_90610 otu_104961 otu_159833 otu_10045 otu_13321
otu_91130 otu_148158 otu_52032 otu_118367 otu_87414 otu_90968 otu_118809
otu_163951 otu_137233 otu_119316 otu_60246 otu_122422 otu_55166 otu_163850
otu_97766 otu_157132 otu_47408 otu_97953 otu_102488 otu_88780 otu_90811
otu_86240 otu_131382 otu_86828 otu_115925 otu_63629 otu_135298 otu_15520
otu_9352 otu_121294 otu_135259 otu_9803 otu_64776 otu_13150 otu_145411
otu_162438 otu_160536 otu_102110 otu_50819 otu_148291 otu_140562 otu_120840
otu_97648 otu_46721 otu_163673 otu_52889 otu_121897 otu_117483 otu_62775
otu_10132 otu_158090 otu_53732 otu_133361 otu_56494 otu_45503 otu_121226
otu_21386 otu_98958 otu_14157 otu_162530 otu_89223 otu_9539 otu_13077 otu_18935
otu_54138 otu_17556 otu_141287 otu_61113 otu_148758 otu_148097 otu_101696
otu_52392 otu_149380 otu_57726 otu_57385 otu_116102 otu_156712 otu_149553
otu_21544 otu_105232 otu_94725 otu_158873 otu_87284 otu_131156 otu_17210
otu_62095 otu_118821 otu_14461 otu_122889 otu_59495 otu_121506 otu_105249
otu_45225 otu_88512 otu_10060 otu_87236 otu_16829 otu_22019 otu_137683
otu_129551 otu_118923 otu_149371 otu_90945 otu_101451 otu_49233 otu_136623

otu_9352 otu_121294 otu_135259 otu_9803 otu_64776 otu_13150 otu_145411
otu_162438 otu_160536 otu_102110 otu_50819 otu_148291 otu_140562 otu_120840
otu_97648 otu_46721 otu_163673 otu_52889 otu_121897 otu_117483 otu_62775
otu_10132 otu_158090 otu_53732 otu_133361 otu_56494 otu_45503 otu_121226
otu_21386 otu_98958 otu_14157 otu_162530 otu_89223 otu_9539 otu_13077 otu_18935
otu_54138 otu_17556 otu_141287 otu_61113 otu_148758 otu_148097 otu_101696
otu_52392 otu_149380 otu_57726 otu_57385 otu_116102 otu_156712 otu_149553
otu_21544 otu_105232 otu_94725 otu_158873 otu_87284 otu_131156 otu_17210
otu_62095 otu_118821 otu_14461 otu_122889 otu_59495 otu_121506 otu_105249
otu_45225 otu_88512 otu_10060 otu_87236 otu_16829 otu_22019 otu_137683
otu_129551 otu_118923 otu_149371 otu_90945 otu_101451 otu_49233 otu_136623
otu_58683 otu_121459 otu_91358 otu_133952 otu_138875 otu_148338 otu_158439
otu_63403 otu_88118 otu_89467 otu_143334 otu_53354 otu_115656 otu_161108
otu_18092 otu_160181 otu_119750 otu_148869 otu_16311 otu_60516 otu_10957
otu_141692 otu_57141 otu_11481 otu_121859 otu_119379 otu_148385 otu_160573
otu_58830 otu_18149 otu_130210 otu_89300 otu_141646 otu_46054 otu_104387
otu_18753 otu_15361 otu_20015 otu_49410 otu_12144 otu_133784 otu_143470
otu_117448 otu_156841 otu_9582 otu_100843 otu_92887 otu_148069 otu_9426 otu_51811
otu_11072 otu_18130 otu_54920 otu_100994 otu_10555 otu_90545 otu_50339
otu_121821 otu_115883 otu_87876 otu_136229 otu_89136 otu_101022 otu_89973
otu_157961 otu_11583 otu_92247 otu_88816 otu_18803 otu_116372 otu_163869
otu_104858 otu_50975 otu_49496 otu_87035 otu_49873 otu_136300 otu_48300
otu_47502 otu_140204 otu_17312 otu_136344 otu_95282 otu_89753 otu_52308
otu_101998 otu_91328 otu_147940 otu_1157 otu_16951 otu_104837 otu_90984
otu_57504 otu_16218 otu_104238 otu_91767 otu_90854 otu_68173 otu_87616 otu_97881
otu_53836 otu_157999 otu_148250 otu_122115 otu_60870 otu_121828 otu_56303
otu_18119 otu_115577 otu_137701 otu_120602 otu_16783 otu_135025 otu_122951
otu_13044 otu_48118 otu_11338 otu_104265 otu_46454 otu_148622 otu_118246
otu_11662 otu_60660 otu_137949 otu_149230 otu_141278 otu_115796 otu_148336
otu_10281 otu_11422 otu_14982 otu_59299 otu_120685 otu_9554 otu_57586 otu_140564
otu_145057 otu_87178 otu_47810 otu_98299 otu_148903 otu_115765 otu_16454
otu_10986 otu_91168 otu_14488 otu_130528 otu_87306 otu_57629 otu_52941
otu_148674 otu_45557 otu_11303 otu_157844 otu_149817 otu_57685 otu_46342
otu_45130 otu_134993 otu_138289 otu_11128 otu_101737 otu_116320 otu_145005
otu_122339 otu_104421 otu_157554 otu_49217 otu_137669 otu_44666 otu_49079
otu_140605 otu_59579 otu_159747 otu_44369 otu_140176 otu_90198 otu_57426
otu_161353 otu_17565 otu_92609 otu_140378 otu_100976 otu_19011 otu_91242
otu_86652 otu_86781 otu_13459 otu_133461 otu_147988 otu_59234 otu_60282
otu_122193 otu_147883 otu_58827 otu_86353 otu_16417 otu_55256 otu_52285
otu_159884 otu_92791 otu_148298 otu_93527 otu_16902 otu_92356 otu_161582
otu_98753 otu_97723 otu_10615 otu_120621 otu_1625 otu_18750 otu_49860 otu_164215
otu_141320 otu_19090 otu_16914 otu_104757 otu_61412 otu_141343 otu_117971
otu_140327 otu_44274 otu_118663 otu_148160 otu_89396 otu_139316 otu_66564
otu_46587 otu_59207 otu_53592 otu_1074 otu_129285 otu_11276 otu_17696 otu_94445
otu_130676 otu_58204 otu_133398 otu_52247 otu_86680 otu_98965 otu_13372
otu_51569 otu_88145 otu_158178 otu_129483 otu_88321 otu_44723 otu_13800
otu_98471 otu_51137 otu_45489 otu_57358 otu_88680 otu_9215 otu_10349 otu_94039
otu_159943 otu_158338 otu_9893 otu_51915 otu_163561 otu_130468 otu_45192
otu_137743 otu_52627 otu_57984 otu_141620 otu_47366 otu_160622 otu_116857
otu_140293 otu_55360 otu_16779 otu_9306 otu_10411 otu_19082 otu_86888 otu_46672
otu_59508 otu_49378 otu_102687 otu_157019 otu_93876 otu_135044 otu_66171
otu_121342 otu_17520 otu_149053 otu_62914 otu_135248 otu_56829 otu_62610
otu_86590 otu_58765 otu_16437 otu_65775 otu_57030 otu_143195 otu_61752
otu_136805 otu_131861 otu_10983 otu_59187 otu_86917 otu_17739 otu_87890
otu_104343 otu_64665 otu_148500 otu_101915 otu_118320 otu_148285 otu_91335
otu_161922 otu_56491 otu_100761 otu_160685 otu_104450 otu_149936 otu_9763
otu_10163 otu_58119 otu_10146 otu_49414 otu_86544 otu_133360 otu_13097 otu_56198
otu_160074 otu_141274 otu_89436 otu_13255 otu_90739 otu_102122 otu_59192
otu_50159 otu_53127 otu_149977 otu_149601 otu_88164 otu_161044 otu_11301
otu_90640 otu_44653 otu_138225 otu_120477 otu_55573 otu_48779 otu_16880
otu_16839 otu_139312 otu_88092 otu_44378 otu_131329 otu_94562 otu_9947 otu_67795
otu_159859 otu_120002 otu_14232 otu_86551 otu_115891 otu_67928 otu_119047
otu_129847 otu_47093 otu_46772 otu_164065 otu_10441 otu_145044 otu_60061
otu_87301 otu_53156 otu_122276 otu_47453 otu_45931 otu_148213 otu_17865
otu_147857 otu_19938 otu_57558 otu_119796 otu_129391 otu_162560 otu_57634
otu_45526 otu_102192 otu_140172 otu_90688 otu_139460 otu_1043 otu_18137
otu_117001 otu_159915 otu_56935 otu_136350 otu_131562 otu_137465 otu_48970
otu_98704 otu_12053 otu_93155 otu_87889 otu_58834 otu_141568 otu_67289 otu_17444
otu_49138 otu_17307 otu_47509 otu_17102 otu_58011 otu_18112 otu_53731 otu_18275
otu_1222 otu_97612 otu_117525 otu_101393 otu_89483 otu_21020 otu_57713 otu_92019
otu_131019 otu_46254 otu_138713 otu_140396 otu_91807 otu_116008 otu_118976
otu_57339 otu_16791 otu_158898 otu_1913 otu_87595 otu_100544 otu_87790 otu_58085
otu_161186 otu_60992 otu_92461 otu_148149 otu_129424 otu_60381 otu_118862
otu_18233 otu_118963 otu_116583 otu_19813 otu_149207 otu_121442 otu_46605
otu_105106 otu_104369 otu_60569 otu_162206 otu_148419 otu_10030 otu_86923
otu_49518 otu_11956 otu_97620 otu_100641 otu_59787 otu_44796 otu_149199
otu_10091 otu_13419 otu_140578 otu_47153 otu_149571 otu_13531 otu_145357
otu_148281 otu_87529 otu_86404 otu_57919 otu_156816 otu_118730 otu_17106
otu_143085 otu_90920 otu_143434 otu_44327 otu_92929 otu_68230 otu_60624
otu_44713 otu_140381 otu_45310 otu_57467 otu_138049 otu_9995 otu_86217 otu_88890
otu_20181 otu_50284 otu_130092 otu_62458 otu_119408 otu_157074 otu_11881
otu_121417 otu_21814 otu_104285 otu_143543 otu_20533 otu_86161 otu_130681
otu_47728 otu_157607 otu_18655 otu_98415 otu_45371 otu_44771 otu_59521 otu_10251
otu_131599 otu_1977 otu_141353 otu_91364 otu_9277 otu_148409 otu_140226
otu_116779 otu_140095 otu_130176 otu_95422 otu_55599 otu_58398 otu_17921
otu_60090 otu_86412 otu_157253 otu_12709 otu_47333 otu_58494 otu_145448
otu_158938 otu_9229 otu_49749 otu_88299 otu_53662 otu_133412 otu_55898 otu_16634
otu_143538 otu_157545 otu_45043 otu_159278 otu_123147 otu_10318 otu_12429
otu_60227 otu_129314 otu_118089 otu_87371 otu_130486 otu_66096 otu_122533
otu_121962 otu_118857 otu_48146 otu_16629 otu_50755 otu_44610 otu_104995
otu_90339 otu_94925 otu_119073 otu_97879 otu_93921 otu_98423 otu_11848 otu_160588
otu_158018 otu_17242 otu_61626 otu_57811 otu_86891 otu_92459 otu_61021
otu_158251 otu_163570 otu_123103 otu_54763 otu_130728 otu_148589 otu_137428
otu_156775 otu_16442 otu_46212 otu_53275 otu_104811 otu_45658 otu_118675
otu_13365 otu_45471 otu_139309 otu_15061 otu_89406 otu_57530 otu_57351 otu_51755
otu_58525 otu_45408 otu_138679 otu_60896 otu_16503 otu_159774 otu_98026
otu_137670 otu_91503 otu_140427 otu_45051 otu_137397 otu_59670 otu_161752
otu_129510 otu_157530 otu_104217 otu_156541 otu_100833 otu_89702 otu_53030
otu_137940 otu_100882 otu_55260 otu_86806 otu_10825 otu_135629 otu_68310
otu_89146 otu_158601 otu_9448 otu_54706 otu_48338 otu_163930 otu_91947 otu_61951
otu_145410 otu_57799 otu_102602 otu_91314 otu_16273 otu_158419 otu_118139
otu_18876 otu_17340 otu_53918 otu_68148 otu_52064 otu_9648 otu_100949 otu_133626
otu_92396 otu_49328 otu_12036 otu_149463 otu_16709 otu_9629 otu_90718 otu_86265
otu_121335 otu_160096 otu_119602 otu_117326 otu_44394 otu_161453 otu_1677
otu_120958 otu_55755 otu_92141 otu_140420 otu_10908 otu_49253 otu_2036 otu_54394
otu_45702 otu_49645 otu_116115 otu_140324 otu_137031 otu_19338 otu_97490
otu_143006 otu_49015 otu_143199 otu_51055 otu_100955 otu_62649 otu_86185
otu_9709 otu_141480 otu_160357 otu_12153 otu_136796 otu_87382 otu_62415
otu_51347 otu_51186 otu_116964 otu_156980 otu_11393 otu_19609 otu_58282

otu_129510 otu_157530 otu_104217 otu_156541 otu_100833 otu_89702 otu_53030
otu_137940 otu_100882 otu_55260 otu_86806 otu_10825 otu_135629 otu_68310
otu_89146 otu_158601 otu_9448 otu_54706 otu_48338 otu_163930 otu_91947 otu_61951
otu_145410 otu_57799 otu_102602 otu_91314 otu_16273 otu_158419 otu_118139
otu_18876 otu_17340 otu_53918 otu_68148 otu_52064 otu_9648 otu_100949 otu_133626
otu_92396 otu_49328 otu_12036 otu_149463 otu_16709 otu_9629 otu_90718 otu_86265
otu_121335 otu_160096 otu_119602 otu_117326 otu_44394 otu_161453 otu_1677
otu_120958 otu_55755 otu_92141 otu_140420 otu_10908 otu_49253 otu_2036 otu_54394
otu_45702 otu_49645 otu_116115 otu_140324 otu_137031 otu_19338 otu_97490
otu_143006 otu_49015 otu_143199 otu_51055 otu_100955 otu_62649 otu_86185
otu_9709 otu_141480 otu_160357 otu_12153 otu_136796 otu_87382 otu_62415
otu_51347 otu_51186 otu_116964 otu_156980 otu_11393 otu_19609 otu_58282
otu_144857 otu_115681 otu_86554 otu_94585 otu_90684 otu_66747 otu_90733
otu_89865 otu_19036 otu_121619 otu_130367 otu_148527 otu_91417 otu_54621
otu_115901 otu_49202 otu_100822 otu_48840 otu_57708 otu_61043 otu_143090
otu_102400 otu_102278 otu_140279 otu_19076 otu_55801 otu_91496 otu_160545
otu_97693 otu_149648 otu_158339 otu_47836 otu_17077 otu_138052 otu_115772
otu_46694 otu_56384 otu_57957 otu_67979 otu_115966 otu_119321 otu_143331
otu_88157 otu_138258 otu_138892 otu_136236 otu_54805 otu_9672 otu_131573
otu_159011 otu_15090 otu_51581 otu_1386 otu_10764 otu_148188 otu_14718 otu_64183
otu_149488 otu_118600 otu_98258 otu_105065 otu_11982 otu_14472 otu_45282
otu_161216 otu_20421 otu_86442 otu_48577 otu_148240 otu_10785 otu_57516
otu_156715 otu_118650 otu_136900 otu_88072 otu_89061 otu_162077 otu_91250
otu_64454 otu_16476 otu_160775 otu_90653 otu_118832 otu_149292 otu_46860
otu_86140 otu_87021 otu_131192 otu_160163 otu_156607 otu_135147 otu_157321
otu_143072 otu_160141 otu_104200 otu_50765 otu_91145 otu_148359 otu_63761
otu_116323 otu_61304 otu_88972 otu_53473 otu_11630 otu_164083 otu_158781
otu_160572 otu_9591 otu_130213 otu_56712 otu_121065 otu_129330 otu_66376
otu_16231 otu_87630 otu_53089 otu_57536 otu_58874 otu_97577 otu_52120 otu_120169
otu_118643 otu_145617 otu_119366 otu_102438 otu_148780 otu_54287 otu_119763
otu_129752 otu_90135 otu_87769 otu_119628 otu_46613 otu_91854 otu_141360
otu_118794 otu_89330 otu_60344 otu_13985 otu_19304 otu_47327 otu_60245
otu_160476 otu_55022 otu_136844 otu_46841 otu_10947 otu_5 otu_53622 otu_139349
otu_11086 otu_1791 otu_62130 otu_58182 otu_65583 otu_18031 otu_105268 otu_98451
otu_54686 otu_10374 otu_52014 otu_2012 otu_48522 otu_59759 otu_9856 otu_104293
otu_117856 otu_12510 otu_49299 otu_105103 otu_53416 otu_158247 otu_105184
otu_47531 otu_12601 otu_57934 otu_19706 otu_91327 otu_118746 otu_91191 otu_147959
otu_58156 otu_119893 otu_57361 otu_158786 otu_57455 otu_13830 otu_47691
otu_10863 otu_115782 otu_104729 otu_115791 otu_157067 otu_17319 otu_64824
otu_45187 otu_148060 otu_92390 otu_157996 otu_122225 otu_119978 otu_117022
otu_93339 otu_88055 otu_1057 otu_17512 otu_101557 otu_159890 otu_51040
otu_143346 otu_98519 otu_158473 otu_116312 otu_88400 otu_116691 otu_11674
otu_13140 otu_86628 otu_45027 otu_63652 otu_160236 otu_48139 otu_160432
otu_104628 otu_9214 otu_91818 otu_101139 otu_102511 otu_86329 otu_91662
otu_134149 otu_12235 otu_149347 otu_90933 otu_90521 otu_145062 otu_44258
otu_133424 otu_44571 otu_93159 otu_90495 otu_9371 otu_116281 otu_91171 otu_60668
otu_21205 otu_119340 otu_133877 otu_13996 otu_14483 otu_118495 otu_15903
otu_63170 otu_66144 otu_86632 otu_136241 otu_137469 otu_47522 otu_94602
otu_119207 otu_306 otu_122090 otu_157979 otu_157480 otu_51075 otu_133418
otu_145288 otu_61416 otu_129310 otu_87261 otu_12439 otu_157020 otu_10300
otu_137416 otu_118937 otu_104966 otu_1735 otu_16970 otu_136618 otu_139450
otu_45578 otu_163943 otu_149201 otu_11208 otu_57922 otu_104950 otu_133586
otu_86489 otu_61502 otu_18677 otu_160757 otu_87458 otu_53418 otu_157571
otu_130191 otu_9991 otu_145376 otu_158273 otu_117074 otu_19347 otu_100567
otu_63836 otu_148416 otu_148734 otu_156740 otu_94009 otu_158763 otu_88923
otu_119013 otu_138694 otu_133873 otu_51079 otu_89682 otu_60000 otu_47355
otu_98665 otu_87628 otu_55299 otu_63599 otu_100593 otu_90035 otu_1040 otu_129386
otu_58778 otu_87794 otu_90533 otu_49685 otu_12391 otu_116869 otu_57614 otu_49531
otu_158656 otu_130405 otu_47463 otu_60007 otu_160734 otu_50225 otu_117420
otu_135668 otu_45072 otu_12984 otu_134138 otu_138044 otu_101379 otu_55286
otu_86874 otu_94990 otu_90730 otu_53420 otu_94713 otu_129294 otu_89994 otu_98612
otu_117681 otu_45081 otu_86775 otu_102170 otu_98553 otu_93150 otu_16700
otu_160853 otu_122849 otu_86428 otu_136803 otu_11307 otu_141350 otu_45377
otu_137425 otu_101337 otu_52807 otu_116378 otu_149272 otu_91745 otu_90800
otu_10672 otu_51550 otu_65072 otu_140417 otu_117451 otu_302 otu_161732
otu_138835 otu_11945 otu_1299 otu_10242 otu_91174 otu_63495 otu_17364 otu_121671
otu_91584 otu_55011 otu_133932 otu_148565 otu_115779 otu_149184 otu_67161
otu_50320 otu_10508 otu_101095 otu_149342 otu_53709 otu_54755 otu_17409
otu_21253 otu_59301 otu_54205 otu_159420 otu_102109 otu_140190 otu_101546
otu_143126 otu_12761 otu_46636 otu_11283 otu_140644 otu_92123 otu_147995
otu_119608 otu_86922 otu_148305 otu_50299 otu_1887 otu_116874 otu_57562
otu_134255 otu_137675 otu_57527 otu_1136 otu_121588 otu_46394 otu_156600
otu_159771 otu_60987 otu_58708 otu_68748 otu_117821 otu_63585 otu_10998
otu_149427 otu_10138 otu_156707 otu_119562 otu_46744 otu_91876 otu_10706
otu_132242 otu_55329 otu_46309 otu_129638 otu_16184 otu_144862 otu_100786
otu_133803 otu_86374 otu_11251 otu_48844 otu_52616 otu_138026 otu_115916
otu_121112 otu_55414 otu_14206 otu_90192 otu_117078 otu_101041 otu_47724
otu_100526 otu_12126 otu_136364 otu_54535 otu_143349 otu_120797 otu_98569
otu_61912 otu_10229 otu_91754 otu_45272 otu_105040 otu_17248 otu_90626 otu_92265
otu_119053 otu_123282 otu_119686 otu_101952 otu_158344 otu_86504 otu_10608
otu_118180 otu_101196 otu_90427 otu_119285 otu_18173 otu_161909 otu_98366
otu_58907 otu_45220 otu_46661 otu_118632 otu_156812 otu_57311 otu_86832 otu_48100
otu_141604 otu_105254 otu_120224 otu_18376 otu_104814 otu_58572 otu_51181
otu_90565 otu_148307 otu_131480 otu_47551 otu_116256 otu_136024 otu_47372
otu_101150 otu_129787 otu_98413 otu_158382 otu_10404 otu_156588 otu_49863
otu_159116 otu_58692 otu_64040 otu_11873 otu_92513 otu_13115 otu_91221 otu_100495
otu_16518 otu_60112 otu_157781 otu_16803 otu_18773 otu_56770 otu_10545 otu_60436
otu_45632 otu_147923 otu_139336 otu_86408 otu_12681 otu_12790 otu_59610
otu_44718 otu_140096 otu_16672 otu_97527 otu_120349 otu_18398 otu_16557
otu_101031 otu_11236 otu_161095 otu_59332 otu_91405 otu_10567 otu_145219
otu_67152 otu_141489 otu_136808 otu_47946 otu_12550 otu_16278 otu_138231
otu_47387 otu_16815 otu_122243 otu_21460 otu_10256 otu_116939 otu_130684
otu_46996 otu_91638 otu_16357 otu_49488 otu_90847 otu_57476 otu_158811 otu_58896
otu_149593 otu_149918 otu_86545 otu_60078 otu_57658 otu_9948 otu_16610 otu_86340
otu_44440 otu_158020 otu_143533 otu_64279 otu_18994 otu_65354 otu_86204
otu_105144 otu_86431 otu_122385 otu_130183 otu_97701 otu_135891 otu_122113
otu_141296 otu_145363 otu_9962 otu_122499 otu_149484 otu_141346 otu_129575
otu_162861 otu_116853 otu_104982 otu_98927 otu_86934 otu_48280 otu_19796
otu_57969 otu_121236 otu_20041 otu_98035 otu_117311 otu_117089 otu_53875
otu_148247 otu_86178 otu_86644 otu_117226 otu_100912 otu_121542 otu_100894
otu_119933 otu_9488 otu_16659 otu_87350 otu_55092 otu_149198 otu_148275
otu_13821 otu_52431 otu_17673 otu_59454 otu_58017 otu_101192 otu_100530
otu_86672 otu_91546 otu_58493 otu_44400 otu_157565 otu_61213 otu_11232
otu_143549 otu_156864 otu_1495 otu_64993 otu_87114 otu_101296 otu_159515
otu_47390 otu_88942 otu_53632 otu_159978 otu_17510 otu_138680 otu_149336
otu_98190 otu_13585 otu_90190 otu_65416 otu_138736 otu_118978 otu_49400
otu_98788 otu_53762 otu_163654 otu_58980 otu_57121 otu_16696 otu_58234
otu_141290 otu_19651 otu_118356 otu_135474 otu_104579 otu_90364 otu_64076
otu_66385 otu_137137 otu_133783 otu_101826 otu_145082 otu_21340 otu_100721

otu_57969 otu_121236 otu_20041 otu_98035 otu_117311 otu_117089 otu_53875
otu_148247 otu_86178 otu_86644 otu_117226 otu_100912 otu_121542 otu_100894
otu_119933 otu_9488 otu_16659 otu_87350 otu_55092 otu_149198 otu_148275
otu_13821 otu_52431 otu_17673 otu_59454 otu_58017 otu_101192 otu_100530
otu_86672 otu_91546 otu_58493 otu_44400 otu_157565 otu_61213 otu_11232
otu_143549 otu_156864 otu_1495 otu_64993 otu_87114 otu_101296 otu_159515
otu_47390 otu_88942 otu_53632 otu_159978 otu_17510 otu_138680 otu_149336
otu_98190 otu_13585 otu_90190 otu_65416 otu_138736 otu_118978 otu_49400
otu_98788 otu_53762 otu_163654 otu_58980 otu_57121 otu_16696 otu_58234
otu_141290 otu_19651 otu_118356 otu_135474 otu_104579 otu_90364 otu_64076
otu_66385 otu_137137 otu_133783 otu_101826 otu_145082 otu_21340 otu_100721
otu_58878 otu_67137 otu_53104 otu_10430 otu_137476 otu_14885 otu_15166 otu_50195
otu_51277 otu_122338 otu_92645 otu_66690 otu_56472 otu_14109 otu_101030 otu_1527
otu_18525 otu_62363 otu_134198 otu_143405 otu_157268 otu_143666 otu_88773
otu_17802 otu_87205 otu_14195 otu_18477 otu_162390 otu_147878 otu_58973
otu_55102 otu_149492 otu_10905 otu_65059 otu_158889 otu_119213 otu_141323
otu_163587 otu_119309 otu_20244 otu_58695 otu_9323 otu_143439 otu_88159
otu_161962 otu_102356 otu_139408 otu_147890 otu_90873 otu_116775 otu_57594
otu_16203 otu_63228 otu_48381 otu_157534 otu_50391 otu_86211 otu_143356
otu_118791 otu_47486 otu_131793 otu_118721 otu_16997 otu_19149 otu_48980
otu_143102 otu_145034 otu_19187 otu_162431 otu_156995 otu_156668 otu_12328
otu_136251 otu_137531 otu_46815 otu_18231 otu_101969 otu_130785 otu_65321
otu_92053 otu_52562 otu_54165 otu_46550 otu_144885 otu_159323 otu_60792
otu_53873 otu_97916 otu_92045 otu_16305 otu_12124 otu_50964 otu_16255 otu_133331
otu_52248 otu_46057 otu_17430 otu_58766 otu_46213 otu_91294 otu_87185 otu_137948
otu_14918 otu_46902 otu_159744 otu_120502 otu_46504 otu_100515 otu_157799
otu_104763 otu_163491 otu_143327 otu_48614 otu_47682 otu_67010 otu_160335
otu_122931 otu_158671 otu_48044 otu_115809 otu_48091 otu_59761 otu_119726
otu_157509 otu_50783 otu_137043 otu_45473 otu_137513 otu_88253 otu_156792
otu_116181 otu_120019 otu_130578 otu_45333 otu_122515 otu_104222 otu_53869
otu_44330 otu_148136 otu_20607 otu_52294 otu_119015 otu_140159 otu_17047
otu_87869 otu_115910 otu_48071 otu_138685 otu_115699 otu_91378 otu_149600
otu_19831 otu_102173 otu_9314 otu_11843 otu_157092 otu_61893 otu_61027
otu_135409 otu_131577 otu_88171 otu_49393 otu_67575 otu_117547 otu_50071
otu_115830 otu_66894 otu_47977 otu_133699 otu_117299 otu_158362 otu_120751
otu_1713 otu_62515 otu_100900 otu_18304 otu_131986 otu_47405 otu_58930 otu_90851
otu_13339 otu_160887 otu_67189 otu_11061 otu_133561 otu_16985 otu_156622
otu_53085 otu_100590 otu_118647 otu_20100 otu_44659 otu_138841 otu_9886
otu_16578 otu_16221 otu_156695 otu_11106 otu_9853 otu_57532 otu_46025 otu_118845
otu_15303 otu_101162 otu_129684 otu_105081 otu_51723 otu_57643 otu_115819
otu_48282 otu_45167 otu_138098 otu_45017 otu_135835 otu_1776 otu_149618
otu_138974 otu_46682 otu_133763 otu_144836 otu_160185 otu_93987 otu_91538
otu_115710 otu_62895 otu_50735 otu_46643 otu_94173 otu_135662 otu_135055
otu_20527 otu_162131 otu_46912 otu_119985 otu_86170 otu_115576 otu_117568
otu_157487 otu_119457 otu_138692 otu_120572 otu_48510 otu_105055 otu_16479
otu_160091 otu_64813 otu_130560 otu_47538 otu_135655 otu_59888 otu_45133
otu_116415 otu_135481 otu_17098 otu_64673 otu_9873 otu_11285 otu_120519
otu_116000 otu_157121 otu_120406 otu_1999 otu_45493 otu_63820 otu_47422
otu_160721 otu_57656 otu_93930 otu_143490 otu_16535 otu_93323 otu_52882
otu_132265 otu_49302 otu_16647 otu_60060 otu_10654 otu_15461 otu_148764
otu_137473 otu_51476 otu_63169 otu_53215 otu_135938 otu_12468 otu_116913
otu_158952 otu_13504 otu_61418 otu_61376 otu_130521 otu_90843 otu_90581
otu_17513 otu_66235 otu_17368 otu_56178 otu_59666 otu_48279 otu_46222 otu_16411
otu_123085 otu_145120 otu_137447 otu_64849 otu_90559 otu_65296 otu_161805
otu_16320 otu_62358 otu_46407 otu_119217 otu_100930 otu_59298 otu_50379
otu_90509 otu_138911 otu_55089 otu_61977 otu_130986 otu_59147 otu_156926
otu_137541 otu_67623 otu_118064 otu_119082 otu_59542 otu_44543 otu_87471
otu_11312 otu_119386 otu_60536 otu_92373 otu_48222 otu_49524 otu_137962
otu_138059 otu_58557 otu_54838 otu_90114 otu_63453 otu_62769 otu_44772 otu_47585
otu_47348 otu_65620 otu_133478 otu_158189 otu_116173 otu_119269 otu_156553
otu_62331 otu_13808 otu_91709 otu_157657 otu_116532 otu_120140 otu_15498
otu_46316 otu_47302 otu_50010 otu_9918 otu_160397 otu_117049 otu_92209 otu_15156
otu_132178 otu_58992 otu_49089 otu_45446 otu_119348 otu_55163 otu_87617
otu_18344 otu_9768 otu_48461 otu_45409 otu_44761 otu_53673 otu_88765 otu_159467
otu_89530 otu_19739 otu_116707 otu_118085 otu_66076 otu_98117 otu_51664
otu_120069 otu_101470 otu_91670 otu_66584 otu_21232 otu_86536 otu_59741
otu_49942 otu_141364 otu_86502 otu_11740 otu_16968 otu_98509 otu_87624
otu_137001 otu_105166 otu_94874 otu_61785 otu_156860 otu_51927 otu_51572
otu_119753 otu_162136 otu_44488 otu_10971 otu_139456 otu_49238 otu_47292
otu_9825 otu_16421 otu_91388 otu_138927 otu_86690 otu_87400 otu_130657 otu_53646
otu_17552 otu_45075 otu_119442 otu_11749 otu_91251 otu_87103 otu_46651 otu_44834
otu_157055 otu_139371 otu_87312 otu_1861 otu_119427 otu_92879 otu_58659
otu_20136 otu_118989 otu_143223 otu_10480 otu_92305 otu_47510 otu_86642
otu_143115 otu_156559 otu_116090 otu_17576 otu_58680 otu_121079 otu_88558
otu_147953 otu_46157 otu_86518 otu_13228 otu_122199 otu_122832 otu_158976
otu_47209 otu_1039 otu_59938 otu_131842 otu_147920 otu_63444 otu_156823
otu_49445 otu_91257 otu_160636 otu_118625 otu_12074 otu_16590 otu_16338
otu_145329 otu_101175 otu_92154 otu_46235 otu_143075 otu_116016 otu_147908
otu_14211 otu_158421 otu_122528 otu_131058 otu_143025 otu_91846 otu_12410
otu_162563 otu_1131 otu_158853 otu_58653 otu_148282 otu_61675 otu_116781
otu_93526 otu_50140 otu_157917 otu_143194 otu_143381 otu_116452 otu_122859
otu_12284 otu_18995 otu_119499 otu_17870 otu_138818 otu_105270 otu_59884
otu_132279 otu_118835 otu_119546 otu_120625 otu_49002 otu_156619 otu_89878
otu_135371 otu_135644 otu_159716 otu_91054 otu_138273 otu_61112 otu_122233
otu_10897 otu_115919 otu_86254 otu_87098 otu_87641 otu_9285 otu_101073
otu_102093 otu_93403 otu_138224 otu_56171 otu_1109 otu_137572 otu_45553
otu_135129 otu_160257 otu_47154 otu_49583 otu_121297 otu_44699 otu_160778
otu_137964 otu_149280 otu_65570 otu_93563 otu_13349 otu_88236 otu_12844
otu_19496 otu_19064 otu_17528 otu_143469 otu_59193 otu_120150 otu_12936
otu_10554 otu_1665 otu_53526 otu_120291 otu_119179 otu_97644 otu_57987 otu_98254
otu_45842 otu_60526 otu_131124 otu_46715 otu_21038 otu_145177 otu_18389
otu_149411 otu_91323 otu_90862 otu_62756 otu_88086 otu_50276 otu_18553 otu_92251
otu_120864 otu_93549 otu_60331 otu_46917 otu_88043 otu_58615 otu_90622
otu_147871 otu_133717 otu_54712 otu_51720 otu_57515 otu_138990 otu_9845
otu_87276 otu_57807 otu_48490 otu_149467 otu_159876 otu_101883 otu_47094
otu_12691 otu_134044 otu_61350 otu_44297 otu_86494 otu_137141 otu_133987
otu_59017 otu_91391 otu_87167 otu_12597 otu_62393 otu_49108 otu_51564 otu_65507
otu_157309 otu_46929 otu_89979 otu_10808 otu_119996 otu_54431 otu_101183
otu_131709 otu_63813 otu_140421 otu_157919 otu_60108 otu_52043 otu_121978
otu_9646 otu_48442 otu_92859 otu_92886 otu_48547 otu_17796 otu_48151 otu_12145
otu_136504 otu_135285 otu_95277 otu_45066 otu_122313 otu_51252 otu_90596
otu_139447 otu_91761 otu_46751 otu_18538 otu_101306 otu_141407 otu_45903
otu_161164 otu_102424 otu_120465 otu_121951 otu_100472 otu_59755 otu_160564
otu_161799 otu_147866 otu_61983 otu_143031 otu_20235 otu_94222 otu_9437
otu_44224 otu_9320 otu_58699 otu_117829 otu_118765 otu_57211 otu_163804
otu_129245 otu_63030 otu_116259 otu_87420 otu_59813 otu_93883 otu_135254
otu_123350 otu_51177 otu_147974 otu_86459 otu_19284 otu_118840 otu_90755
otu_139419 otu_17699 otu_9637 otu_11051 otu_11464 otu_91604 otu_44531 otu_45206
otu_1643 otu_97474 otu_19881 otu_140479 otu_65287 otu_116341 otu_131300

otu_157309 otu_46929 otu_89979 otu_10808 otu_119996 otu_54431 otu_101183
otu_131709 otu_63813 otu_140421 otu_157919 otu_60108 otu_52043 otu_121978
otu_9646 otu_48442 otu_92859 otu_92886 otu_48547 otu_17796 otu_48151 otu_12145
otu_136504 otu_135285 otu_95277 otu_45066 otu_122313 otu_51252 otu_90596
otu_139447 otu_91761 otu_46751 otu_18538 otu_101306 otu_141407 otu_45903
otu_161164 otu_102424 otu_120465 otu_121951 otu_100472 otu_59755 otu_160564
otu_161799 otu_147866 otu_61983 otu_143031 otu_20235 otu_94222 otu_9437
otu_44224 otu_9320 otu_58699 otu_117829 otu_118765 otu_57211 otu_163804
otu_129245 otu_63030 otu_116259 otu_87420 otu_59813 otu_93883 otu_135254
otu_123350 otu_51177 otu_147974 otu_86459 otu_19284 otu_118840 otu_90755
otu_139419 otu_17699 otu_9637 otu_11051 otu_11464 otu_91604 otu_44531 otu_45206
otu_1643 otu_97474 otu_19881 otu_140479 otu_65287 otu_116341 otu_131300
otu_45474 otu_62617 otu_105102 otu_46601 otu_148112 otu_157940 otu_98154
otu_98667 otu_137437 otu_11736 otu_157474 otu_134958 otu_63772 otu_145442
otu_91269 otu_120591 otu_16724 otu_145244 otu_54244 otu_13720 otu_98466
otu_59093 otu_148009 otu_158363 otu_160117 otu_16200 otu_51041 otu_53262
otu_104431 otu_89148 otu_18324 otu_64927 otu_16937 otu_91106 otu_90899 otu_46075
otu_66605 otu_97661 otu_136918 otu_58005 otu_91190 otu_134277 otu_117300
otu_105304 otu_148816 otu_16632 otu_20371 otu_52217 otu_121511 otu_156547
otu_161811 otu_10832 otu_121589 otu_163540 otu_44890 otu_17767 otu_130186
otu_58782 otu_61044 otu_16816 otu_65558 otu_160290 otu_117083 otu_129936
otu_99013 otu_1347 otu_136383 otu_117227 otu_115744 otu_57004 otu_9608 otu_54521
otu_63844 otu_100630 otu_119075 otu_44715 otu_67015 otu_62539 otu_67250
otu_89154 otu_21952 otu_133401 otu_158582 otu_145424 otu_148667 otu_68052
otu_10893 otu_97743 otu_18678 otu_16302 otu_2002 otu_44781 otu_9559 otu_47546
otu_1658 otu_140208 otu_116549 otu_120682 otu_86753 otu_46019 otu_16446
otu_61461 otu_142982 otu_10022 otu_46787 otu_121023 otu_101545 otu_117713
otu_104625 otu_64377 otu_51978 otu_130759 otu_45347 otu_156924 otu_98047
otu_54368 otu_118517 otu_59604 otu_119218 otu_46357 otu_47558 otu_16412
otu_119656 otu_44282 otu_140570 otu_119159 otu_97832 otu_66018 otu_136922
otu_145266 otu_92479 otu_157502 otu_105130 otu_63081 otu_149517 otu_57679
otu_97877 otu_148011 otu_122091 otu_138236 otu_137153 otu_122901 otu_10243
otu_91988 otu_89159 otu_98615 otu_53207 otu_101422 otu_134261 otu_44525 otu_9663
otu_49519 otu_119966 otu_51778 otu_118952 otu_157093 otu_14675 otu_141733
otu_163695 otu_130246 otu_143277 otu_20652 otu_156560 otu_53054 otu_54586
otu_62329 otu_100465 otu_19158 otu_11289 otu_157520 otu_120116 otu_61645
otu_45703 otu_10817 otu_148105 otu_119982 otu_46312 otu_66398 otu_118397
otu_133357 otu_49370 otu_148595 otu_59400 otu_118148 otu_67673 otu_47684
otu_50579 otu_118300 otu_45573 otu_1137 otu_61950 otu_118751 otu_120403
otu_115689 otu_105211 otu_92359 otu_11863 otu_92853 otu_94411 otu_60745
otu_162167 otu_157187 otu_88365 otu_162551 otu_156713 otu_50685 otu_87111
otu_136395 otu_148545 otu_104731 otu_122513 otu_144839 otu_116552 otu_49327
otu_14556 otu_135268 otu_160785 otu_16536 otu_59104 otu_138769 otu_136349
otu_61945 otu_149892 otu_57896 otu_90886 otu_119928 otu_123785 otu_101579
otu_104486 otu_119128 otu_53159 otu_139439 otu_160677 otu_138971 otu_139354
otu_67917 otu_65060 otu_12134 otu_45730 otu_9777 otu_12248 otu_45352 otu_58999
otu_138706 otu_147851 otu_21204 otu_14886 otu_58461 otu_90572 otu_97860
otu_138012 otu_16247 otu_158267 otu_15012 otu_17005 otu_131322 otu_67538
otu_9903 otu_160512 otu_157715 otu_21191 otu_56088 otu_46884 otu_140249
otu_10673 otu_54190 otu_102359 otu_159129 otu_49958 otu_51599 otu_102573
otu_149029 otu_11983 otu_57537 otu_117848 otu_104411 otu_133366 otu_9279
otu_102149 otu_160728 otu_100656 otu_17477 otu_104560 otu_149432 otu_44593
otu_45319 otu_119605 otu_46764 otu_140433 otu_50220 otu_16183 otu_101199
otu_15329 otu_61583 otu_57772 otu_66571 otu_64337 otu_98294 otu_59033 otu_116472
otu_1081 otu_158234 otu_93942 otu_53986 otu_120161 otu_19856 otu_120081
otu_116134 otu_45758 otu_158537 otu_140482 otu_145338 otu_94856 otu_87906
otu_16359 otu_138217 otu_50780 otu_46617 otu_149401 otu_58372 otu_13402
otu_19056 otu_87527 otu_157746 otu_92732 otu_145469 otu_149241 otu_50209
otu_162568 otu_160675 otu_94833 otu_100806 otu_11442 otu_119617 otu_10981
otu_9265 otu_57782 otu_94084 otu_59893 otu_9502 otu_122589 otu_91211 otu_120662
otu_138750 otu_156540 otu_119117 otu_46565 otu_148156 otu_131796 otu_45047
otu_105085 otu_53264 otu_119244 otu_90261 otu_91482 otu_16267 otu_93903
otu_123423 otu_46298 otu_149049 otu_157232 otu_53061 otu_93750 otu_20313
otu_93691 otu_131619 otu_157042 otu_9375 otu_160639 otu_10377 otu_12915
otu_129303 otu_56670 otu_88003 otu_1051 otu_149251 otu_62071 otu_135793 otu_11118
otu_46540 otu_44350 otu_143350 otu_162436 otu_160653 otu_120413 otu_134936
otu_129530 otu_138710 otu_118653 otu_86679 otu_94976 otu_13544 otu_9740
otu_89905 otu_91541 otu_15924 otu_160630 otu_46021 otu_47373 otu_115667
otu_160706 otu_48388 otu_104590 otu_160045 otu_86222 otu_17692 otu_129740
otu_118012 otu_98917 otu_45567 otu_12748 otu_18126 otu_11781 otu_118781 otu_86967
otu_14258 otu_9302 otu_131999 otu_104755 otu_118211 otu_45913 otu_61901 otu_64448
otu_48421 otu_88854 otu_105072 otu_1864 otu_143084 otu_44728 otu_131008
otu_14013 otu_288 otu_157635 otu_119176 otu_139429 otu_12011 otu_137606 otu_20940
otu_104963 otu_156796 otu_141282 otu_45364 otu_47399 otu_60329 otu_62118
otu_61624 otu_140526 otu_148161 otu_97504 otu_59142 otu_45577 otu_44511 otu_11209
otu_67145 otu_140077 otu_13916 otu_86612 otu_137144 otu_94609 otu_118899
otu_46995 otu_91497 otu_90917 otu_9329 otu_11668 otu_104175 otu_18586 otu_143651
otu_143156 otu_59175 otu_11631 otu_138036 otu_14526 otu_100568 otu_51718
otu_131014 otu_92740 otu_135641 otu_143588 otu_10308 otu_91034 otu_90050
otu_137410 otu_149475 otu_89190 otu_12551 otu_59270 otu_157558 otu_59390
otu_60288 otu_101964 otu_140122 otu_88426 otu_16367 otu_61658 otu_2013 otu_53725
otu_12741 otu_116573 otu_157071 otu_138751 otu_148314 otu_90991 otu_133477
otu_44253 otu_1190 otu_59851 otu_88819 otu_61480 otu_1417 otu_1961 otu_145390
otu_148518 otu_161826 otu_15873 otu_87282 otu_45125 otu_141297 otu_53509
otu_12837 otu_17163 otu_118820 otu_1914 otu_116920 otu_104475 otu_20011
otu_123516 otu_14735 otu_159580 otu_137179 otu_17279 otu_17289 otu_158443
otu_161025 otu_61146 otu_149298 otu_102089 otu_63040 otu_104981 otu_59052
otu_86215 otu_119439 otu_55148 otu_9440 otu_62469 otu_51008 otu_53919 otu_148705
otu_121311 otu_46411 otu_90593 otu_129484 otu_158824 otu_52790 otu_130510
otu_117062 otu_90842 otu_130055 otu_59625 otu_139482 otu_11411 otu_51376
otu_47786 otu_130888 otu_147970 otu_119428 otu_61492 otu_64163 otu_156857
otu_65067 otu_87598 otu_98671 otu_17290 otu_129293 otu_118194 otu_1601
otu_158292 otu_87089 otu_88410 otu_11227 otu_47828 otu_158764 otu_140343
otu_46160 otu_45231 otu_10923 otu_49341 otu_88755 otu_87854 otu_159827 otu_16435
otu_159190 otu_63642 otu_52662 otu_10653 otu_12219 otu_133984 otu_160923
otu_65795 otu_137170 otu_45093 otu_90629 otu_148237 otu_136310 otu_117412
otu_93889 otu_130322 otu_86390 otu_11282 otu_16343 otu_134281 otu_44296
otu_119736 otu_121068 otu_86588 otu_119862 otu_117865 otu_148091 otu_54225
otu_120301 otu_17614 otu_19501 otu_61317 otu_93304 otu_19597 otu_149660
otu_138223 otu_156629 otu_92073 otu_12500 otu_21349 otu_59258 otu_16263
otu_105034 otu_87145 otu_120068 otu_101094 otu_58517 otu_87041 otu_102376
otu_19021 otu_161884 otu_92707 otu_46247 otu_149546 otu_116889 otu_17491
otu_13109 otu_56028 otu_93972 otu_13293 otu_121991 otu_129757 otu_17898
otu_144891 otu_59956 otu_100584 otu_45271 otu_48114 otu_1387 otu_48272 otu_14680
otu_65962 otu_243 otu_89569 otu_143339 otu_17422 otu_49576 otu_157290 otu_115650
otu_160014 otu_129439 otu_14448 otu_45400 otu_149632 otu_117269 otu_92243
otu_60853 otu_11136 otu_58700 otu_159766 otu_12199 otu_93166 otu_161612 otu_10987
otu_100663 otu_46386 otu_92295 otu_97926 otu_47919 otu_57877 otu_130106
otu_149213 otu_17235 otu_86371 otu_53117 otu_49024 otu_132276 otu_20580

[illegible]
otu_120301 otu_17614 otu_19501 otu_61317 otu_93304 otu_19597 otu_149660
otu_138223 otu_156629 otu_92073 otu_12500 otu_21349 otu_59258 otu_16263
otu_105034 otu_87145 otu_120068 otu_101094 otu_58517 otu_87041 otu_102376
otu_19021 otu_161884 otu_92707 otu_46247 otu_149546 otu_116889 otu_17491
otu_13109 otu_56028 otu_93972 otu_13293 otu_121991 otu_129757 otu_17898
otu_144891 otu_59956 otu_100584 otu_45271 otu_48114 otu_1387 otu_48272 otu_14680
otu_65962 otu_243 otu_89569 otu_143339 otu_17422 otu_49576 otu_157290 otu_115650
otu_160014 otu_129439 otu_14448 otu_45400 otu_149632 otu_117269 otu_92243
otu_60853 otu_11136 otu_58700 otu_159766 otu_12199 otu_93166 otu_161612 otu_10987
otu_100663 otu_46386 otu_92295 otu_97926 otu_47919 otu_57877 otu_130106
otu_149213 otu_17235 otu_86371 otu_53117 otu_49024 otu_132276 otu_20580
otu_62041 otu_46763 otu_59904 otu_157370 otu_45841 otu_18687 otu_140134
otu_131067 otu_158368 otu_115875 otu_148420 otu_49941 otu_158770 otu_97850
otu_16911 otu_133369 otu_21321 otu_104203 otu_97475 otu_55722 otu_115748
otu_136363 otu_115918 otu_61648 otu_149338 otu_53868 otu_10708 otu_149209
otu_100627 otu_86599 otu_105202 otu_13238 otu_9274 otu_100724 otu_160470
otu_134075 otu_140392 otu_141664 otu_130059 otu_59322 otu_12660 otu_16246
otu_49104 otu_47917 otu_123304 otu_158049 otu_157893 otu_89106 otu_156618
otu_12805 otu_66577 otu_122975 otu_88529 otu_160560 otu_49366 otu_141358
otu_45507 otu_90771 otu_130772 otu_120365 otu_143295 otu_9493 otu_54995
otu_97673 otu_91601 otu_104805 otu_88596 otu_104489 otu_141578 otu_90859
otu_130152 otu_46913 otu_57542 otu_16796 otu_159945 otu_101913 otu_133351
otu_45523 otu_136927 otu_60141 otu_55306 otu_45990 otu_131720 otu_102027
otu_157186 otu_140405 otu_94358 otu_139423 otu_138786 otu_59983 otu_121031
otu_18889 otu_101582 otu_117805 otu_134893 otu_54880 otu_120730 otu_1192
otu_133371 otu_158231 otu_102679 otu_118860 otu_118859 otu_12536 otu_117527
otu_97610 otu_62105 otu_61284 otu_92055 otu_58355 otu_130587 otu_129302
otu_62950 otu_90750 otu_9303 otu_1413 otu_87001 otu_10618 otu_131385 otu_118542
otu_66296 otu_58466 otu_142999 otu_100464 otu_65095 otu_48329 otu_50900
otu_116505 otu_119837 otu_90561 otu_115777 otu_98481 otu_55923 otu_130730
otu_131641 otu_67452 otu_135489 otu_93059 otu_100455 otu_52264 otu_158788
otu_13764 otu_64578 otu_47549 otu_102260 otu_119768 otu_157289 otu_86818
otu_90634 otu_138951 otu_59546 otu_130458 otu_45620 otu_115721 otu_1956
otu_116198 otu_92155 otu_57678 otu_90540 otu_101650 otu_10717 otu_58253
otu_50672 otu_19806 otu_62476 otu_60847 otu_63560 otu_130565 otu_17480 otu_50176
otu_11815 otu_10406 otu_158792 otu_159956 otu_119861 otu_101215 otu_104899
otu_58315 otu_47059 otu_104839 otu_119406 otu_47455 otu_140224 otu_60640
otu_47161 otu_157143 otu_97560 otu_138923 otu_101232 otu_55711 otu_86275
otu_98030 otu_48563 otu_17638 otu_44798 otu_16042 otu_90123 otu_141298 otu_11715
otu_101000 otu_102096 otu_18624 otu_143532 otu_157135 otu_21471 otu_121889
otu_53206 otu_62612 otu_91021 otu_44269 otu_87479 otu_145097 otu_149215
otu_118766 otu_140248 otu_156579 otu_130189 otu_93205 otu_17175 otu_46580
otu_101576 otu_122241 otu_86978 otu_133912 otu_101665 otu_18833 otu_129437
otu_131411 otu_46004 otu_159987 otu_122773 otu_141606 otu_16690 otu_17145
otu_131209 otu_89187 otu_49452 otu_161806 otu_18319 otu_44678 otu_143525
otu_145351 otu_60998 otu_52411 otu_148973 otu_59357 otu_100635 otu_98441
otu_138932 otu_55336 otu_17260 otu_158021 otu_86238 otu_129753 otu_59696
otu_116682 otu_102311 otu_14804 otu_133943 otu_18049 otu_143364 otu_18214
otu_88627 otu_100 otu_46361 otu_104197 otu_161854 otu_62359 otu_119095 otu_16673
otu_138746 otu_160270 otu_121771 otu_100507 otu_149916 otu_120758 otu_89235
otu_59171 otu_49666 otu_130822 otu_44586 otu_91229 otu_139333 otu_143055
otu_115684 otu_94344 otu_21957 otu_143050 otu_91425 otu_90189 otu_139307
otu_60782 otu_51364 otu_9335 otu_45596 otu_133917 otu_137399 otu_46355 otu_14664
otu_141308 otu_20440 otu_13565 otu_143451 otu_98209 otu_1125 otu_122555
otu_147965 otu_143036 otu_92210 otu_160080 otu_19511 otu_122675 otu_148668
otu_19722 otu_15784 otu_48988 otu_92891 otu_149519 otu_156614 otu_46207
otu_17553 otu_53454 otu_1295 otu_67041 otu_99052 otu_100622 otu_97521 otu_117171
otu_90667 otu_14527 otu_100482 otu_143144 otu_14392 otu_49215 otu_123100 otu_68
otu_53051 otu_101190 otu_105281 otu_131950 otu_97597 otu_160382 otu_13910
otu_49982 otu_149688 otu_135725 otu_132050 otu_119051 otu_148356 otu_59996
otu_19657 otu_61822 otu_115752 otu_123022 otu_48144 otu_66098 otu_55667
otu_148813 otu_95096 otu_11117 otu_130084 otu_18102 otu_101900 otu_93754
otu_61886 otu_9832 otu_57413 otu_60794 otu_100674 otu_136999 otu_19258 otu_13546
otu_57584 otu_44508 otu_16596 otu_51583 otu_119512 otu_157562 otu_141613
otu_44619 otu_16080 otu_48529 otu_135061 otu_133950 otu_12281 otu_137708
otu_164007 otu_51244 otu_94066 otu_121205 otu_159345 otu_156681 otu_148394
otu_49181 otu_63480 otu_90852 otu_89980 otu_143461 otu_149560 otu_145238
otu_141452 otu_86936 otu_57096 otu_120850 otu_119090 otu_10121 otu_9889 otu_11652
otu_56956 otu_58263 otu_46566 otu_121364 otu_50362 otu_143173 otu_50048
otu_58301 otu_14753 otu_1207 otu_94485 otu_161326 otu_9298 otu_47417 otu_161699
otu_159077 otu_122957 otu_11266 otu_51103 otu_44368 otu_44293 otu_51143
otu_100879 otu_91787 otu_65553 otu_54418 otu_138865 otu_161411 otu_119441
otu_11111 otu_129771 otu_87622 otu_1101 otu_157259 otu_54388 otu_104323 otu_64433
otu_118981 otu_47982 otu_118879 otu_118087 otu_158177 otu_160586 otu_119990
otu_47962 otu_130771 otu_89571 otu_158913 otu_159835 otu_129665 otu_16619
otu_130010 otu_48613 otu_16475 otu_104190 otu_1031 otu_58112 otu_105037
otu_104254 otu_158088 otu_58728 otu_16887 otu_13815 otu_93576 otu_118557
otu_98912 otu_93981 otu_11479 otu_133487 otu_118606 otu_144894 otu_47054
otu_97539 otu_92291 otu_1797 otu_88800 otu_10499 otu_98578 otu_60736 otu_48050
otu_88070 otu_143332 otu_86792 otu_9872 otu_116052 otu_86231 otu_91936 otu_87408
otu_12520 otu_148090 otu_94107 otu_89476 otu_118144 otu_101996 otu_46855
otu_61503 otu_86497 otu_148583 otu_116272 otu_148062 otu_134942 otu_134975
otu_9407 otu_59446 otu_1748 otu_149216 otu_132056 otu_57356 otu_46657 otu_44355
otu_148529 otu_156643 otu_17211 otu_133342 otu_157417 otu_148459 otu_130793
otu_101163 otu_11629 otu_162328 otu_138940 otu_66436 otu_159065 otu_16011
otu_50971 otu_88706 otu_143344 otu_16046 otu_140429 otu_129828 otu_130883
otu_54004 otu_115981 otu_48835 otu_60400 otu_130369 otu_86354 otu_65727
otu_20036 otu_13156 otu_158742 otu_88814 otu_148598 otu_129687 otu_45820
otu_156989 otu_161451 otu_115898 otu_120723 otu_45153 otu_158209 otu_2042
otu_60826 otu_20709 otu_141655 otu_12043 otu_133381 otu_20472 otu_122140
otu_161249 otu_46000 otu_149178 otu_94633 otu_65058 otu_164039 otu_50623
otu_140206 otu_12409 otu_158944 otu_49498 otu_48542 otu_1265 otu_10432 otu_45946
otu_20933 otu_47479 otu_143147 otu_118844 otu_122327 otu_10418 otu_47861
otu_64135 otu_17906 otu_144837 otu_44824 otu_12639 otu_48549 otu_53574 otu_18665
otu_158712 otu_46585 otu_101735 otu_116218 otu_137939 otu_161136 otu_160891
otu_120362 otu_52549 otu_144841 otu_102197 otu_10224 otu_59606 otu_63232
otu_135153 otu_10024 otu_94816 otu_157486 otu_21960 otu_44526 otu_90826 otu_1359
otu_13968 otu_136390 otu_86314 otu_1733 otu_16624 otu_95327 otu_60635 otu_52522
otu_20029 otu_19729 otu_1196 otu_1469 otu_51597 otu_130663 otu_59292 otu_93931
otu_47109 otu_19457 otu_137136 otu_66844 otu_19626 otu_10854 otu_52547 otu_49738
otu_89055 otu_12544 otu_17567 otu_45120 otu_47257 otu_117892 otu_121035
otu_86618 otu_120181 otu_9977 otu_119611 otu_159758 otu_91406 otu_157695
otu_135677 otu_93824 otu_16301 otu_46819 otu_59773 otu_12863 otu_104404
otu_121139 otu_12193 otu_115798 otu_56447 otu_139324 otu_149672 otu_88651
otu_141571 otu_62379 otu_66323 otu_86406 otu_160423 otu_1653 otu_64647 otu_14984
otu_117509 otu_159879 otu_120188 otu_57683 otu_119789 otu_91238 otu_144874
otu_161242 otu_115993 otu_17033 otu_10571 otu_44725 otu_116951 otu_140448
otu_50390 otu_161466 otu_157763 otu_160952 otu_88057 otu_14478 otu_16548
[illegible]

otu_13968 otu_136390 otu_86314 otu_1733 otu_16624 otu_95327 otu_60635 otu_52522
otu_20029 otu_19729 otu_1196 otu_1469 otu_51597 otu_130663 otu_59292 otu_93931
otu_47109 otu_19457 otu_137136 otu_66844 otu_19626 otu_10854 otu_52547 otu_49738
otu_89055 otu_12544 otu_17567 otu_45120 otu_47257 otu_117892 otu_121035
otu_86618 otu_120181 otu_9977 otu_119611 otu_159758 otu_91406 otu_157695
otu_135677 otu_93824 otu_16301 otu_46819 otu_59773 otu_12863 otu_104404
otu_121139 otu_12193 otu_115798 otu_56447 otu_139324 otu_149672 otu_88651
otu_141571 otu_62379 otu_66323 otu_86406 otu_160423 otu_1653 otu_64647 otu_14984
otu_117509 otu_159879 otu_120188 otu_57683 otu_119789 otu_91238 otu_144874
otu_161242 otu_115993 otu_17033 otu_10571 otu_44725 otu_116951 otu_140448
otu_50390 otu_161466 otu_157763 otu_160952 otu_88057 otu_14478 otu_16548
otu_10307 otu_18541 otu_116146 otu_87440 otu_143602 otu_59313 otu_18070
otu_157346 otu_136908 otu_104479 otu_44817 otu_66239 otu_119649 otu_118951
otu_159736 otu_13319 otu_122208 otu_57936 otu_129562 otu_45267 otu_1983
otu_17631 otu_94906 otu_148331 otu_138280 otu_134969 otu_138033 otu_59648
otu_92099 otu_135135 otu_1699 otu_119260 otu_115683 otu_44765 otu_97801
otu_119976 otu_115627 otu_147964 otu_160359 otu_45029 otu_130671 otu_145295
otu_97489 otu_16996 otu_116555 otu_160043 otu_11615 otu_66210 otu_115759
otu_118384 otu_10048 otu_18760 otu_11722 otu_44841 otu_87221 otu_16904 otu_116840
otu_147976 otu_141329 otu_92793 otu_59073 otu_143471 otu_92143 otu_148374
otu_14536 otu_161399 otu_117852 otu_49696 otu_51264 otu_17763 otu_135459
otu_58561 otu_52602 otu_121432 otu_119186 otu_9912 otu_64624 otu_120244
otu_53804 otu_68629 otu_47433 otu_1935 otu_44467 otu_135628 otu_119291
otu_129708 otu_86741 otu_9247 otu_92413 otu_149162 otu_148199 otu_91473
otu_102072 otu_10935 otu_101267 otu_120872 otu_91828 otu_52002 otu_88759
otu_120130 otu_57690 otu_158044 otu_93584 otu_115848 otu_9338 otu_123829
otu_118508 otu_10869 otu_136615 otu_12570 otu_104585 otu_129523 otu_91748
otu_11511 otu_156784 otu_137051 otu_91228 otu_121324 otu_47329 otu_117277
otu_119691 otu_149534 otu_92038 otu_120884 otu_51424 otu_149023 otu_118686
otu_147859 otu_141562 otu_44631 otu_161706 otu_57564 otu_129440 otu_140256
otu_161903 otu_12453 otu_58094 otu_66991 otu_59340 otu_137544 otu_50897
otu_11947 otu_141288 otu_137132 otu_12596 otu_137208 otu_100872 otu_17440
otu_10077 otu_120353 otu_89518 otu_129689 otu_57744 otu_44702 otu_91216
otu_53151 otu_12904 otu_130787 otu_1710 otu_158510 otu_102363 otu_115908
otu_138965 otu_161381 otu_143130 otu_141584 otu_20377 otu_87176 otu_90578
otu_133409 otu_158085 otu_143402 otu_138222 otu_10589 otu_91842 otu_97584
otu_90497 otu_64760 otu_1579 otu_1922 otu_52745 otu_16805 otu_119845 otu_141276
otu_104671 otu_17899 otu_13509 otu_86661 otu_147901 otu_17239 otu_92375
otu_45876 otu_136667 otu_131027 otu_50908 otu_118243 otu_117998 otu_20686
otu_16208 otu_235 otu_15476 otu_134076 otu_119438 otu_45727 otu_61496 otu_48526
otu_9685 otu_92221 otu_97546 otu_129631 otu_87142 otu_89792 otu_140192 otu_57788
otu_100528 otu_17840 otu_115970 otu_19502 otu_12826 otu_118726 otu_116212
otu_118702 otu_62810 otu_116860 otu_1670 otu_121776 otu_104719 otu_120613
otu_45392 otu_115592 otu_60738 otu_157458 otu_91309 otu_50076 otu_56690
otu_104419 otu_1309 otu_17560 otu_137471 otu_1979 otu_134971 otu_139305
otu_119411 otu_21605 otu_49142 otu_131888 otu_54666 otu_157323 otu_58960
otu_104874 otu_55075 otu_91951 otu_98411 otu_65201 otu_9479 otu_100921
otu_140080 otu_87956 otu_47675 otu_56510 otu_63255 otu_9652 otu_135103
otu_102377 otu_9319 otu_68459 otu_90714 otu_54723 otu_129379 otu_161184
otu_104189 otu_58898 otu_102021 otu_163979 otu_17691 otu_101601 otu_102650
otu_18547 otu_60176 otu_131116 otu_115851 otu_136531 otu_2090 otu_90924 otu_45692
otu_16489 otu_87311 otu_17575 otu_122428 otu_52296 otu_147915 otu_148200
otu_86259 otu_157543 otu_45634 otu_57765 otu_138894 otu_58586 otu_12097
otu_158302 otu_45189 otu_86361 otu_129652 otu_149269 otu_17439 otu_50312
otu_58369 otu_12255 otu_46509 otu_139311 otu_18017 otu_9290 otu_20316 otu_148931
otu_90290 otu_50587 otu_145018 otu_88696 otu_64341 otu_91920 otu_145439
otu_59725 otu_157168 otu_117482 otu_9311 otu_120190 otu_50480 otu_145314
otu_93260 otu_98065 otu_116132 otu_11022 otu_65654 otu_90560 otu_13568 otu_115713
otu_105046 otu_86313 otu_130964 otu_98427 otu_115942 otu_90038 otu_62453
otu_117157 otu_156749 otu_101967 otu_65331 otu_101725 otu_119867 otu_100624
otu_13972 otu_160198 otu_138000 otu_62249 otu_120553 otu_62330 otu_88139
otu_137971 otu_65733 otu_159998 otu_116156 otu_117312 otu_148436 otu_18055
otu_10644 otu_46306 otu_141293 otu_15723 otu_1760 otu_148204 otu_92668 otu_61555
otu_61985 otu_157318 otu_137942 otu_92910 otu_9961 otu_90904 otu_159042
otu_57967 otu_48981 otu_44789 otu_14824 otu_67139 otu_161519 otu_10757 otu_18969
otu_46686 otu_93292 otu_141385 otu_134892 otu_161491 otu_149426 otu_1777
otu_145354 otu_13543 otu_57917 otu_138928 otu_133922 otu_130708 otu_10209
otu_86639 otu_1494 otu_12653 otu_19533 otu_145123 otu_59174 otu_118718
otu_131013 otu_160428 otu_123097 otu_51948 otu_17200 otu_66378 otu_91540
otu_156934 otu_129237 otu_116488 otu_105140 otu_149572 otu_135109 otu_88101
otu_59426 otu_44747 otu_141409 otu_140197 otu_49908 otu_63177 otu_9935 otu_90972
otu_46559 otu_10795 otu_156815 otu_11090 otu_65352 otu_98108 otu_58116 otu_116199
otu_91020 otu_119772 otu_13989 otu_92306 otu_115975 otu_58744 otu_44633
otu_135022 otu_14241 otu_48321 otu_97691 otu_51763 otu_120882 otu_159099
otu_157957 otu_120296 otu_162639 otu_156586 otu_16939 otu_156943 otu_19507
otu_55110 otu_90686 otu_20966 otu_120517 otu_144899 otu_97833 otu_56911 otu_11856
otu_50532 otu_136001 otu_131121 otu_9331 otu_134118 otu_58080 otu_122504
otu_129554 otu_159862 otu_131236 otu_134269 otu_18841 otu_130834 otu_58587
otu_115622 otu_91725 otu_53389 otu_156714 otu_97574 otu_94329 otu_45695 otu_1639
otu_159474 otu_63380 otu_100601 otu_11017 otu_156769 otu_140175 otu_89399
otu_149431 otu_14086 otu_121942 otu_141576 otu_148637 otu_141704 otu_119799
otu_11357 otu_21785 otu_1159 otu_157930 otu_9624 otu_16468 otu_160892 otu_44392
otu_93945 otu_156662 otu_141550 otu_46555 otu_160038 otu_9641 otu_16729
otu_50057 otu_13031 otu_58452 otu_63659 otu_47254 otu_156938 otu_9569 otu_98014
otu_20837 otu_104158 otu_93474 otu_118587 otu_129839 otu_100661 otu_57068
otu_16328 otu_59329 otu_17481 otu_147985 otu_46793 otu_158317 otu_16857
otu_141640 otu_48414 otu_148051 otu_46626 otu_133443 otu_11212 otu_14146
otu_101931 otu_52943 otu_50060 otu_57907 otu_131162 otu_141610 otu_133749
otu_148288 otu_19476 otu_45390 otu_100952 otu_149285 otu_120197 otu_116332
otu_60698 otu_60367 otu_117734 otu_130164 otu_88455 otu_119590 otu_64581
otu_100547 otu_149286 otu_49281 otu_49174 otu_46616 otu_135066 otu_134018
otu_93419 otu_93098 otu_47985 otu_63337 otu_54555 otu_130721 otu_97465
otu_131533 otu_122236 otu_148935 otu_61942 otu_129629 otu_158471 otu_15186
otu_120153 otu_119377 otu_119993 otu_158997 otu_148134 otu_51532 otu_92187
otu_15966 otu_123220 otu_44232 otu_149505 otu_1407 otu_136246 otu_47151
otu_52672 otu_64538 otu_91813 otu_65379 otu_148186 otu_145051 otu_86196
otu_101671 otu_61007 otu_21320 otu_61085 otu_18359 otu_143022 otu_20068
otu_58224 otu_13216 otu_133456 otu_49658 otu_44669 otu_11241 otu_148777
otu_121359 otu_44276 otu_11601 otu_159388 otu_15192 otu_117775 otu_158249
otu_65089 otu_101503 otu_10501 otu_10346 otu_133662 otu_147848 otu_133375
otu_49509 otu_9748 otu_119460 otu_115649 otu_13811 otu_1034 otu_136609 otu_102513
otu_49407 otu_19946 otu_161788 otu_18054 otu_45981 otu_140321 otu_18962
otu_139340 otu_119553 otu_123082 otu_87680 otu_48975 otu_156974 otu_19703
otu_59734 otu_144852 otu_62720 otu_139409 otu_118877 otu_47953 otu_45960
otu_10323 otu_63800 otu_13142 otu_118628 otu_149173 otu_14956 otu_53717
otu_13987 otu_11274 otu_57424 otu_45169 otu_58800 otu_135833 otu_17000
otu_105024 otu_141712 otu_19733 otu_143243 otu_116704 otu_115669 otu_9436
otu_46039 otu_18823 otu_66885 otu_149854 otu_44542 otu_60693 otu_161769
otu_120534 otu_129658 otu_118711 otu_91835 otu_46072 otu_136243 otu_15124

		[illegible]<br>otu_121359 otu_44276 otu_11601 otu_159388 otu_15192 otu_117775 otu_158249<br>otu_65089 otu_101503 otu_10501 otu_10346 otu_133662 otu_147848 otu_133375<br>otu_49509 otu_9748 otu_119460 otu_115649 otu_13811 otu_1034 otu_136609 otu_102513<br>otu_49407 otu_19946 otu_161788 otu_18054 otu_45981 otu_140321 otu_18962<br>otu_139340 otu_119553 otu_123082 otu_87680 otu_48975 otu_156974 otu_19703<br>otu_59734 otu_144852 otu_62720 otu_139409 otu_118877 otu_47953 otu_45960<br>otu_10323 otu_63800 otu_13142 otu_118628 otu_149173 otu_14956 otu_53717<br>otu_13987 otu_11274 otu_57424 otu_45169 otu_58800 otu_135833 otu_17000<br>otu_105024 otu_141712 otu_19733 otu_143243 otu_116704 otu_115669 otu_9436<br>otu_46039 otu_18823 otu_66885 otu_149854 otu_44542 otu_60693 otu_161769<br>otu_120534 otu_129658 otu_118711 otu_91835 otu_46072 otu_136243 otu_15124<br>otu_157194 otu_158949 otu_46897 otu_11463 otu_90111 otu_93282 otu_18929 otu_16744<br>otu_16607 otu_49057 otu_118594 otu_158141 otu_11339 otu_100777 otu_47715<br>otu_56249 otu_118774 otu_49831 otu_45070 otu_115784 otu_64035 otu_120126<br>otu_57962 otu_91549 otu_135549 otu_101570 otu_137929 otu_120333 otu_148982<br>otu_149698 otu_130372 otu_149577 otu_104924 otu_87564 otu_51749 otu_117178<br>otu_63420 otu_59562 otu_115793 otu_98531 otu_62975 otu_21392 otu_12190 otu_10232<br>otu_160168 otu_160289 otu_46378 otu_105263 otu_140218 otu_17718 otu_44882<br>otu_9915 otu_160234 otu_55453 otu_137567 otu_59165 otu_11350 otu_60639<br>otu_160128 otu_138995 otu_121624 otu_157696 otu_134136 otu_61551 otu_17666<br>otu_49930 otu_88348 otu_138663 otu_58577 otu_1771 otu_130802 otu_141284<br>otu_45288 otu_64846 otu_157065 otu_12640 otu_89712 otu_57329 otu_90931<br>otu_141748 otu_149072 otu_86673 otu_16391 otu_90158 otu_17597 otu_129231<br>otu_142986 otu_135082 otu_157512 otu_57627
mpolyiv	1656	otu_69837 otu_70938 otu_70763 otu_70840 otu_125006 otu_71018 otu_125302<br>otu_75265 otu_22232 otu_70807 otu_22061 otu_163050 otu_162894 otu_73167<br>otu_74039 otu_71644 otu_163128 otu_71538 otu_71085 otu_69421 otu_22089<br>otu_162921 otu_163007 otu_69177 otu_73934 otu_71355 otu_72741 otu_68991<br>otu_22237 otu_75049 otu_124978 otu_72877 otu_105323 otu_163052 otu_69592<br>otu_69949 otu_74343 otu_125235 otu_72440 otu_70031 otu_76499 otu_70849 otu_70882<br>otu_70642 otu_69710 otu_70777 otu_72005 otu_73477 otu_162907 otu_71132 otu_70293<br>otu_73567 otu_70415 otu_69623 otu_74862 otu_74047 otu_105343 otu_70482<br>otu_125844 otu_163107 otu_69036 otu_123961 otu_69342 otu_72810 otu_72869<br>otu_124234 otu_69673 otu_71529 otu_75132 otu_73836 otu_73917 otu_22114 otu_75513<br>otu_124075 otu_162967 otu_69289 otu_125069 otu_22154 otu_162919 otu_162982<br>otu_22173 otu_125576 otu_71335 otu_22094 otu_72435 otu_70277 otu_74121 otu_73941<br>otu_162924 otu_125424 otu_22168 otu_72714 otu_72343 otu_125366 otu_22102<br>otu_75236 otu_69701 otu_69976 otu_125871 otu_70579 otu_69665 otu_72923<br>otu_124927 otu_123983 otu_22196 otu_71348 otu_70557 otu_70446 otu_75500<br>otu_141765 otu_69253 otu_70677 otu_126136 otu_69548 otu_22215 otu_125635<br>otu_2094 otu_73931 otu_69133 otu_70771 otu_163092 otu_74964 otu_70211 otu_72475<br>otu_69435 otu_70377 otu_69088 otu_22134 otu_68968 otu_71658 otu_69414 otu_72157<br>otu_73209 otu_124439 otu_69137 otu_69908 otu_69712 otu_69041 otu_76425 otu_2098<br>otu_163095 otu_162883 otu_105375 otu_71477 otu_70754 otu_69361 otu_71784<br>otu_162956 otu_69988 otu_70953 otu_70349 otu_69300 otu_69310 otu_69382 otu_69014<br>otu_69619 otu_150014 otu_22111 otu_70691 otu_70242 otu_69127 otu_22053 otu_71194<br>otu_69389 otu_68987 otu_73465 otu_70646 otu_72019 otu_126003 otu_22184<br>otu_123954 otu_70037 otu_124168 otu_72212 otu_75518 otu_74422 otu_124201<br>otu_73018 otu_70533 otu_69164 otu_69081 otu_150033 otu_71514 otu_70612 otu_22052<br>otu_71109 otu_124205 otu_72959 otu_70530 otu_69392 otu_74765 otu_70873 otu_72139<br>otu_70839 otu_70927 otu_124047 otu_69052 otu_72478 otu_70066 otu_71791<br>otu_150040 otu_73262 otu_162901 otu_74000 otu_71967 otu_22195 otu_76387<br>otu_69139 otu_124076 otu_68916 otu_70711 otu_69360 otu_72388 otu_70077<br>otu_124926 otu_124011 otu_72962 otu_69174 otu_22057 otu_22139 otu_75296<br>otu_69429 otu_141766 otu_74669 otu_76585 otu_124901 otu_22147 otu_162929<br>otu_124689 otu_145850 otu_22125 otu_162904 otu_70658 otu_70140 otu_70593<br>otu_69066 otu_71388 otu_70351 otu_73546 otu_125879 otu_70100 otu_69078<br>otu_150022 otu_125607 otu_150030 otu_124165 otu_124280 otu_150038 otu_71356<br>otu_71652 otu_22078 otu_124768 otu_143734 otu_68907 otu_22214 otu_71125<br>otu_70187 otu_69711 otu_69352 otu_69956 otu_70505 otu_69283 otu_141770 otu_74757<br>otu_75770 otu_72166 otu_70696 otu_105351 otu_105372 otu_145637 otu_76563<br>otu_75635 otu_125710 otu_69107 otu_71563 otu_72723 otu_73873 otu_138387<br>otu_69239 otu_69043 otu_73338 otu_69278 otu_70040 otu_69572 otu_125042 otu_73181<br>otu_70084 otu_125017 otu_162884 otu_105324 otu_70263 otu_76200 otu_69879<br>otu_73853 otu_75017 otu_145651 otu_74190 otu_71228 otu_71201 otu_71891<br>otu_163026 otu_22238 otu_69698 otu_71962 otu_71505 otu_70844 otu_163121<br>otu_72693 otu_69657 otu_69373 otu_69476 otu_70940 otu_69597 otu_22199 otu_72222<br>otu_71515 otu_70872 otu_125954 otu_70294 otu_334 otu_145642 otu_69940 otu_70883<br>otu_124044 otu_74188 otu_69272 otu_71155 otu_73023 otu_162908 otu_22142<br>otu_69138 otu_162891 otu_75421 otu_69149 otu_22086 otu_124037 otu_72983<br>otu_69624 otu_75341 otu_71579 otu_71037 otu_125152 otu_69560 otu_73633 otu_74764<br>otu_145761 otu_75752 otu_75112 otu_71495 otu_69775 otu_123982 otu_22245<br>otu_68938 otu_70159 otu_73271 otu_73911 otu_74686 otu_72233 otu_124239 otu_72614<br>otu_70019 otu_124141 otu_137796 otu_150025 otu_72464 otu_75106 otu_70878<br>otu_124174 otu_71750 otu_69522 otu_73244 otu_125084 otu_22119 otu_72082<br>otu_69232 otu_162974 otu_75018 otu_126116 otu_132386 otu_69308 otu_163110<br>otu_69249 otu_123964 otu_69898 otu_74196 otu_74318 otu_74161 otu_70227 otu_69807<br>otu_22066 otu_22071 otu_71293 otu_162950 otu_73803 otu_71566 otu_70814 otu_69458<br>otu_71353 otu_74357 otu_74248 otu_70664 otu_71319 otu_73948 otu_71874 otu_124008<br>otu_163076 otu_69892 otu_72587 otu_71223 otu_74158 otu_69146 otu_73202 otu_69463<br>otu_73033 otu_69582 otu_162905 otu_70258 otu_68940 otu_69609 otu_75919<br>otu_141767 otu_70525 otu_22207 otu_69166 otu_102786 otu_162948 otu_71363<br>otu_69452 otu_22099 otu_22051 otu_69829 otu_22080 otu_69644 otu_70107 otu_70954<br>otu_70057 otu_70046 otu_124228 otu_73801 otu_69340 otu_69377 otu_69486 otu_74060<br>otu_68954 otu_70644 otu_70229 otu_68903 otu_124859 otu_68917 otu_69218 otu_70146<br>otu_72669 otu_125043 otu_70403 otu_76280 otu_22152 otu_69783 otu_71320<br>otu_124185 otu_69770 otu_72208 otu_69028 otu_75306 otu_22225 otu_72700 otu_22162<br>otu_124559 otu_69120 otu_71219 otu_71153 otu_71395 otu_22132 otu_124989<br>otu_124733 otu_162896 otu_69931 otu_72998 otu_75456 otu_70210 otu_70299<br>otu_71278 otu_70640 otu_22068 otu_69152 otu_70809 otu_72922 otu_69689 otu_163131<br>otu_70067 otu_69727 otu_70310 otu_70206 otu_145653 otu_22174 otu_72084<br>otu_163015 otu_69682 otu_163105 otu_69658 otu_69208 otu_73482 otu_72155<br>otu_124147 otu_150028 otu_70226 otu_73954 otu_74094 otu_105325 otu_162971<br>otu_69503 otu_69541 otu_73192 otu_69380 otu_73561 otu_69175 otu_74778 otu_73975<br>otu_69585 otu_71521 otu_74291 otu_69653 otu_123957 otu_73130 otu_69505 otu_72017<br>otu_71671 otu_69387 otu_69374 otu_72928 otu_69114 otu_68906 otu_73821 otu_70904<br>otu_124567 otu_22116 otu_75463 otu_69641 otu_143727 otu_73695 otu_68980<br>otu_71423 otu_70286 otu_68970 otu_73258 otu_74970 otu_72712 otu_70194 otu_69960<br>otu_70547 otu_71581 otu_163051 otu_124093 otu_22137 otu_69324 otu_69307<br>otu_163005 otu_72184 otu_125261 otu_69055 otu_74033 otu_70746 otu_70820<br>otu_70804 otu_70813 otu_71424 otu_71374 otu_69697 otu_71887 otu_72382 otu_150044<br>otu_162973 otu_22079 otu_70429 otu_75221 otu_72838 otu_124005 otu_69849<br>otu_70448 otu_70502 otu_125487 otu_124117 otu_75071 otu_76484 otu_22178<br>otu_70675 otu_70001 otu_70009 otu_69233 otu_2096 otu_72826 otu_69533 otu_162914<br>otu_69178 otu_69046 otu_69768 otu_70611 otu_125437 otu_162988 otu_73737<br>otu_143725 otu_125683 otu_70163 otu_69071 otu_69556 otu_69997 otu_72242<br>otu_73168 otu_162938 otu_124320 otu_22113 otu_73010 otu_124250 otu_125468<br>[illegible]

otu_124567 otu_22116 otu_75463 otu_69641 otu_143727 otu_73695 otu_68980
otu_71423 otu_70286 otu_68970 otu_73258 otu_74970 otu_72712 otu_70194 otu_69960
otu_70547 otu_71581 otu_163051 otu_124093 otu_22137 otu_69324 otu_69307
otu_163005 otu_72184 otu_125261 otu_69055 otu_74033 otu_70746 otu_70820
otu_70804 otu_70813 otu_71424 otu_71374 otu_69697 otu_71887 otu_72382 otu_150044
otu_162973 otu_22079 otu_70429 otu_75221 otu_72838 otu_124005 otu_69849
otu_70448 otu_70502 otu_125487 otu_124117 otu_75071 otu_76484 otu_22178
otu_70675 otu_70001 otu_70009 otu_69233 otu_2096 otu_72826 otu_69533 otu_162914
otu_69178 otu_69046 otu_69768 otu_70611 otu_125437 otu_162988 otu_73737
otu_143725 otu_125683 otu_70163 otu_69071 otu_69556 otu_69997 otu_72242
otu_73168 otu_162938 otu_124320 otu_22113 otu_73010 otu_124250 otu_125468
otu_70901 otu_72911 otu_69679 otu_68909 otu_69726 otu_143736 otu_71603 otu_73124
otu_71376 otu_72259 otu_68975 otu_72757 otu_72668 otu_22182 otu_162886
otu_124138 otu_69751 otu_163084 otu_162916 otu_124805 otu_69102 otu_22231
otu_71249 otu_75055 otu_74610 otu_69207 otu_124942 otu_22236 otu_69791
otu_162880 otu_164232 otu_70588 otu_162879 otu_124049 otu_105322 otu_69574
otu_73078 otu_70246 otu_76459 otu_68978 otu_150041 otu_74397 otu_70788 otu_72220
otu_72095 otu_69543 otu_22050 otu_22140 otu_69590 otu_105382 otu_69530
otu_145709 otu_124361 otu_71233 otu_22193 otu_70895 otu_70870 otu_71336
otu_69224 otu_74382 otu_70064 otu_124895 otu_69132 otu_69165 otu_125282
otu_69200 otu_71804 otu_75931 otu_124330 otu_69359 otu_72960 otu_72548 otu_70917
otu_72732 otu_69341 otu_71808 otu_70549 otu_69937 otu_69453 otu_22120 otu_71039
otu_75114 otu_123995 otu_71558 otu_124313 otu_22123 otu_71107 otu_125205
otu_74410 otu_70552 otu_162927 otu_70656 otu_124791 otu_125267 otu_22105
otu_71163 otu_70271 otu_70966 otu_72245 otu_75820 otu_70866 otu_69515 otu_69481
otu_105374 otu_105370 otu_123984 otu_162984 otu_69116 otu_124655 otu_163063
otu_73273 otu_73631 otu_70143 otu_71366 otu_73423 otu_105363 otu_71015 otu_69401
otu_69513 otu_105340 otu_70016 otu_123962 otu_69876 otu_69713 otu_73698
otu_69752 otu_73268 otu_164359 otu_72799 otu_22251 otu_124601 otu_70837
otu_162976 otu_70413 otu_124181 otu_71855 otu_124283 otu_22197 otu_72500
otu_124021 otu_143742 otu_124050 otu_71136 otu_162962 otu_162903 otu_125356
otu_70192 otu_125025 otu_69777 otu_69432 otu_162980 otu_69311 otu_125552
otu_69375 otu_69183 otu_70012 otu_74372 otu_71268 otu_72768 otu_69474 otu_69112
otu_22047 otu_145723 otu_72399 otu_69688 otu_70634 otu_73995 otu_70203 otu_74437
otu_70095 otu_71008 otu_124838 otu_22209 otu_163136 otu_125733 otu_69094
otu_69943 otu_70215 otu_71487 otu_74537 otu_125561 otu_124849 otu_70880
otu_75294 otu_71405 otu_71331 otu_22095 otu_70931 otu_73883 otu_75200 otu_69383
otu_69075 otu_22194 otu_74212 otu_69595 otu_71475 otu_22084 otu_76198 otu_71362
otu_73742 otu_69740 otu_70122 otu_69968 otu_124015 otu_76076 otu_124095
otu_22058 otu_71712 otu_74768 otu_124587 otu_69904 otu_70078 otu_69744
otu_145645 otu_124010 otu_124322 otu_69326 otu_72431 otu_125060 otu_71031
otu_22090 otu_22082 otu_70500 otu_68924 otu_22124 otu_75417 otu_69717 otu_125564
otu_22223 otu_22164 otu_150015 otu_124934 otu_22244 otu_124112 otu_70921
otu_125148 otu_73671 otu_72658 otu_69393 otu_125178 otu_74177 otu_99169
otu_69856 otu_141761 otu_72963 otu_75771 otu_75432 otu_125053 otu_70774
otu_124443 otu_69211 otu_125836 otu_70621 otu_73973 otu_71562 otu_70172
otu_124145 otu_69766 otu_70124 otu_69648 otu_162885 otu_69853 otu_70272
otu_70750 otu_125204 otu_22221 otu_124026 otu_72934 otu_73867 otu_70262
otu_69461 otu_73998 otu_69743 otu_74360 otu_70731 otu_69079 otu_73778 otu_68963
otu_69261 otu_71653 otu_150037 otu_74640 otu_22077 otu_71880 otu_22242 otu_73604
otu_162930 otu_162946 otu_75595 otu_69919 otu_74419 otu_69912 otu_70105
otu_69201 otu_162912 otu_105371 otu_145638 otu_150023 otu_69296 otu_74653
otu_72834 otu_22210 otu_70498 otu_69442 otu_141762 otu_22085 otu_76079
otu_123974 otu_124159 otu_162890 otu_74624 otu_22156 otu_71014 otu_124116
otu_69880 otu_150009 otu_74254 otu_69962 otu_124039 otu_73442 otu_70683
otu_125484 otu_75298 otu_70013 otu_74835 otu_125287 otu_69005 otu_73762
otu_163133 otu_125037 otu_124629 otu_124045 otu_69115 otu_125018 otu_68998
otu_68995 otu_70519 otu_72384 otu_70879 otu_70386 otu_70239 otu_69531 otu_73989
otu_70443 otu_71445 otu_69897 otu_162998 otu_70631 otu_71639 otu_70007 otu_75639
otu_74818 otu_132383 otu_125591 otu_124175 otu_72307 otu_71088 otu_124127
otu_69680 otu_124726 otu_73532 otu_70228 otu_145734 otu_22065 otu_72367
otu_162888 otu_105320 otu_73394 otu_71497 otu_73345 otu_163069 otu_71939
otu_74411 otu_22045 otu_124511 otu_71045 otu_70574 otu_69446 otu_22234 otu_69643
otu_123959 otu_126117 otu_71673 otu_125480 otu_69500 otu_74989 otu_69484
otu_163939 otu_143729 otu_69519 otu_69274 otu_162933 otu_124238 otu_150019
otu_70470 otu_71326 otu_69521 otu_125461 otu_70044 otu_71692 otu_162943
otu_72816 otu_69838 otu_22118 otu_70357 otu_124040 otu_70680 otu_162876
otu_105380 otu_71019 otu_163036 otu_70109 otu_22130 otu_72990 otu_69205
otu_68964 otu_70673 otu_76614 otu_124829 otu_163162 otu_69006 otu_22049
otu_69154 otu_70805 otu_125870 otu_74791 otu_68926 otu_125332 otu_70191
otu_70273 otu_70068 otu_69620 otu_124908 otu_125325 otu_70912 otu_69189
otu_70282 otu_69369 otu_73866 otu_69510 otu_71051 otu_70968 otu_69020 otu_22108
otu_162895 otu_125026 otu_69145 otu_71720 otu_69437 otu_99175 otu_76191
otu_105365 otu_125003 otu_74335 otu_68972 otu_71297 otu_124563 otu_69451
otu_69753 otu_69821 otu_76234 otu_124220 otu_69558 otu_71184 otu_125206
otu_22067 otu_124259 otu_69640 otu_68981 otu_124353 otu_73169 otu_69338
otu_105379 otu_323 otu_69365 otu_105358 otu_145745 otu_72586 otu_71431 otu_75911
otu_71131 otu_70335 otu_150018 otu_163887 otu_70141 otu_72205 otu_71220
otu_71574 otu_69091 otu_73578 otu_69498 otu_22226 otu_69269 otu_69881 otu_22115
otu_73205 otu_70481 otu_69654 otu_75004 otu_71847 otu_69406 otu_124854 otu_75455
otu_69037 otu_70383 otu_2095 otu_70520 otu_125554 otu_70495 otu_69547 otu_22044
otu_71142 otu_72665 otu_69159 otu_76531 otu_73560 otu_124836 otu_124404
otu_162932 otu_70212 otu_69334 otu_22056 otu_68899 otu_73036 otu_69083 otu_74102
otu_70440 otu_69023 otu_124727 otu_163174 otu_69569 otu_22093 otu_69195
otu_163023 otu_124623 otu_125842 otu_22129 otu_143717 otu_68923 otu_72368
otu_124335 otu_145771 otu_150043 otu_70317 otu_70341 otu_70554 otu_162960
otu_75064 otu_68986 otu_69256 otu_163020 otu_70499 otu_71309 otu_69073 otu_75260
otu_68918 otu_72018 otu_123953 otu_22181 otu_70150 otu_70447 otu_71028 otu_69508
otu_73100 otu_71866 otu_163004 otu_70730 otu_74001 otu_162915 otu_124822
otu_75080 otu_69460 otu_70907 otu_70183 otu_69677 otu_22074 otu_69080 otu_22040
otu_124985 otu_124025 otu_105328 otu_22165 otu_163094 otu_74227 otu_124247
otu_124423 otu_71993 otu_69581 otu_145686 otu_105345 otu_162882 otu_69926
otu_124023 otu_125895 otu_71548 otu_68977 otu_150013 otu_71877 otu_162957
otu_69987 otu_70791 otu_73227 otu_162887 otu_69246 otu_143748 otu_69276
otu_125637 otu_70283 otu_22235 otu_162881 otu_124243 otu_123946 otu_69617
otu_69302 otu_70724 otu_71416 otu_163011 otu_69998 otu_71202 otu_125132
otu_162878 otu_71437 otu_72746 otu_68943 otu_126104 otu_22092 otu_70831
otu_22240 otu_69820 otu_74784 otu_72677 otu_124321 otu_69630 otu_2099 otu_73865
otu_75998 otu_71662 otu_125829 otu_69282 otu_125055 otu_68953 otu_162965
otu_69914 otu_75861 otu_73211 otu_22200 otu_70685 otu_72984 otu_70514 otu_73127
otu_124815 otu_71281 otu_71428 otu_69502 otu_22122 otu_68947 otu_164202
otu_163009 otu_125959 otu_163055 otu_71559 otu_72376 otu_163090 otu_22104
otu_70992 otu_72530 otu_69328 otu_68969 otu_71642 otu_163126 otu_162926
otu_145791 otu_69650 otu_69131 otu_70384 otu_69059 otu_22198 otu_125233
otu_74018 otu_22155 otu_22131 otu_124360 otu_70369 otu_73401 otu_69082 otu_71143
otu_69213 otu_22185 otu_69259 otu_70455 otu_70847 otu_71544 otu_70506 otu_71333
otu_123963 otu_71321 otu_70565 otu_70176 otu_74734 otu_71226 otu_124129
otu_71782 otu_105341 otu_73354 otu_72641 otu_69035 otu_69238 otu_72936 otu_72246
otu_70395 otu_162996 otu_73638 otu_124705 otu_69242 otu_71616 otu_74645

		otu_22240 otu_69820 otu_74784 otu_72677 otu_124321 otu_69630 otu_2099 otu_73865<br>otu_75998 otu_71662 otu_125829 otu_69282 otu_125055 otu_68953 otu_162965<br>otu_69914 otu_75861 otu_73211 otu_22200 otu_70685 otu_72984 otu_70514 otu_73127<br>otu_124815 otu_71281 otu_71428 otu_69502 otu_22122 otu_68947 otu_164202<br>otu_163009 otu_125959 otu_163055 otu_71559 otu_72376 otu_163090 otu_22104<br>otu_70992 otu_72530 otu_69328 otu_68969 otu_71642 otu_163126 otu_162926<br>otu_145791 otu_69650 otu_69131 otu_70384 otu_69059 otu_22198 otu_125233<br>otu_74018 otu_22155 otu_22131 otu_124360 otu_70369 otu_73401 otu_69082 otu_71143<br>otu_69213 otu_22185 otu_69259 otu_70455 otu_70847 otu_71544 otu_70506 otu_71333<br>otu_123963 otu_71321 otu_70565 otu_70176 otu_74734 otu_71226 otu_124129<br>otu_71782 otu_105341 otu_73354 otu_72641 otu_69035 otu_69238 otu_72936 otu_72246<br>otu_70395 otu_162996 otu_73638 otu_124705 otu_69242 otu_71616 otu_74645<br>otu_22203 otu_70121 otu_73177 otu_71494 otu_72100 otu_71564 otu_71467 otu_22252<br>otu_73498 otu_74174 otu_145634 otu_105338 otu_69203 otu_69214 otu_123985<br>otu_69482 otu_74298 otu_124422 otu_69598 otu_68913 otu_124448 otu_150021<br>otu_124255 otu_70167 otu_69158 otu_22055 otu_125303 otu_74424 otu_69281<br>otu_124182 otu_74594 otu_140660 otu_68930 otu_74434 otu_71367 otu_150017<br>otu_71080 otu_70081 otu_69252 otu_163114 otu_124736 otu_73780 otu_69514<br>otu_124203 otu_22166 otu_70790 otu_22145 otu_69141 otu_163010 otu_143714<br>otu_22083 otu_22063 otu_69563 otu_69199 otu_70596 otu_69248 otu_69628 otu_125973<br>otu_69024 otu_73672 otu_71400 otu_73920 otu_71313 otu_162892 otu_22158<br>otu_124346 otu_22189 otu_74260 otu_22072 otu_75263 otu_70669 otu_72868 otu_70072<br>otu_72398 otu_162902 otu_69971 otu_69182 otu_70355 otu_71963 otu_70177 otu_70011<br>otu_75968 otu_73984 otu_125047 otu_74825 otu_163160 otu_70682 otu_71269<br>otu_162935 otu_70139 otu_69776 otu_125007 otu_162941 otu_124613 otu_69944<br>otu_72890 otu_95540 otu_70375 otu_69468 otu_124960 otu_150032 otu_70707<br>otu_124763 otu_69526 otu_70474 otu_72466 otu_69884 otu_22208 otu_150016<br>otu_22243 otu_69293 otu_69030 otu_69645 otu_72494 otu_69258 otu_70291 otu_69524<br>otu_69266 otu_70147 otu_74198 otu_70348 otu_69319 otu_68955 otu_124576<br>otu_124131 otu_73118 otu_69798 otu_70799 otu_162906 otu_124013 otu_22042<br>otu_124003 otu_73949 otu_72702 otu_22219 otu_68902 otu_70848 otu_73210 otu_69612<br>otu_74302 otu_69778 otu_125436 otu_71633 otu_68984 otu_74004 otu_72987 otu_69629<br>otu_71825 otu_74036 otu_72939 otu_124690 otu_71470 otu_132388 otu_71725<br>otu_69448 otu_69015 otu_74385 otu_70431 otu_163022 otu_75425 otu_69109 otu_71641<br>otu_74053 otu_71987 otu_70089 otu_71261 otu_70542 otu_22039 otu_69180 otu_70243<br>otu_70136 otu_123972 otu_69051 otu_69470 otu_74104 otu_22133 otu_162931<br>otu_71881 otu_69098 otu_68992 otu_74153 otu_162966 otu_72976 otu_145702<br>otu_69662 otu_69847 otu_73143 otu_145630 otu_162913 otu_125021 otu_70240<br>otu_70671 otu_71257 otu_74755 otu_71447 otu_124053 otu_71349 otu_70698 otu_69959<br>otu_69029 otu_69622 otu_143750 otu_71717 otu_125289 otu_75204 otu_68932<br>otu_22060 otu_72592 otu_124244 otu_162897 otu_124786 otu_71162 otu_22088<br>otu_71218 otu_69143 otu_125847 otu_70742 otu_70925 otu_70523 otu_73903<br>otu_124518 otu_125656 otu_69196 otu_163001 otu_74715 otu_162920 otu_75601<br>otu_73923 otu_74368 otu_22091 otu_72001 otu_69386 otu_72046 otu_70181 otu_70490<br>otu_70606 otu_72902 otu_69316 otu_68905 otu_71765 otu_73886 otu_22110 otu_150027<br>otu_71621 otu_73786 otu_72788 otu_22117 otu_69550 otu_69685 otu_163072 otu_70802<br>otu_69529 otu_71988 otu_163156 otu_69490 otu_72553 otu_70276 otu_105347<br>otu_69010 otu_74122 otu_68996 otu_72074 otu_70209 otu_124463 otu_69416 otu_73208<br>otu_73851 otu_22167 otu_69852 otu_69142 otu_22157 otu_74351 otu_69809 otu_163080<br>otu_125382 otu_124252 otu_70770 otu_22046 otu_74475 otu_69136 otu_70526<br>otu_22233 otu_71033 otu_22064 otu_70002 otu_72858 otu_124118 otu_70647 otu_126118<br>otu_124046 otu_73709 otu_71524 otu_70610 otu_73832 otu_124467 otu_123991<br>otu_72872 otu_22054 otu_71704 otu_124886 otu_99177 otu_22127 otu_71533<br>otu_162970 otu_163018 otu_69275 otu_69810 otu_73965 otu_70722 otu_72454<br>otu_69301 otu_22204 otu_69403 otu_125888 otu_72577 otu_69332 otu_69355<br>otu_143733 otu_69872 otu_22081 otu_22098 otu_71246 otu_70556 otu_71799 otu_73464<br>otu_72209 otu_75193
